# Molybdenum(VI) Nitrido Complexes with Tripodal Silanolate Ligands. Structure and Electronic Character of an Unsymmetrical Dimolybdenum μ-Nitrido Complex Formed by Incomplete Nitrogen Atom Transfer

**DOI:** 10.1021/acs.inorgchem.4c00762

**Published:** 2024-04-25

**Authors:** Daniel Rütter, Maurice van Gastel, Markus Leutzsch, Nils Nöthling, Daniel SantaLucia, Frank Neese, Alois Fürstner

**Affiliations:** § Max-Planck-Institut für Kohlenforschung, 45470 Mülheim/Ruhr, Germany; + Max-Planck-Institut für Chemische Energiekonversion, 45470 Mülheim/Ruhr, Germany

## Abstract

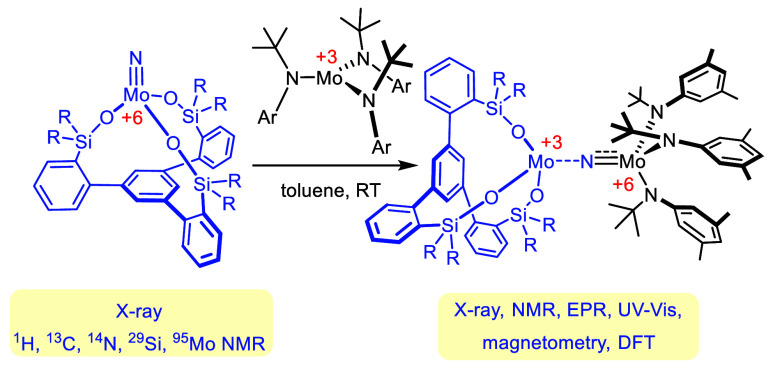

In contrast to a tungsten nitrido complex endowed with a tripodal silanolate ligand framework, which was reported in the literature to be a dimeric species with a metallacyclic core, the corresponding molybdenum nitrides **3** are monomeric entities comprising a regular terminal nitride unit, as proven by single-crystal X-ray diffraction (SC-XRD). Their electronic character is largely determined by the constraints imposed on the metal center by the podand ligand architecture. ^95^Mo nuclear magnetic resonance (NMR) and, to a lesser extent, ^14^N NMR spectroscopy allow these effects to be studied, which become particularly apparent upon comparison with the spectral data of related molybdenum nitrides comprising unrestrained silanolate, alkoxide, or amide ligands. Attempted nitrogen atom transfer from these novel terminal nitrides to [(*t*BuArN)_3_Mo] (Ar = 3,5-dimethylphenyl) as the potential acceptor stopped at the stage of unsymmetric dimolybdenum μ-nitrido complex **13a** as the first intermediate along the reaction pathway. SC-XRD, NMR, electron paramagnetic resonance, and ultraviolet–visible spectroscopy as well as magnetometry in combination with density functional theory allowed a clear picture of the geometric and electronic structure of this mixed-valent species to be drawn. **13a** is formally best described as an adduct of the type [(Mo^[O]^)^+III^–(μN)^−III^–(Mo^[N]^)^+VI^], *S* = ^1^/_2_ complex with (Mo^[O]^)^+III^ in the low-spin configuration, whereas related complexes such as [(AdS)_3_Mo–(μN)–Mo(N*t*BuAr)_3_] (**19**; Ad = 1-adamantyl) have previously been regarded in the literature as mixed-valent Mo^+IV^/Mo^+V^ species. The spin population at the two Mo centers is uneven and notably larger at the more reduced Mo^[O]^ atom, whereas the only spin present at the (μN) bridge is derived from spin polarization.

## Abstract 2

Whereas tungsten nitrido complexes with a tripodal silanolate ligand framework are dimeric species with a metallacyclic core, their molybdenum analogues are ordinary monomeric entities with terminal nitride ligands. Attempted transfer of a N atom to [Mo(N*t*BuAr)_3_] as the acceptor furnished an unsymmetrical mixed-valent dinuclear μ-nitrido complex, the electronic structure of which was determined by a combined experimental and computational approach.

## Introduction

**Figure 1:**
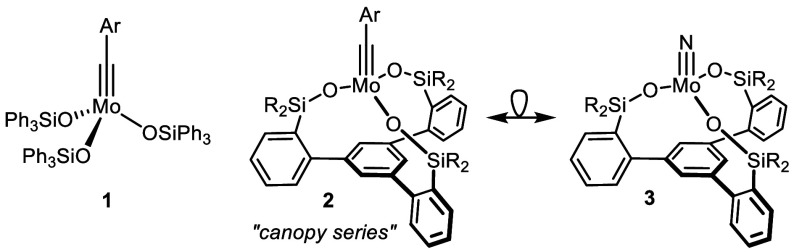
Standard catalysts for alkyne metathesis and targeted isolobal nitrido complexes.

Molybdenum alkylidynes such as **1** endowed with silanolate ligands are arguably (among) the most active and selective known catalysts for alkyne metathesis (Figure 1).^1−4^ Although they have withstood numerous tests in material science and target-oriented synthesis,^5−8^ substrates bearing unhindered (primary) alcohols marked one of the few serious limitations; such compounds may eventually react with the complex, replace the silanolates, and, in doing so, efface catalytic activity. To remedy the issue, we have recently designed a set of tripodal silanolate ligands to harness the chelate effect;^9−12^ the same ligand design was independently pursued by Lee and co-workers.^13,14^ Indeed, “canopy” catalysts of type **2** are more compatible with protic groups and were found to apply to a number of challenging substrates beyond the reach of parent complex **1**.^10^ At the same time, **2** and congeners are distinguished by a remarkable tolerance for Lewis basic functionality, even though they comprise an early transition metal in its highest oxidation state.^10,15−20^

These virtues derive from the synergy between the ancillary silanolates and the high-valent molybdenum alkylidyne unit, which has been studied in detail in the past.^10,14,21−23^ As a next step, investigating whether these tripodal silanolates also match molybdenum complexes beyond the alkylidyne estate seemed interesting and relevant. Nitrido complexes are obvious candidates, as they are isolobal with the alkylidynes.^24,25^ As such, they are of interest in their own right and, at the same time, constitute a potential entry point into the realm of low-valent molybdenum silanolate coordination chemistry.^26,27^ The results of our first foray are summarized below.

## Results and Discussion

### Tripodal Molybdenum Nitrido Complexes

**Scheme 1:**
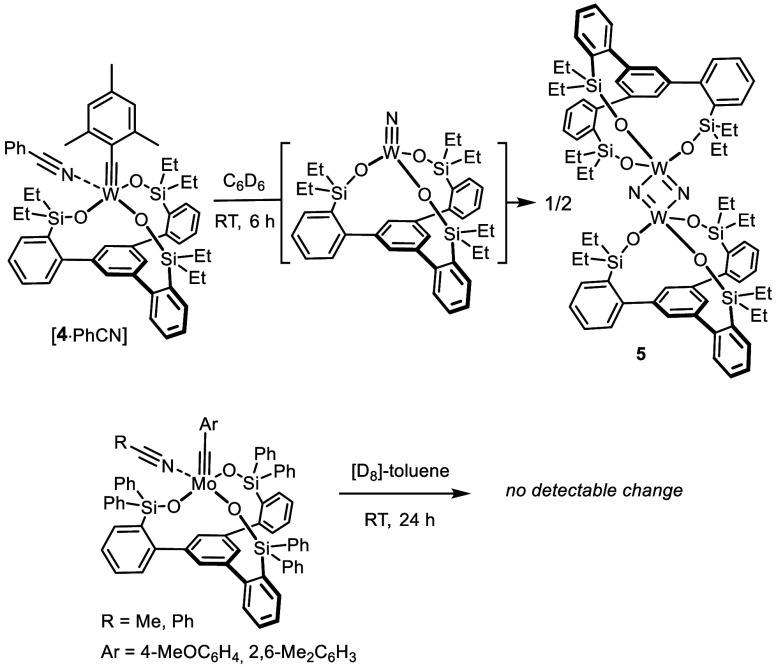
A Dimeric Tungsten Nitride Complex with Tripodal Silanolate Ligands Formed by Stoichiometric Nitrile Metathesis^14^^,^ The related molybdenum alkylidynes basically lack this reactivity.

Lee and co-workers reported that benzonitrile adduct [**4**·PhCN] of a tripodal tungsten alkylidyne transforms at ambient temperature into the corresponding tungsten nitride **5** by a stoichiometric nitrile metathesis reaction (Scheme 1).^14,28,29^ In contrast to many other well-characterized [X_3_W≡N] complexes,^30,31^**5** is a dimeric species with a metallacyclic core comprised of alternating W=N and W–N bonds. It is not clear whether this constitution is enforced by the constraints of the podand ligand framework;^14^ if so, the corresponding molybdenum nitrides might also be dimeric entities.^32−34^

To clarify the issue, a flexible entry into Mo(VI) nitrido complexes endowed with tripodal silanolate ligands was sought. Nitrile metathesis, however, is not the way forward.^35^ Actually, the reverse reaction is favored, which allows certain molybdenum nitrides to be converted into the corresponding alkylidynes upon their treatment with a sacrificial alkyne.^36,37^ Whereas the highly Lewis acidic W(VI) center favors binding to a nitride over an alkylidyne on thermodynamic grounds, Mo(VI) shows the opposite preference.^38^ Control experiments confirmed this notion in that solutions of the nitrile adducts of **2** were found to be stable for extended periods of time (Scheme 1).^9,10^

**Scheme 2:**
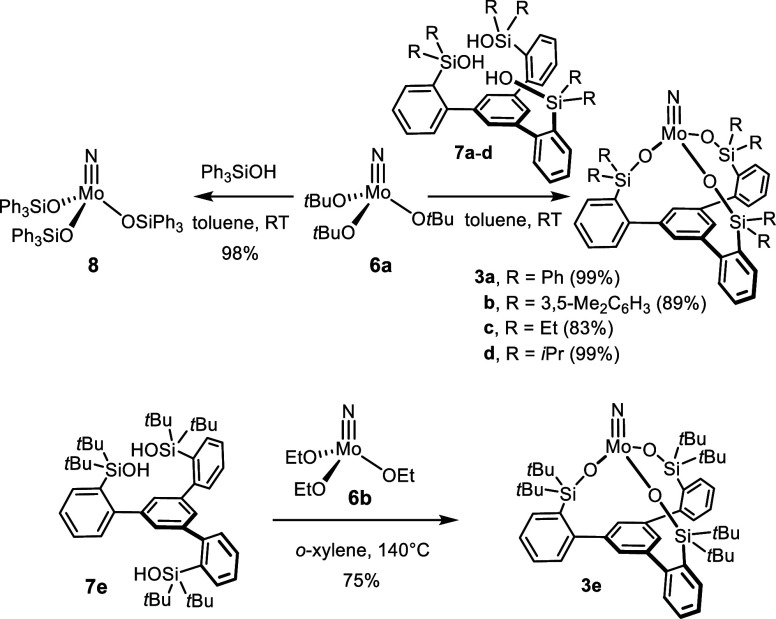
Preparation of the Podand Trissilanolate Molybdenum Nitrido Complexes

**Figure 2:**
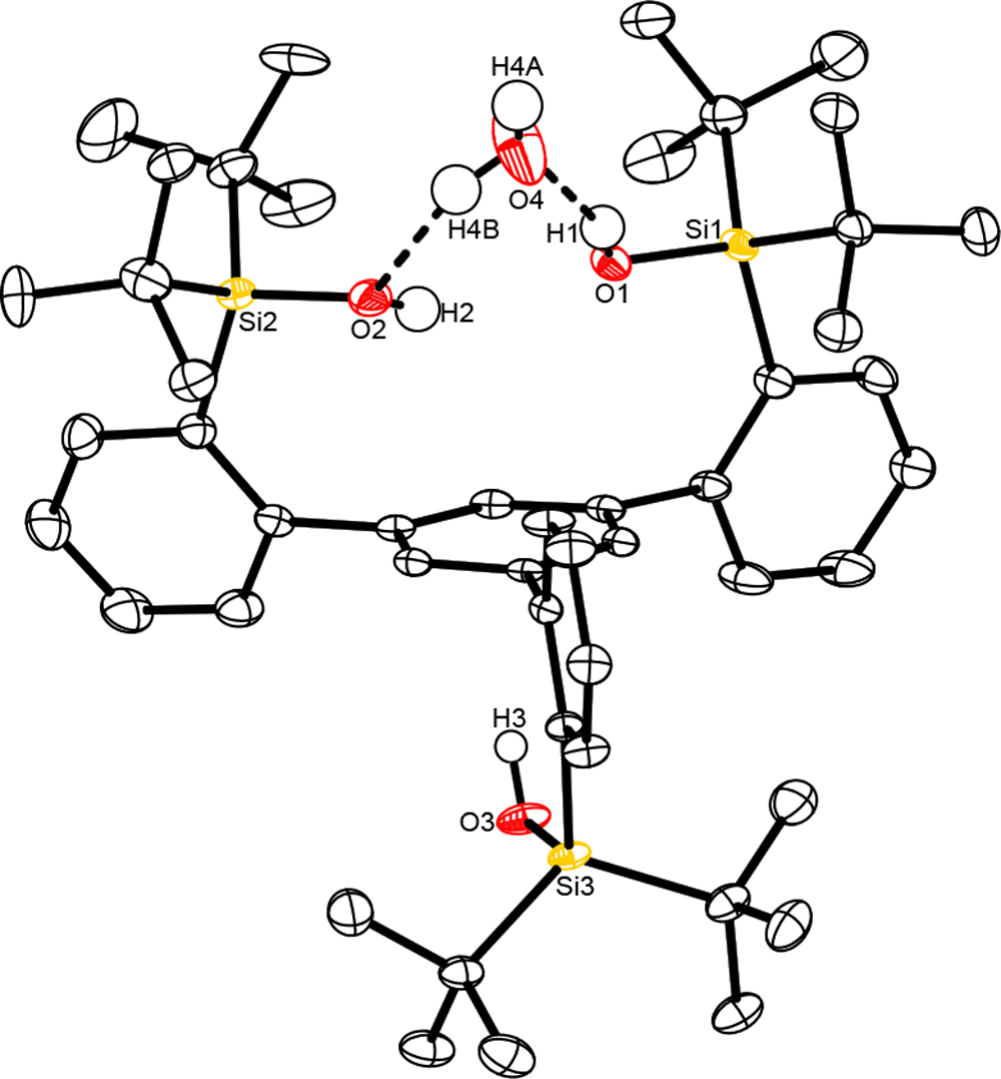
Structure of the monohydrate of ligand **7e** in the solid state. All H atoms (except those of the Si–OH groups and the co-crystallized water molecule), the hexamethyldisiloxane solute in the unit cell, and the disordered parts of the structure have been omitted for the sake of clarity. The full structure is available in the Supporting Information.

The targeted podand molybdenum nitrido complexes can be readily accessed by ligand exchange, which takes advantage of the fact that silanols are generally more acidic than ordinary alcohols (Scheme 2).^39^ Thus, treatment of literature-known precursor complex **6a**(40) with Ph_3_SiOH in toluene at ambient temperature furnished [(Ph_3_SiO)_3_Mo≡N] (**8**) in essentially quantitative yield.^41,42^ Tripodal ligands **7a**–**d** are equally well behaved, affording targeted podand complexes **3a**–**d**, respectively, without oligomerization competing to any notable degree. The ease of chelate formation likely reflects the favorable preorganization of these ligands; they have previously been shown to adopt favorable conformations in that all three silanol headgroups are “upward/inward” oriented as a result of mutual hydrogen bonding interactions between the individual −SiO–H units.^10,11^ Only in the case of **7e** bearing bulky *tert*-butyl substituents on silicon does steric hindrance come into play (for the preparation of this ligand, see the Supporting Information). Specifically, line broadening in the nuclear magnetic resonance (NMR) spectra indicates dynamic behavior on the NMR time scale. When the the sample is cooled to 233 K, two distinct conformers are present in solution, the major one of which is non-*C*_3_ symmetric; this conformer is captured upon crystallization, which is evident from the structure of **7e** in the solid state, in which one silanol side arm is pointing “downward” (Figure 2).^43^ On the contrary, the sharp signal sets observed when the NMR spectra were recorded at 353 K indicate a *C*_3_-symmetric conformation at this temperature (for details, see the Supporting Information). In line with this notion, the formation of the corresponding podand complex **3e** mandated heating; the best results were obtained by stirring of a mixture of **7e** and the slimmer molybdenum nitrido precursor complex **6b**(40) in refluxing *o*-xylene overnight.

**Figure 3:**
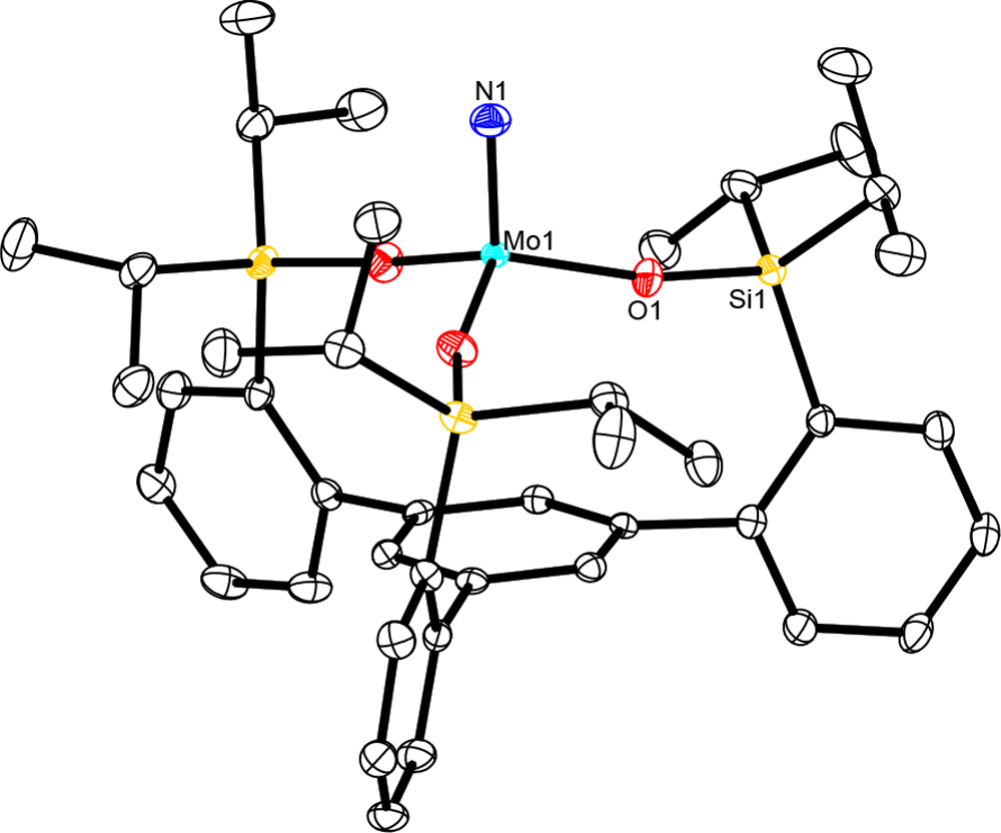
Structure of complex **3d** in the unit cell. All H atoms have been omitted for the sake of clarity. The full structure is available in the Supporting Information.

**Figure 4:**
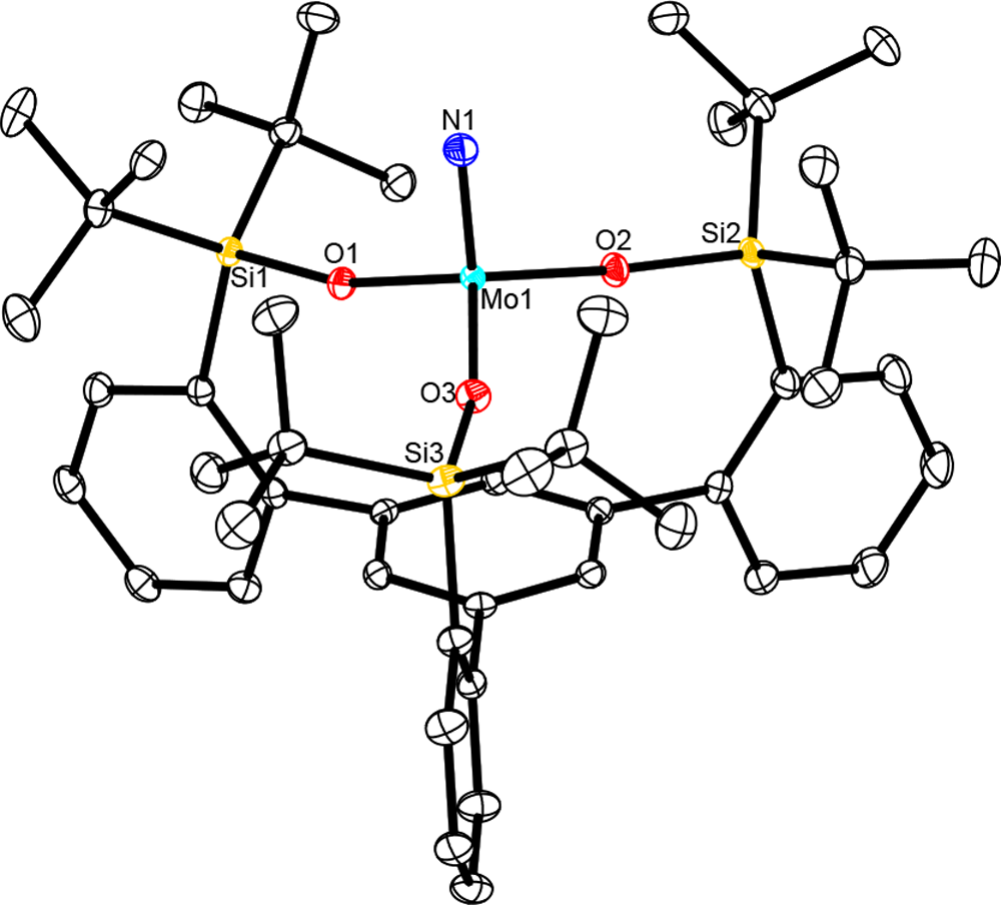
Structure of complex **3e** in the solid state. All H atoms and disordered solute Et_2_O in the unit cell have been omitted for the sake of clarity. The full structure is available in the Supporting Information.

Complexes **3d** and **3e** are ordinary monomeric terminal nitrides (Figures 3 and 4, respectively). Although an X-ray crystal structure of **3c** with ethyl substituents on the silicon tethers has not been obtained for direct comparison with tungsten complex **5** reported by the Lee group,^14^ we have every reason to believe that this species is also monomeric. Strong evidence is provided by ^95^Mo NMR spectroscopy,^44,45^ which has recently been shown to be a particularly useful probe for the alkylidyne series.^9−11,23^ The fact that the ^95^Mo NMR shifts of **3a**–**e** are almost identical (116 ppm ≤ δ_Mo_ ≤ 121 ppm) makes a different aggregation state highly improbable and shows that the lateral substituents on silicon exert hardly any influence. It is the tripodal ligand scaffold that defines the electronic structure at large. Specifically, the geometric constraints enforce a characteristic stretching of the Mo–O–Si linkages [in **3e** for example, 165.60(8)°, 165.60(9)°, and 168.14(9)°], very similar to what has previously been observed in the alkylidyne series.^10,11^

**Figure 5:**
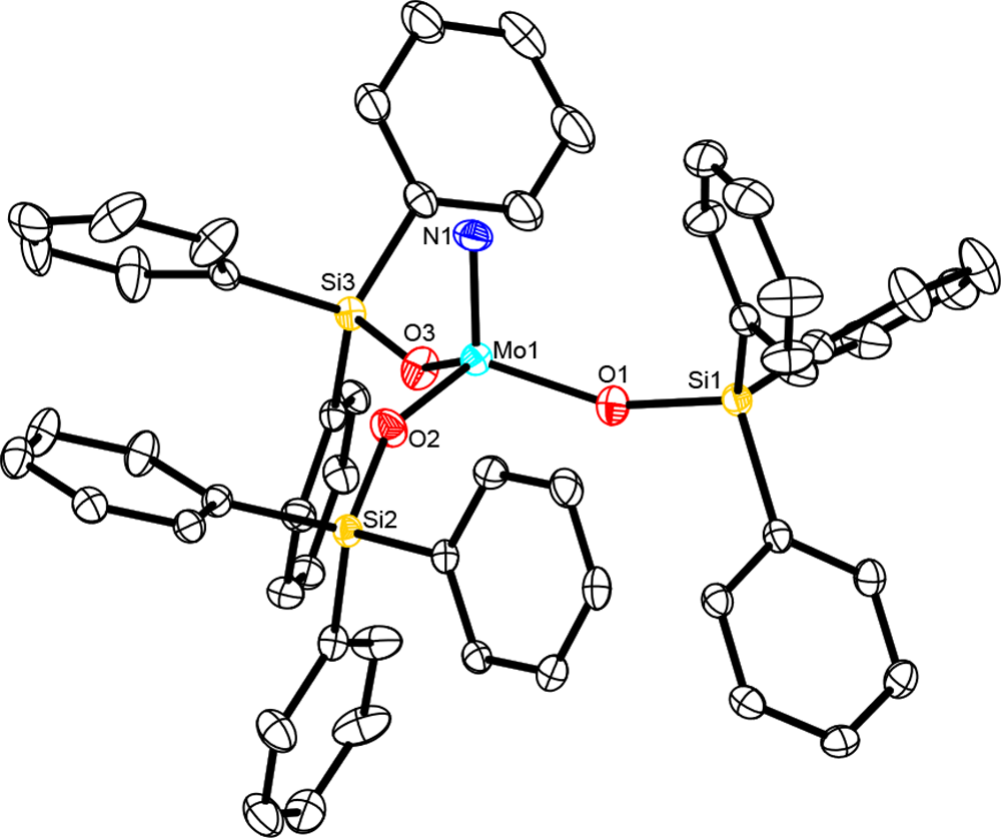
Structure of complex **8** in the solid state. All H atoms and the disorder of one of the phenyl rings have been omitted for the sake of clarity. The full structure is available in the Supporting Information.

**Figure 6:**
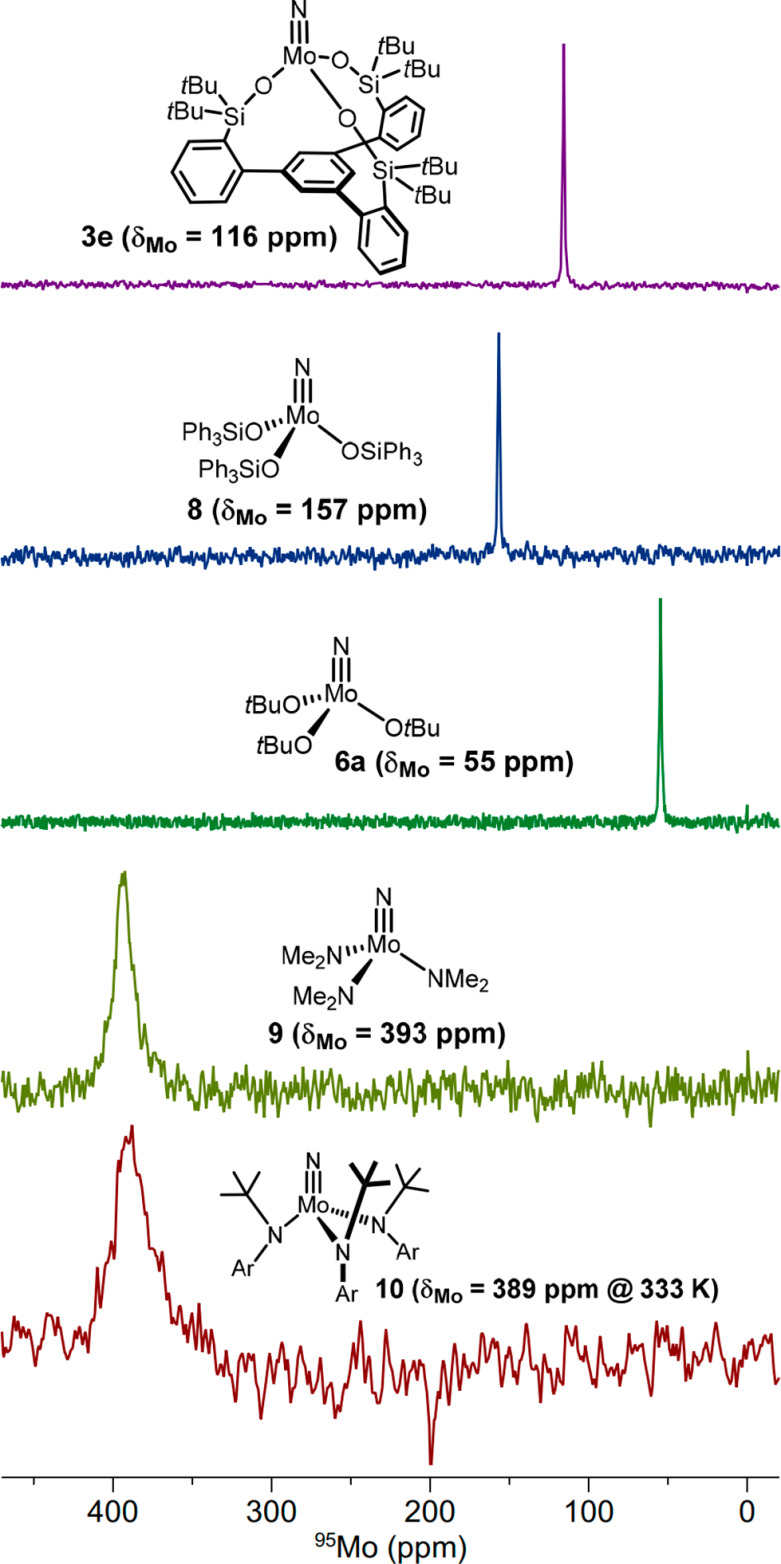
^95^Mo NMR spectra [*d*_8_]-toluene, 298 K (except for **10**)] of selected molybdenum nitrido complexes. Ar = 3,5-dimethylphenyl.

**Figure 7:**
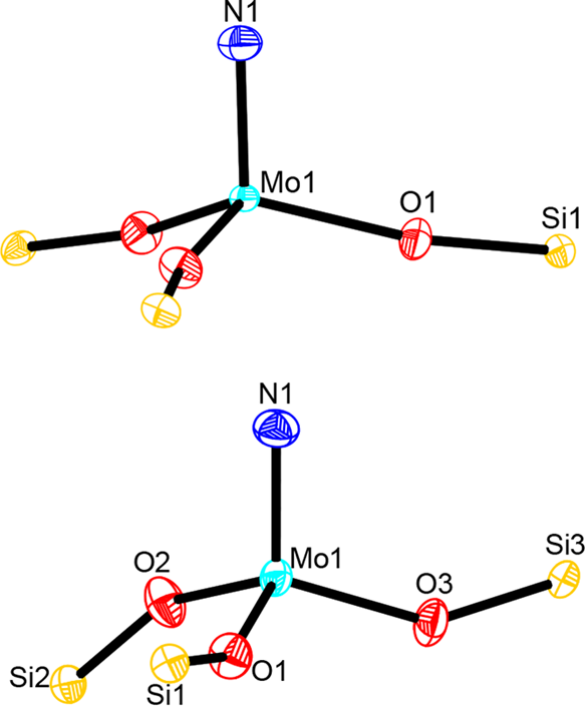
Different curvature of the cores of tripodal nitride complex **3d** (top) (one of three molecules in the unit cell; the N–Mo–O–Si torsion angles of the three independent molecules are −64.68°, −70.66°, and −51.91°) and parent complex [(Ph_3_SiO)_3_Mo≡N] (**8**) (bottom) (the three different N–Mo–O–Si torsion angles of **8** are 16.40°, −155.97°, and −59.20°). The color code is the same as that used in Figures 3–5.

As the donor capacity of the oxygen lone pairs is strongly dependent on the angle,^22,23^ this conserved curvature resulting from the ligand architecture is deemed the critical determinant for why the metal centers of **3a**–**e** can hardly be distinguished by ^95^Mo NMR. Under this proviso, relaxation of the ligand backbone should entail notable shift differences (Figure 5). In line with this notion, the otherwise very similar and also monomeric complex [(Ph_3_SiO)_3_Mo≡N] (**8**) carrying monodentate silanolates has a distinct ^95^Mo NMR shift of 157 ppm (Figure 6); the X-ray crystal structure of this complex shows that the curvature of the core in the solid state is in fact different from that of **3d**. Specifically, the Mo–O–Si bond angles are less obtuse [148.06(9)°, 153.62(10)°, and 154.64(9)°], with two “legs” pointing “upward” and one being “downward” oriented (Figure 7).^46,47^ Even more profound consequences are expected if the type of ancillary ligand is changed: indeed, precursor complex **6a** endowed with three alkoxides has a ^95^Mo shift of 55 ppm,^39^ whereas the Mo centers of the nitrido complexes [(Me_2_N)_3_Mo≡N] (**9**; δ_Mo_ = 393 ppm)^48−50^ and [(*t*BuArN)_3_Mo≡N] (**10**; δ_Mo_ = 389 ppm)^51^ bearing amide ligands of different steric demands both resonate at a notably lower field.

**Figure 8:**
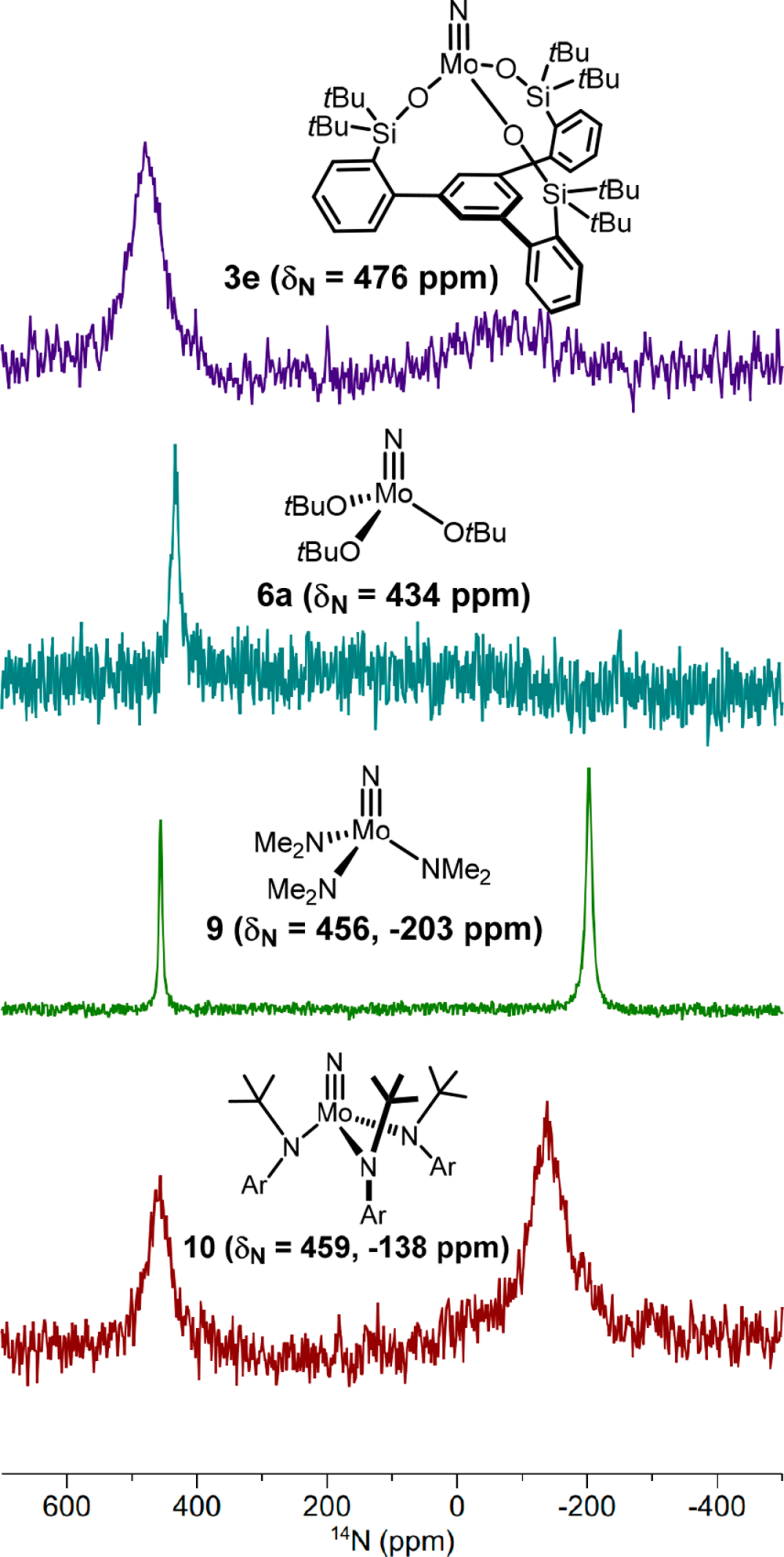
^14^N NMR spectra (*d*_8_-toluene, 333 K) of selected molybdenum nitrido complexes. Ar = 3,5-dimethylphenyl.

For the high symmetry of the terminal nitride complexes of this series, ^14^N NMR spectroscopy might also qualify for their study. The method has the advantage of saving time-consuming and expensive labeling as the ^14^N isotope is abundant, though quadrupolar.^52^ Interestingly, the recorded shifts fall into a quite narrow range (434–476 ppm), independent of whether the complexes carry alkoxides, amides, or tripodal silanolates (Figure 8). Whereas the ^95^Mo NMR shifts of **3e** and **10** are no less than 273 ppm apart, their ^14^N NMR shifts (476/459 ppm) are very similar, especially if one takes the massive line widths into consideration. Therefore ^14^N NMR turns out to be a less sensitive probe for the electronic structure upon variation of the ligand sphere than ^95^Mo NMR. While a better understanding of these effects mandates future in-depth computational investigations, the trends reinforce our general impression gained in several independent studies that transition metal NMR spectroscopy is a truly powerful yet still largely underutilized tool for organometallic chemistry and homogeneous catalysis.^10,23,53−55^

### Incomplete Nitrogen Transfer

**Scheme 3:**
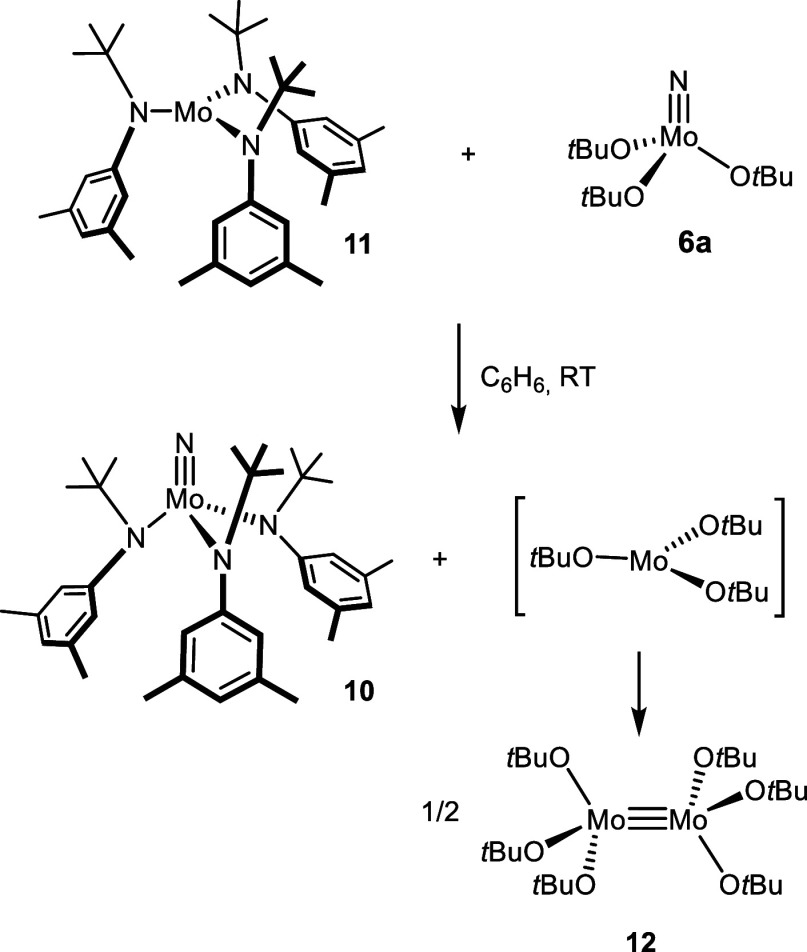
Literature Redox Couple Resulting in Nitrogen Atom Transfer^60^

[(*t*BuArN)_3_Mo] not only is famous for its spectacular ability to cleave dinitrogen (and other small molecules such as N_2_O) under mild conditions^50,56−59^ but also was found to react with [(*t*BuO)_3_Mo≡N] (**6a**) in a benzene solution at 28 °C to give [(*t*BuArN)_3_Mo≡N] (**10**) and 0.5 equiv of [Mo_2_(O*t*Bu)_3_] (**12**) (Scheme 3).^60^ This remarkably facile nitrogen atom transfer is driven, at least in part, by the formation of the thermodynamically favorable and kinetically rather inert Mo≡Mo bond of **12**,^61,62^ by which the initially formed monomeric [(*t*BuO)_3_Mo] fragment becomes trapped. Although only transiently present, labeling studies showed that this fleeting tricoordinate species engages very effectively in dinitrogen splitting.^60^

**Scheme 4:**
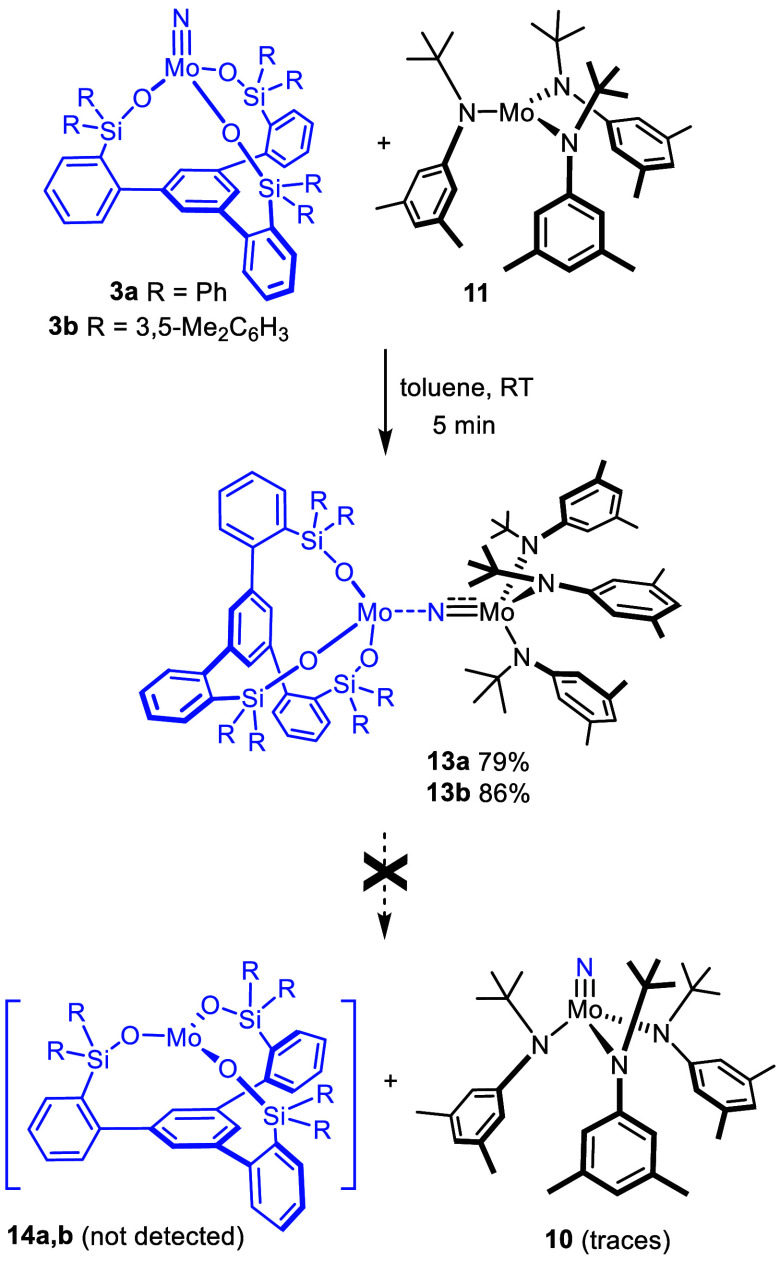
Incomplete Nitrogen Transfer Reaction Stopping at a Stable Adduct The drawn bond orders in **13** reflect the analysis of the localized orbitals (see below).

With this and related precedent in mind,^63,64^ it was tempting to study whether nitrides of type **3** endowed with tripodal silanolates are amenable to analogous nitrogen transfer processes (Scheme 4).^65^ If one extrapolates the rationale for the different propensity of tungsten versus molybdenum alkylidynes to undergo stoichiometric nitrile metathesis (see above) to the projected N transfer process and takes the ^95^Mo NMR shifts as a proxy for the Lewis acidity of the metal centers,^66^ the projected abstraction of the N atom of **3** by **11** seems possible. Qualitatively, the nitrido ligand should be more firmly bound in the resulting product **10** than in **3**, as the former complex is the more deshielded of the two. However, it was unclear at the outset if putative tripodal Mo(III) fragment **14** that is supposed to be concomitantly formed would be able to dimerize in view of the “fence” of lateral substituents about the central atom held in an upright position by the podand ligand backbone.^67^ Should formation of a Mo≡Mo bond be precluded on steric grounds, the thermodynamic driving force for N atom transfer might be insufficient.^68^

Treatment of **3a** and **3b** with **11** in toluene at ambient temperature resulted in the instant formation of new paramagnetic products, which were identified as highly air-sensitive μ-nitrido complexes **13a** and **13b**, respectively (Scheme 4).^69^ All attempts to force the projected nitrogen transfer to proceed beyond this stage were largely met with failure, although small amounts of targeted complex **10** (and free ligand) were detected in the samples by NMR spectroscopy (for details, see the Supporting Information).

**Figure 9:**
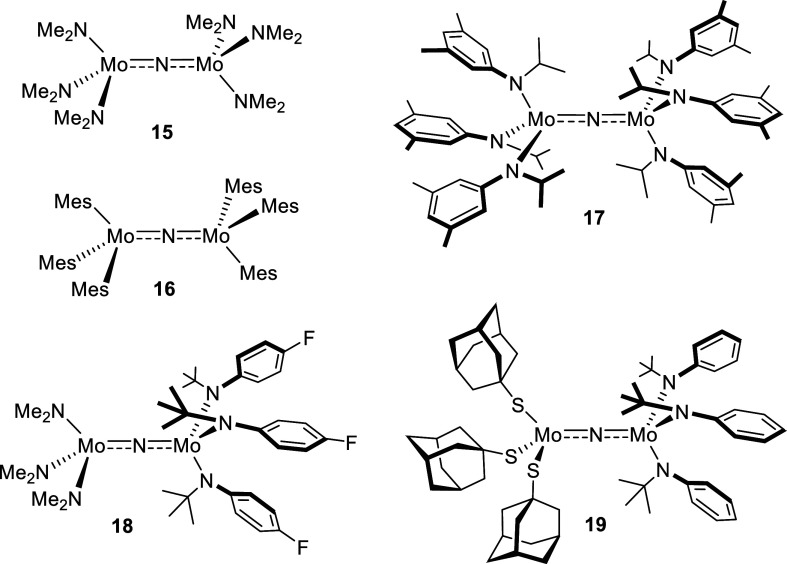
Literature-known dimolybdenum μ-nitrido complexes. Because detailed analyses are missing (see the text), broken bond orders are arbitrarily drawn.

A small number of related μ-bridged dimolybdenum complexes have been described in the literature (Figure 9);^51,70−73^ their role as the first intermediates involved in nitrogen atom transfer was unambiguously confirmed. Because canonical Lewis structures might not capture the bonding situation correctly, interesting questions about the actual electronic structure do arise. This is particularly true for those rare cases in which the two molybdenum fragments linked by the N atom are non-equivalent. Such complexes are mixed-valence species; specifically, adduct **19** was seen as a Mo^+IV^/Mo^+V^ complex, whereby the asymmetry of the μ-nitrido bridge manifested in the X-ray crystal structure was taken as an indication that the [Mo(N*t*BuAr)_3_] fragment comprises the more highly oxidized of the two metal centers.^72^ This conclusion was later supported by density functional theory (DFT) calculations on heavily truncated model system [(HS)_3_Mo(μ-N)Mo(NH_2_)_3_].^74^ The same study, however, issued the warning that the nature of the ancillary ligands capping both ends is a critical determinant of the exact electronic character of complexes of this type; truncation is, hence, potentially misleading.

Complex **13a** provided an opportunity to revisit this intricate question. We set out to draw a detailed picture of the electronic structure of this unsymmetrical dimolydenum μ-nitrido complex by a combined experimental and theoretical approach without resorting to any truncations of the ligand sphere in the computations.

### Structure in the Solid State and in Solution

**Figure 10:**
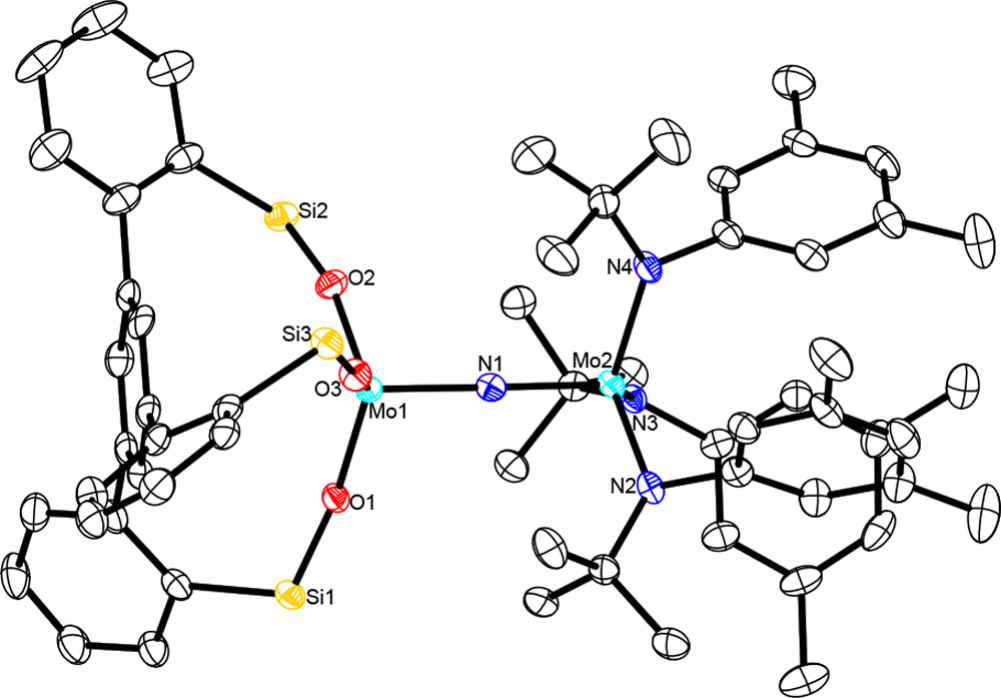
Truncated structure of complex **13a** in the solid state. The phenyl substituents on the silicon atoms of the tripodal ligand about Mo1, all H atoms, and solvate molecules in the unit cell have been omitted for the sake of clarity. The full structure is available in the Supporting Information.

Single crystals of **13a** and **13b** suitable for X-ray diffraction were grown by vapor diffusion of pentane into a solution of the complexes in C_6_D_6_ and toluene, respectively. Their structures in the solid state are very similar (Figure 10; for the X-ray structure of **13b**, see the Supporting Information). In both cases, the Mo^[N]^–(μN)–Mo^[O]^ axis is almost perfectly linear [**13a**, Mo1–N1–Mo2, 178.56(18)] but notably unsymmetrical (in Mo^[X]^, the symbol X indicates the heteroatoms bound to the specific Mo center). Specifically, the Mo^[O]^–(μN) distance [1.838(3) Å] is notably longer than the Mo^[N]^–(μN) distance [1.808(3) Å]; this fact is reminiscent of the asymmetric core of the thiolate-containing heterodimeric complex **19** mentioned above.^72^ The coordination sphere of both molybdenum atoms of **13** is closer to trigonal-pyramidal than tetrahedral; the peripheral ligands capping the ends of the Mo–(μN)–Mo core adopt a staggered conformation relative to each other.

The electronic structure of **13**a was analyzed using DFT calculations using the B3LYP functional^75,76^ and the def2-TZVP basis set^77^ and effective core potentials at the molybdenum atoms.^78^ All calculations were carried out with the ORCA program suite, version 5.0.4.^79−82^ All structures were fully optimized without constraints and confirmed to be minima on the potential energy surface through frequency calculations that showed only one negative mode at −9 cm^–1^ that was considered to be numerical noise. The experimentally determined geometric features are all well reproduced by the geometry-optimized computed structure of **13a**, including the asymmetry of the Mo−μN bonds (Table 1; for the computed structure, see the Supporting Information).

**Table 1:** Comparison of Selected Bond Distances (angstroms) and Angles (degrees) of Complex **13a** from the X-ray Crystal Structure (SC-XRD) and the Geometry-Optimized Computed Structure (B3LYP functional, Def2-TZVP basis set)

	SC-XRD	optimized structure
*d*(Mo^[O]^–μN)	1.838	1.842
*d*(Mo^[N]^–μN)	1.808	1.780
*d*(Mo^[O]^–O)	1.910	1.907
	1.912	1.915
	1.920	1.927
*d*(Mo^[N]^–N)	1.970	1.978
	1.966	1.968
	1.968	1.970
∠(Mo^[N]^–μN–Mo^[O]^)	178.6	178.1
∠(μN–Mo^[O]^–O)	102.7	101.9
	102.9	100.3
	102.3	103.0
∠(μN–Mo^[N]^–N)	103.4	105.4
	104.6	105.0
	105.5	105.2

As expected, complex **13a** is paramagnetic; the closer the atoms are to the central Mo–(μN)–Mo axis, the stronger their paramagnetic shifts. Due to its exceptional sensitivity toward air and moisture, the NMR spectra recorded in C_6_D_6_ always contained traces of diamagnetic impurities, which were identified as free aniline ligand HN(*t*Bu)Ar and terminal nitrido complex [(*t*BuArN)_3_Mo≡N] (**10**) (Ar = 3,5-dimethylphenyl).^48^ This complication notwithstanding, all signals could be assigned and the expected large temperature dependence of the spectra could be demonstrated by variable-temperature NMR (see the Supporting Information). In solution, the tripodal silanolate ligand of **13a** itself is *C*_3_-symmetric on the NMR time scale, whereas the pairs of peripheral phenyl groups on the silicon linkers are diastereotopic.

### Electronic Structure

We first investigated whether complex **13a** has to be described as a Mo^IV^/Mo^V^, 4d^2^/4d^1^ complex with three unpaired electrons (high-spin *S* = 1, 4d^2^ Mo^IV^ antiferromagnetically coupled to an *S* = ^1^/_2_ 4d^1^ Mo^V^) or whether it is best viewed as containing only one unpaired electron (either Mo^III^/Mo^VI^ or Mo^IV^/Mo^V^ with low-spin, *S* = 0 Mo^IV^). Therefore, three calculations have been performed: (i) a high-spin, *S* = ^3^/_2_, calculation, (ii) a broken-symmetry, *M*_S_ = ^1^/_2_ calculation starting from the high-spin electronic configuration and then flipping the spin of one electron, and (iii) a low-spin *S* = ^1^/_2_ calculation. The low-spin and broken-symmetry calculation converged to the same electronic energy. The expectation value of the *S*^2^ operator was 0.952, which is slightly above the theoretically expected value of 0.75 for a pure doublet. The quartet was found to be 49 kJ/mol higher in energy than the doublet state. The expectation value of the *S*^2^ operator was 3.797, close to the value of 3.75 for a pure quartet state. Thus, energetically an *S* = ^1^/_2_ ground state appears to be favored over electronic configurations with a Mo^IV^ in a local high-spin state. Below, we investigate the electronic structure of this state in more detail and reconcile it with the measured spectroscopic properties.

To visualize the frontier orbital structure, it is often more instructive to examine localized molecular orbitals (LMOs) rather than canonical molecular orbitals. The latter diagonalize the Fock operator and have associated energies but tend to be rather delocalized, which may obscure the character of the chemical bonds. By contrast, localized orbitals (LMOs) can be obtained by a unitary transformation of the occupied orbitals that leaves the overall wave function unchanged but more clearly exposes the chemical nature of bonding. The LMOs resemble the σ- and π-bonds and lone pairs one draws in resonance structures. While these orbitals do not have well-defined orbital energies and can therefore not be plotted on an energy scale in a typical Aufbau manner, they are chemically intuitive and easy to interpret (for a complementary canonical MO scheme of **13a** with orbital energies, see the Supporting Information). To cleanly separate the doubly occupied orbitals from the singly occupied MO, the quasi-restricted orbital (QRO^83,84^) transformation was applied and the localization was performed only in the doubly occupied space. Importantly, the singly occupied MO (SOMO) is not subject to localization because it spans a one-dimensional space (of one singly occupied orbital). A requisite for the application of this analysis is that the electronic structure is qualitatively correctly described by a single determinant, which is the case when there is no spin coupling between the metal centers, which is the case for **13a**.

**Figure 11:**
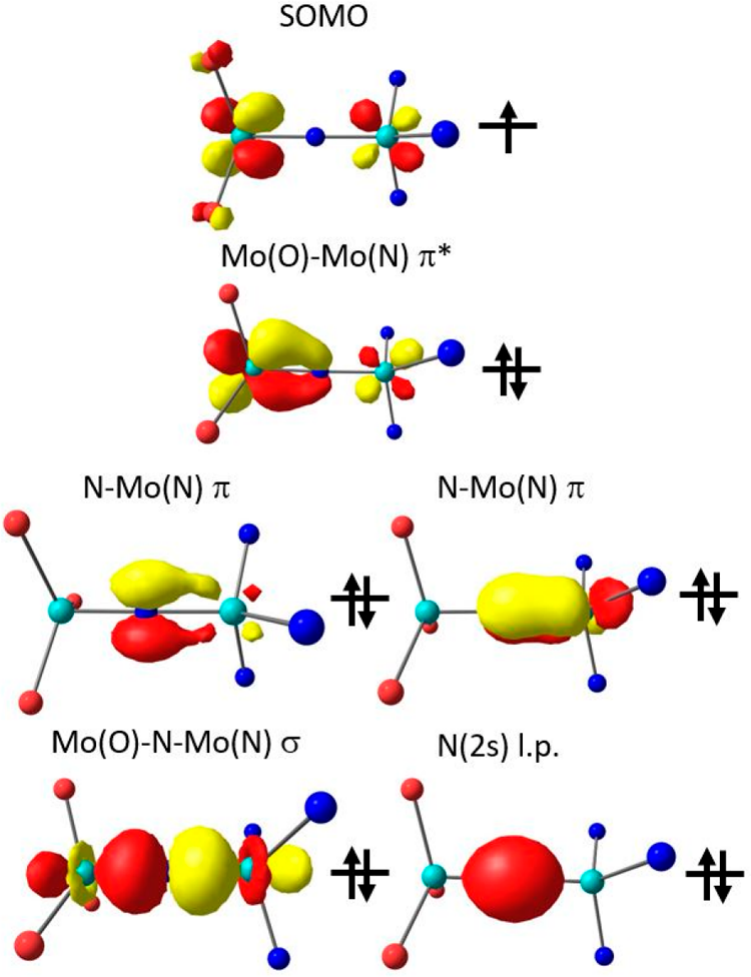
SOMO of **13a** (top) and five localized QROs of the Mo^[N]^–(μN)–Mo^[O]^ core of this complex showing the σ- and π-bonds with the bridging ligand and a Mo–Mo π* bond (bottom). Color code: light blue for molybdenum, blue for nitrogen, and red for oxygen.

The plot of the singly occupied natural orbital of **13a** shows that the unpaired electron is unevenly delocalized over both molybdenum-based 4d orbitals (Figure 11, top). The unrestricted Löwdin spin populations of Mo^[O]^ (91%) and Mo^[N]^ (2%) reflect this electronic asymmetry (Table 2). The bridging nitrogen atom is not involved; hence, the antibonding interaction between the respective Mo 4d orbitals comprising the SOMO is very weak.

**Table 2:** Spin Populations and Löwdin Charges of **13a**

	Löwdin charge	Löwdin spin
Mo^[O]^	0.52	0.91
Mo^[N]^	0.56	0.02
μN	–0.31	–0.08

In total, 11 electrons, eight from N^3–^ and three from both molybdenum atoms, are required to describe the bonding situation of the Mo^[N]^–(μN)–Mo^[O]^ moiety. They may be visualized by a total of five LMOs centered on the core atoms (Figure 11, bottom) and the SOMO (Figure 11, top). One doubly occupied LMO is similar in appearance to the SOMO and describes a second π-antibonding interaction between the Mo atoms. Both orbitals have the largest contribution arising from Mo^[O]^. In contrast to the SOMO that has no nitrido contribution, this LMO features a weak π bonding interaction between Mo^[O]^ and the nitrido ligand. Two LMOs are clearly recognized as Mo^[N]^–(μN) π-bonding orbitals. The σ-orbital (Figure 11, bottom, lower left) is delocalized over all three atoms and thus represents a three-center, two-electron σ-bond. Finally, the μN 2s orbital is essentially not hybridized and represents a nitrogen-centered lone pair. The formal Mo^[N]^–(μN) and Mo^[O]^–(μN) bond orders amount to 2.5 and 0.5, respectively, which are in qualitative agreement with the computed short Mo^[N]^–(μN) bond [1.780 Å (Table 1)] and the notably longer Mo^[O]^–(μN) distance [1.842 Å (Table 1)]. For comparison, the respective Mayer bond orders are 1.55 and 1.18, respectively, thus following the same trend. In addition, the Mayer bond order analysis picks up on the two-electron, three-center σ-bond (Figure 11) and gives a direct Mo^[N]^–Mo^[O]^ bond order of 0.49.

The spin-unrestricted B3LYP calculation gives an ⟨*S*^2^⟩ value of 0.95, which slightly deviates from the expected value of 0.75 for a pure spin doublet state. Hence, the calculation shows a limited amount of spin contamination. The contamination largely stems from the spin polarization of the central nitrogen atom that carries a −8% Löwdin spin population. An analysis of unrestricted corresponding orbitals (UCOs)^85^ shows that all electrons are paired (α–β overlap equals 1), and one electron pair has an α–β overlap of 0.90. This imperfect, polarized pair largely spots onto the SOMO–1 and the Mo–Mo π* LMO in Figure 11. The α and β UCOs for this pair are largely equal; however, the β orbital contains additional N 2p character, whereas the α orbital does not, thus leading to the net observed negative spin polarization. In more chemical terms, the calculations suggest that the more electron-donating amide ligands at Mo^[N]^ give rise to a stabilization of a higher oxidation state and increased propensity for π backbonding in the Mo^[N]^–μN moiety as compared to the siloxide-ligated Mo^[O]^ atom. As a result, the nitrido interacts significantly more strongly with Mo^[N]^ than with Mo^[O]^. The three Mo-based valence electrons are associated with the SOMO and SOMO–1, which both have the highest density at Mo^[O]^, thus leading to the formal charge distribution [(Mo^[O]^)^+III^–(μN)^−III^–(Mo^[N]^)^+VI^], *S* = ^1^/_2_ with the bulk spin at Mo^[O]^ in the low-spin configuration. The actual distribution, represented by the Löwdin values in Table 2, is of course much more even-tempered, largely owing to the delocalization of the orbitals, to the point where the Löwdin charges of both Mo atoms are approximately equal.

### Ultraviolet–Visible (UV–vis) Spectroscopy

**Figure 12:**
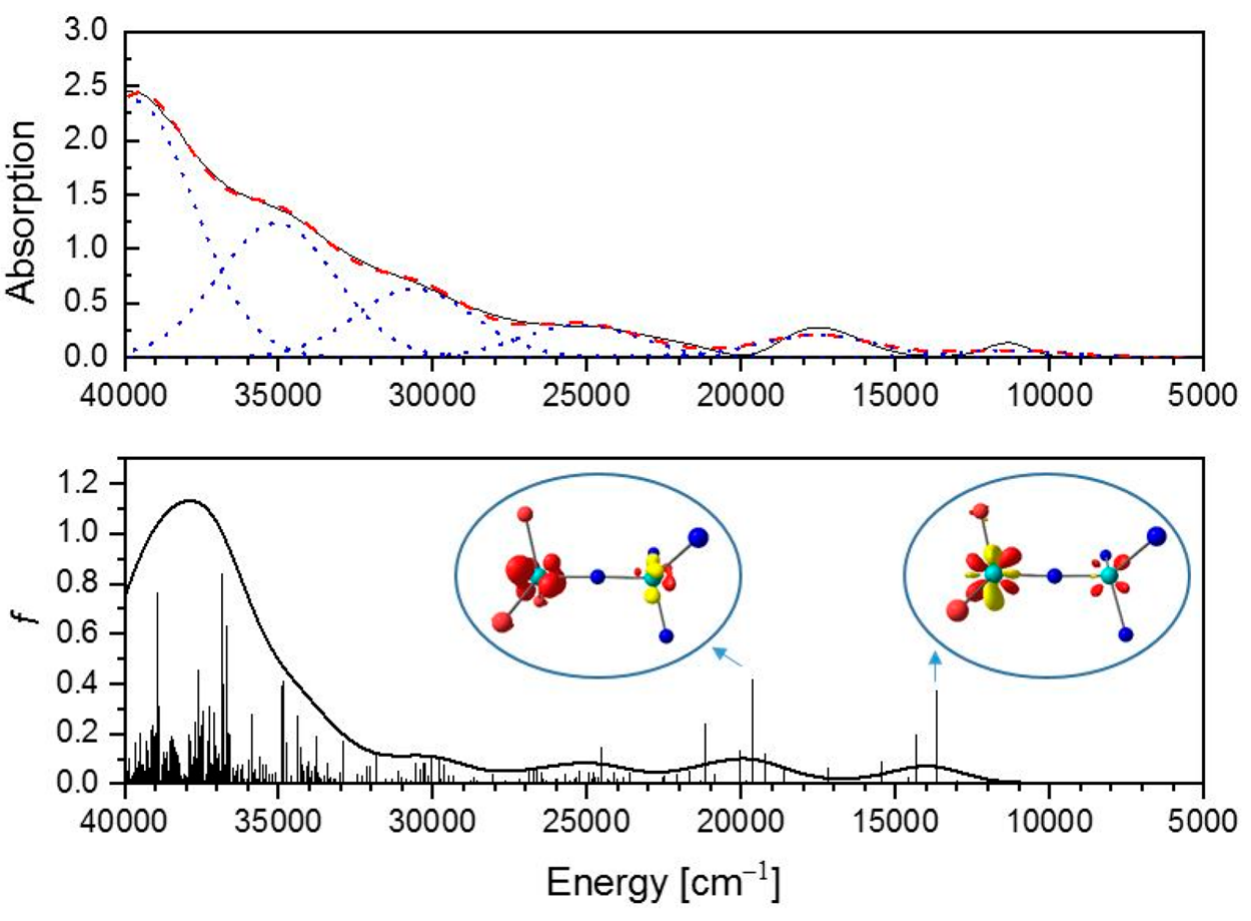
UV–vis measurement of a mull of **13a** in paraffin oil and Gaussian deconvolution in individual Gaussians (blue) and sum (red) (top). TDDFT calculation (bottom). Difference density plots, i.e., the electron density of the acceptor state minus the electron density of the donor state for the transitions at 13 661 and 19 642 cm^–1^, representative for the bands at 11 396 and 17 523 cm^–1^, respectively, are shown as insets (positive density is colored yellow, and negative density red).

Given the extreme sensitivity of complex **13a** to oxygen and moisture, the UV–vis spectrum had to be measured in a mull using paraffin oil. The spectrum exhibits two isolated bands at 11 030 and 17 400 cm^–1^ of different line width, as well as a set of poorly resolved bands in the range of 20 000–40 000 cm^–1^ (Figure 12). The bands differ in line width by a factor of almost 3, but the overall agreement with the computed UV–vis spectrum from a TDDFT calculation is nevertheless good (Figure 12, bottom). We are thus confident that the calculation presented in the previous section describes the electronic features of complex **13a** reasonably well. A Gaussian deconvolution of the UV–vis spectrum in paraffin oil gives rise to bands at 11 396, 17 523, 22 000, 25 229, 30 609, 35 071, and 39 674 cm^–1^. Given the plethora of transitions of roughly equal oscillator strength that occur above 20 000 cm^–1^ found in the calculation (cf. Figure 12), a clear and unambiguous assignment of each Gaussian to a particular transition seems largely unfeasible in this spectral region. For the lower-lying transitions, the difference density suggests that the band at 11 396 cm^–1^ corresponds largely to a Mo^[O]^ d–d transition and the band at 17 523 cm^–1^ corresponds to a Mo^[O]^-to-Mo^[N]^ metal-to-metal charge transfer transition involving multiple MO 4d orbitals on both molybdenum atoms. The bands observed in the UV–vis spectrum are similar to those observed for complex **19**, where transitions at 11 891, 15 385, 17 857, and 20 121 cm^–1^ have been observed,^72^ indicating that the electronic structure and the core Mo–N–Mo bonding parameters of **13a** and **19** are similar. In the latter case, the thiolate ligands are also the less electron-donating ligands, which leads to a comparable or maybe even more pronounced asymmetry of the Mo–N–Mo unit.

### Magnetometry

**Figure 13:**
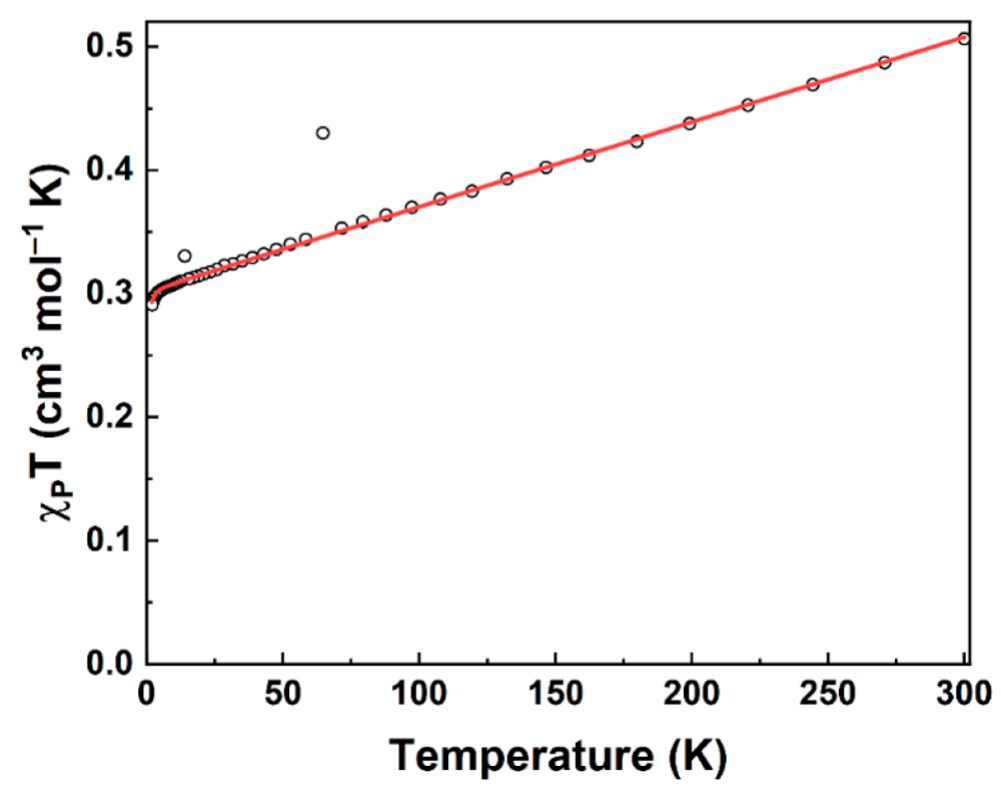
Direct current paramagnetic susceptibility data for **13a** from 2.00 to 300 K (black dots) and fit to the data with the spin Hamiltonian model (red trace). The outliers at 13.96 and 64.65 K were excluded from the model. Fit parameters: 9.7% *S* = 0 (diamagnetic) impurity (fixed on the basis of the reported NMR purity of 90.3%), TIP = 0.0006866 cm^3^ mol^–1^, *g*_avg_ = 1.89, and residual = 0.00198.

The effective magnetic moment (*μ*_eff_) of **13a** was determined via the Evans method to be 2.16(6) *μ*_B_ at 298 K.^86,87^ This is consistent with the expected spin-only value of 1.73 *μ*_B_ for an *S* = ^1^/_2_ system (with *g*_avg_ = 2). It should be noted that the value obtained via the Evans method is not corrected for temperature-independent paramagnetism (TIP), and therefore, it should not be taken at face value. In fact, it is apparent from the magnetometry data that this compound contains substantial TIP as shown by the steady linear increase in the data between ≈6 and 300 K (Figure 13). Thus, only the TIP-corrected value for the magnetic moment should be considered as informative about the average *g* value of the complex.

The *χ*_P_*T* value is 0.506(18) cm^3^ K mol^–1^ [2.01(7) *μ*_B_] at 300 K; the *χ*_P_*T* values decrease steadily and linearly with temperature over most of the temperature range until reaching 0.304(12) cm^3^ K mol^–1^ [1.56(6) *μ*_B_] at 6.16 K. The data then curve sharply downward (due to saturation), finally arriving at 0.291(11) cm^3^ K mol^–1^ [1.53(6) μ_B_] at 2.00 K. The *χ*_P_*T* value at 300 K is consistent with the value obtained via the Evans method (again, neither value is corrected for TIP).

The TIP-corrected value for *χ*_P_*T* at 300 K is 0.300(11) cm^3^ K mol^–1^ [1.55(6) *μ*_B_]. This suggests that *g*_avg_ < 2.002319 (the value for a free electron) for **13a**, which is expected for this system considering that substantial spin–orbit coupling is operative for both of the Mo centers and that their d shells are less than half-filled. In fact, the *g*_avg_ obtained from modeling the electron paramagnetic resonance (EPR) spectrum with the spin Hamiltonian is 1.96 (for details, see the Supporting Information). The model of the magnetometry data gives a *g*_avg_ of 1.89, in excellent agreement with the value obtained from EPR (*vide infra*).

### EPR Spectroscopy

**Figure 14:**
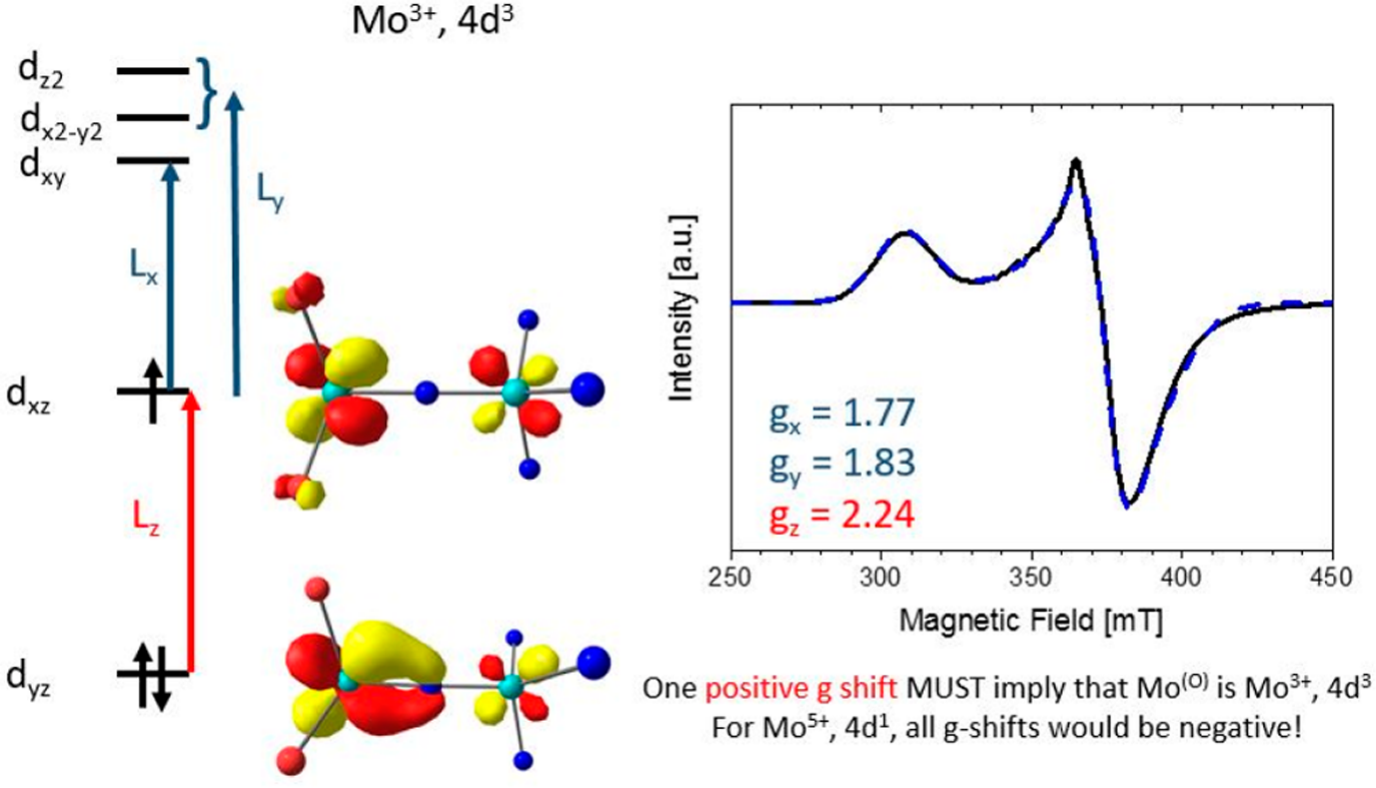
Continuous wave (cw) EPR spectrum of a 4.2 mM frozen solution of **13a** in toluene. Experimental conditions: *T* = 10 K, ν = 9.629 GHz, *P*_mw_ = 2 mW, and modulation amplitude = 0.5 mT. Also included is an Easyspin simulation (dotted line) with *g* values of 1.77, 1.83, and 2.24. Given the sensitivity of the complex, spin quantification resulted in a spin concentration of 3.3 mM. Also included is a schematic overview of the Mo^(O)^ 4d orbitals relevant for rationalizing the observed *g* shifts.

The EPR spectrum of **13a** is broad and relatively featureless (Figure 14). It is characterized by a nearly axial set of *g* values amounting to 1.77, 1.83, and 2.24. In general, the more symmetric the spin distribution over the Mo atoms, the less pronounced the shift of the *g* values. The significant shift of all *g* values away from the free electron *g* value and the absence of a resolved nitrogen hyperfine coupling indicate that the complex is indeed *S* = ^1^/_2_ and that the bulk spin density is molybdenum-centered and not centered on the μN atom. A B3LYP calculation of the *g* values with an all-electron basis set,^88,89^ relativistic corrections accounted for by the two-component X2C model,^90^ and a mean-field spin–orbit operator^91^ gave rise to values of 1.81, 1.90, and 2.19, in reasonable agreement with experiment. The EPR *g* values both in experiment and in calculation support of a dominantly Mo^[O]^-centered singly occupied natural orbital as shown in Figure 11 and Table 2 and indicate that the calculation has correctly captured the frontier 4d orbital structure of **13a**.

Because the Löwdin spin is largely located at Mo^[O]^, the measured *g* values and in particular the signs of the three *g* shifts can be rationalized by examining the 4d orbital occupations. From second-order perturbation theory, positive *g* shifts are expected for an excited state connected to the ground state by spin–orbit coupling that is obtained by promotion of an electron from a doubly occupied orbital to the SOMO. A typical example is Cu^II^, 3d^9^, where the only possible electron promotions are from doubly occupied 3d orbitals to the SOMO and all *g* shifts are positive. Negative *g* shifts are expected from perturbation theory for an excited state connected to the ground state by spin–orbit coupling, where the unpaired electron is promoted from the SOMO into an empty 4d orbital. For example, for a d^1^ system, e.g., Mo^V^, all *g* shifts are negative. The EPR spectrum of **13a** provides one positive *g* shift (+0.24) and two negative *g* shifts (−0.23 and −0.17). Most importantly, the presence of one positive shift is only compatible with a 4d^3^ system in which the positive *g* shift derives from an excited state by one-electron promotion from the doubly occupied d_*yz*_ orbital to the SOMO. As such, the presence of the positive *g* shift allows assignment with confidence of Mo^[O]^ as a formal Mo^III^, 4d^3^ ion. In turn, the positive *g* shift also implies that Mo^[N]^ is semantically best described as a formal Mo^VI^, 4d^0^.

## Conclusions

Ligation of silanols in general and tripodal silanols in particular to molybendum alkylidynes endows the resulting complexes with excellent catalytic activity for alkyne metathesis as well as a remarkable functional group tolerance; therefore, these catalysts define the standards in the field.^5−7^ Interestingly, no such synergy is gained in the tungsten series; rather, silanolates seem to upregulate the Lewis acidity of the W(VI) center to an extent that the relevant reactive intermediates are overstabilized and turnover comes to a halt.^22,53^ Equally astounding is a recent report that an isolobal tungsten nitride endowed with a tripodal silanolate ligand framework spontaneously dimerizes upon formation by a stoichiometric nitrile metathesis reaction.^14^ However, it was unclear at the outset of this study whether this behavior reflects a certain tungsten/silanolate mismatch or whether the analogous molybdenum nitrido complexes would also be dimeric entities.

To answer this question, a set of molybdenum nitrido complexes carrying monodentate or tridentate silanolate ligands were prepared by ligand exchange and fully characterized by spectroscopic and crystallographic means. All of them are regular monomeric species comprising a terminal nitride ligand. Their individual electronic character is determined, to a notable degree, by the geometric constraints of the ligand framework, whereby ^95^Mo NMR and, to a lesser degree, ^14^N NMR spectroscopy proved to be relevant probes.

Because it is known in the literature that [(*t*BuO)_3_Mo≡N] (**6a**) forms a redox couple with [(*t*BuArN)_3_Mo] (**11**) that results in smooth nitrogen atom transfer between the partners,^60^ we tested whether nitrido complexes **3** endowed with a tripodal silanolate ligand react with **11** in a similar manner. However, complete nitride transfer could not be enforced; rather, the reaction stops at nonsymmetrical μ-nitrido dimolybdenum adduct **13** as the key intermediate along the N atom transfer pathway. Although a few related complexes had previously been described in the literature, a full analysis of their electronic nature was missing. We filled this gap by a combined experimental and computational approach, which involved SC-XRD, NMR, EPR, and UV–vis spectroscopy as well as magnetometry in combination with DFT to gain a clear picture of the structure and bonding of this dinuclear mixed-valent species. Semantically, complex **13a** is best described as a [(Mo^[O]^)^+III^–(μN)^−III^–(Mo^[N]^)^+VI^], *S* = ^1^/_2_ complex with Mo^[O]^ in the low-spin configuration; it is important to note that this conclusion differs from what has been proposed in the literature for the related complex [(AdS)_3_Mo–(μN)–Mo(N*t*BuAr)_3_] (**19**), which was described as a mixed-valent Mo^+IV^/Mo^+V^ species.^60^ The formal Mo–N bond orders in **13a** are found to be 0.5 and 2.5, respectively, thus explaining why nitrogen atom transfer is incomplete, as both bond orders are larger than 0, and the calculation even provides a formal Mo–Mo bond order of 0.5. The bond orders also indicate that the tug-of-war over the nitrido ligand and its electrons is won by the more oxidized (Mo^[N]^)^+VI^. This is chemically reasonable because amides are better donors than silanolates, and hence π backbonding in (μN)–Mo^[N]^ is dominating. As such, the spin population at the Mo centers is uneven and notably larger at the more reduced Mo^[O]^ atom, whereas no direct spin density is localized at the μ-bridging N atom.

## Supporting Information Available

Experimental section (PDF)
Copies of spectra of new compounds (PDF)
Supporting computational data (PDF)
Supporting crystallographic data (PDF)

## Notes

The preparation of the ligands requires the use of *tert*-butyllithium and *m*-chloroperbenzoic acid. *tert*-Butyllithium is extremely pyrophoric. It must be handled with the greatest care under an inert atmosphere using proper Schlenk techniques. *m*-Chloroperbenzoic acid is a strong oxidant that can react violently with organic materials and must be handled with the greatest care; any excess reagent must be destroyed by reductive work up, for example, with the help of Na_2_S_2_O_3_. Further safety information is available in the Supporting Information.
