# Supplementary material for: Molybdenum(VI) Nitrido Complexes with Tripodal Silanolate Ligands. Structure and Electronic Character of an Unsymmetrical Dimolybdenum μ-Nitrido Complex Formed by Incomplete Nitrogen Atom Transfer
Source: Inorg Chem. 2024 Apr 25;63(18):8376–89. doi: 10.1021/acs.inorgchem.4c00762 (PMC11080062; doi:10.1021/acs.inorgchem.4c00762)
Supplement: Supplementary file 1 — ic4c00762_si_001.pdf [file ic4c00762_si_001.pdf]

SUPPORTING INFORMATION

Part I

Molybdenum(VI) Nitrido Complexes with Tripodal Silanolate Ligands.

Structure and Electronic Character of an Unsymmetrical Dimolybdenum μ-Nitrido Complex Formed by Incomplete Nitrogen Atom Transfer

Daniel Rütter, Maurice van Gastel, Markus Leutzsch, Nils Nöthling, Daniel SantaLucia,[+]

Frank Neese,* and Alois Fürstner*

Max-Planck-Institut für Kohlenforschung, 45470 Mülheim/Ruhr, Germany

[+] *Max-Planck-Institut für Chemische Energiekonversion, 45470 Mülheim/Ruhr, Germany*

Email: fuerstner@kofo.mpg.de; neese@kofo.mpg.de

Table of Contents

GENERAL 2
EXPERIMENTAL DATA 6
Bis(3,5-dimethylphenyl)dimethoxysilane (**S1**) 6
Ligand **7b** 7
Silane **S2** 8
Ligand **7e** 9
Complex **3a** 10
Complex **3b** 11
Complex **3c** 12
Complex **3d** 13
Complex **3e** 14
Complex **8** 15
Complex **13a** 16
Complex **13b** 20
REFERENCES 21

GENERAL

Working with Hazardous Chemicals

tert-Butyllithium is extremely pyrophoric. It must be handled with greatest care under inert atmosphere using proper Schlenk techniques.

meta-Chloroperbenzoic acid is a strong oxidant that can react violently with organic materials and must be handled with greatest care; any excess reagent must be destroyed by reductive work up with $Na_2S_2O_3$ as outlined in the procedure.

Na/K and sodium tetraethylaluminate chosen as drying agents for certain solvents as specified below are extremely pyrophoric and react violently with water. They must be handled with greatest care under inert atmosphere using proper Schlenk techniques.

All hazardous materials should be handled using the standard procedures for work with chemicals described in references such as "Prudent Practices in the Laboratory" (The National Academies Press, Washington, D.C., 2011; the full text can be accessed free of charge at https://www.nap.edu/catalog.php?record_id=12654).

Air and moisture sensitive compounds

Manipulations of air and moisture sensitive substances were carried out using standard Schlenk techniques. Unless stated otherwise, all reactions were carried out in glassware that had previously been heated in high vacuum with a Bunsen burner, cooled to ambient temperature and then filled with argon. The cannulas and syringes used to transfer reagents and solutions were carefully flushed with argon before use.

Reagents and solvents

The solvents were distilled after drying over the following reagents and stored under an argon atmosphere over molecular sieves (3 Å): tetrahydrofuran (magnesium/anthracene), benzene (CaH_2), dichloromethane (CaH_2), diethyl ether (Na/K), toluene (sodium tetraethylaluminate), *n*-pentane (Na/K). Hexamethyldisiloxane (HMDSO), [D_6]-benzene and [D_8]-toluene were degassed by freeze-pump-thaw cycles and stored over molecular sieves (3 Å). Molecular sieves were activated at 180 °C for 3 d under high vacuum (10^{-3} mbar) and stored under argon. The ligands used were dried as follows: The ligand was dissolved in toluene and then powdered molecular sieves (4 Å) were added. The resulting suspension was stirred overnight, filtered *via* cannula and then all volatile components were removed *in vacuo* (10^{-3} mbar).

Known compounds were prepared according to the literature: N≡Mo(O*t*Bu)$_3$ (**6a**),[1] N≡Mo(OEt)$_3$ (**6b**),[1] N≡Mo(N(*t*Bu)(Ar))$_3$ (**10**),[2-3] N≡Mo(NMe$_2$)$_3$ (**9**),[4] Mo(N(*t*Bu)(Ar))$_3$ (**11**),[2,5-7] ligands **7a**, **7c**, **7d**.[8-10]

Chromatography

Flash chromatographic separations were performed on silica gel (Merck, type 9385, 230-400 mesh, 60 Å pore diameter) as stationary phase. Reaction control was performed on ready-to-use films for thin-layer chromatography (40 x 80 mm, Polygram® SIL G/UV254). Detection was achieved under UV-light (254 nm) and by staining with either acidic *p*-anisaldehyde, cerium ammonium molybdate, or basic $KMnO_4$ solution.

Commercially available starting materials

Commercially available chemicals were used without purification unless otherwise noted.

Elemental analysis

Elemental analyses: Mikroanalytisches Laboratorium H. Kolbe, Mülheim/Ruhr, Germany.

Mass spectrometry (MS)

High-resolution mass spectra (HRMS) were measured with a Finnigan MAT 95 spectrometer (EI) or a Bruker APEX III FT-ICR-MS (ESI). All values are given in mass units per elementary charge (*m/z*).

Nuclear magnetic resonance spectroscopy (NMR)

Spectra were recorded on Bruker AvanceIIIHD 400 MHz or Avance Neo 600 MHz NMR spectrometers in the indicated solvents; chemical shifts (δ) are given in ppm relative to tetramethylsilane (TMS), coupling constants (*J*) in Hz. Solvent signals were used as reference and the chemical shifts were converted to the TMS scale ([D$_6$]-benzene: δ_C = 128.06 ppm, residual ^{1}H: δ_H = 7.16 ppm; CD_2Cl_2: δ_C = 53.84 ppm, residual ^{1}H: δ_H = 5.32 ppm; [D$_8$]-toluene: δ_C = 20.43 ppm, residual ^{1}H: δ_H = 2.09 ppm). 1D ^{29}Si NMR spectra were acquired with a polarisation transfer pulse sequence (refocussed INEPT) and broadband proton decoupling. ^{14}N and ^{95}Mo NMR spectra were acquired using the Bruker aring pulse sequence to minimize acoustic ringing from the NMR probe. ^{95}Mo NMR spectra were acquired at 298 K unless noted otherwise. The π/2 ^{95}Mo pulse was calibrated with a Na_2MoO_4 (2 M in D_2O) sample and had a typical length of 22.5 μs. ^{14}N NMR spectra were generally acquired at 333 K. The π/2 ^{14}N Pulse was calibrated using saturated solution of Et_4NBr in $CDCl_3$ and had a typical length of 21.0 μs. ^{15}N NMR shifts were extracted from cross peaks in a ^{1}H-^{15}N-HMBC experiments. ^{14}N, ^{29}Si and ^{95}Mo chemical shifts were referenced indirectly to the ^{1}H chemical shift of the solvent according to IUPAC

recommendations using the *xiref* macro in Bruker Topspin.[11] ^{14}N chemical shifts are reported relative to CH_3NO_2 (δ = 0 ppm; Ξ = 7.226317%), ^{15}N chemical shifts are reported relative to CH_3NO_2 (δ = 0 ppm; Ξ = 10.136767%), ^{29}Si chemical shifts are reported relative to Me_4Si (δ = 0 ppm; Ξ = 19.867187%) and ^{95}Mo chemical shifts relative to Na_2MoO_4 (δ = 0 ppm; Ξ = 6.516926%). The multiplicities of the signals are described by the following abbreviations: s: singlet, d: doublet, t: triplet, q: quartet, pent: quintet, sept: septet, m: multiplet, bs: broad signal.

Infrared spectroscopy (IR)

IR spectra were measured on a Spectrum One (Perkin-Elmer) spectrometer at room temperature. IR spectra of air and moisture sensitive compounds were measured on a Nicolet iS5 Spectrometer (Thermo Scientific) at room temperature inside a Glovebox (Argon). Absorption bands are reported in wavenumbers (cm^{-1}).

UV-vis spectroscopy

The UV-vis spectra of **13a** were recorded in a 2 mm quartz cuvette at room temperature on a Cary6000i UV-vis-NiR spectrometer. The highly sensitive complex **13a** was mulled with paraffin oil in order to protect the sample from oxygen and water. The measured spectrum was subsequently deconvoluted with 7 Gaussian bands.

EPR spectroscopy

EPR spectra were recorded at T = 10K on an X-Band Bruker Elexsys continuous-wave (cw) EPR spectrometer. The microwave frequency was 9.629 GHz, the microwave power was 2 mW. The modulation amplitude was 0.5 mT. Spin quantification was achieved by double integration of the EPR signal and comparison to a frozen Cu(II)-based solution of known concentration.

Magnetometry

Variable temperature direct current (DC) magnetic susceptibility data for **13a** was measured using a MPMS3 Quantum Design SQUID magnetometer at the Max Planck Institute for Chemical Energy Conversion (MPI CEC) in the Joint Workspace of the Max-Planck-Institut für Kohlenforschung (MPI KoFo).

The magnetic moment of the sample was measured in increments (with logarithmic spacing) from 300 to 2.00 K in the presence of an applied external magnetic field of 1 T. The data exhibit two outliers, one at 13.96 K and one at 64.65 K; these outliers were excluded from modelling the data. The data were corrected for inherent diamagnetism using the formula: $\chi_D = -(M_W/2) \cdot 10^{-6}$ cm^3 mol^{-1}.[12] The susceptibility data were modeled using the in-house software JulX20.v1.4.SL, written by Eckhard Bill

and available upon request via email to: daniel.santalucia@cec.mpg.de.[13] The data were modelled using the Zeeman spin Hamiltonian (Eq. 1):

$$\hat{H} = \mu_B \vec{S}\, \overleftrightarrow{\boldsymbol{g}}\, \vec{H} \quad (1)$$

where μ_B is the Bohr magneton, $\vec{S}$ is the (effective) electron spin vector, $\overleftrightarrow{\boldsymbol{g}}$ is the electron g-tensor, and $\vec{H}$ is the applied external magnetic field. The data were modeled with an average g-value.[14] Temperature independent paramagnetism (TIP) was also included in the model of the data to account for the steady linear increase of the data over most of the temperature range (from ~6 to 300 K). Based on the purity of the compound as determined by NMR spectroscopy (90.3%), a 9.7% diamagnetic impurity was also included as a fixed parameter in the model to the data.

EXPERIMENTAL DATA

Bis(3,5-dimethylphenyl)dimethoxysilane (**S1**)

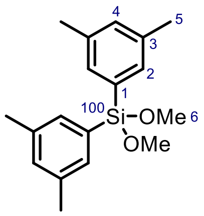

A solution of 5-bromo-*m*-xylene (15 mL, 0.11 mol) in THF (75 mL) was added dropwise over a period of 1 h to a suspension of Mg turnings (2.9 g, 0.12 mol) and LiCl (4.7 g, 0.11 mol) in THF (40 mL). After 5 min, heat evolution was observed. Stirring was continued for 30 min before the mixture was transferred *via* cannula into the dropping funnel used in the reaction described below.

This solution of (3,5-dimethylphenyl)magnesium bromide in THF was added dropwise over a period of 10 min to a solution of $Si(OMe)_4$ (7.5 mL, 51 mmol) in THF (135 mL) at 0 °C. Once the addition was complete, stirring was continued for 2 h at ambient temperature before water (150 mL) was carefully added to quench the reaction. The mixture was transferred into a separatory funnel and the aqueous phase extracted with EtOAc (3 x 100 mL). The combined organic layers were dried over $MgSO_4$, and all volatile components were removed *in vacuo* to give a colorless oil. The crude product was purified by distillation in high vacuum (10^{-3} mbar, bath temperature: 185 °C; b. p. 145-147 °C) to give the title compound as a colorless solid (7.6 g, 50%).

^{1}H NMR (600 MHz, [D_2]-dichloromethane, 298 K): δ = 7.24−7.23 (m, 4H; H-2), 7.08−7.07 (m, 2H; H-4), 3.59 (s, 6H; H-6), 2.30 ppm (q, *J* = 0.6 Hz, 12H; H-5).

^{13}C NMR (151 MHz, [D_2]-dichloromethane, 298 K): δ = 137.7 (C-3), 132.69 (C-1), 132.68 (C-2), 132.4 (C-4), 51.0 (C-6), 21.5 ppm (C-5).

^{29}Si NMR (119 MHz, [D_2]-dichloromethane, 298 K): δ = 28.9 ppm.

IR (ATR): $\tilde{\nu}$ = 2934, 2857, 2834, 1594, 1455, 1404, 1379, 1273, 1182, 1139, 1072, 991, 938, 870, 851, 799, 720, 696, 569, 547, 532, 485, 447, 431, 417 cm^{-1}.

HRMS (EI^{+}): *m/z* calculated for $C_{18}H_{24}O_2Si$ [M]$^{+}$: 300.15401, found: 300.15412.

Ligand **7b**

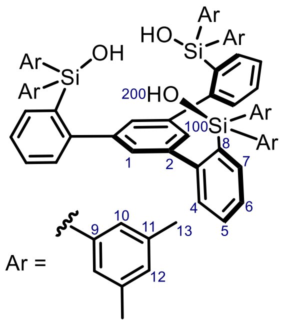

A solution of *tert*-butyllithium (1.7 M in *n*-pentane, 28 mL, 48 mmol) was added dropwise over a period of 5 min to a suspension of 1,3,5-tris-2'-bromophenylbenzene (4.3 g, 4.4 mmol)[9] in Et_2O (48 mL) at −110 °C. Stirring was continued for 1 h at ambient temperature, after which the reaction mixture was cooled again to −110 °C and a solution of compound **S1** (7.3 g, 24 mmol) in Et_2O (20 mL) was slowly added. Stirring was continued for 17 h at ambient temperature before the reaction was carefully quenched with water (50 mL). The aqueous phase was extracted with CH_2Cl_2 (3 x 100 mL) and the combined organic layers were dried over $MgSO_4$. All volatile components were removed *in vacuo* to give as a light yellow foam (9.2 g), which was used in the next step without further purification.

Concentrated aq. HCl (60 mL) was added to a solution of this crude material (9.2 g) in THF (200 mL) at 0 °C in air. Stirring was continued for 2 h at ambient temperature before aq. NaOH (3 M, 250 mL) was carefully added. The aqueous phase was extracted with CH_2Cl_2 (3 x 100 mL) and the combined organic layers were dried over $MgSO_4$. All volatile components were removed *in vacuo* to give a light yellow crystalline solid, which was washed with CH_2Cl_2 (3 x 5 mL) and dried under high vacuum (10^{-3} mbar) to give the title compound as a white powder (7.5 g, 88% over two steps).

^{1}H NMR (600 MHz, [D_2]-dichloromethane, 298 K): δ = 7.41−7.39 (m, 3H; H-7), 7.25−7.20 (m, 6H; H-5 and H-6), 7.13−7.12 (m, 12H; H-10), 7.07 (s, 3H; H-1), 6.91−6.90 (m, 6H; H-12), 6.84−6.83 (m, 3H; H-4), 3.96 (s, 3H; H-200), 2.12 ppm (s, 36H; H-13).

^{13}C NMR (151 MHz, [D_2]-dichloromethane, 298 K): δ = 148.9 (C-3), 144.1 (C-2), 137.8 (C-7), 137.5 (C-11), 134.5 (C-8), 132.2 (C-10), 131.6 (C-12), 130.0 (C-5), 129.2 (C-4), 129.0 (C-1), 126.3 (C-6), 21.4 ppm (C-13).

^{29}Si NMR (119 MHz, [D_2]-dichloromethane, 298 K): δ = 12.4 ppm.

IR (ATR): $\tilde{\nu}$ = 3415, 3013, 2916, 2857, 1584, 1557, 1469, 1437, 1402, 1376, 1269, 1168, 1138, 1088, 1064, 1037, 989, 939, 896, 860, 845, 800, 761, 739, 714, 696, 666, 652, 637, 622, 553, 537, 520, 491, 475, 411x cm^{-1}.

HRMS (ESI$^+$): *m/z* calculated for $C_{72}H_{72}NaO_3Si_3$ $[M+Na]^+$: 1091.46815, found: 1091.46797.

Silane **S2**

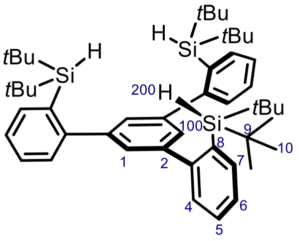

A solution of *tert*-butyllithium (1.7 M in *n*-pentane, 10 mL, 17 mmol) was added dropwise over a period of 5 min to a solution of 1,3,5-tris-2'-bromophenylbenzene (1.5 g, 2.8 mmol)[9] in THF (30 mL) at −78 °C. The mixture was allowed to reach ambient temperature while the color changed to red. The reaction mixture was quickly cooled to −78 °C again and $t\mathrm{Bu_2SiHCl}$ (6.5 mL, 32 mmol) was added. Stirring was continued for 20 h at ambient temperature before isopropanol (5 mL) was carefully added to quench the reaction. All volatile components were removed *in vacuo*. The residue was dispersed in *n*-hexane and the suspension filtered through a plug of silica. The filtrate was evaporated and the residue purified by flash chromatography on silica gel (*n*-hexane) to give the title compound as a colorless solid (0.78 g, 39%). Colorless single crystals suitable for X-ray diffraction were grown by slow evaporation of a solution of the compound in *n*-pentane.

^{1}H NMR (600 MHz, [D$_2$]-dichloromethane, 298 K): δ = 7.69 (d, *J* = 7.4 Hz, 3H; H-7), 7.42−7.39 (m, 6H; H-4 and H-5), 7.35−7.31 (m, 3H; H-6), 7.15 (s, 3H; H-1), 3.86 (s, 3H; H-200), 0.98 ppm (s, 54H; H-10).

^{13}C NMR (151 MHz, [D$_2$]-dichloromethane, 298 K): δ = 151.0 (C-3), 142.4 (C-2), 135.3 (C-7), 134.3 (C-8), 130.6 (C-1 and C-4), 128.8 (C-5), 125.8 (C-6), 29.6 (C-10), 19.7 ppm (C-9).

^{29}Si NMR (119 MHz, [D$_2$]-dichloromethane, 298 K): δ = 0.9 ppm.

IR (ATR): $\tilde{\nu}$ = 2926, 2889, 2853, 2149, 1584, 1557, 1468, 1437, 1409, 1386, 1363, 1263, 1121, 1098, 1086, 1063, 1048, 1012, 935, 896, 872, 806, 764, 737, 723, 682, 631, 615, 575, 526, 466, 450 cm^{-1}.

HRMS (ESI$^+$): *m/z* calculated for $C_{48}H_{72}NaSi_3$ [M+Na]$^+$: 755.48340, found: 755.48302.

Ligand **7e**

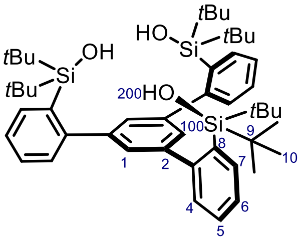

*m*CPBA (77% *w/w*, 1.23 g, 5.50 mmol) was added in portions to a solution of silane **S2** (671 mg, 0.915 mmol) in THF (9 mL) at 0 °C in air. The mixture was stirred at ambient temperature for 3 d before sat. aq. $Na_2S_2O_3$ (10 mL) was added. The mixture was transferred into a separatory funnel and diluted with *tert*-butyl methyl ether (100 mL), and the aqueous phase was separated. The organic phase was washed with sat. aq. Na_2CO_3 solution (3 x 50 mL) and dried over $MgSO_4$ before all volatile components were removed *in vacuo* to give a colorless oil. The residue was dissolved in THF (40 mL) and aq. NaOH solution (3 M, 40 mL) was added. The resulting mixture was vigorously stirred for 30 min, transferred into a separatory funnel and diluted with *tert*-butyl methyl ether (50 mL). The aqueous phase was separated and the organic phase was washed with brine (50 mL). The organic layer was dried over $MgSO_4$ before all volatile components were removed *in vacuo* to give a colorless oil.

The oil was dissolved in *n*-pentane (15 mL) and the solution stored over 3 Å MS for 16 h. All volatile components were removed *in vacuo* (10^{-3} mbar) to give a colorless oil. The oil was dissolved in toluene (1 mL), which was then evaporated to give a white foam. The foam was dried at 120 °C for 30 min under high vacuum (10^{-3} mbar) whereupon it started to melt. While cooling to ambient temperature, ligand **7e** started to crystallize as a white solid (617 mg, 86%). Colorless single crystals of the monohydrate of complex **7e** suitable for X-ray diffraction were obtained by storing a solution of the compound in hexamethyldisiloxane (HMDSO) at −20 °C.

^{1}H NMR (600 MHz, [D$_8$]-toluene, 353 K): δ = 7.64 (dd, *J* = 7.4, 1.5 Hz, 3H; H-7), 7.45 (s, 3H; H-1), 7.32 (bs, 3H; H-4), 7.17 (td, *J* = 7.5, 1.5 Hz, 3H; H-5), 7.13 (td, *J* = 7.4, 1.4 Hz, 3H; H-6), 1.68 (bs, 3H; H-200), 1.06 ppm (s, 54H; H-10).

^{13}C NMR (151 MHz, [D$_8$]-toluene, 353 K): δ = 149.3 (C-3), 144.7 (C-2), 135.1 (C-7), 135.0 (C-8), 131.0 (C-4), 128.9 (C-5), 126.2 (C-6), 28.9 (C-10), 21.4 ppm (C-9). The signal of C-1 is broad and overlaps with a [D$_8$]-toluene peak at 129.0 ppm.

^{29}Si NMR (119 MHz, [D$_8$]-toluene, 353 K): δ = 3.7 ppm.

IR (ATR): $\tilde{\nu}$ = 3644, 3607, 3051, 2932, 2889, 2856, 1583, 1558, 1471, 1429, 1409, 1388, 1363, 1259, 1121, 1096, 1063, 1012, 935, 885, 820, 760, 738, 727, 714, 683, 629, 577, 529, 479, 437, 413 cm^{-1}.

HRMS (ESI$^+$): *m/z* calculated for $C_{48}H_{72}NaO_3Si_3$ [M+Na]$^+$: 803.46815, found: 803.46838.

Complex **3a**

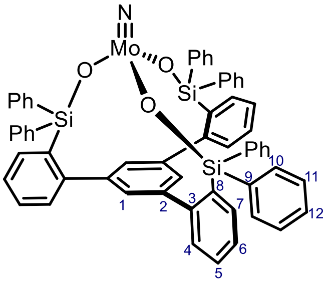

A solution of complex **6a** (75.0 mg, 0.228 mmol) in toluene (1 mL) was added *via* cannula to a solution of ligand **7a** (205 mg, 0.228 mmol) in toluene (20 mL). The resulting colorless solution was stirred for 4 h before all volatile components were removed *in vacuo* (10^{-3} mbar) at ambient temperature to give a light yellow sticky solid. The residue was washed with *n*-pentane (5 mL) and the filter cake dried *in vacuo* (10^{-3} mbar) to give the title complex as a white solid material (226 mg, 99%).

^{1}H NMR (600 MHz, [D$_6$]-benzene, 333 K): δ = 7.79 (d, *J* = 6.7 Hz, 12H; H-10), 7.70 – 7.65 (m, 3H; H-7), 7.27 (s, 3H, 1), 7.11 – 7.02 (m, 24H; H-5, H-6, H-11 & H-12), 6.95 – 6.89 ppm (m, 3H; H-4).

^{13}C NMR (151 MHz, [D$_6$]-benzene, 333 K): δ = 149.4 (C-3), 144.4 (bs, C-2), 137.5 (C-7), 137.5 (bs, C-9), 135.1 (C-10), 134.6 (bs, C-8), 130.4 (bs, C-4), 130.2 (C-5), 130.1 (C-12), 129.2 (bs, C-1), 126.4 ppm (C-6).

^{95}Mo NMR (26 MHz, [D$_6$]-benzene, 298 K): δ = 117 ppm.

IR (ATR): $\tilde{\nu}$ = 3049, 1585, 1470, 1428, 1407, 1115, 1088, 1064, 1029, 1019, 997, 926, 916, 829, 765, 740, 711, 693, 637, 622, 563, 537, 510, 496, 451, 436, 408 cm^{-1}.

HRMS (EI$^+$): *m/z* calculated for $C_{60}H_{45}MoNO_3Si_3$ [M]$^+$ 1009.17569, found: 1009.17660.

Elemental analysis (%) calculated for $C_{60}H_{45}MoNO_3Si_3$: C 71.48, H 4.50, Mo 9.52, N 1.39, Si 8.36; found: C 71.13, H 4.54, Mo 9.49, N 1.37, Si 8.31.

Complex **3b**

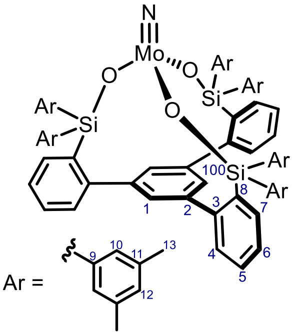

A solution of complex **6a** (112 mg, 0.340 mmol) in toluene (6 mL) was added *via* cannula to a solution of ligand **7b** (361 mg, 0.338 mmol) in toluene (33 mL). The resulting colorless mixture was stirred for 2 h at ambient temperature before all volatile components were removed *in vacuo* (10^{-3} mbar) to give a white solid. The residue was extracted with toluene (2 x 2 mL) and insoluble material filtered off *via* cannula. The filtrate was evaporated *in vacuo* (10^{-3} mbar) at ambient temperature to give a white solid, which was suspended in *n*-pentane (5 mL). All volatile components were removed *in vacuo* (10^{-3} mbar) to give a white solid. This trituration with *n*-pentane was performed three times in total. The residue was dried in high vacuum (10^{-3} mbar) at 60 °C for 1 h to give the title complex as a white solid material (354 mg, 89%).

^{1}H NMR (600 MHz, [D$_6$]-benzene, 298 K): δ = 7.86 (d, *J* = 7.2 Hz, 3H; H-7), 7.67 (s, 12H; H-10), 7.61 (s, 3H; H-1), 7.05 (td, *J* = 7.4, 1.5 Hz, 3H; H-6), 7.02 (td, *J* = 7.4, 1.7 Hz, 3H; H-5), 6.96 (d, *J* = 7.5 Hz, 3H; H-4), 6.75 (s, 6H; H-12), 1.98 ppm (s, 36H; H-13).

^{13}C NMR (151 MHz, [D$_6$]-benzene, 298 K): δ = 149.2 (C-3), 144.2 (C-2), 137.73 (C-11), 137.70 (C-7), 137.5 (C-9), 135.2 (C-8), 132.6 (C-10), 132.1 (C-12), 130.1 (C-5), 129.9 (C-4), 129.6 (C-1), 126.3 (C-6), 21.4 ppm (C-13).

^{29}Si NMR (119 MHz, [D$_6$]-benzene, 298 K): δ = 7.4 ppm.

^{95}Mo NMR (26 MHz, [D$_6$]-benzene, 298 K): δ = 114 ppm.

^{95}Mo NMR (26 MHz, [D$_8$]-toluene, 298 K): δ = 116 ppm.

IR (ATR): $\tilde{\nu}$ = 3010, 2916, 2856, 1584, 1557, 1469, 1437, 1405, 1377, 1268, 1136, 1089, 1022, 995, 928, 862, 842, 764, 740, 717, 695, 665, 651, 638, 623, 578, 558, 549, 528, 520, 508, 475, 463, 443, 422, 408 cm^{-1}.

HRMS (ESI$^+$): *m/z* calculated for $C_{72}H_{70}MoNO_3Si_3$ [M+H]$^+$: 1178.37121, found: 1178.37152.

Elemental analysis (%) calculated for $C_{72}H_{69}MoNO_3Si_3$: C 73.50, H 5.91, Mo 8.16, N 1.19, Si 7.16; found: C 72.44, H 5.87, Mo 8.07, N 1.17, Si 7.09.

Complex **3c**

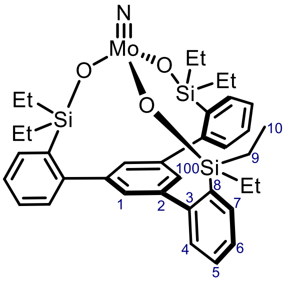

A solution of complex **6a** (250 mg, 0.758 mmol) in toluene (8 mL) was added *via* cannula to a solution of ligand **7c** (465 mg, 0.759 mmol) in toluene (78 mL). The resulting solution was stirred for 10 min before all volatile components were removed *in vacuo* (10^{-3} mbar) at ambient temperature over a period of 1 h to leave a light yellow foam. This residue was dissolved in toluene (2 mL) and *n*-pentane (40 mL), the resulting mixture was filtered *via* cannula and the filtrate stored at −78 °C overnight to form a white precipitate. The supernatant solution was syphoned off *via* cannula at −78 °C and the filter cake was dried under high vacuum (10^{-3} mbar) to give a first crop of the title complex as a white solid (325 mg, 60%). The filtrate was evaporated *in vacuo* (10^{-3} mbar) and the residue dissolved in toluene (0.4 mL) and *n*-pentane (8 mL). The resulting mixture was filtered *via* cannula and the filtrate stored at −78 °C overnight to yield a white precipitate. The supernatant solution was removed *via* cannula at −78 °C and the filter cake dried under high vacuum (10^{-3} mbar) to give a second crop of the complex as a white solid (127 mg, 23%). Overall yield: 452 mg, 83 %

^{1}H NMR (600 MHz, [D$_8$]-toluene, 298 K): δ = 7.36 (bs, 3H; H-1), 7.34−7.33 (m, 3H; H-7), 7.21−7.20 (m, 3H; H-4), 7.17−7.12 (m, 6H; H-5 and H-6), 1.06 (t, *J* = 7.8 Hz, 18H; H-10), 0.98−0.84 ppm (m, 12H; H-9).

^{13}C NMR (151 MHz, [D$_8$]-toluene, 298 K): δ = 149.3 (C-3), 144.6 (C-2), 135.9 (C-8), 134.6 (C-7), 130.7 (C-4), 129.3 (C-5), 128.2 (C-1), 126.8 (C-6), 9.4 (C-9), 7.2 ppm (C-10).

^{29}Si NMR (119 MHz, [D$_8$]-toluene): δ = 13.7 ppm.

^{95}Mo NMR (26 MHz, [D$_8$]-toluene, 298 K): δ = 120 ppm.

^{14}N NMR (29 MHz, [D$_8$]-toluene, 333 K): δ = 468 ppm; $\Delta\nu_{½}$ = 1425 Hz.

IR (ATR): $\tilde{\nu}$ = 3051, 2954, 2910, 2874, 1583, 1557, 1460, 1409, 1378, 1260, 1233, 1124, 1098, 1061, 1043, 1004, 861, 759, 711, 624, 507, 462 cm^{-1}.

HRMS (ESI^{+}): *m/z* calculated for $C_{36}H_{46}MoNO_3Si_3$ [M+H]$^{+}$: 722.18341, found: 722.18272.

Elemental analysis (%) calculated for $C_{36}H_{45}MoNO_3Si_3$: C 60.06, H 6.30, Mo 13.33, N 1.95, Si 11.70; found: C 59.88, H 6.22, Mo 13.09, N 1.92, Si 11.51.

Complex **3d**

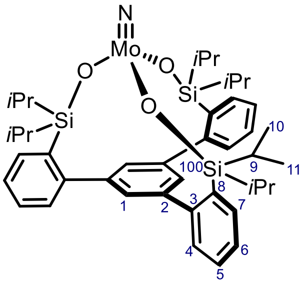

A solution of complex **6a** (173 mg, 0.526 mmol) in toluene (8 mL) was added *via* cannula to a solution of ligand **7d** (367 mg, 0.527 mmol) in toluene (52 mL). The resulting colorless solution was stirred for 5 min before all volatile components were removed *in vacuo* (10^{-3} mbar) at ambient temperature over a period of 2 h to give a beige solid residue. *n*-Pentane (40 mL) was added before all volatile components were removed *in vacuo* (10^{-3} mbar) to give the title complex as a white solid material (419 mg, 99%). Colorless single crystals suitable for X-ray diffraction were grown by slow cooling of a saturated solution of the complex in Et_2O from 20 °C to 5 °C.

^{1}H NMR (600 MHz, [D$_8$]-toluene, 298 K): δ = 7.38 (s, 3H; H-1), 7.36−7.33(m, 3H; H-7), 7.21−7.20 (m, 3H; H-4), 7.15−7.12 (m, 6H; H-5 and H-6), 1.33−1.26 (m, 6H; H-9), 1.23 (d, *J* = 7.0 Hz, 18H; H-11), 1.08 ppm (d, *J* = 7.3 Hz, 18H; H-10).

^{13}C NMR (151 MHz, [D$_8$]-toluene, 298 K): δ = 149.7 (C-3), 144.1 (C-2), 134.6 (C-8), 134.4 (C-7), 131.1 (C-4), 129.0 (C-5) [signal overlaps with a solvent peak (confirmed by DEPT-135)], 128.0 (C-1), 126.5 (C-6), 18.3 (C-10), 17.9 (C-11), 15.6 ppm (C-9).

^{29}Si NMR (119 MHz, [D$_8$]-toluene, 298 K): δ = 13.3 ppm.

^{95}Mo NMR (26 MHz, [D$_8$]-toluene, 298 K): δ = 121 ppm.

^{14}N NMR (29 MHz, [D$_8$]-toluene, 333 K): δ = 472 ppm; $\Delta\nu_{½}$ = 1500 Hz.

IR (ATR): $\tilde{\nu}$ = 3052, 2956, 2939, 2889, 2861, 1585, 1461, 1433, 1411, 1382, 1242, 1126, 1093, 1065, 1015, 1004, 908, 876, 773, 760, 738, 724, 698, 667, 653, 628, 531, 471, 417 cm^{-1}.

HRMS (ESI$^+$): *m/z* calculated for $C_{42}H_{58}MoNO_3Si_3$ [M+H]$^+$: 806.27731, found: 806.27669.

Elemental analysis (%) calculated for $C_{42}H_{57}MoNO_3Si_3$: C 62.73, H 7.15, Mo 11.93, N 1.74, Si 10.48; found: C 62.42, H 7.11, Mo 11.80, N 1.73, Si 10.27.

Complex **3e**

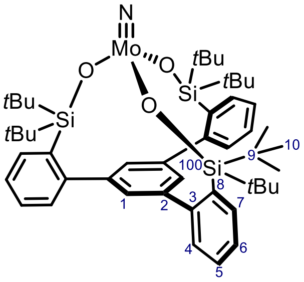

Ligand **7e** is extremely hygroscopic. Therefore, a mixture of ligand **7e** (117 mg, 0.150 mmol) and 4 Å MS in *o*-xylene (5 mL) was vigorously stirred for 7 h at ambient temperature before it was filtered *via* cannula into a suspension of complex **6b** (36.7 mg, 0.150 mmol) in *o*-xylene (5 mL). The cannula was washed with *o*-xylene (4 mL). The resulting white suspension was stirred at 140 °C (bath temperature), leading to the formation of a colorless solution. After 14 h at 140 °C, the mixture was allowed to reach ambient temperature before it was filtered *via* cannula into a flame-dried 25-mL Schlenk flask. All volatile components were removed *in vacuo* (10^{-3} mbar) to give a colorless foam. Hexamethyldisiloxane (10 mL) was added which resulted in the formation of a white suspension. The suspension was vigorously stirred and then concentrated *in vacuo* (10^{-3} mbar) to ca. 1/4 of its original volume. The supernatant was filtered off *via* cannula. The residue was dissolved in *n*-pentane (2 mL) before all volatile components were removed *in vacuo* (10^{-3} mbar) to give the title complex as a white solid (99.2 mg, 75%). Yellow single crystals suitable for X-ray diffraction were grown by slow cooling of a saturated solution of the complex in Et_2O from 20 °C to −20 °C.

^{1}H NMR (600 MHz, [D_8]-toluene, 298 K): δ = 7.60−7.58 (m, 3H; H-7), 7.19 (s, 3H; H-1), 7.16−7.10 (m, 9H; H-4, H-5 and H-6), 1.24 ppm (s, 54H; H-10).

^{13}C NMR (151 MHz, [D_8]-toluene, 298 K): δ = 149.8 (C-3), 144.7 (C-2), 134.6 (C-8), 134.1 (C-7), 131.8 (C-4), 128.6 (C-5), 126.9 (C-1), 125.9 (C-6), 29.5 (C-10), 22.4 ppm (C-9).

^{29}Si NMR (119 MHz, [D_8]-toluene, 298 K): δ = 10.2 ppm.

^{95}Mo NMR (26 MHz, [D_8]-toluene, 298 K): δ = 116 ppm.

^{14}N NMR (29 MHz, [D_8]-toluene, 333 K): δ = 476 ppm; $\Delta\nu_{1/2}$ = 1724 Hz.

IR (ATR): $\tilde{\nu}$ = 3045, 2966, 2947, 2856, 1582, 1558, 1473, 1428, 1408, 1395, 1364, 1256, 1121, 1087, 1064, 1045, 994, 891, 820, 759, 738, 725, 716, 635, 594, 525, 482, 420 cm^{-1}.

HRMS (ESI$^+$): *m/z* calculated for $C_{48}H_{70}MoNO_3Si_3$ [M+H]$^+$: 890.37121, found: 890.37107.

Elemental analysis (%) calculated for $C_{48}H_{69}MoNO_3Si_3$: C 64.90, H 7.83, Mo 10.80, N 1.58, Si 9.49; found: C 64.81, H 7.85, Mo 10.68, N 1.57, Si 9.51.

Complex **8**

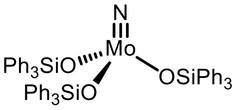

A solution of complex **6a** (116 mg, 0.351 mmol) in toluene (5 mL) was added *via* cannula to a solution of Ph_3SiOH (290 mg, 1.05 mmol) in toluene (35 mL). The resulting colorless mixture was stirred for 10 min before all volatile components were removed *in vacuo* (10^{-3} mbar) at ambient temperature over a period of 1 h to give a white solid material (*note*: the slow evaporation was necessary to ensure full conversion in this case). The crude product was dissolved in toluene (10 mL) which was then evaporated (10^{-3} mbar) at ambient temperature to give the title complex as a white solid (322 mg, 98%). The analytical and spectroscopic data is in agreement with the literature.[15]

Light yellow single crystals suitable for X-ray diffraction were grown by slow vapor diffusion of *n*-pentane into a solution of the complex in benzene.

Complex **13a**

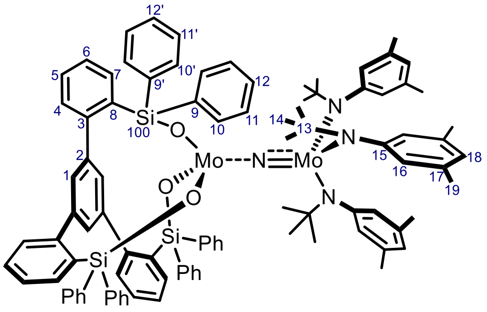

A dark red solution of complex **11** (132 mg, 0.211 mmol) in toluene (1.3 mL) was added *via* syringe to a light yellow suspension of complex **3a** (189 mg, 0.188 mmol) in toluene (19 mL), causing an immediate color change to dark purple. The syringe was washed with toluene (0.2 mL). After stirring for 5 min, all volatile components were removed *in vacuo* (10^{-3} mbar) to give a black solid. This residue was washed with *n*-pentane (3 x 3 mL) and the filter cake dried under high vacuum (10^{-3} mbar) at 60 °C to give the title complex as a black solid material (242 mg, 79%). Orange single crystals suitable for X-ray diffraction were grown by slow vapor diffusion of *n*-pentane into a solution of the complex in [D_6]-benzene.

Due to the high sensitivity of the complex, ^{1}H and ^{13}C NMR spectra showed small amounts of free aniline HN(*t*Bu)(Ar) (Ar = 3,5-dimethylphenyl) and the terminal nitrido complex [N≡Mo(N(*t*Bu)(Ar))$_3$] (**10**).

^{1}H NMR (600 MHz, [D_6]-benzene, 298 K): δ = 10.02 (s, 3H; H-1), 7.94 (d, *J* = 7.7 Hz, 3H; H-4), 7.78 (t, *J* = 7.3 Hz, 3H; H-6), 7.70 (d, *J* = 6.8 Hz, 3H; H-7), 7.54 (s, 3H; H-18), 7.36 (t, *J* = 7.4 Hz, 3H; H-5), 7.21 (t, *J* = 7.3 Hz, 6H; H-11), 6.89 (d, *J* = 6.3 Hz, 6H; H-10), 6.86 (t, *J* = 7.4 Hz, 3H; H-12'), 6.77 (bs, 6H; H-11'), 6.56 (t, *J* = 7.5 Hz, 3H; H-12), 4.78 (s, 27H; H-14), 2.68 ppm (s, 18H; H-19) (Note: Signals for H-10' and H16 were not observed at 298 K due to the hindered rotation of the Ar groups. However, it was possible to assign them at 333 K and 343 K, see the VT NMR data).

^{13}C NMR (151 MHz, [D_6]-benzene, 298 K): δ = 484.4 (bs, C-13), 269.0 (C-15), 257.3 (C-9), 240.8 (C-8), 178.4 (C-9‘), 161.4 (C-16), 150.4 (C-2), 148.6 (C-7), 148.1 (C-3), 146.8 (bs; C-17), 145.0 (bs; C-17), 140.1 (C-10), 136.4 (bs; C-10‘), 134.7 (C-1), 132.8 (C-4), 131.4 (C-12), 131.3 (C-6), 130.5 (C-5), 129.5 (C-12‘), 129.2 (bs; C-11 and C11‘), 127.4 (C-18), 95.7 (bs; C-14), 23.3 ppm (bs; C-19).

^{29}Si NMR (119 MHz, [D_6]-benzene, 298 K): δ = 93.2 ppm.

Magnetic Susceptibility (Evans method, 600 MHz, [D_6]-benzene, 298 K): μ_{eff} = 2.16(6) μ_B (see below).

IR (ATR): $\tilde{\nu}$ = 2968, 1585, 1428, 1356, 1177, 1121, 1112, 1086, 1000, 984, 960, 940, 920, 898, 848, 771, 761, 730, 708, 699, 688, 548, 532 cm^{-1}.

HRMS (ESI$^+$): *m/z* calculated for $C_{96}H_{99}Mo_2N_4O_3Si_3$ [M]$^+$: 1635.51277, found: 1635.51489.

Elemental analysis (%) calculated for $C_{96}H_{99}Mo_2N_4O_3Si_3$: C 70.61, H 6.11, Mo 11.75, N 3.43, Si 5.16; found: C 69.89, H 6.05, Mo 11.63, N 3.39, Si 5.11.

Quantitative NMR and Evans Method. **Sample Preparation.** Inside a glovebox (argon), a vial was charged with complex **13a** (84.1 mg, 0.0515 mmol) and 1,2,4,5-tetramethylbenzene (45.5 mg, 0.339 mmol) in [D$_6$]-benzene (total volume: 7.0 mL). NMR measurements and the magnetic susceptibility measurements *via* the Evans method were performed with this stock solution. The purity determined by quantitative NMR was 90.3% ±1.0%. The purity was used to correct the values obtained *via* the Evans method (measured from the same stock solution) and the SQUID measurement (measured from the same batch).

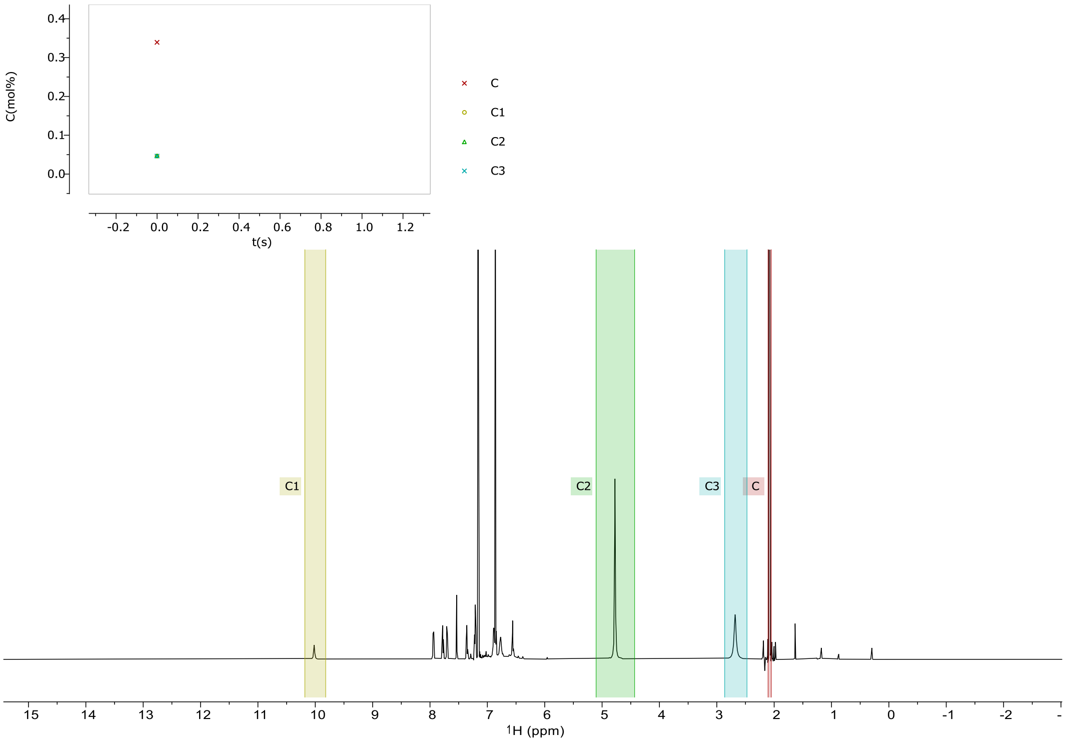

^{1}H NMR spectrum of complex **13a** with 1,2,4,5-tetramethylbenzene (C) as the internal standard: [D$_6$]-benzene, 298 K, 600 MHz.

Table S-1. Parameters of the quantitative NMR experiment.

	Standard	Complex		
		C1	C2	C3
C (mmol)	0.339	0.046005	0.04699	0.046509
M (g/mol)	134.2182	1633.04		
m (mg)	45.50	84.1		
m_{det} (mg)			75.9377	±0.803368
		purity	90.3%	±1.0%

Table S-2. Parameters used in the determination of the effective magnetic moment μ_{eff} *via* Evans method.

$F_{spectrometer}$ (Hz)	600200000	
c (mol/L)	0.00664	± 5.00%
M (g/mol)	1633.03	
χ_D (cm^3/mol)	-8.17*10^{-4}	± 10.00%
c (g/L)	10.85	

Table-S3. Parameters obtained *via* the Evans method.

**T (K)**	**ΔF_1 (Hz)**	**ΔF_2 (Hz)**	**ΔF (Hz)**	**χ_m (l/g)**	**χ_m (cm^3/mol)**	**χ_P (cm^3/mol)**	**μ_{eff} (μ_B)**
298	18.83	19.40	19.12 ± 0.40	7.01*10^{-10}	1.14*10^{-3} ± 6.20*10^{-5}	1.96*10^{-3} ± 1.02*10^{-4}	2.16 ± 0.06

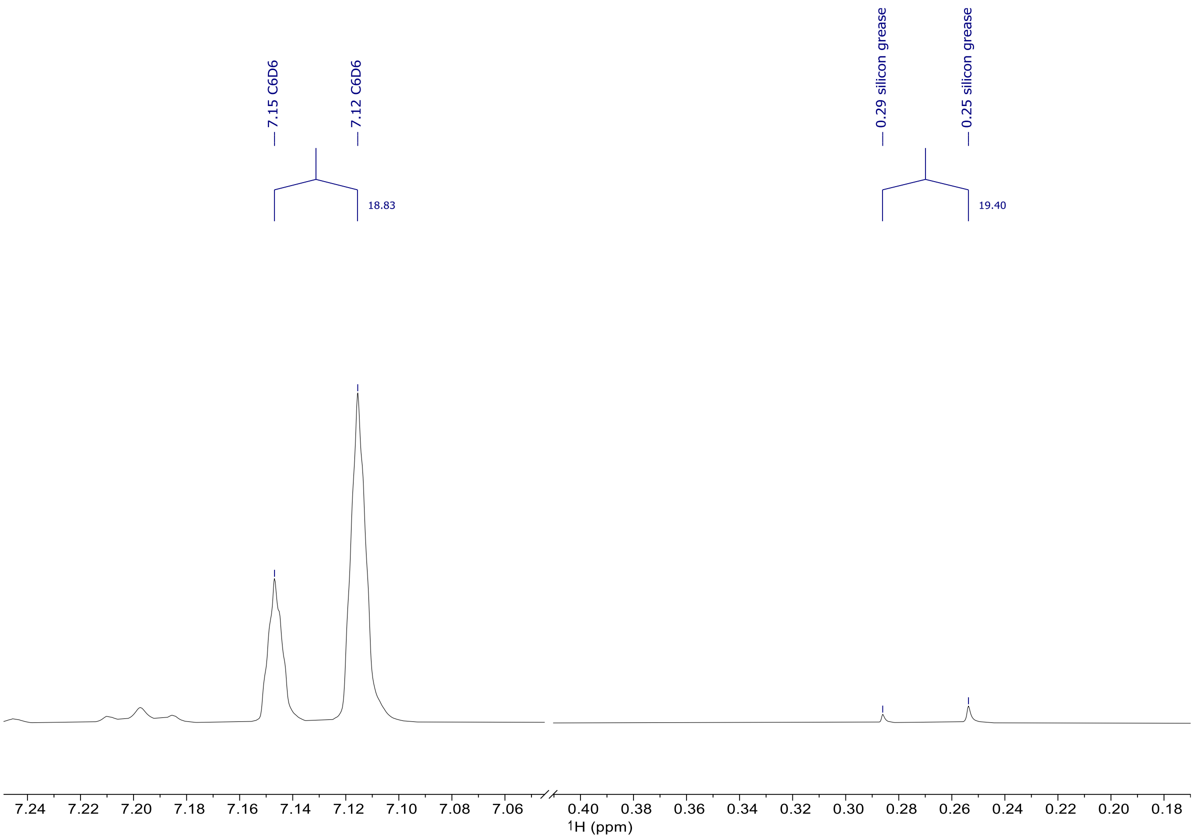

^{1}H NMR spectrum of **13a** for the determination of μ_{eff} *via* the Evans method: [D$_6$]-benzene, 298 K, 600 MHz.

Complex **13b**

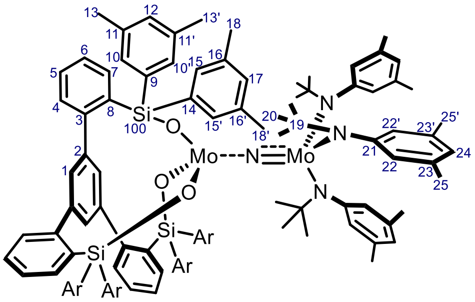

A dark red solution of complex **11** (115 mg, 0.184 mmol) in toluene (1 mL) was added *via* cannula to a light yellow suspension of complex **3b** (206 mg, 0.175 mmol) in toluene (10 mL), causing an immediate color change to dark turquoise. After stirring for 5 min, all volatile components were removed *in vacuo* (10^{-3} mbar) to give a black solid. The residue was washed with *n*-pentane (3 x 3 mL) and the filter cake dried under high vacuum (10^{-3} mbar) at 60 °C to give the title compound as a black solid material (270 mg, 86%). Green single crystals suitable for X-ray diffraction were grown by slow vapor diffusion of *n*-pentane into a solution of the complex in toluene.

Due to its high sensitivity, ^{1}H and ^{13}C NMR spectra contained small amounts of free aniline HN(*t*Bu)(Ar) and the terminal nitrido complex [N≡Mo(N(*t*Bu)(Ar))$_3$] (**10**).

^{1}H NMR (600 MHz, [D$_6$]-benzene, 283 K): δ = 11.42 (s, 3H; H-1), 8.96 (bs, 3H; H-22), 8.30 (d, *J* = 7.8 Hz, 3H; H-4), 7.86−7.81 (m, 6H; H-6 and H-24), 7.79 (d, *J* = 6.9 Hz, 3H; H-7), 7.44 (t, *J* = 7.3 Hz, 3H; H-5), 6.69 (bs; H-15), 6.59 (s, 3H; H-12), 6.02 (s, 3H; H-17), 5.87 (bs; H-15’), 5.70 (bs, 3H; H-22’), 4.83 (s, 27H; H-20), 3.47 (bs, 9H; H-25), 2.33 (bs, 9H; H-25’), 1.78 (s, 18H; H-13 and H-13’), 1.67 (bs; H-18’), 1.49 ppm (bs; H-18) (Note: the signals for H-10 and H-10’ were not found at 283 K due to their broad linewidths at this temperature).

^{13}C NMR (151 MHz, [D$_6$]-benzene, 283 K): δ = 520.3 (bs, C-19), 289.7, 270.9, 244.6 (C-8), 170.0, 164.3 (bs), 161.2 (bs), 156.5 (bs), 153.2 (C-2), 149.3 (C-7), 147.6 (C-3), 142.1 (bs, C-15), 139.2 (bs), 138.0, 137.9 (bs), 135.5, 134.9 (bs), 134.1 (bs), 133.2, 133.1, 132.2 (bs, C-10 and C10’), 131.8 (C-6), 131.0 (C-12), 130.2 (C-5), 127.2 (C-1), 81.5 (C-20), 26.6 (bs, C-25), 22.5 (C-18), 21.9 (C-13 and C-13’), 21.4, 20.2 ppm (Note: not all signals could assigned based on the 2D NMR data due line broadening at 283 K.).

^{29}Si NMR (119 MHz, [D$_6$]-benzene, 283 K): δ = −117.7 ppm.

IR (ATR): $\tilde{\nu}$ = 2967, 2918, 1585, 1555, 1411, 1385, 1357, 1286, 1266, 1178, 1138, 1087, 1045, 1000, 985, 941, 921, 902, 846, 763, 728, 717, 698, 689, 635, 623, 580, 558, 551, 535 cm^{-1}.

HRMS (ESI$^+$): *m/z* calculated for $C_{108}H_{123}Mo_2N_4O_3Si_3$ [M]$^+$: 1803.70057, found: 1803.70276.

Elemental analysis (%) calculated for $C_{108}H_{123}Mo_2N_4O_3Si_3$: C 72.01, H 6.88, Mo 10.65, N 3.11, Si 4.68; found: C 71.90, H 6.84, Mo 10.59, N 3.07, Si 4.61.

REFERENCES

[1] D. M. T. Chan, M. H. Chisholm, K. Folting, J. C. Huffman, N. S. Marchant, *Inorg. Chem.* **1986**, *25*, 4170-4174.

[2] C. E. Laplaza, M. J. A. Johnson, J. C. Peters, A. L. Odom, E. Kim, C. C. Cummins, G. N. George, I. J. Pickering, *J. Am. Chem. Soc.* **1996**, *118*, 8623-8638.

[3] J. J. Curley, E. L. Sceats, C. C. Cummins, *J. Am. Chem. Soc.* **2006**, *128*, 14036-14037.

[4] M. J. A. Johnson, P. M. Lee, A. L. Odom, W. M. Davis, C. C. Cummins, *Angew. Chem. Int. Ed. Engl.* **1997**, *36*, 87-91.

[5] C. E. Laplaza, A. L. Odom, W. M. Davis, C. C. Cummins, J. D. Protasiewicz, *J. Am. Chem. Soc.* **1995**, *117*, 4999-5000.

[6] A. Fürstner, C. Mathes, C. W. Lehmann, *Chem. Eur. J.* **2001**, *7*, 5299-5317.

[7] J. J. Curley, Dissertation, Massachusetts Institute of Technology, **2009**.

[8] J. Hillenbrand, M. Leutzsch, A. Fürstner, *Angew. Chem.* **2019**, *131*, 15837-15843; *Angew. Chem. Int. Ed.* **2019**, *58*, 15690-15696.

[9] J. Hillenbrand, M. Leutzsch, E. Yiannakas, C. P. Gordon, C. Wille, N. Nöthling, C. Copéret, A. Fürstner, *J. Am. Chem. Soc.* **2020**, *142*, 11279-11294.

[10] J. Hillenbrand, M. Leutzsch, C. P. Gordon, C. Copéret, A. Fürstner, *Angew. Chem.* **2020**, *132*, 21942-21952; *Angew. Chem. Int. Ed.* **2020**, *59*, 21758-21768.

[11] R. K. Harris, E. D. Becker, S. M. C. d. Menezes, P. Granger, R. E. Hoffman, K. W. Zilm, *Pure Appl. Chem.* **2008**, *80*, 59-84.

[12] G. A. Bain, J. F. Berry, Diamagnetic Corrections and Pascal's Constants. *J. Chem. Educ.* **2008**, *85*, 532–536.

[13] E. Bill, JulX20 version 1.4, Max Planck Institute for Chemical Energy Conversion.

[14] The average g-value refers to the powder average, given by Eq. 2

$$g_{avg} = \sqrt{(g_x{}^2 + g_y{}^2 + g_z{}^2)/3} \quad (2)$$

[15] E. S. Wiedner, K. J. Gallagher, M. J. A. Johnson, J. W. Kampf, *Inorg. Chem.* **2011**, *50*, 5936-5945.
