# Supplementary material for: Molybdenum(VI) Nitrido Complexes with Tripodal Silanolate Ligands. Structure and Electronic Character of an Unsymmetrical Dimolybdenum μ-Nitrido Complex Formed by Incomplete Nitrogen Atom Transfer
Source: Inorg Chem. 2024 Apr 25;63(18):8376–89. doi: 10.1021/acs.inorgchem.4c00762 (PMC11080062; doi:10.1021/acs.inorgchem.4c00762)

SUPPORTING INFORMATION

Part II

COPIES OF SPECTRA

Molybdenum(VI) Nitrido Complexes with Tripodal Silanolate Ligands.

Structure and Electronic Character of an Unsymmetrical Dimolybdenum μ-Nitrido Complex Formed by Incomplete Nitrogen Atom Transfer

Daniel Rütter, Maurice van Gastel, Markus Leutzsch, Nils Nöthling, Daniel SantaLucia,[+] Frank Neese,* and Alois Fürstner*

Max-Planck-Institut für Kohlenforschung, 45470 Mülheim/Ruhr, Germany
[+] *Max-Planck-Institut für Chemische Energiekonversion, 45470 Mülheim/Ruhr, Germany*

Email: fuerstner@kofo.mpg.de; neese@kofo.mpg.de

[1]**H NMR spectrum of** Bis(3,5-dimethylphenyl)dimethoxysilane **(S1):** [D_2]-dichloromethane, 298 K, 600 MHz.

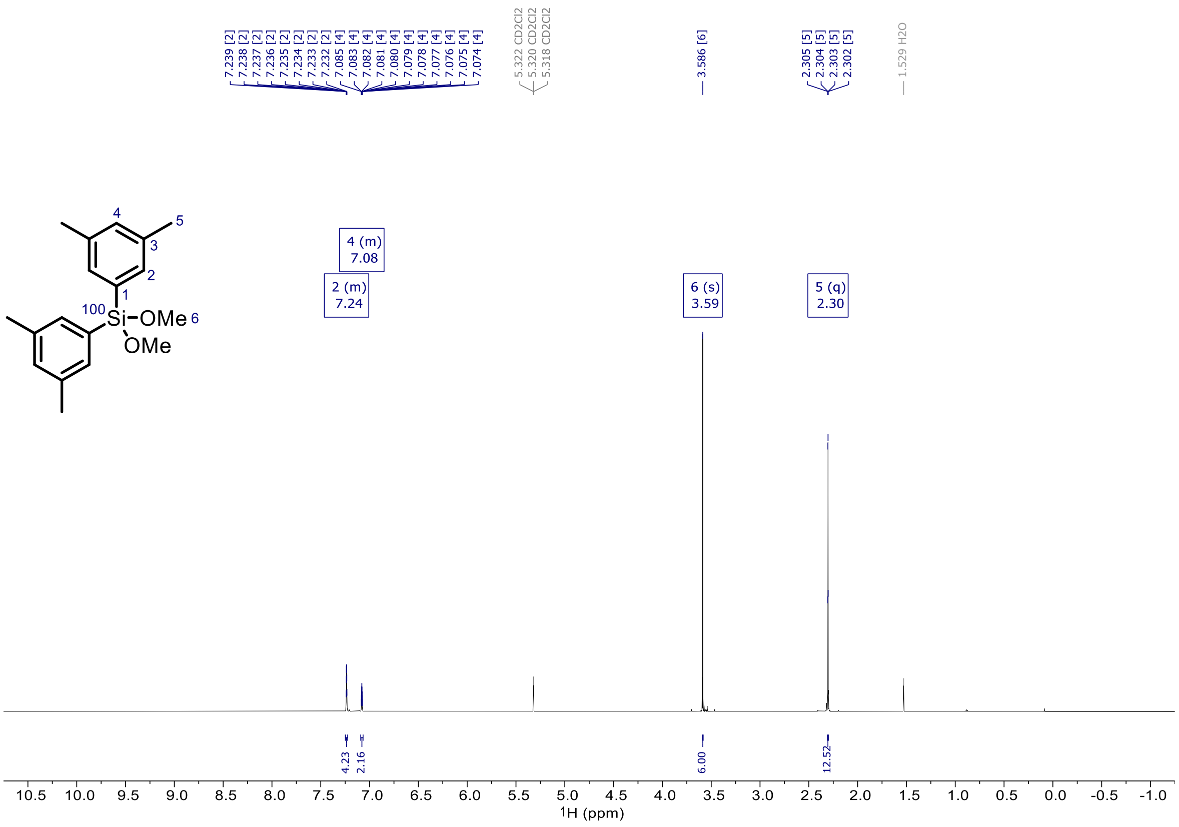

[13]**C NMR spectrum of Bis(3,5-dimethylphenyl)dimethoxysilane (S1):** [D_2]-dichloromethane, 298 K, 151 MHz.

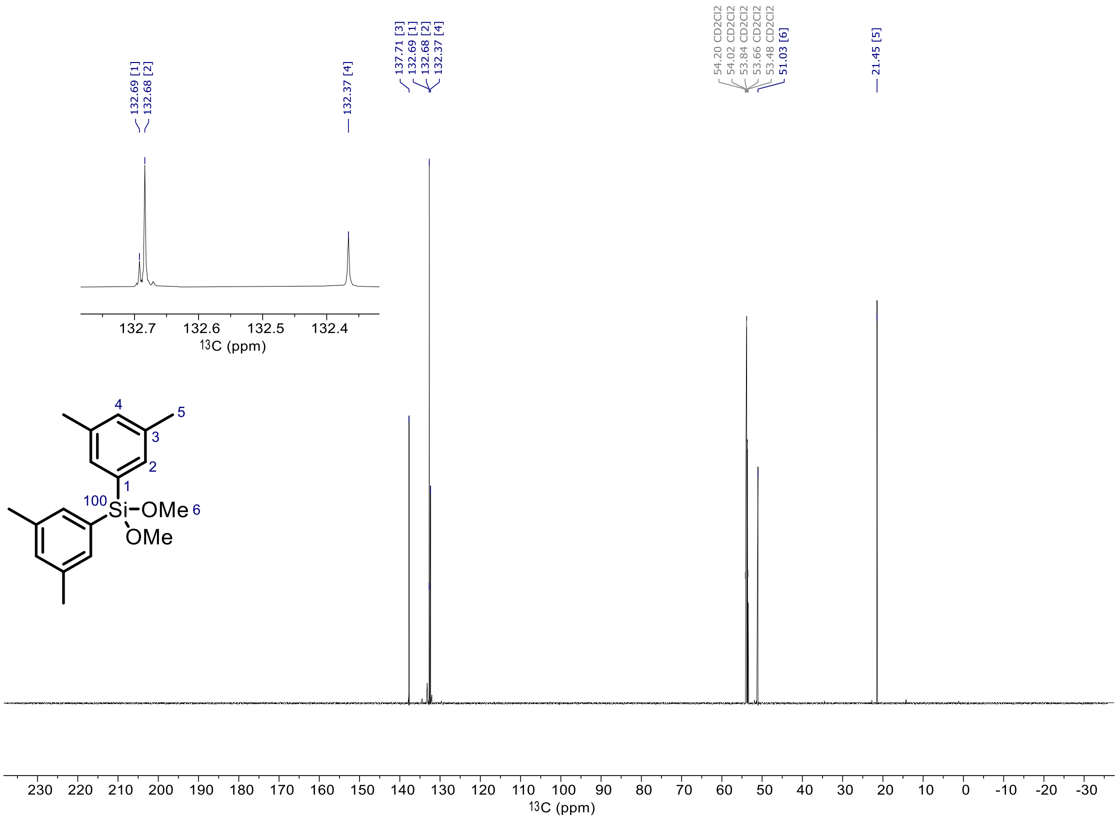

^{1}H-^{13}C HSQC NMR spectrum of Bis(3,5-dimethylphenyl)dimethoxysilane (S1): [D_2]-dichloromethane, 298 K, 600 MHz, 151 MHz.

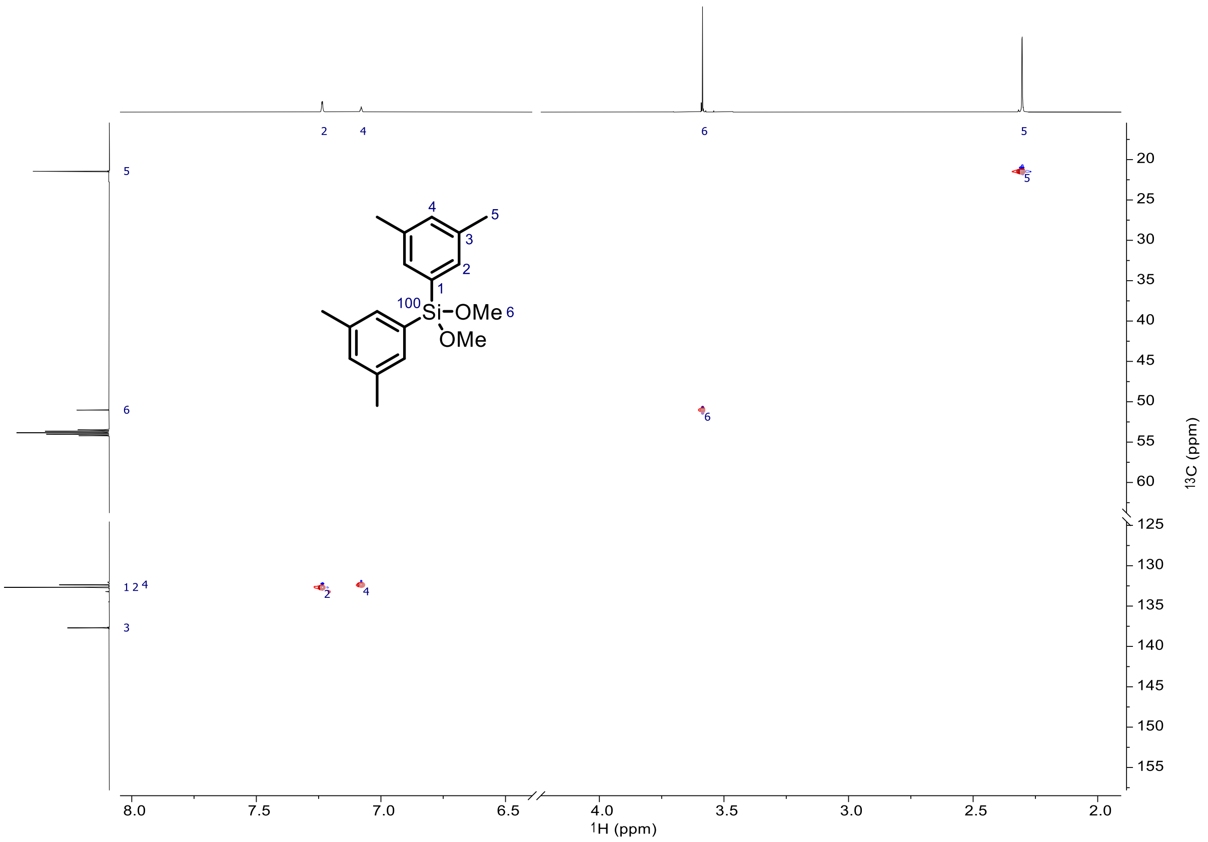

^{1}H-^{13}C HMBC NMR spectrum of Bis(3,5-dimethylphenyl)dimethoxysilane (S1): [D_2]-dichloromethane, 298 K, 600 MHz, 151 MHz.

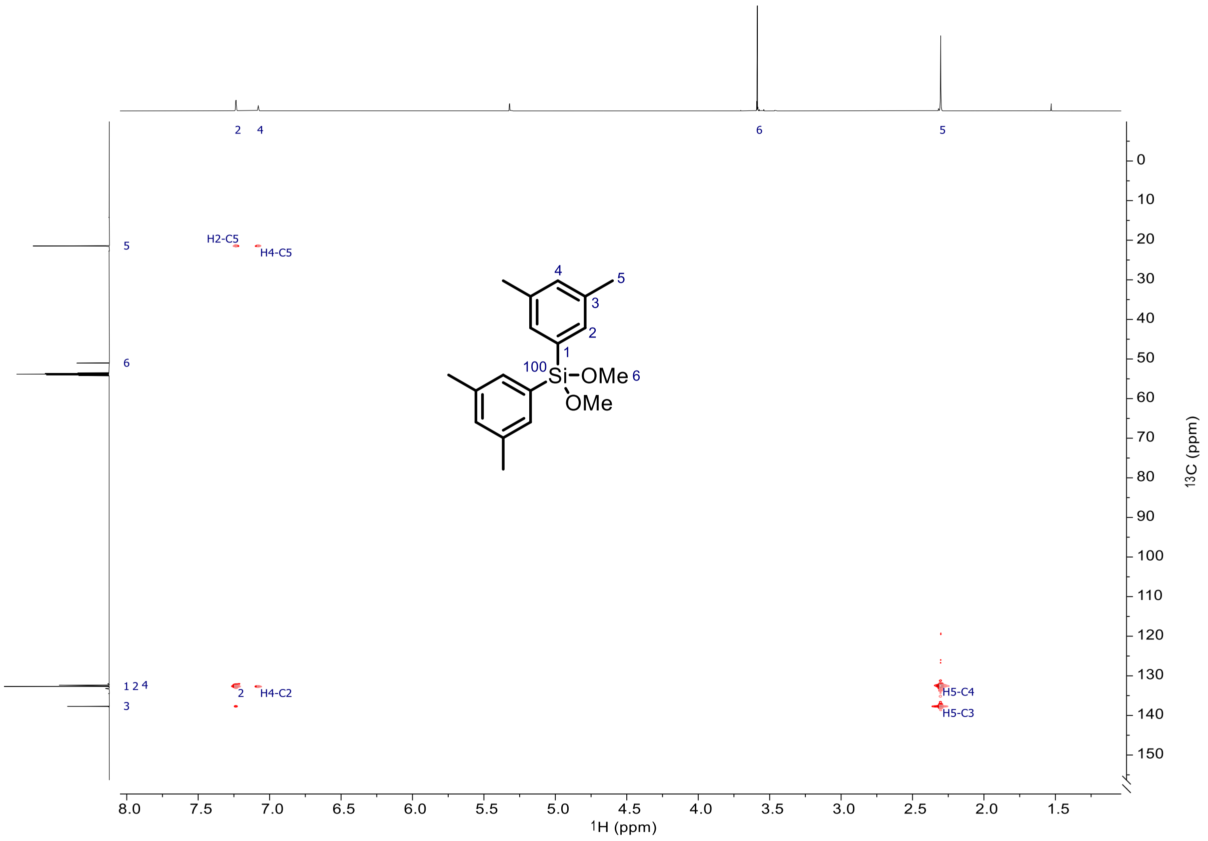

^{1}H-^{1}H COSY NMR spectrum of Bis(3,5-dimethylphenyl)dimethoxysilane (S1): [D$_2$]-dichloromethane, 298 K, 600 MHz, 600 MHz.

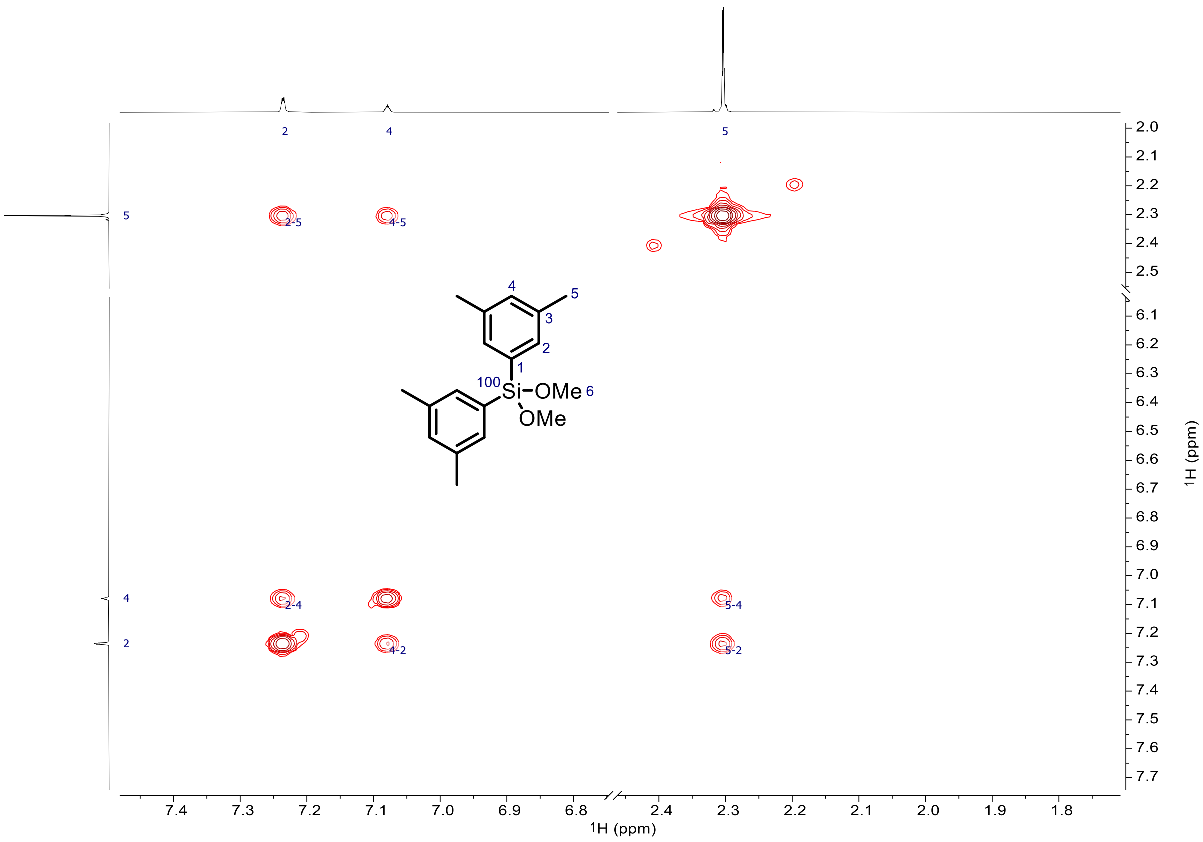

Bis(3,5-dimethylphenyl)dimethoxysilane (S1): ^{29}Si NMR spectrum ([D$_2$]-dichloromethane, 298 K, 119 MHz) and **^{1}H-^{29}Si HMBC NMR spectrum** ([D$_2$]-dichloromethane, 298 K, 600 MHz, 119 MHz).

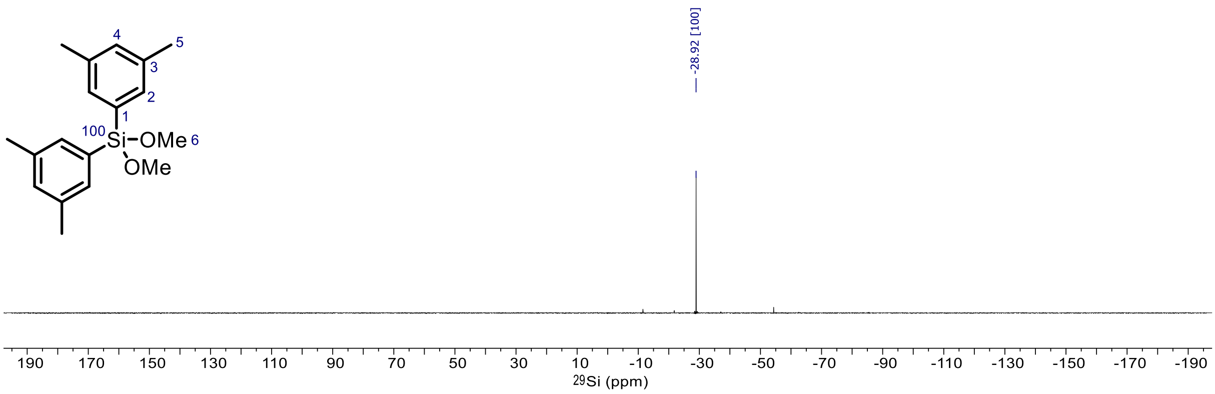

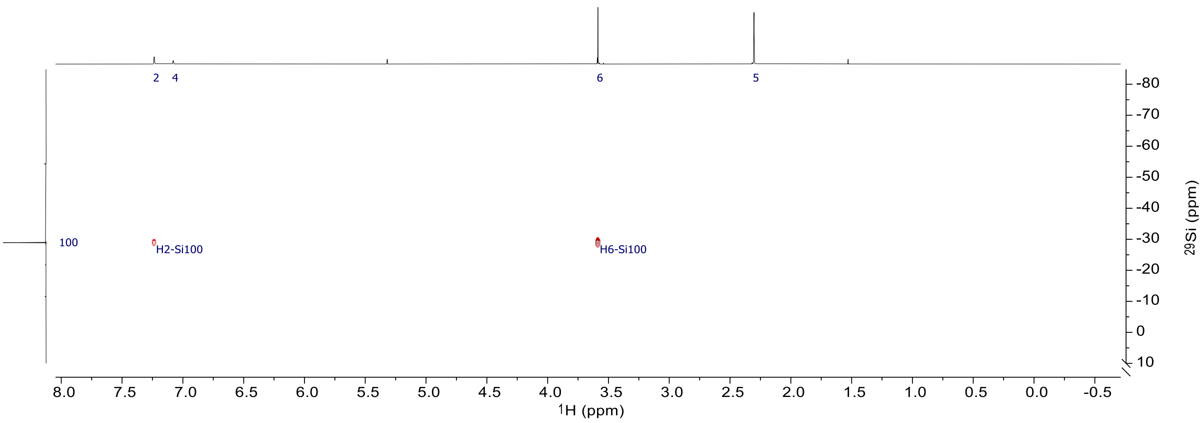

NMR analysis of ligand 7b: NMR analysis indicates that ligand **7b** is C_3 symmetric at 298 K. It was not possible to assign C9, presumably because it is overlapping with C10, C11 or C12 and does not show a cross peak in the ^{1}H-^{13}C HMBC NMR spectrum.

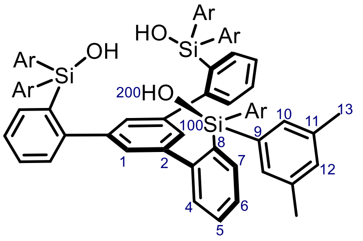

^{1}H NMR spectrum of ligand 7b: [D$_2$]-dichloromethane, 298 K, 600 MHz.

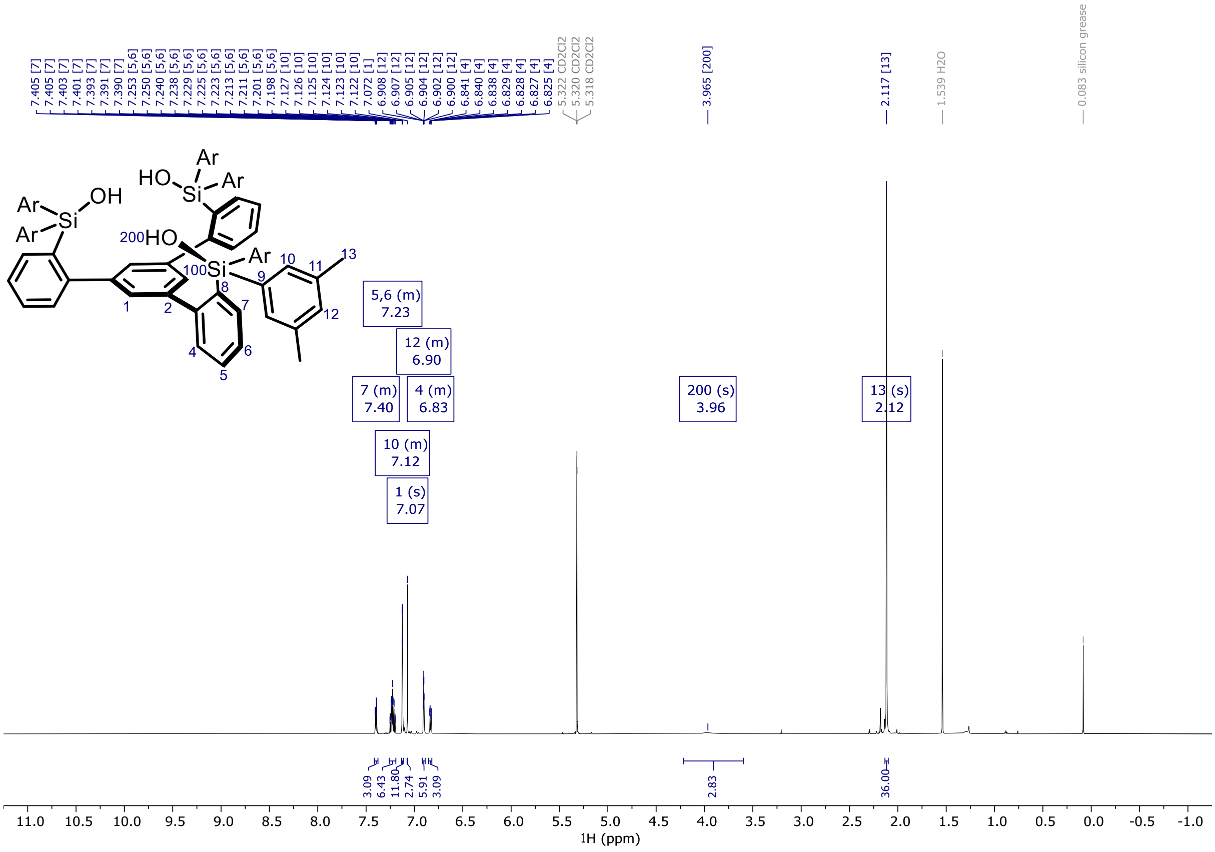

^{13}C NMR spectrum of ligand 7b: [D_2]-dichloromethane, 298 K, 151 MHz.

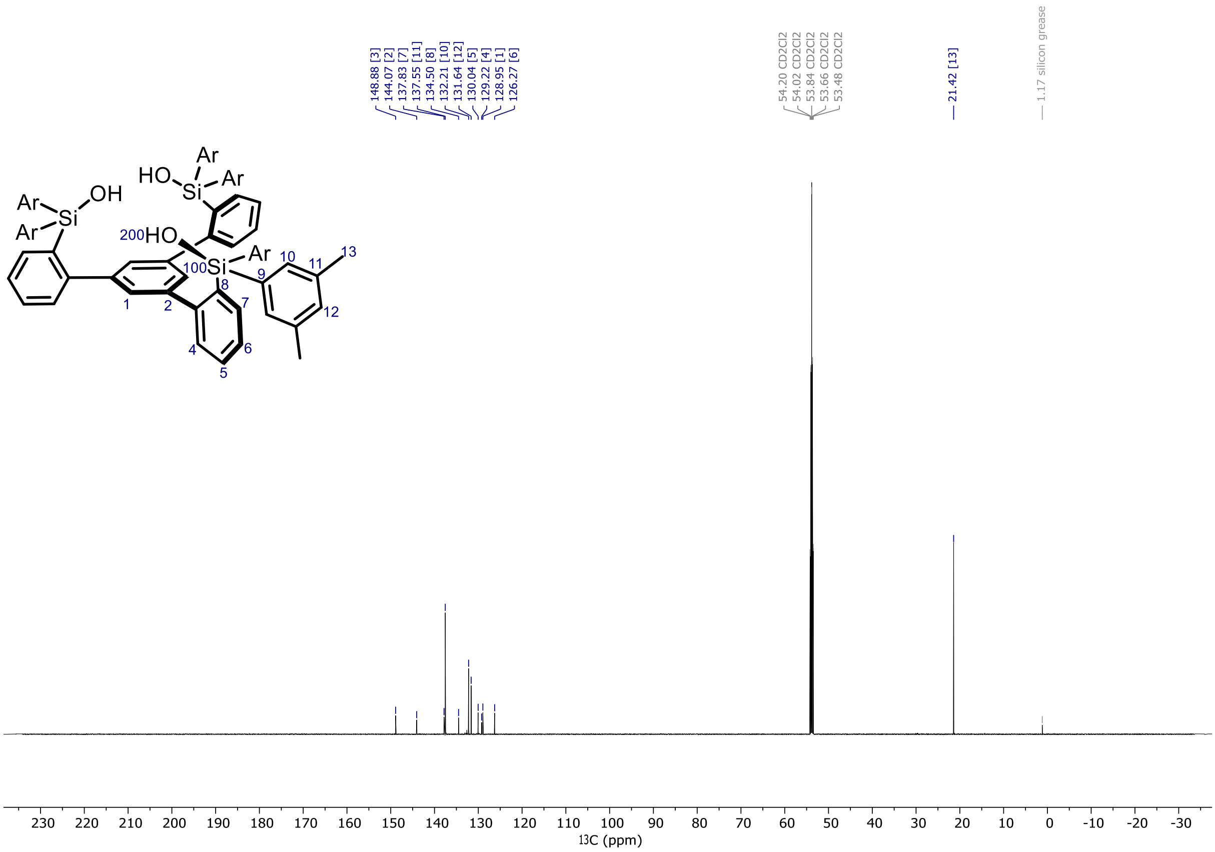

^{1}H-^{13}C HSQC NMR spectrum of ligand 7b: [D_2]-dichloromethane, 298 K, 600 MHz, 151 MHz.

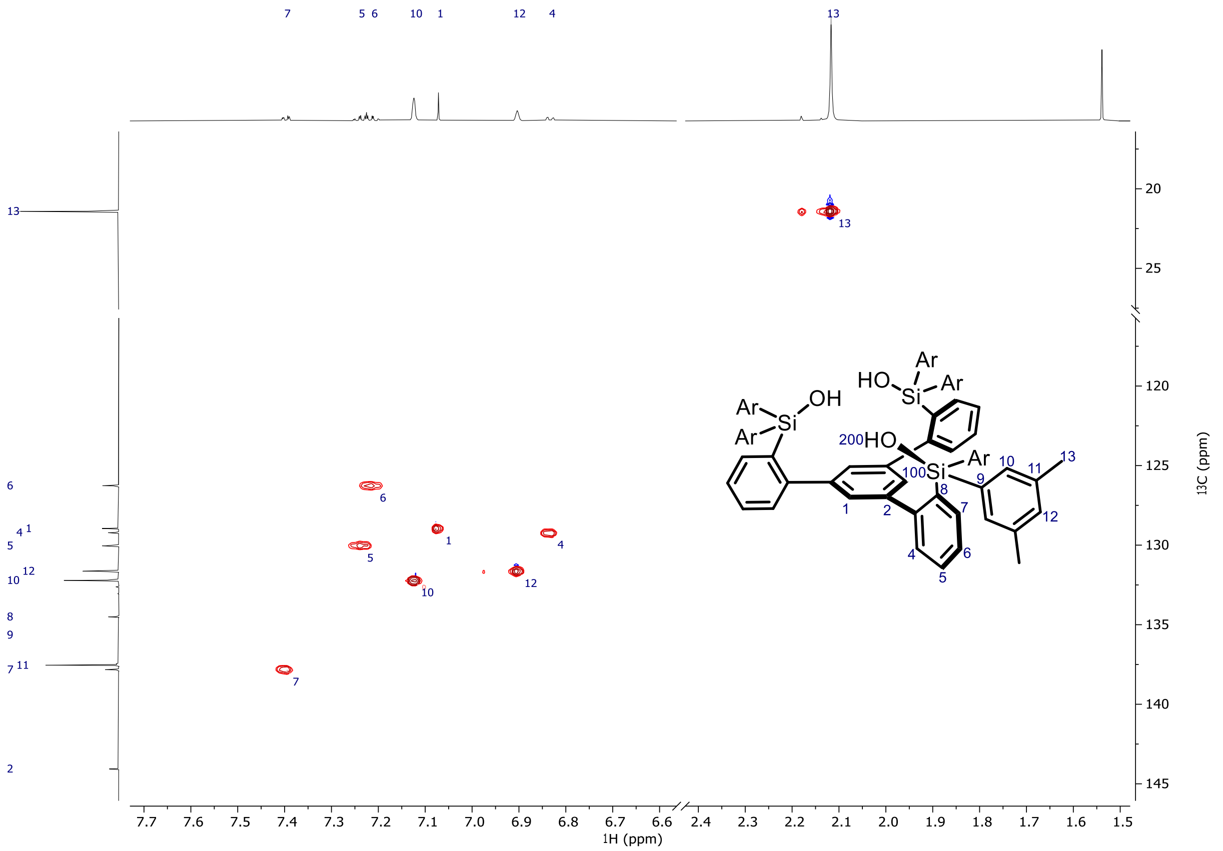

^{1}H-^{13}C HMBC NMR spectrum of ligand 7b: [D_2]-dichloromethane, 298 K, 600 MHz, 151 MHz.

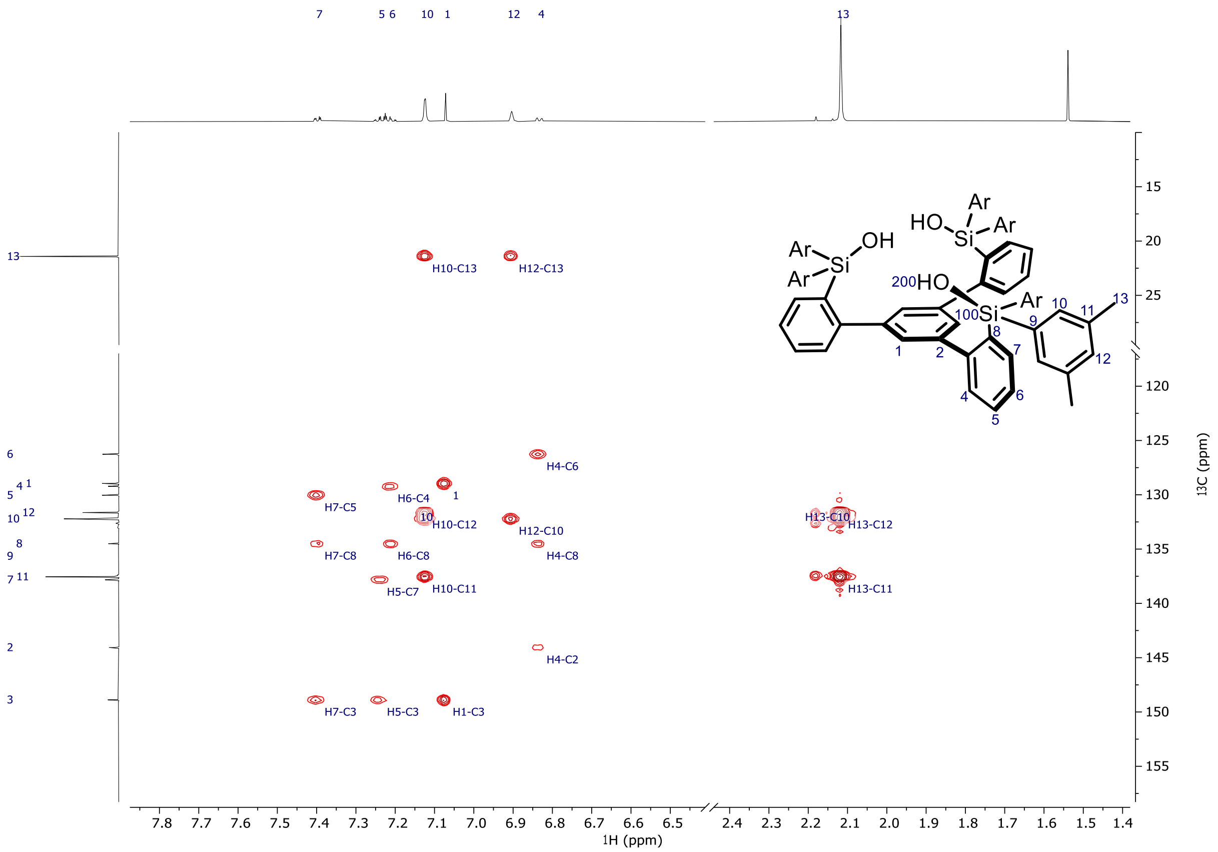

^{1}H-^{1}H COSY NMR spectrum of ligand 7b: [D_2]-dichloromethane, 298 K, 600 MHz, 600 MHz.

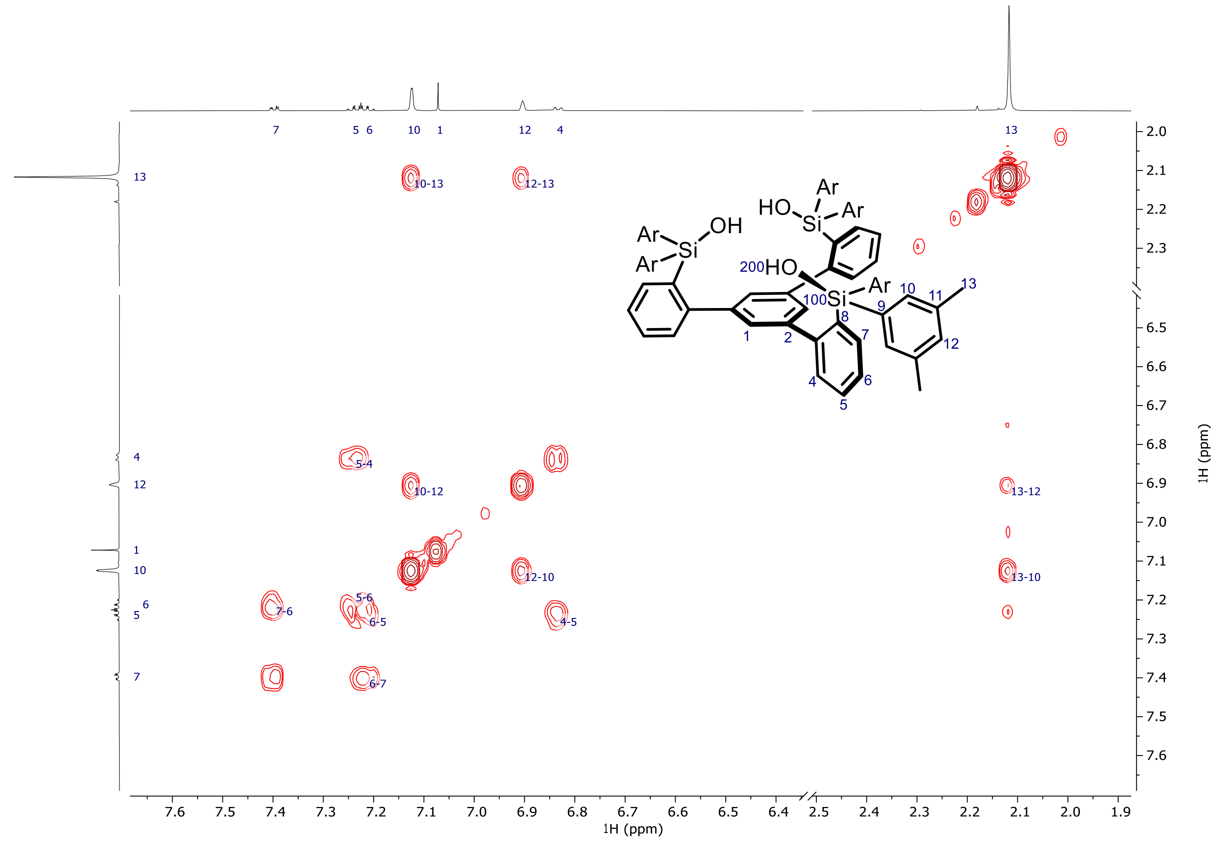

^{29}Si NMR spectrum of ligand 7b: [D$_2$]-dichloromethane, 298 K, 119 MHz.

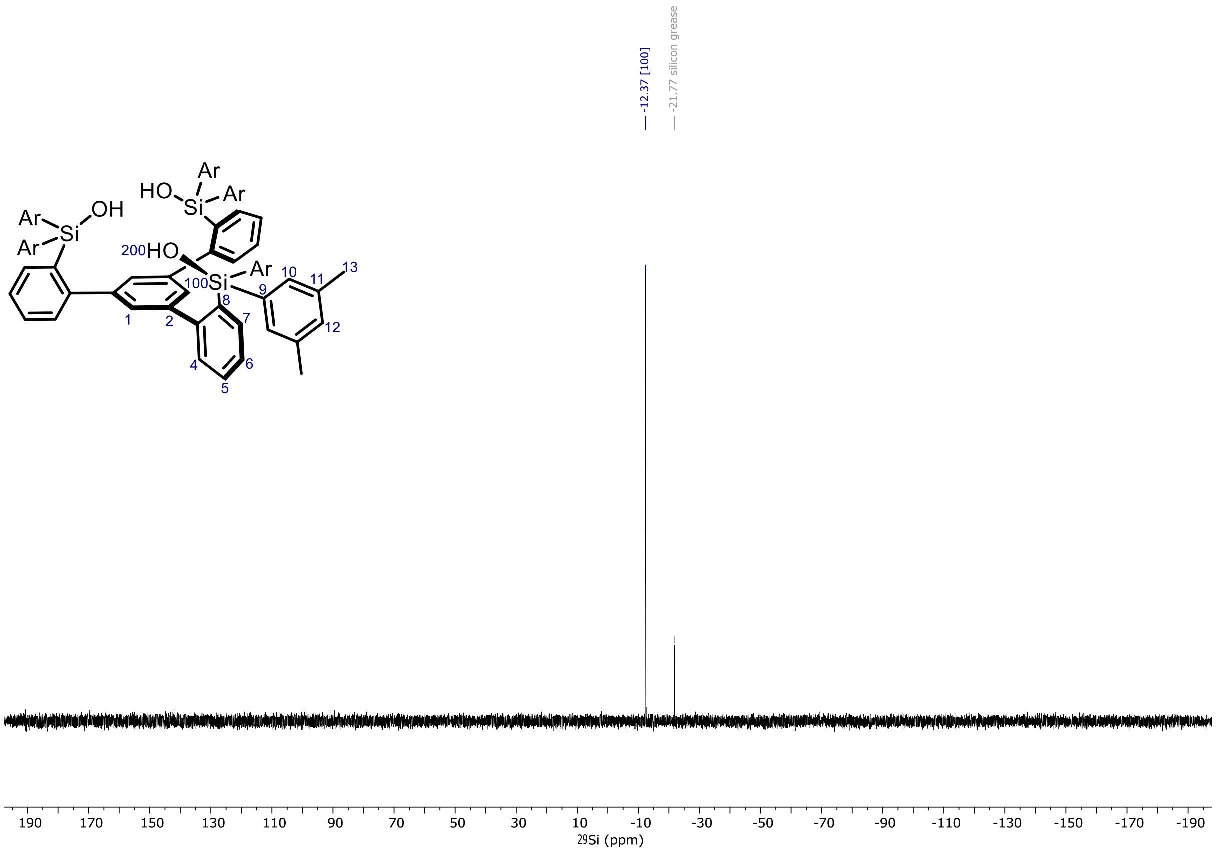

^{1}H-^{29}Si HMBC NMR spectrum of ligand 7b: [D$_2$]-dichloromethane, 298 K, 600 MHz, 119 MHz.

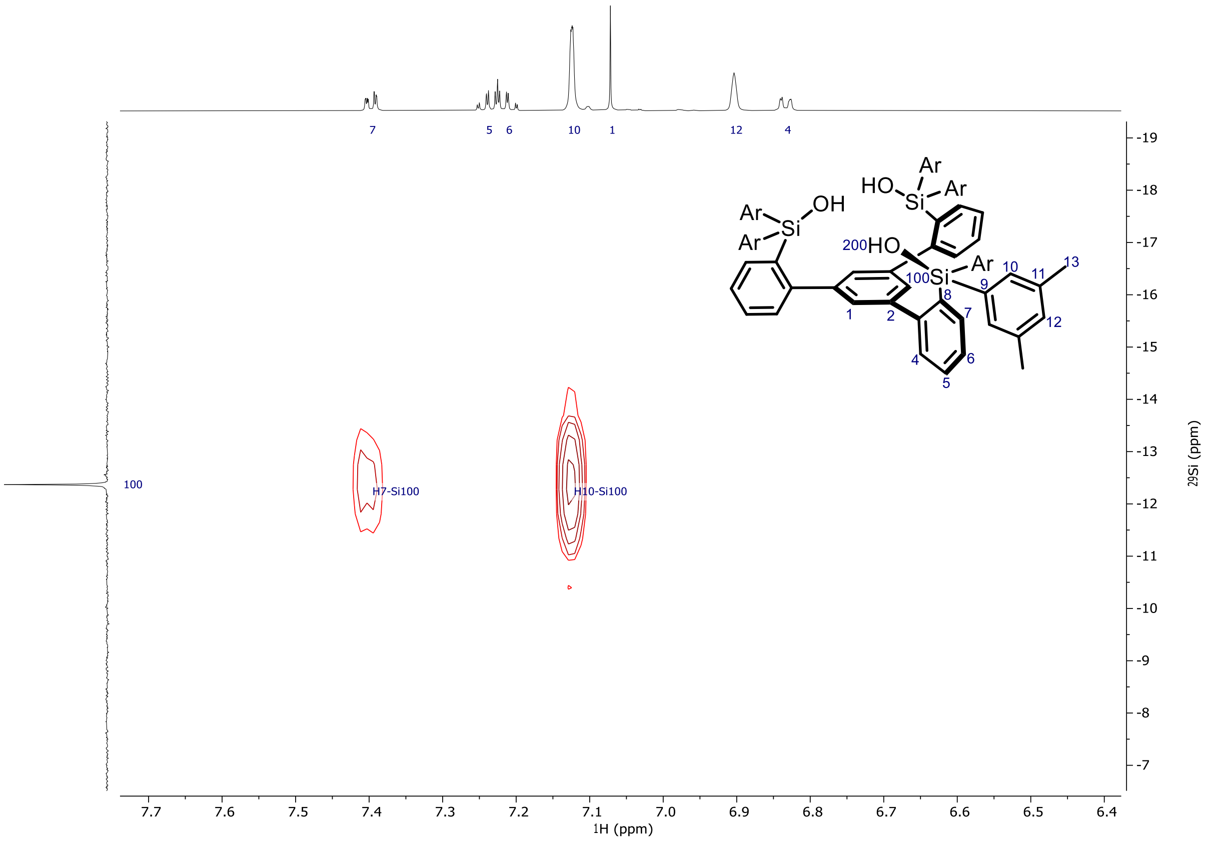

NMR analysis of silane S2: NMR analysis indicates that this silane is C_3 symmetric at 298 K. The signals of C1 and C4 are overlapping in the ^{13}C NMR spectrum and had to be assigned from the ^{1}H-^{13}C HMBC NMR spectrum.

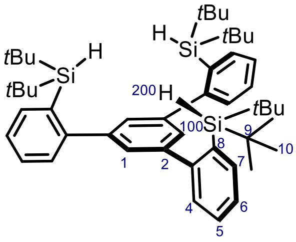

^{1}H NMR spectrum of silane S2: [D$_2$]-dichloromethane, 298 K, 600 MHz.

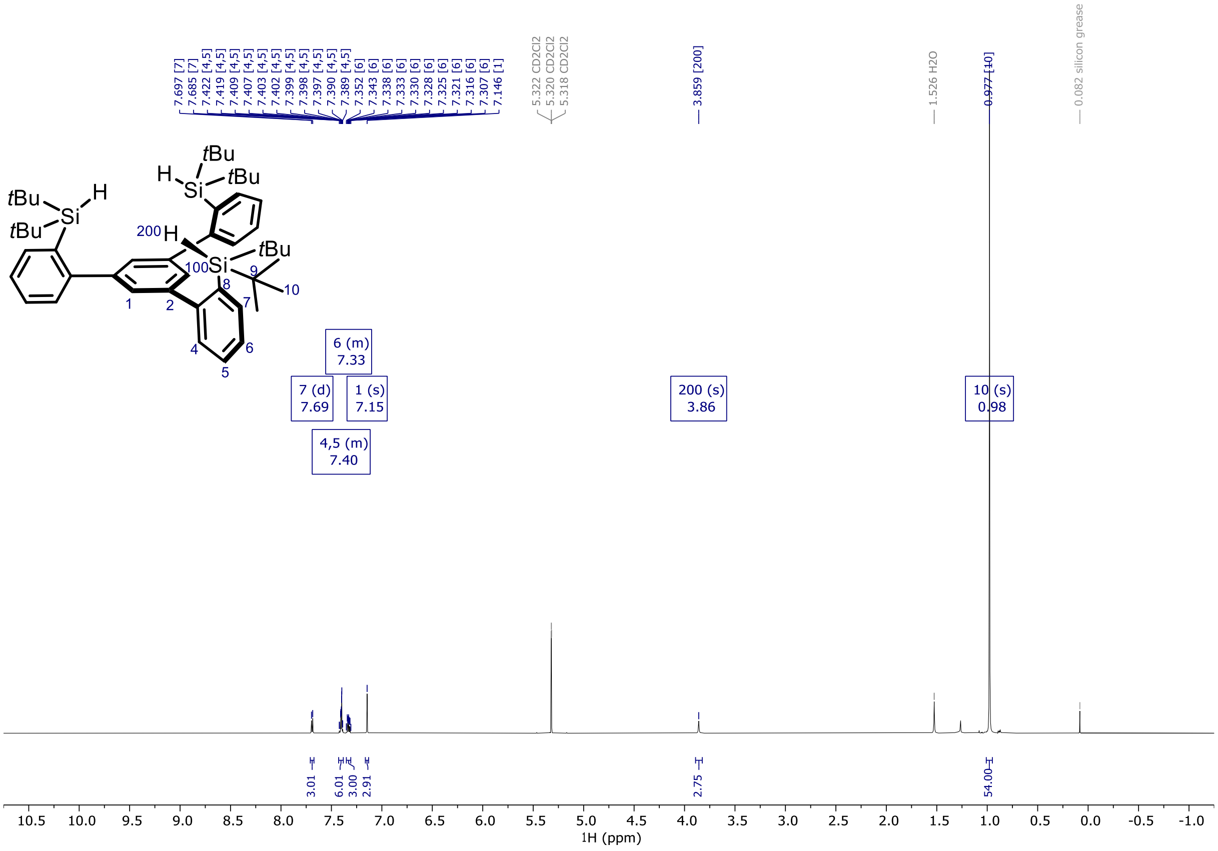

^{13}C NMR spectrum of silane S2: [D$_2$]-dichloromethane, 298 K, 151 MHz.

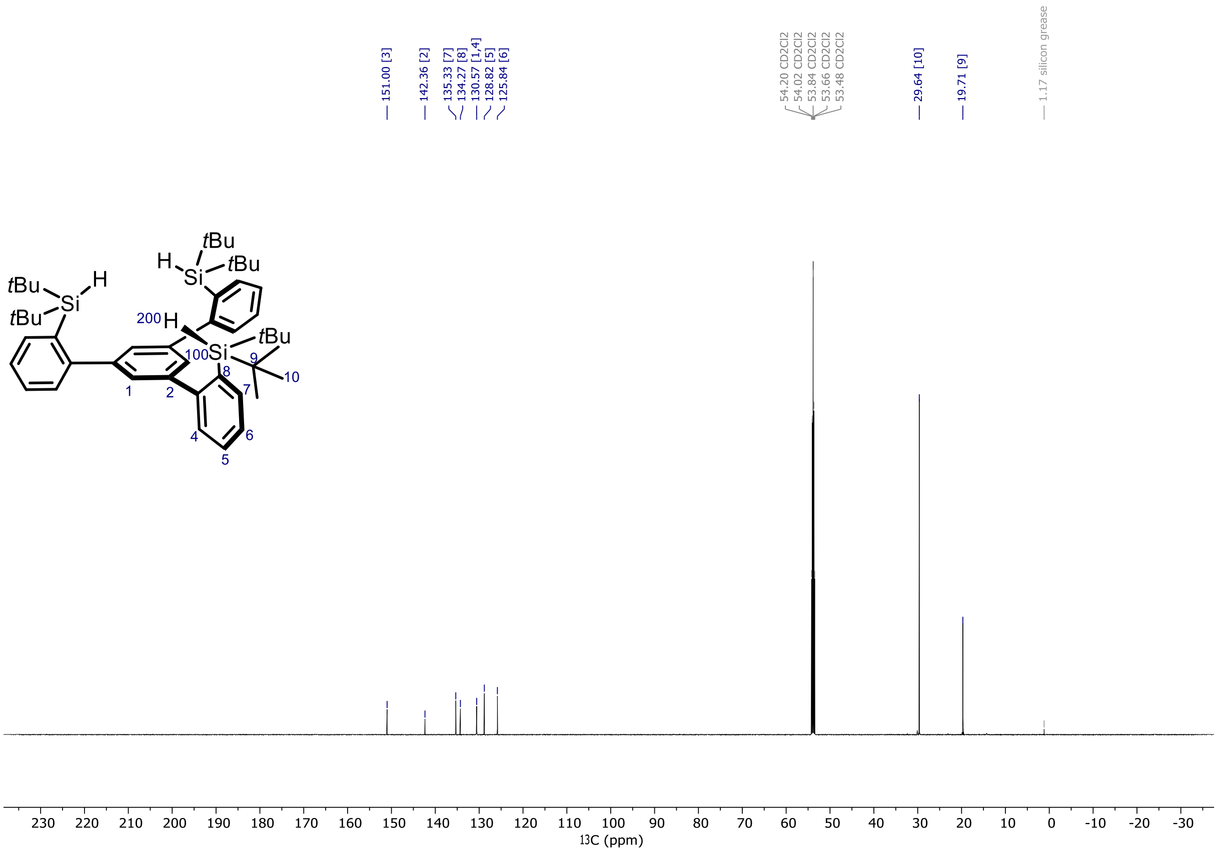

^{1}H-^{13}C HSQC NMR spectrum of silane S2: [D$_2$]-dichloromethane, 298 K, 600 MHz, 151 MHz.

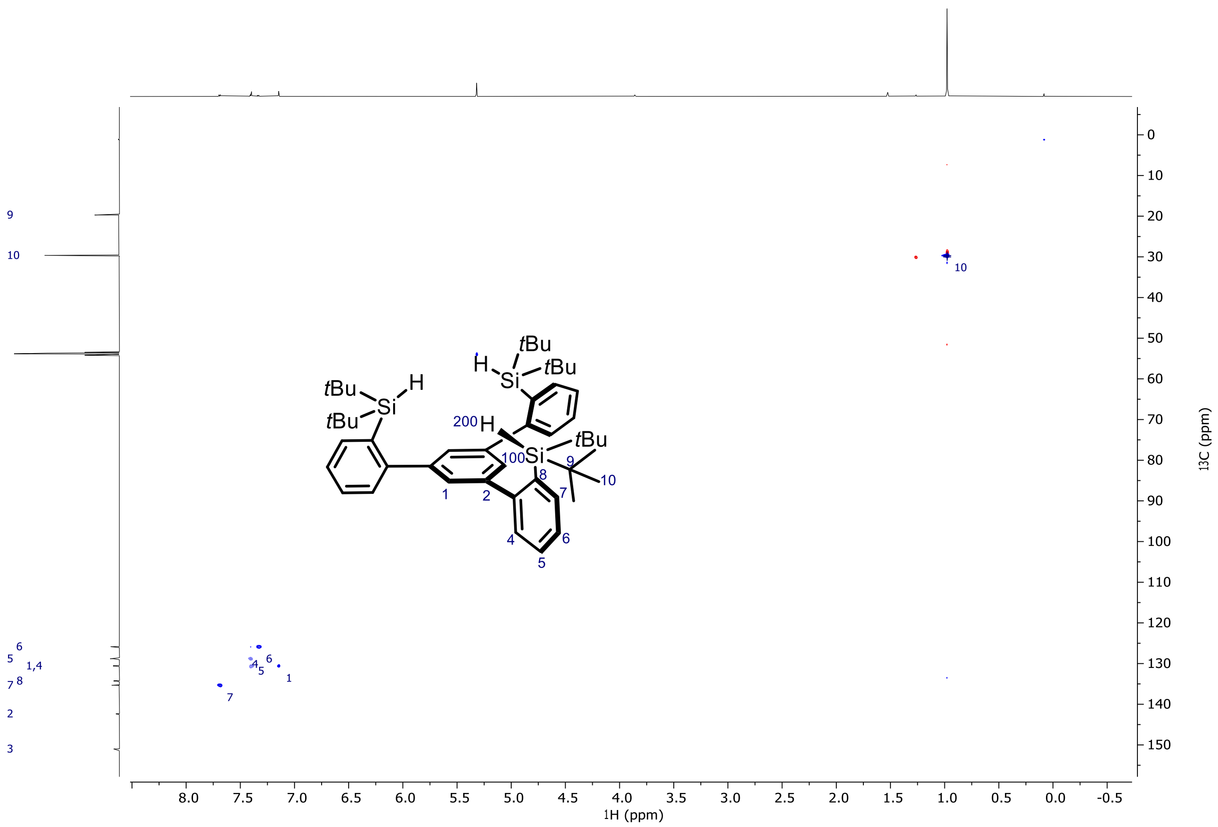

^{1}H-^{13}C HMBC NMR spectrum of silane S2: [D$_2$]-dichloromethane, 298 K, 600 MHz, 151 MHz.

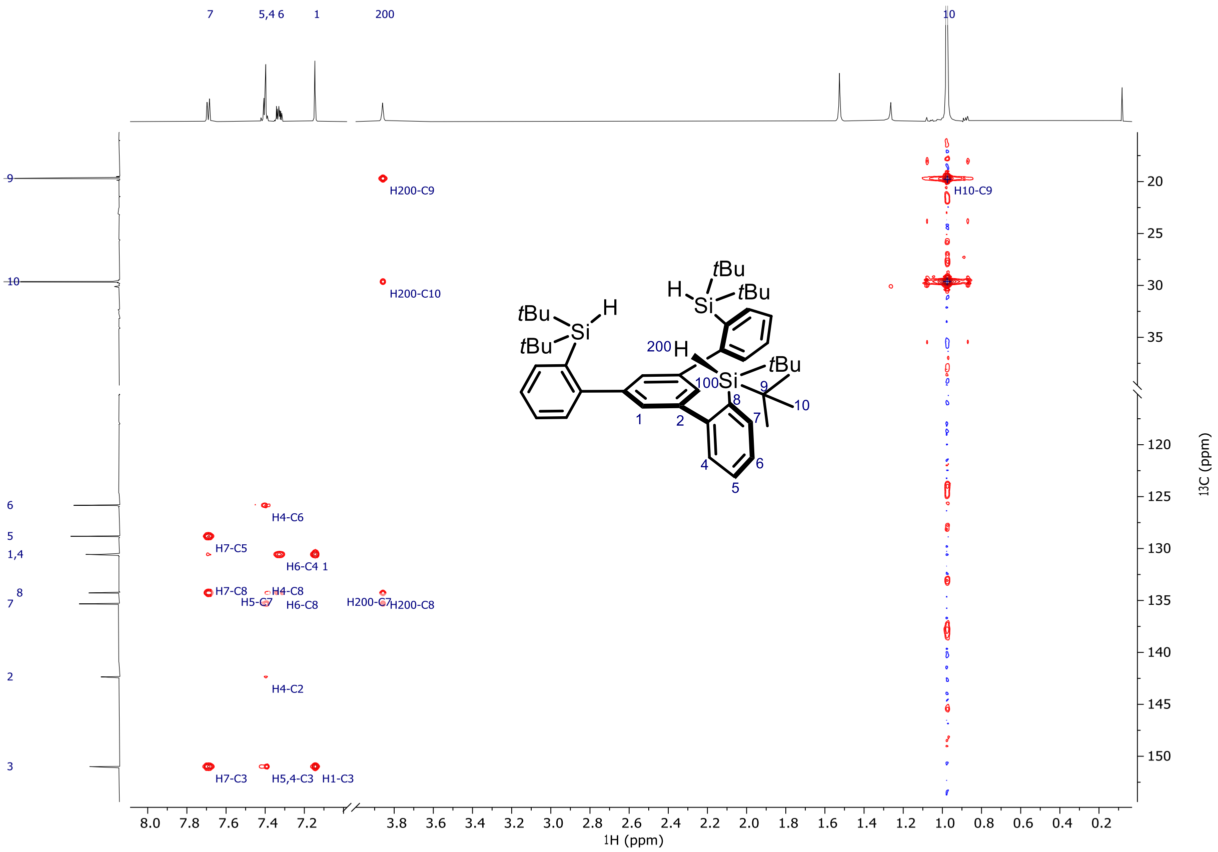

^{1}H-^{1}H COSY NMR spectrum of silane S2: [D$_2$]-dichloromethane, 298 K, 600 MHz, 600 MHz.

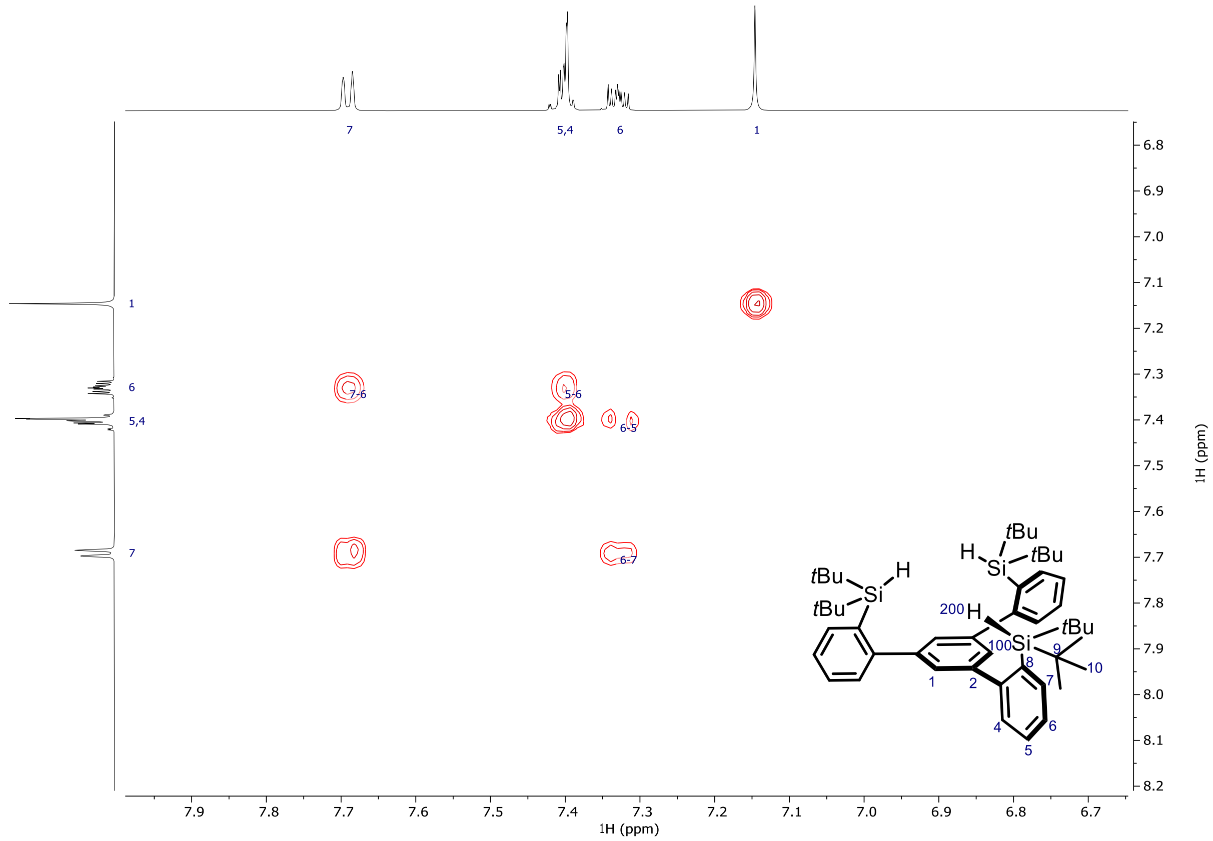

^{1}H-^{1}H NOESY NMR spectrum of silane S2: [D$_2$]-dichloromethane, 298 K, 600 MHz, 600 MHz.

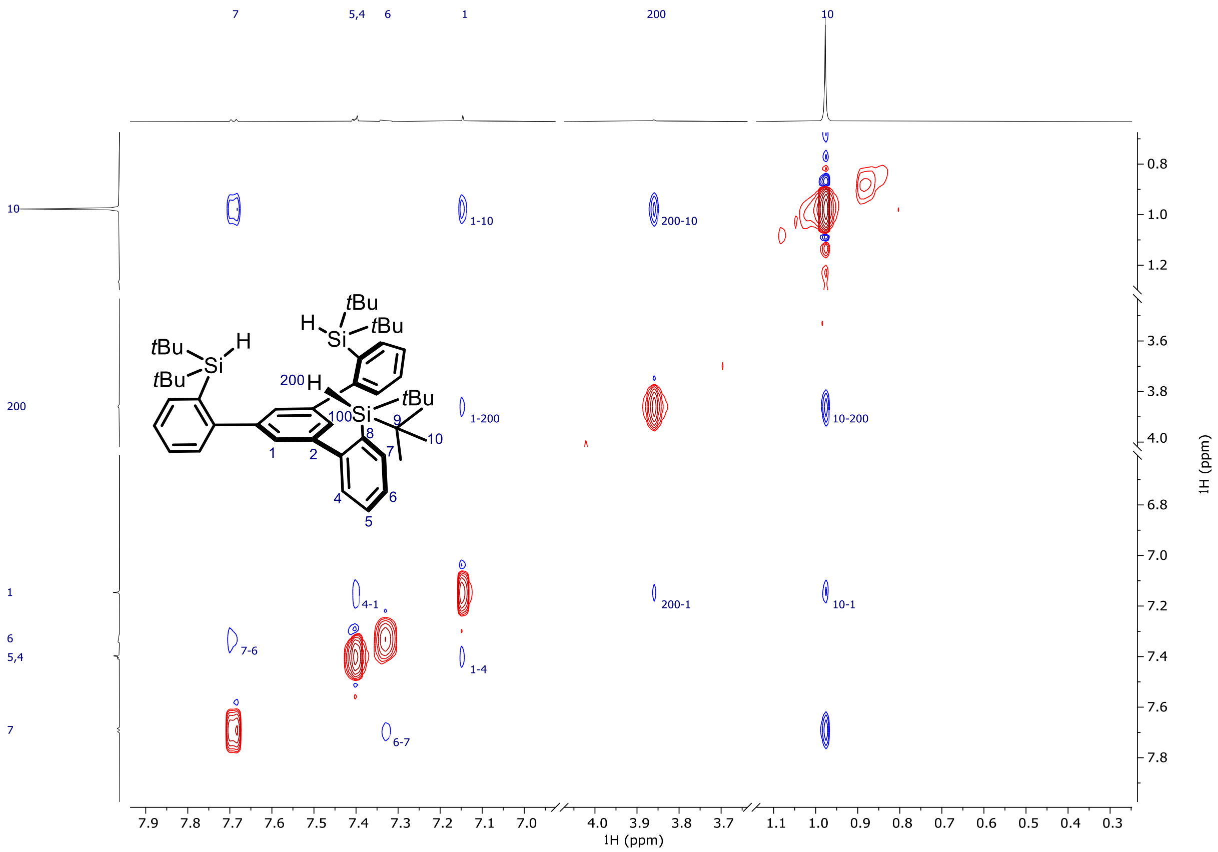

^{29}Si NMR spectrum of silane S2: [D$_2$]-dichloromethane, 298 K, 119 MHz.

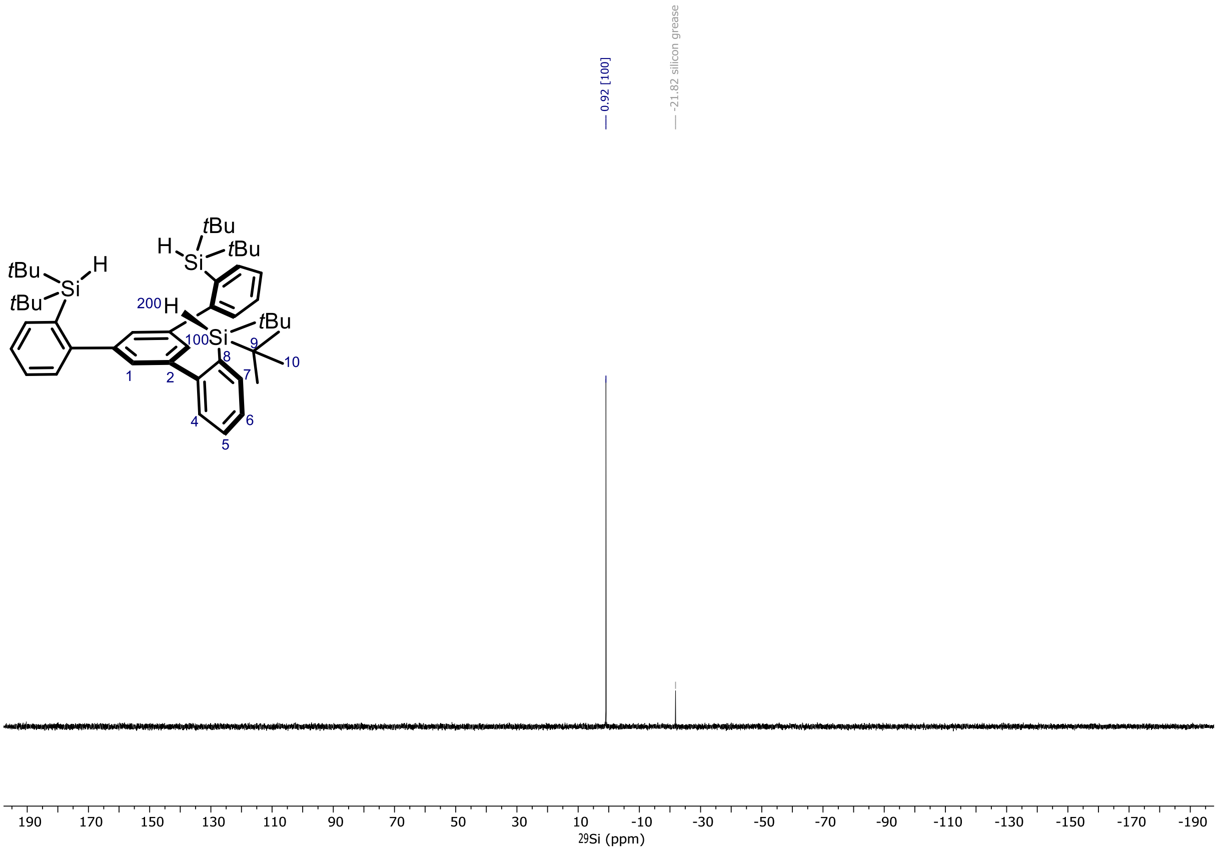

^{1}H-^{29}Si HMBC NMR spectrum of silane S2: [D$_2$]-dichloromethane, 298 K, 600 MHz, 119 MHz.

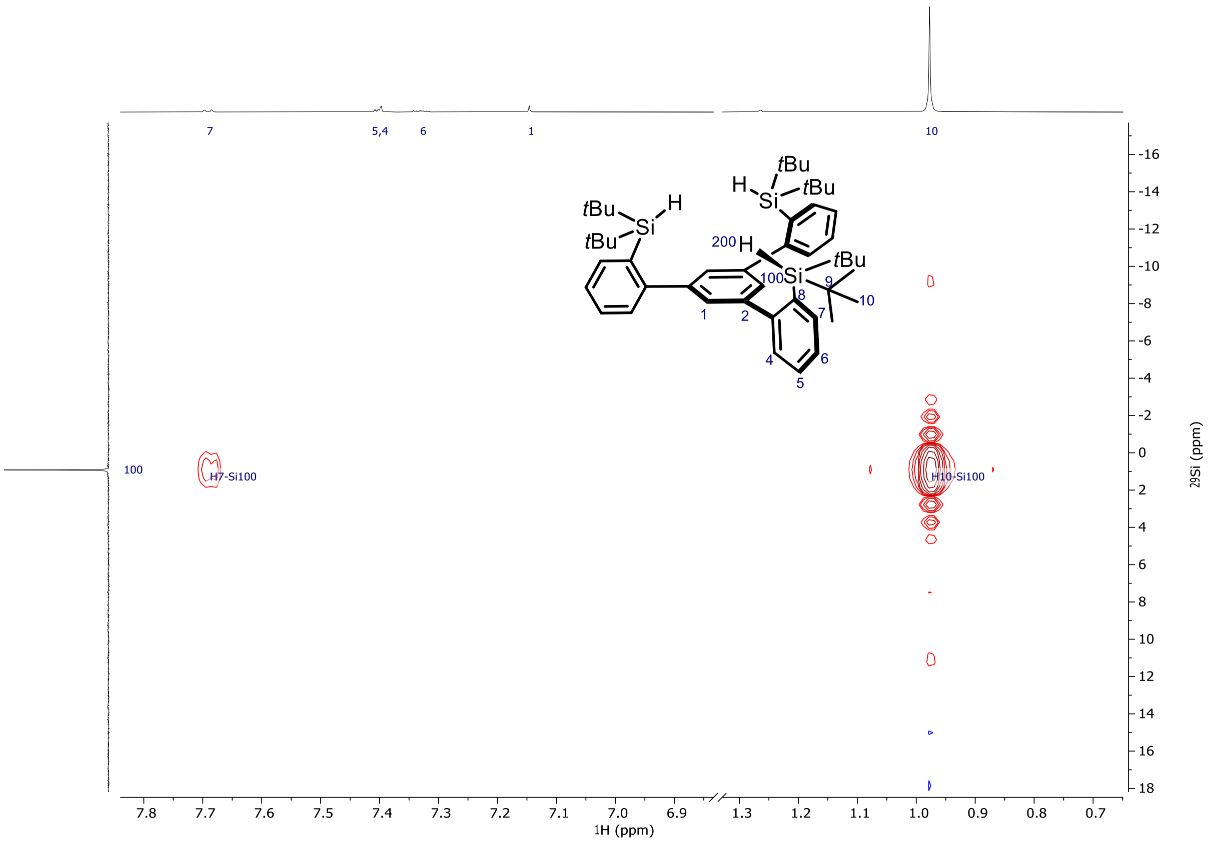

NMR analysis of ligand 7e: At lower temperature, ligand **7e** is a mixture of a C_3 symmetric and a C_S symmetric rotamer (see ^{29}Si NMR spectrum at 233 K). However, at higher temperature (353 K) the rotamers are averaged on the NMR timescale and a simpler NMR dataset was obtained. Therefore the assignments are based on the spectra recorded at 353 K.

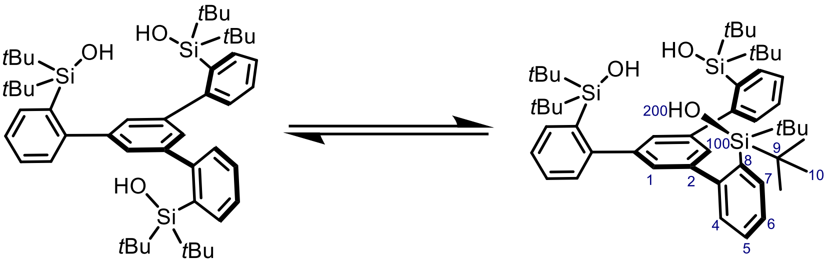

^{1}H NMR spectrum of ligand 7e: [D$_8$]-toluene, 353 K, 600 MHz.

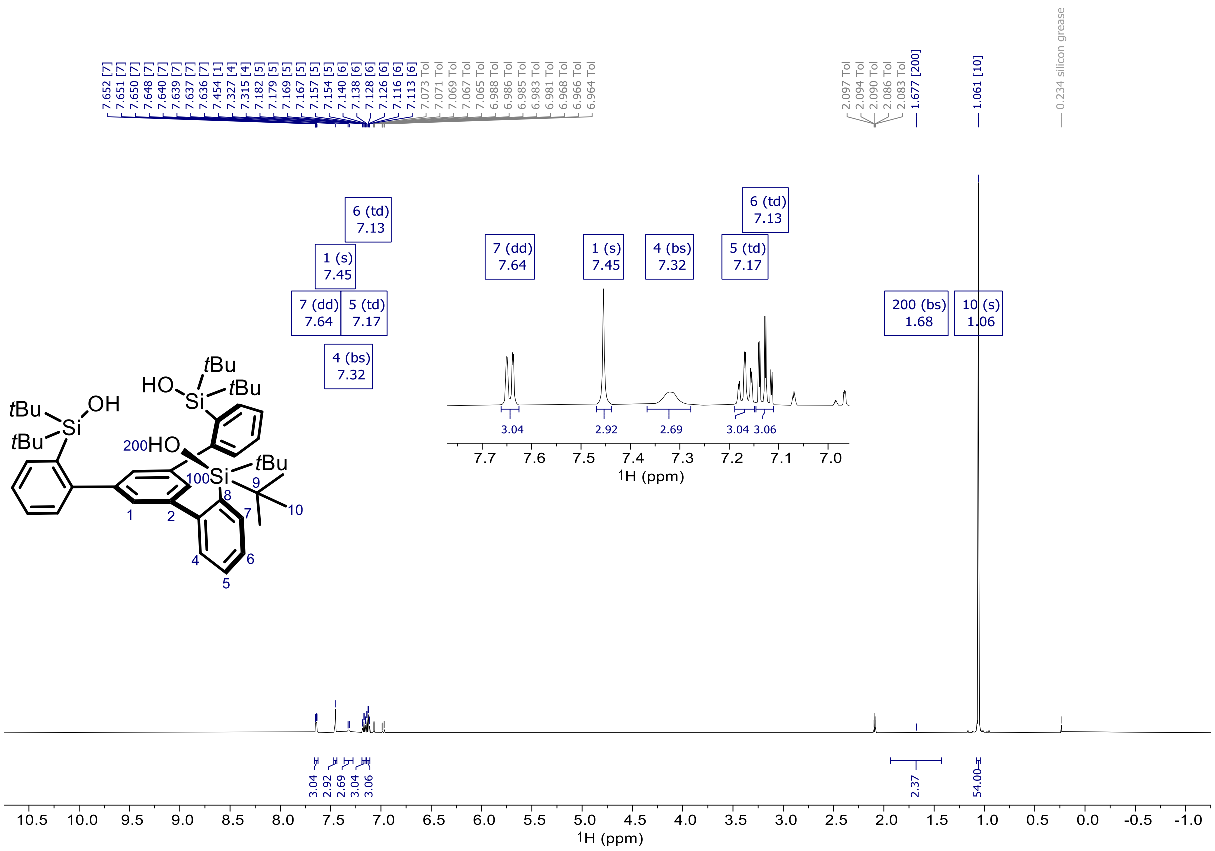

^{13}C NMR spectrum of ligand 7e: [D$_8$]-toluene, 353 K, 151 MHz.

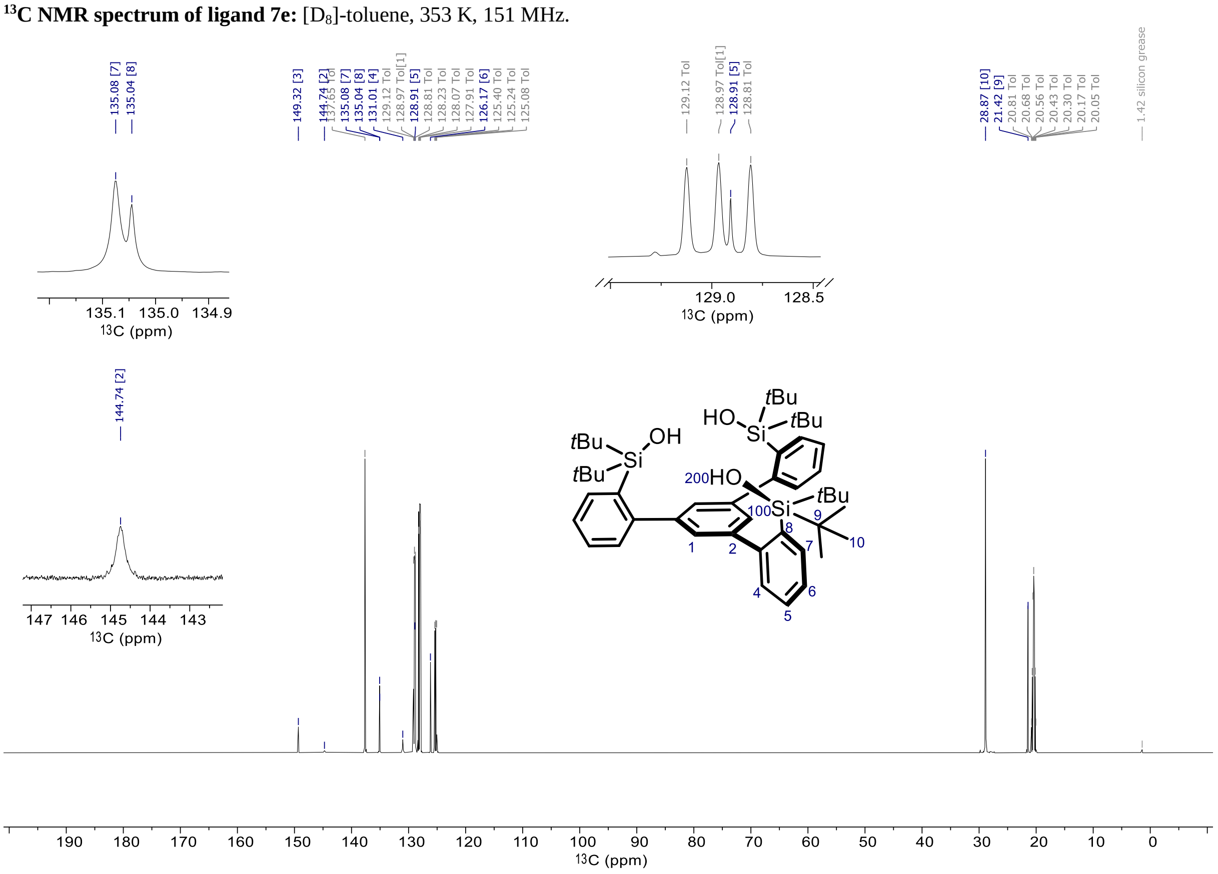

^{1}H-^{13}C HSQC NMR spectrum of ligand 7e: [D$_8$]-toluene, 353 K, 600 MHz, 151 MHz.

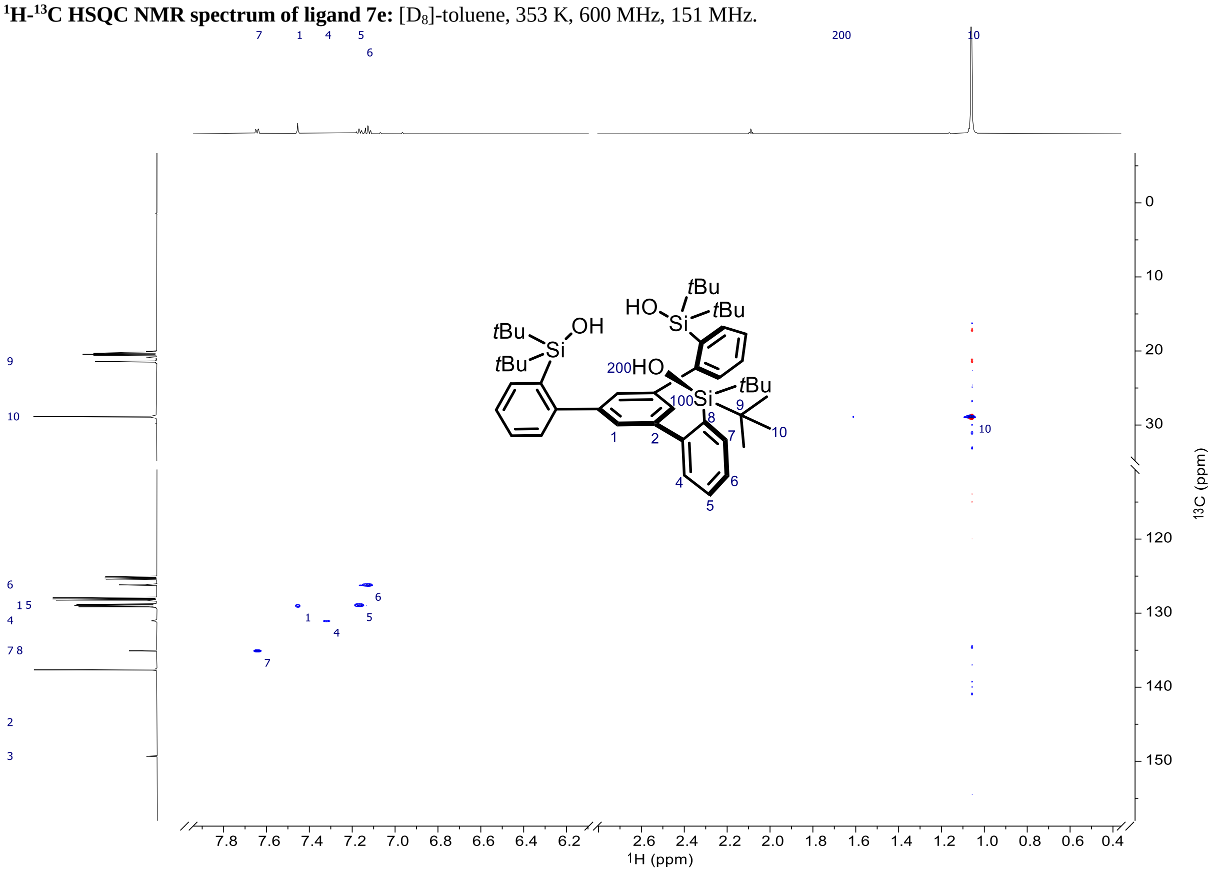

^{1}H-^{13}C HMBC NMR spectrum of ligand 7e: [D_8]-toluene, 353 K, 600 MHz, 151 MHz.

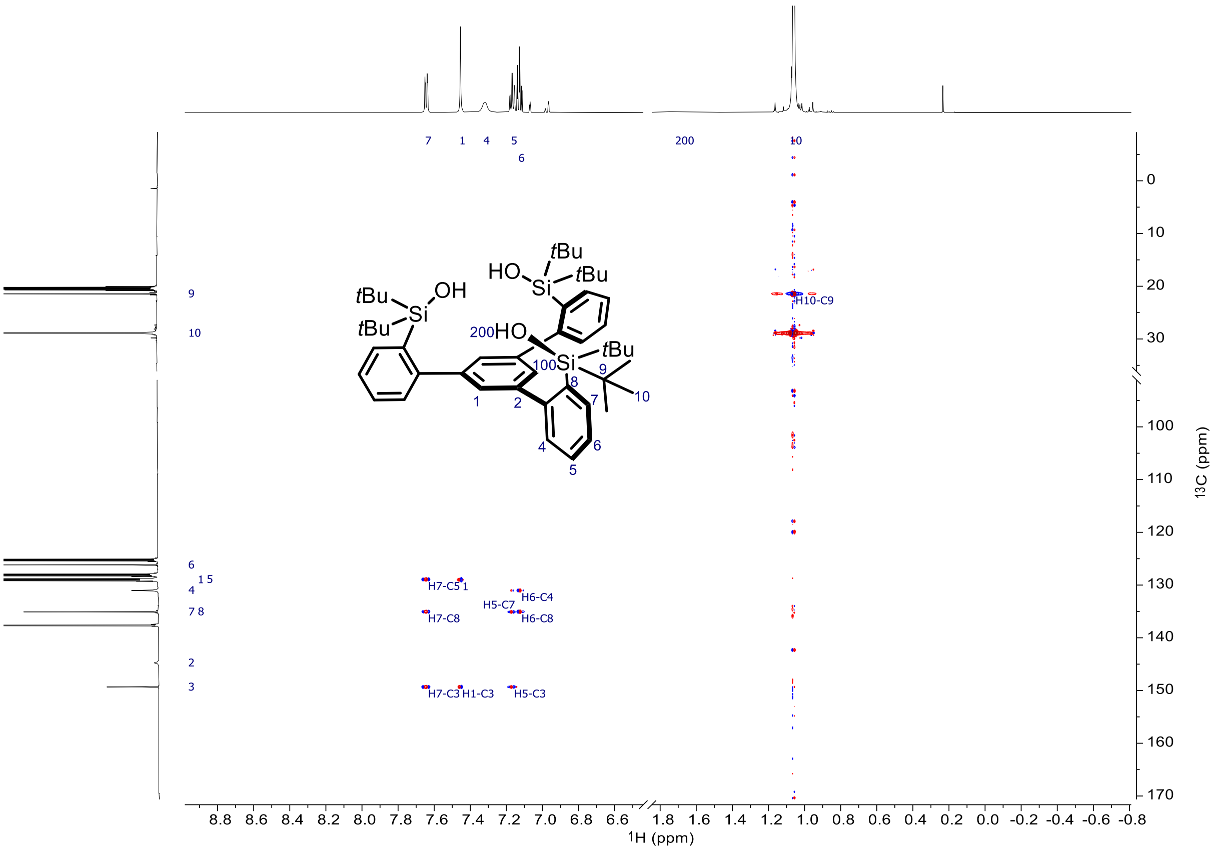

^{1}H-^{1}H COSY NMR spectrum of ligand 7e: [D_8]-toluene, 353 K, 600 MHz, 600 MHz.

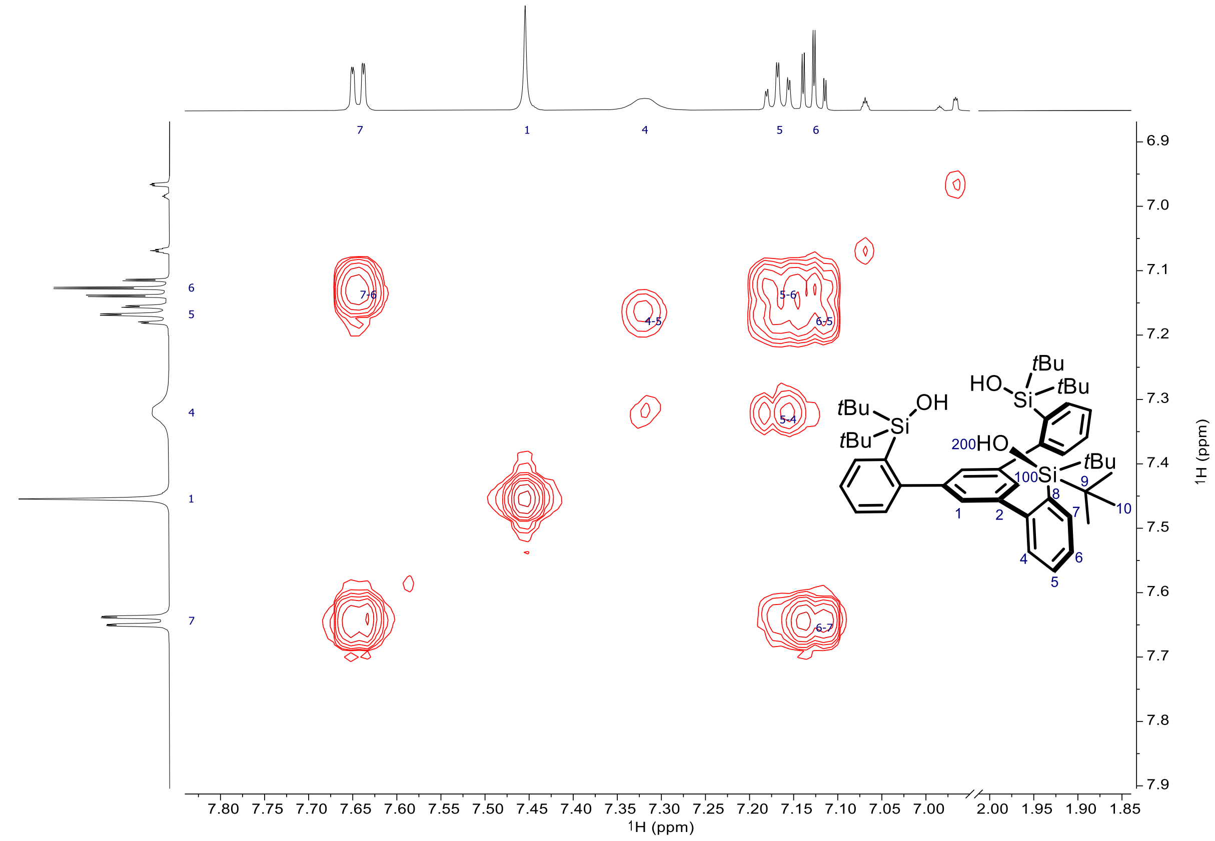

^{13}C DEPT-135 NMR spectrum of ligand 7e: [D$_8$]-toluene, 353 K, 151 MHz.

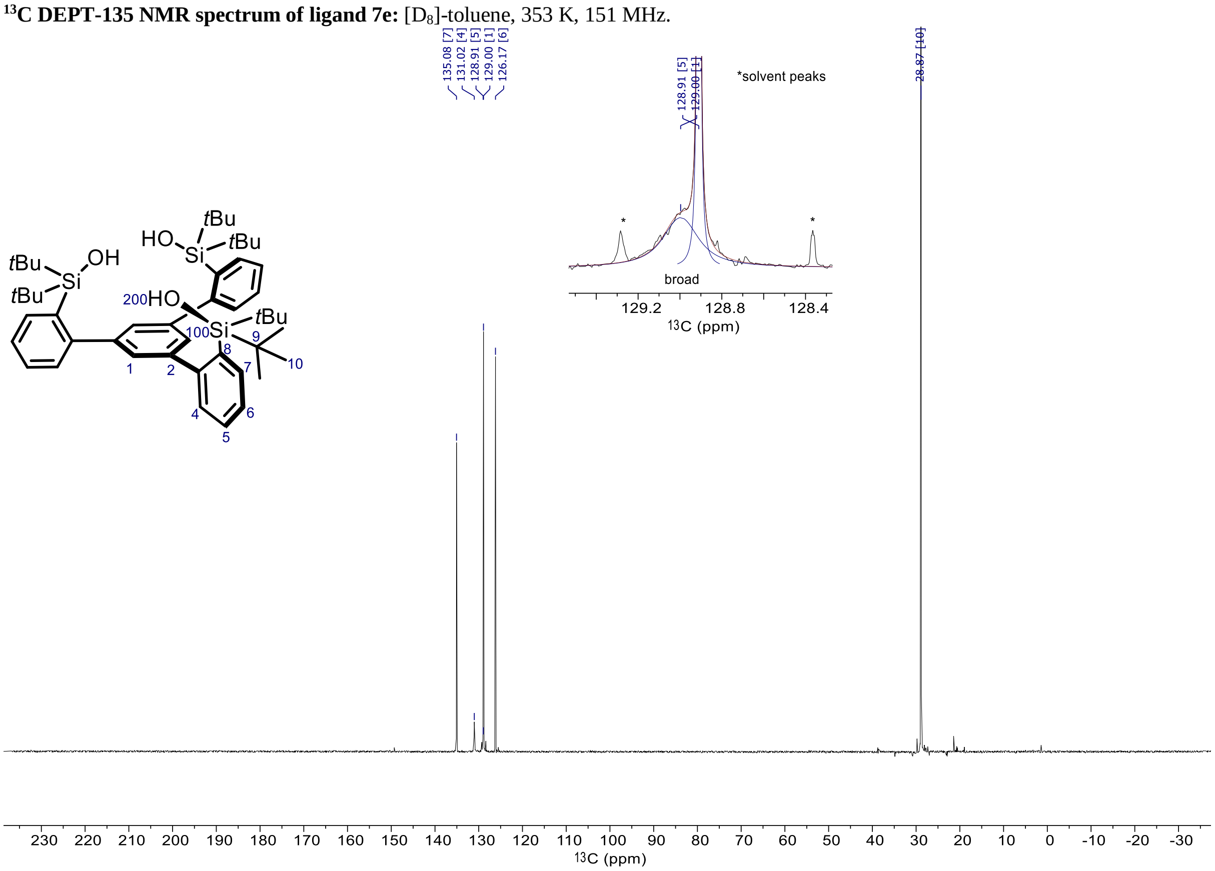

^{29}Si NMR spectrum of ligand 7e: [D$_8$]-toluene, 353 K, 119 MHz.

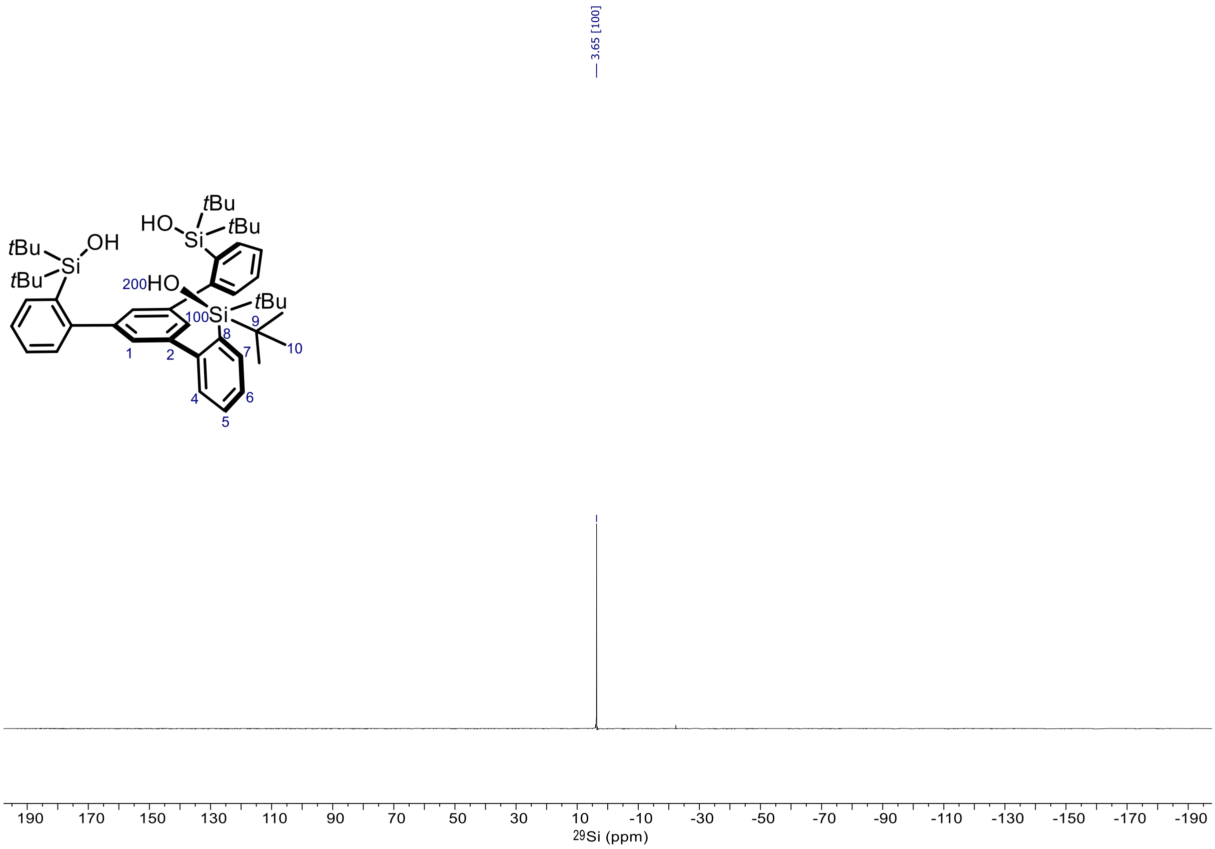

^{29}Si NMR spectrum of ligand 7e: [D$_8$]-toluene, 233 K, 119 MHz.

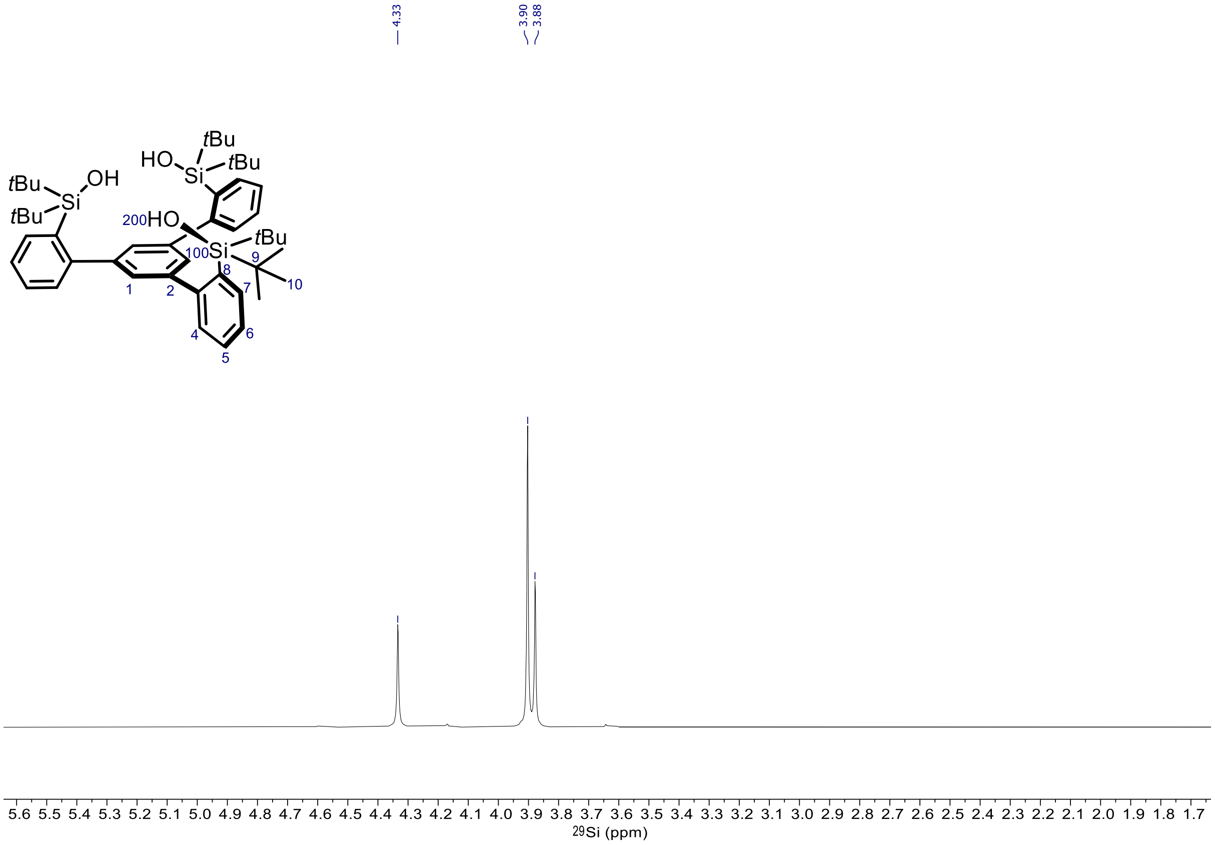

^{1}H-^{29}Si HMBC NMR spectrum of ligand 7e: [D$_8$]-toluene, 353 K, 600 MHz, 119 MHz.

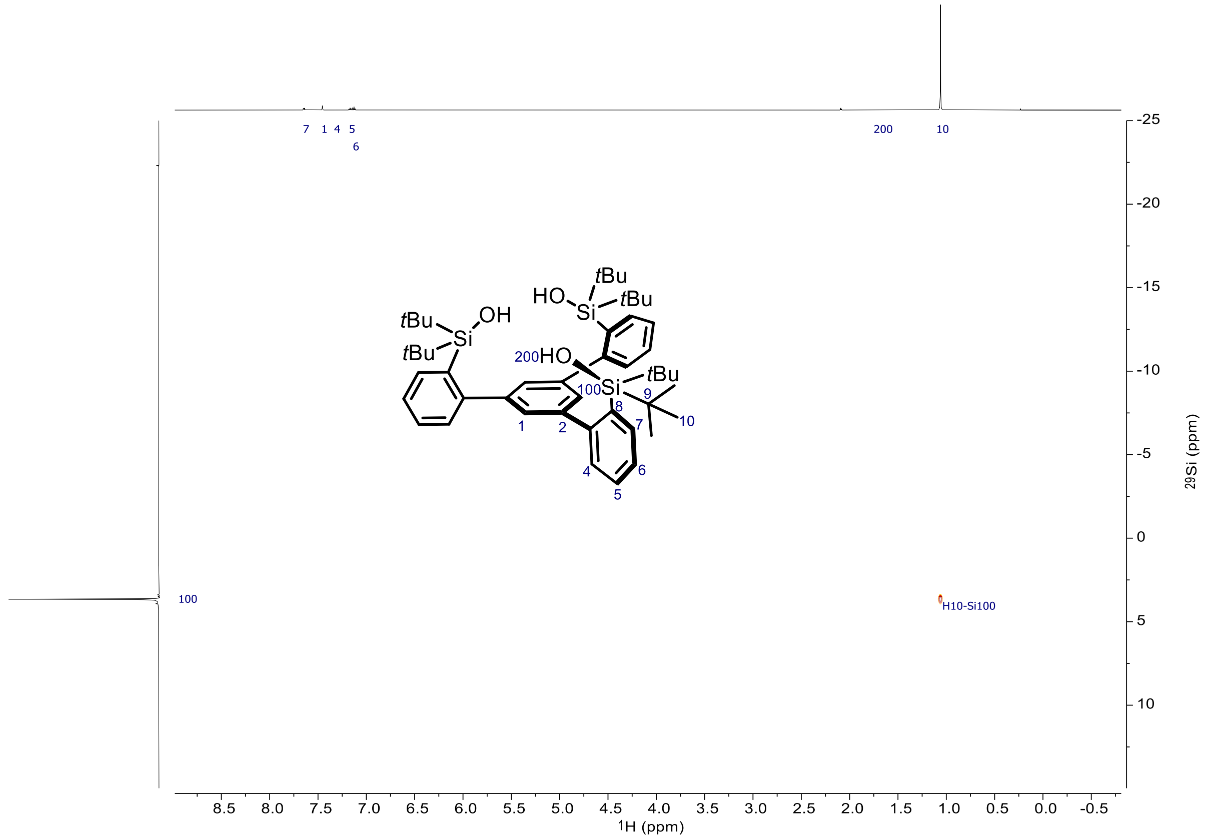

Temperature-dependence of the ^{1}H NMR spectra of ligand 7e: [D_8]-toluene, 233 K−353 K, 600 MHz.

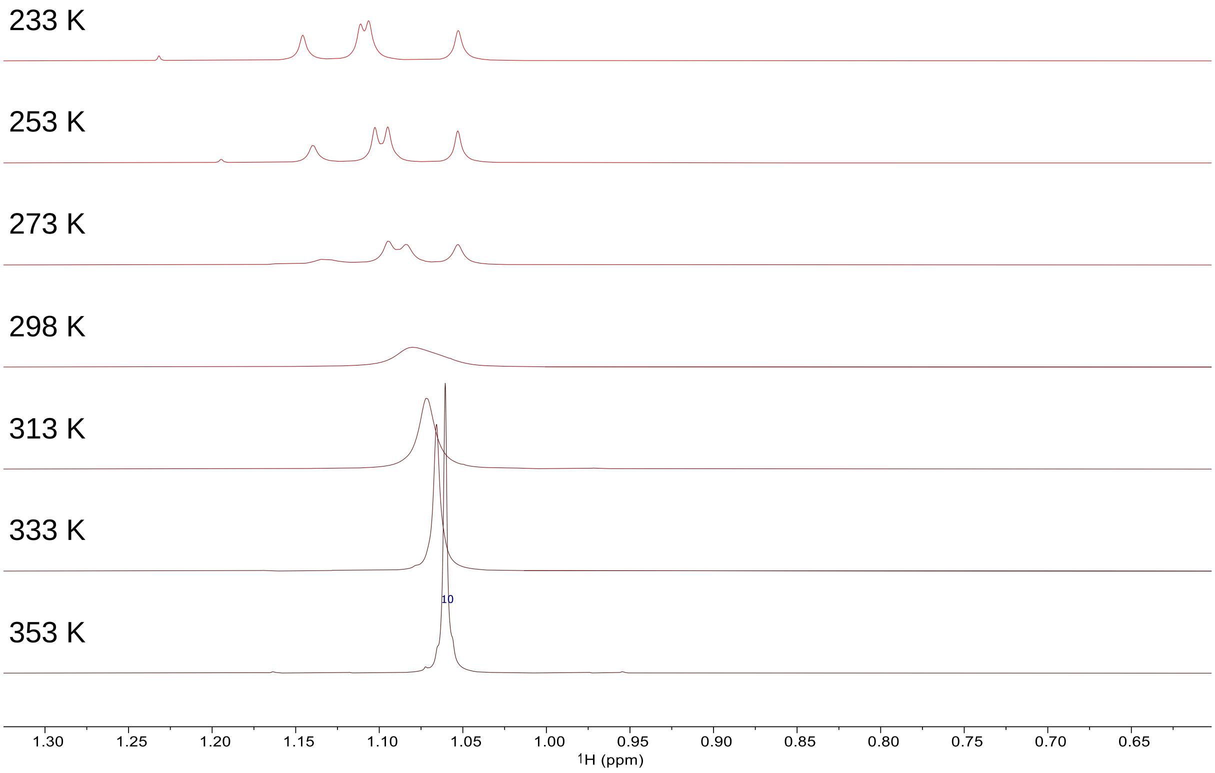

NMR analysis of complex 3a: ^{1}H NMR signals of complex **3a** were broad at 298 K. The complex was characterized at 333 K where all the signals were sharper (see VT NMR data). The analysis indicates that the complex is C_3 symmetric at this temperature. The ^{95}Mo NMR shift was extracted after peak fitting and measured at 298 K.

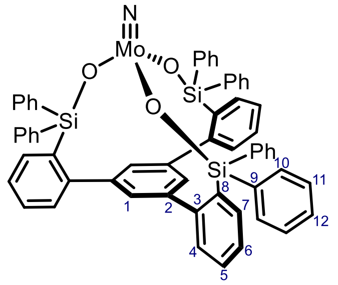

^{1}H NMR spectrum of complex 3a: [D$_6$]-benzene, 333 K, 600 MHz.

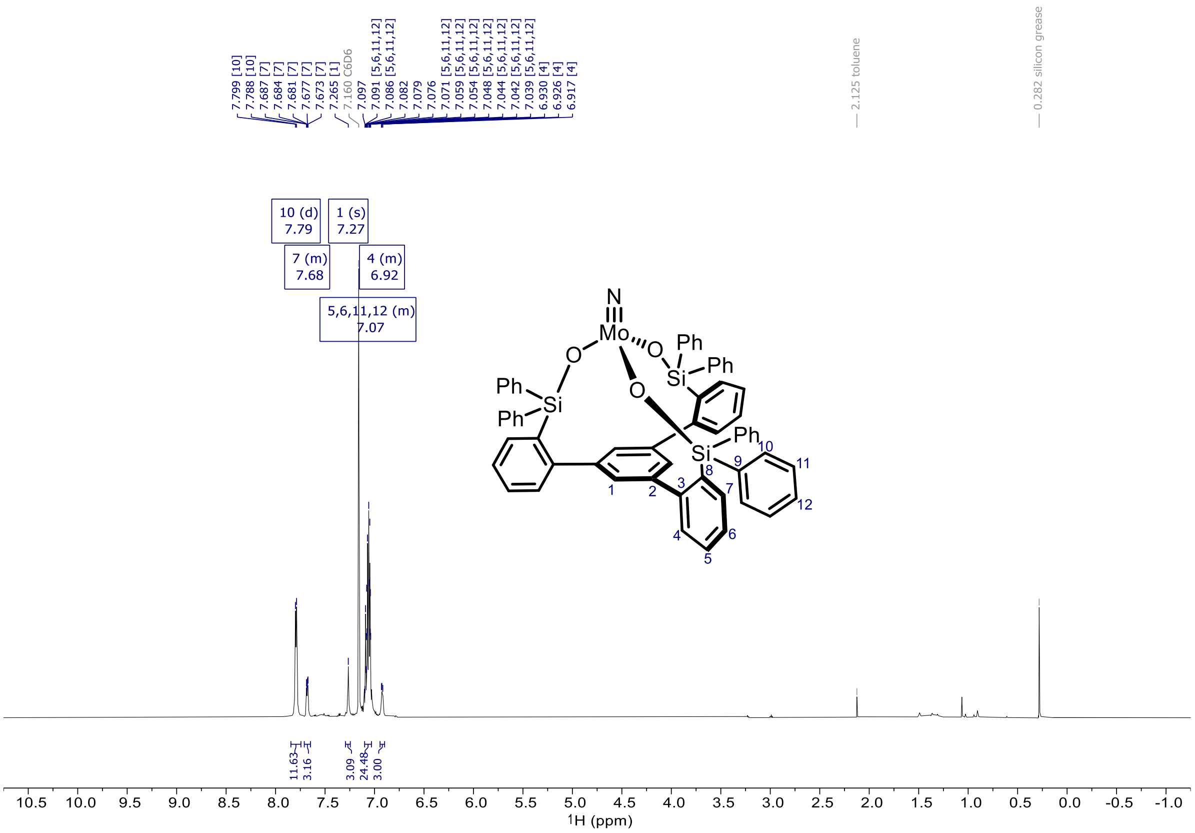

Temperature-dependence of the ^{1}H NMR spectra of complex 3a: [D$_6$]-benzene, 298 K−323 K, 600 MHz.

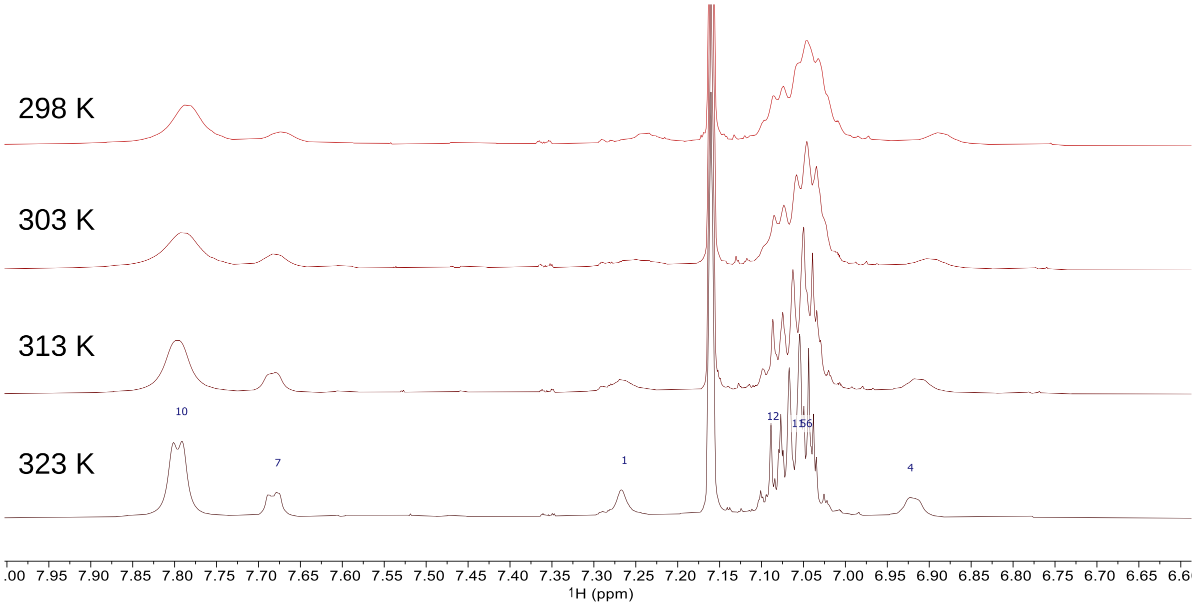

^{13}C NMR spectrum of complex 3a: [D$_6$]-benzene, 333 K, 151 MHz.

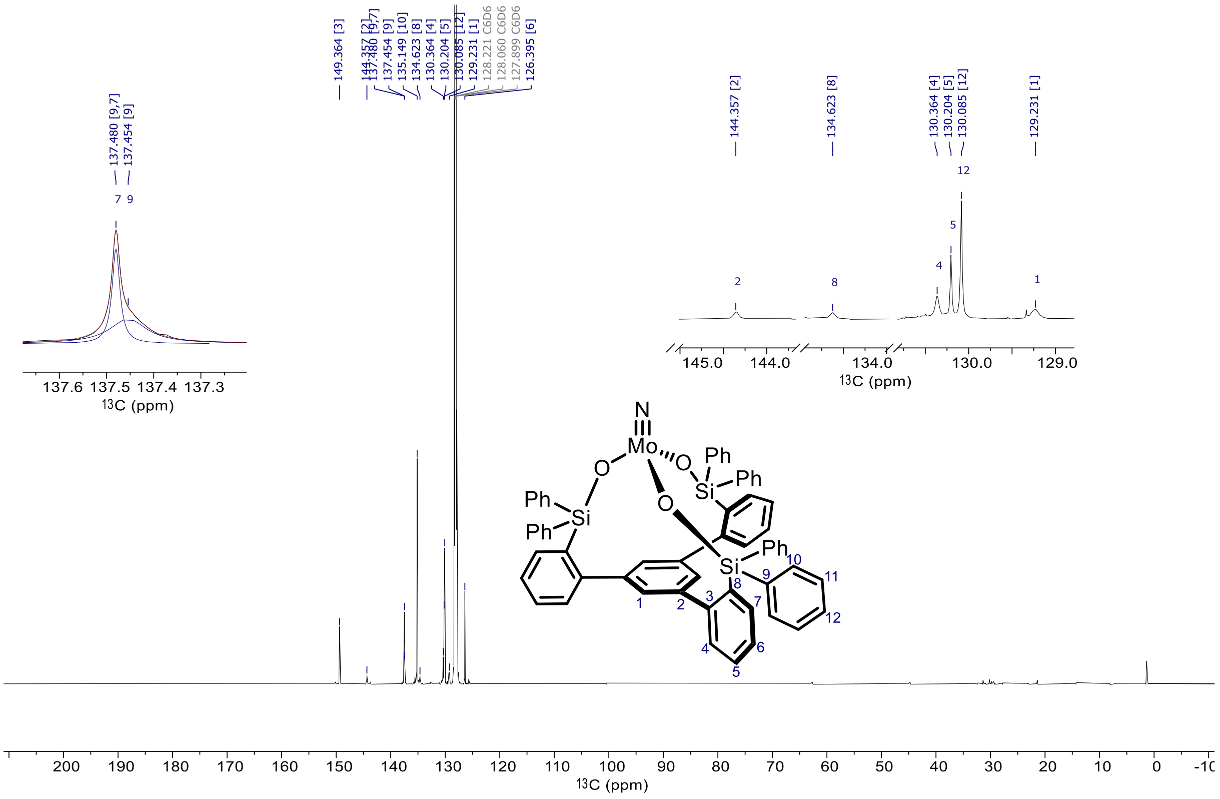

^{1}H-^{13}C HSQC NMR spectrum of complex 3a: [D$_6$]-benzene, 333 K, 600 MHz, 151 MHz.

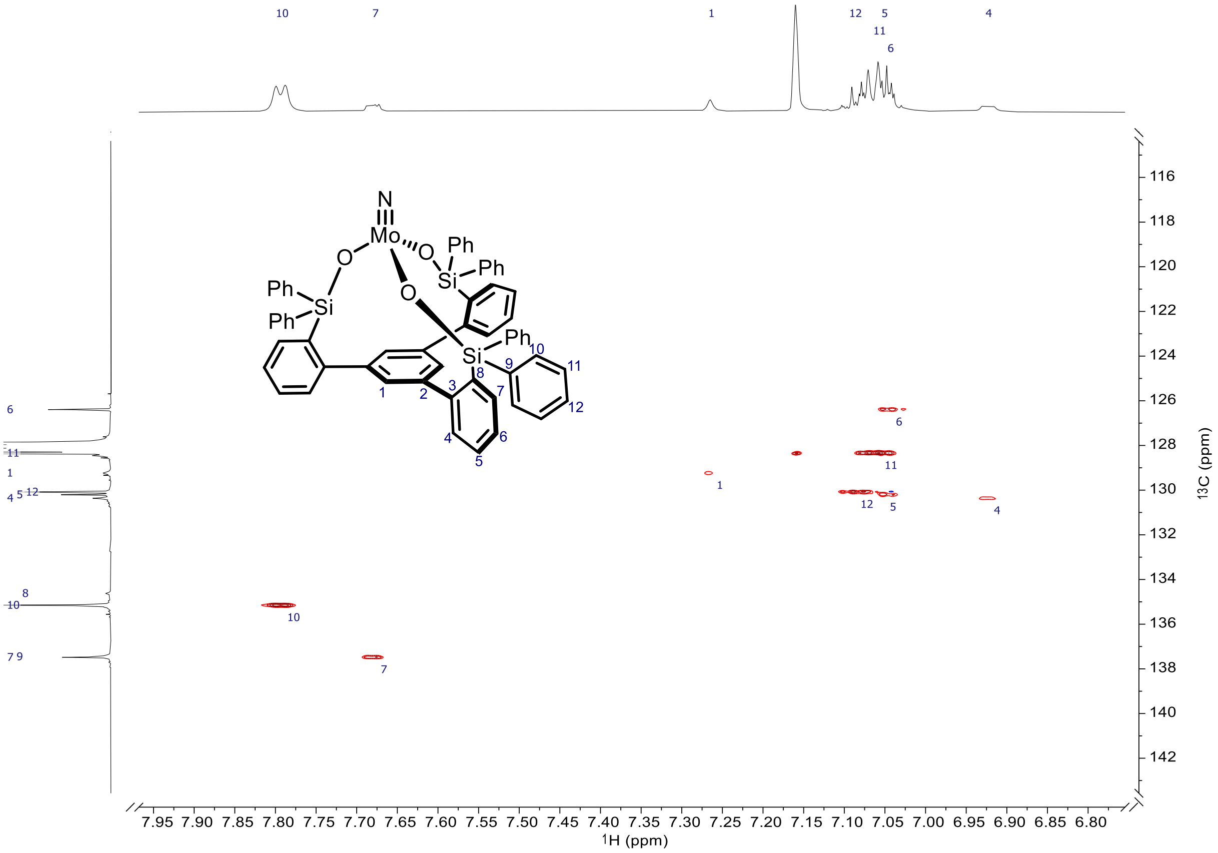

^{1}H-^{13}C HMBC NMR spectrum of complex 3a: [D$_6$]-benzene, 333 K, 600 MHz, 151 MHz.

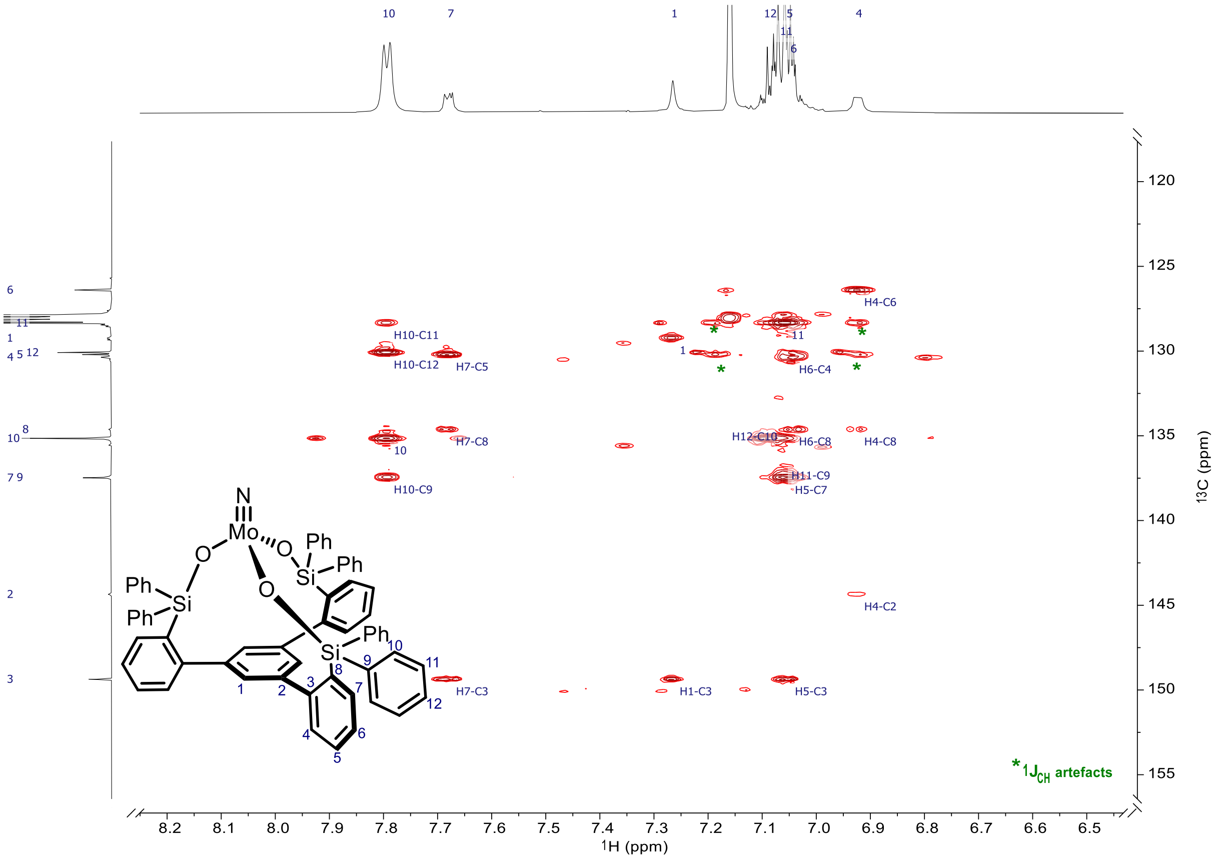

^{1}H-^{1}H COSY NMR spectrum of complex 3a: [D$_6$]-benzene, 333 K, 600 MHz.

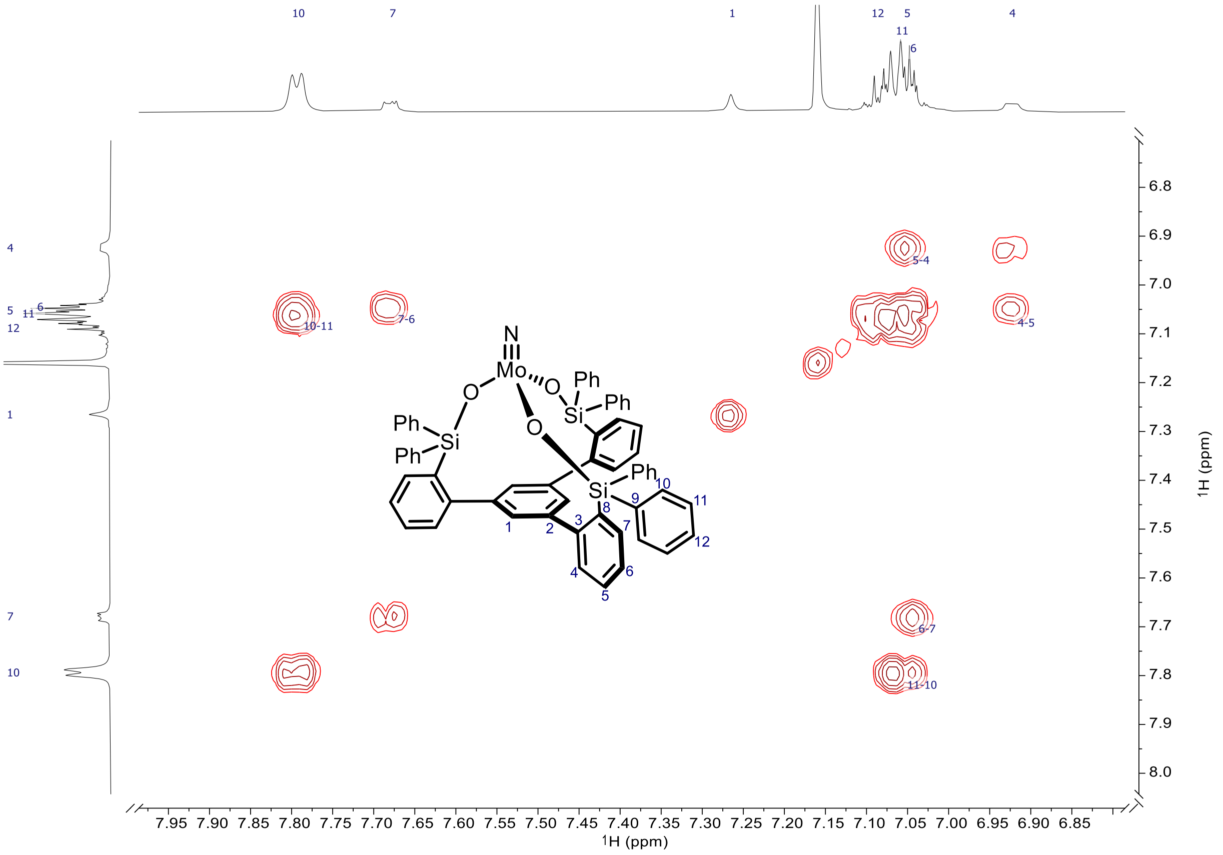

^{13}C DEPT-135 NMR spectrum of complex 3a: [D$_6$]-benzene, 333 K, 151 MHz.

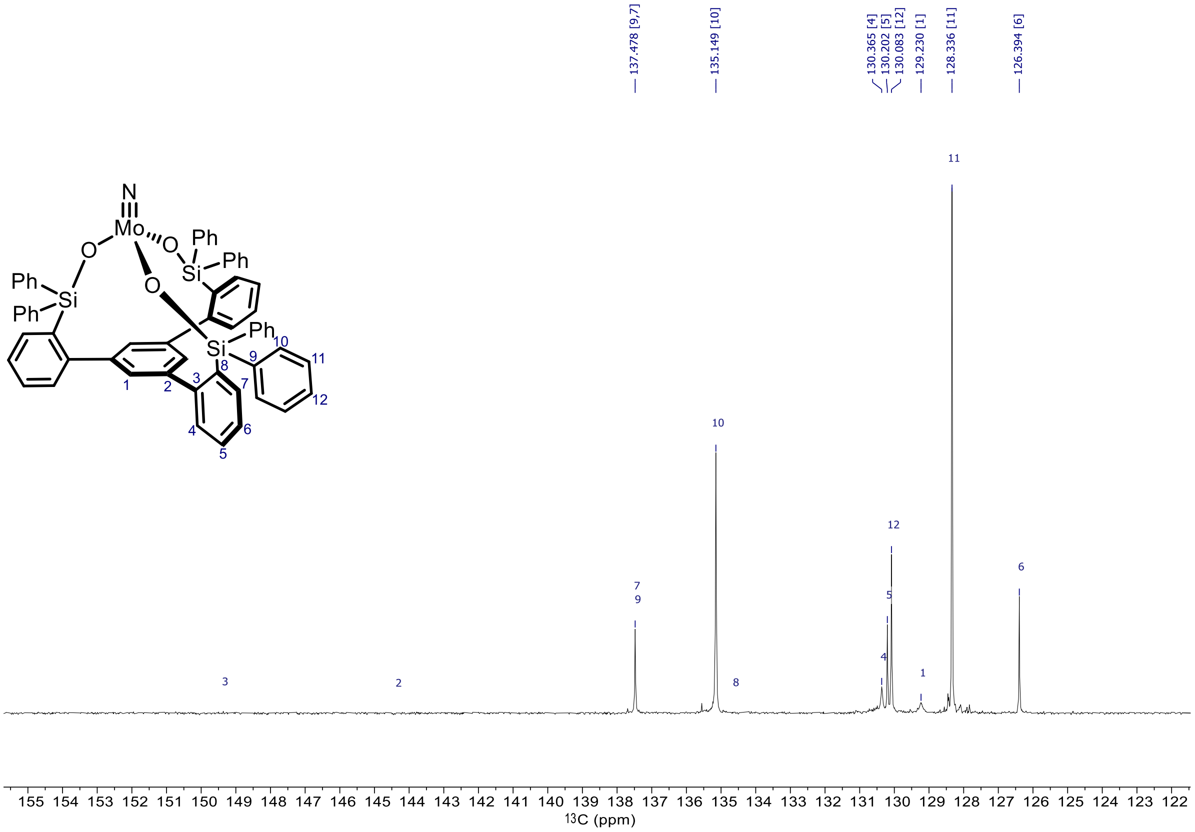

^{95}Mo NMR spectrum of complex 3a: [D$_6$]-benzene, 298 K, 26.1 MHz

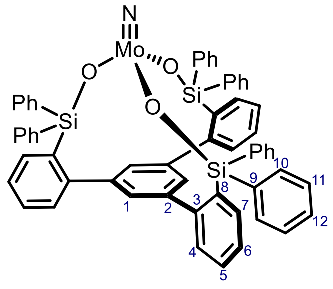

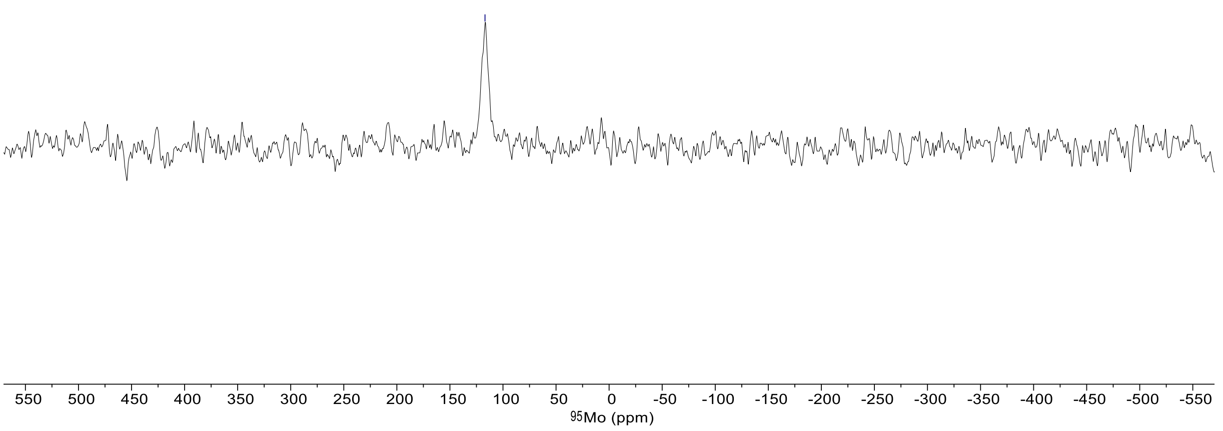

NMR analysis of complex 3b: The NMR signals of this complex are broadened at 298 K. However, NMR analysis at 298 K indicates C_3 symmetry. Therefore, the assignments of the peaks are based on the spectra recorded at 298 K. ^{95}Mo NMR data was measured 298 K and the shift was extracted after peak fitting.

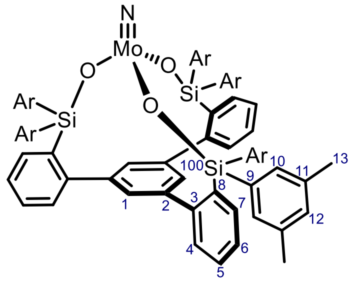

^{1}H NMR spectrum of complex 3b: [D$_6$]-benzene, 298 K, 600 MHz.

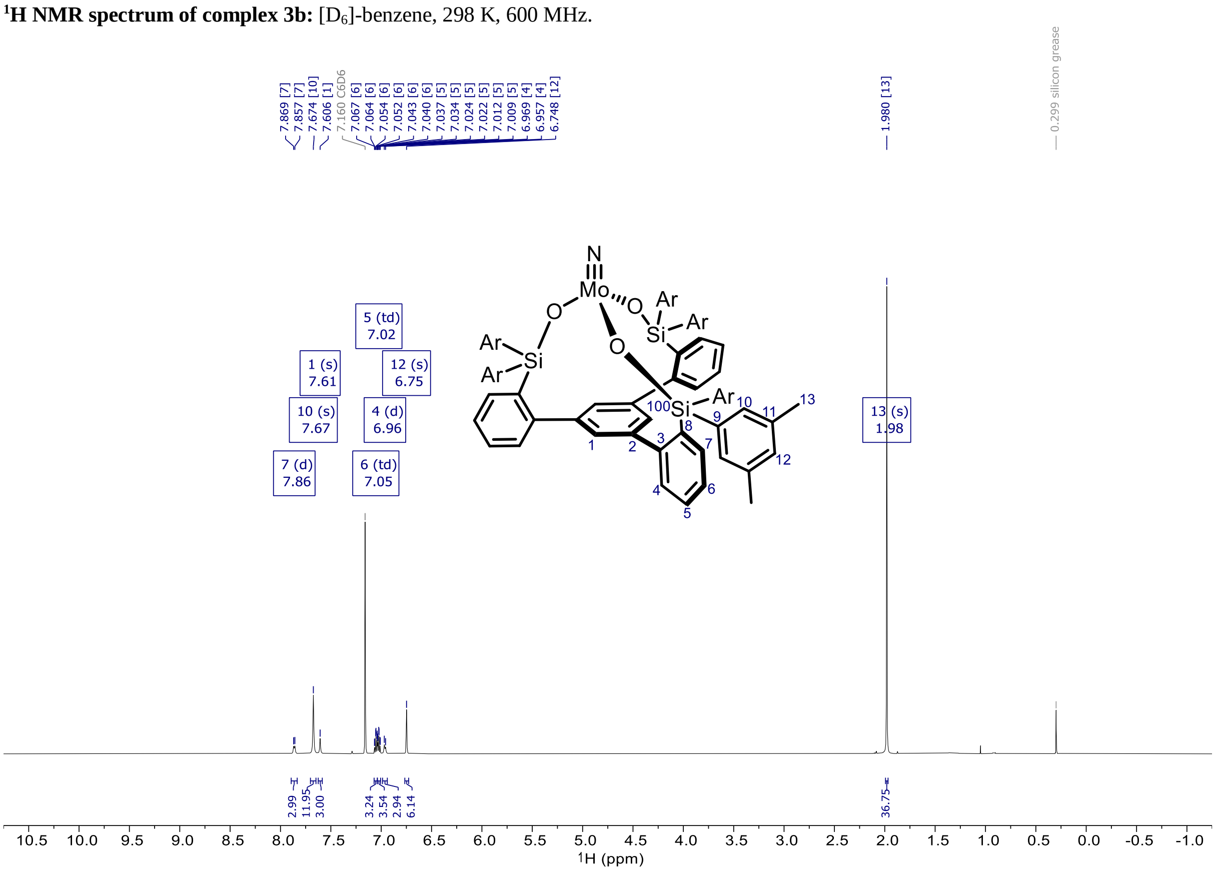

^{13}C NMR spectrum of complex 3b: [D$_6$]-benzene, 298 K, 151 MHz.

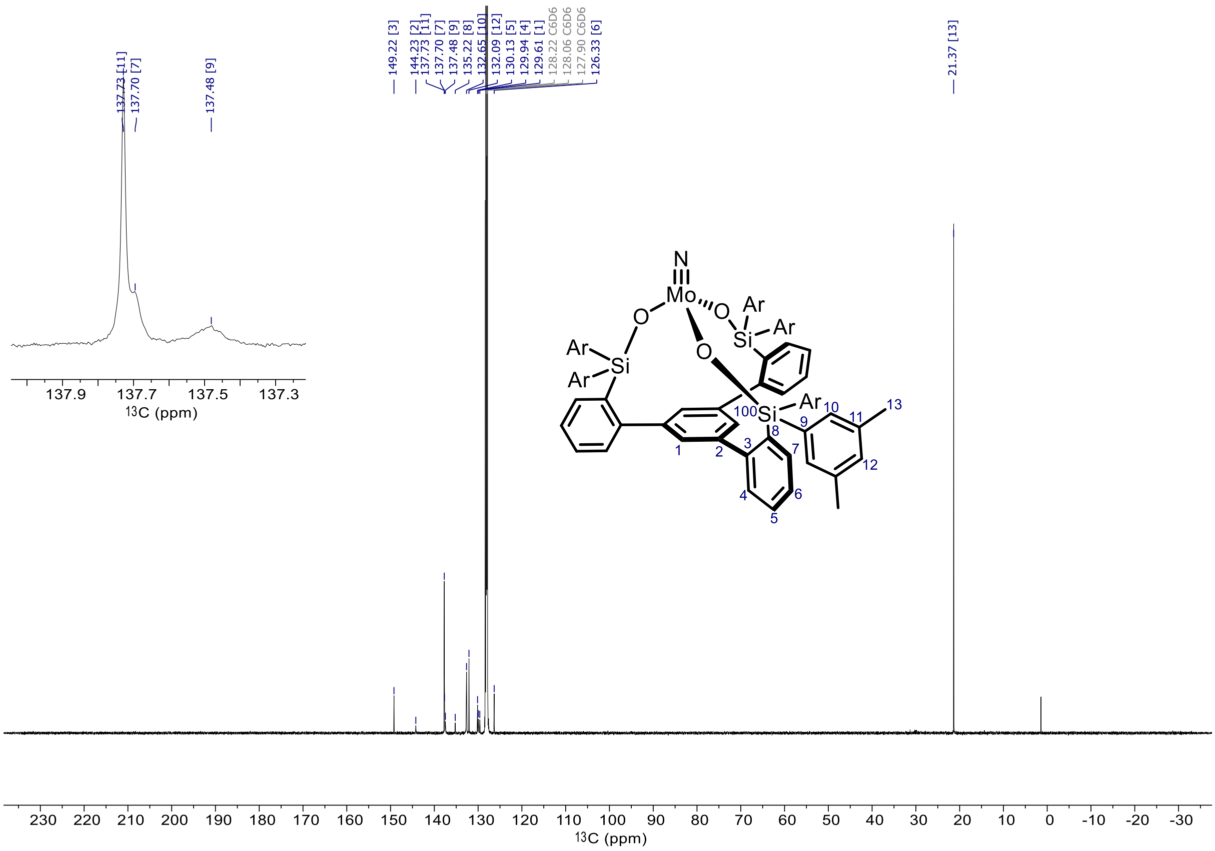

^{1}H-^{13}C HSQC NMR spectrum of complex 3b: [D$_6$]-benzene, 298 K, 600 MHz, 151 MHz.

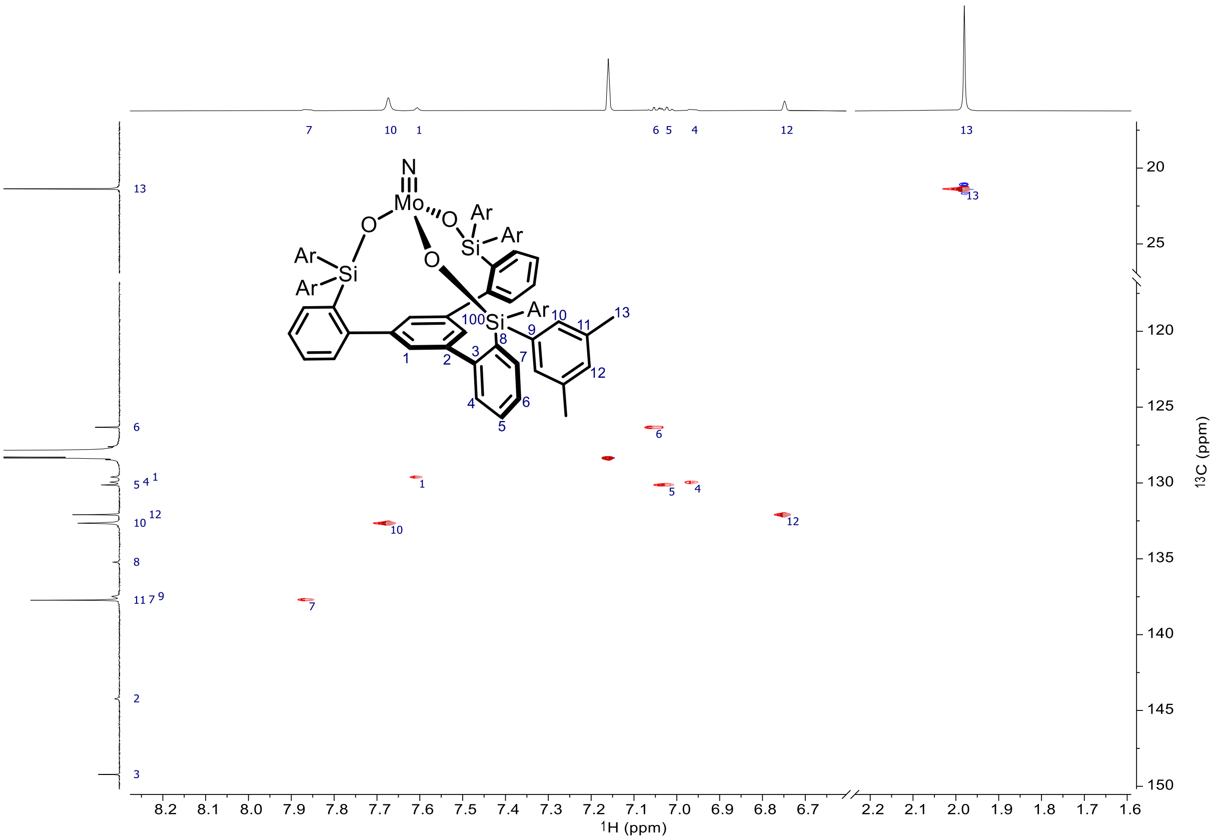

^{1}H-^{13}C HMBC NMR spectrum of complex 3b: [D_6]-benzene, 298 K, 600 MHz, 151 MHz.

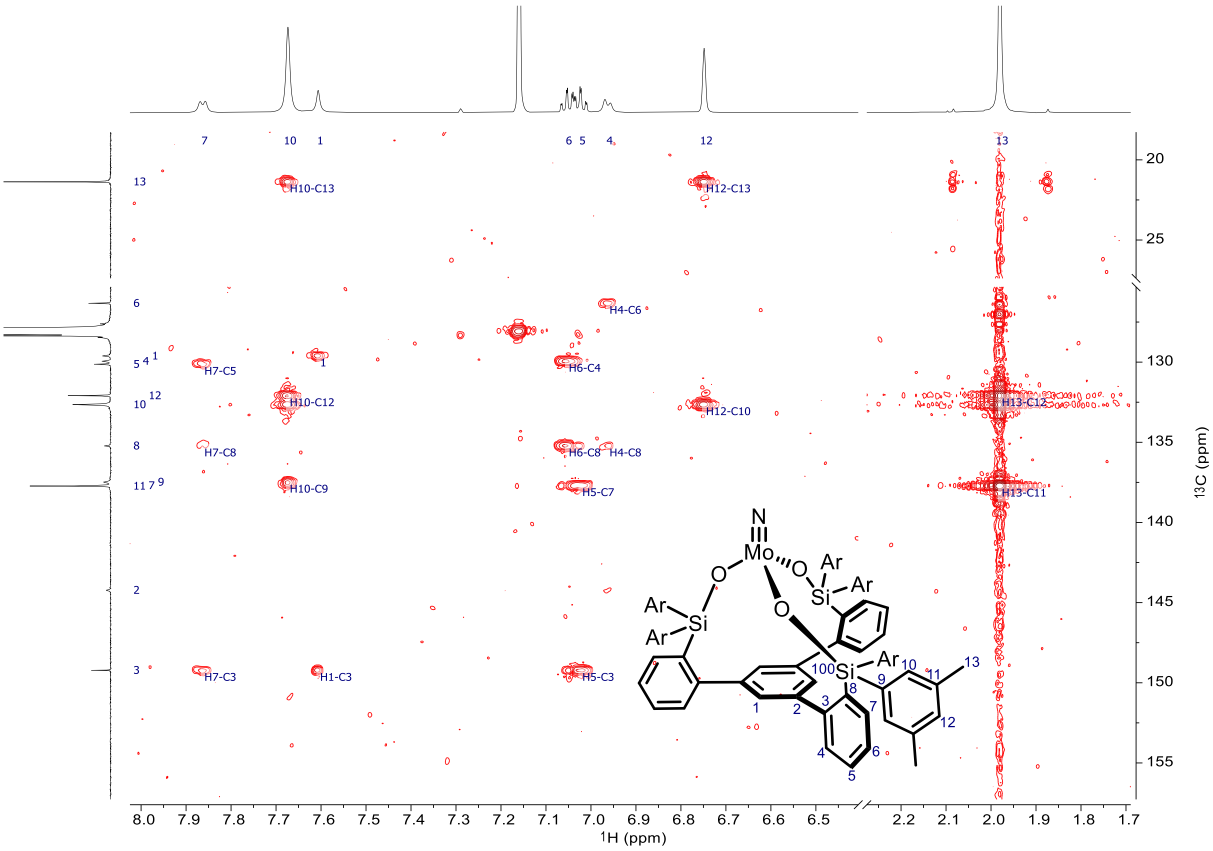

^{1}H-^{1}H COSY NMR spectrum of complex 3b: [D_6]-benzene, 298 K, 600 MHz, 600 MHz.

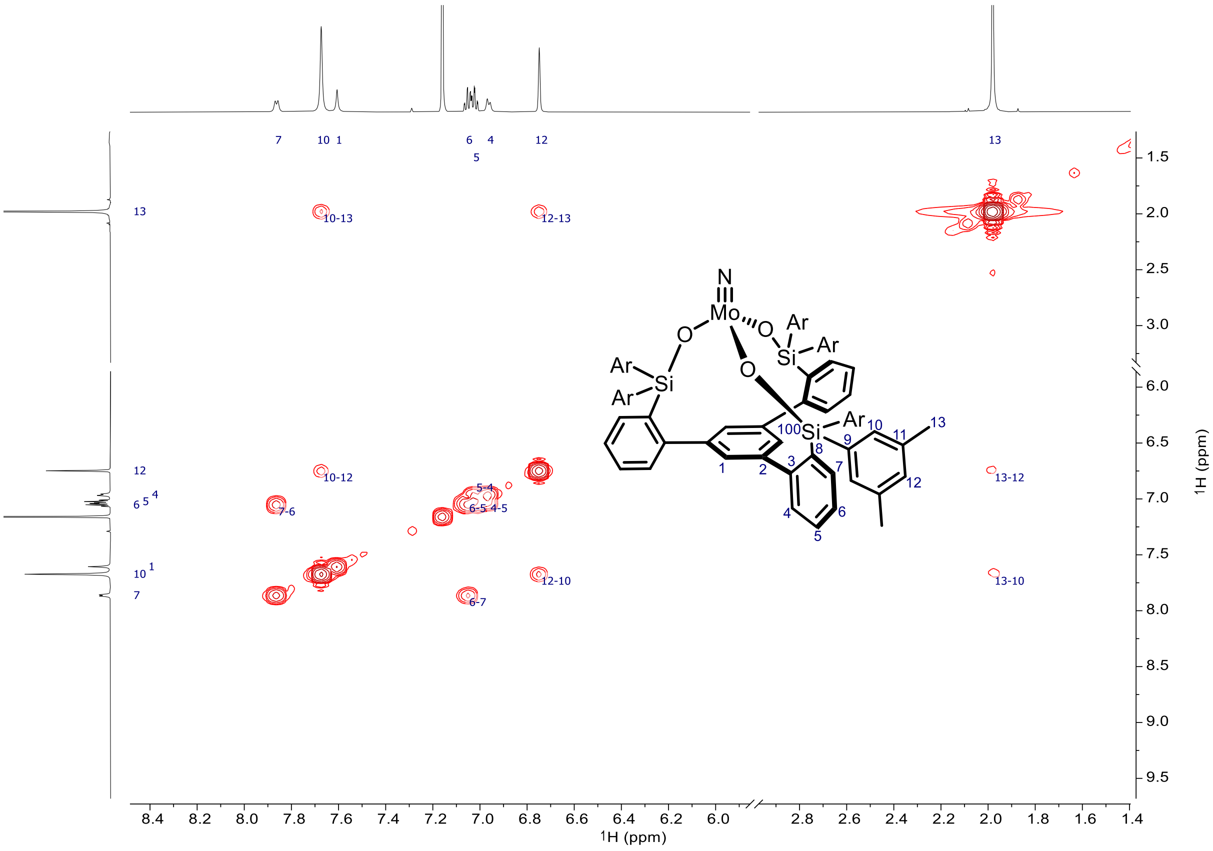

^{29}Si NMR spectrum of complex 3b: [D$_6$]-benzene, 298 K, 119 MHz and **^{1}H-^{29}Si HMBC NMR:** [D$_6$]-benzene, 298 K, 600 MHz, 119 MHz.

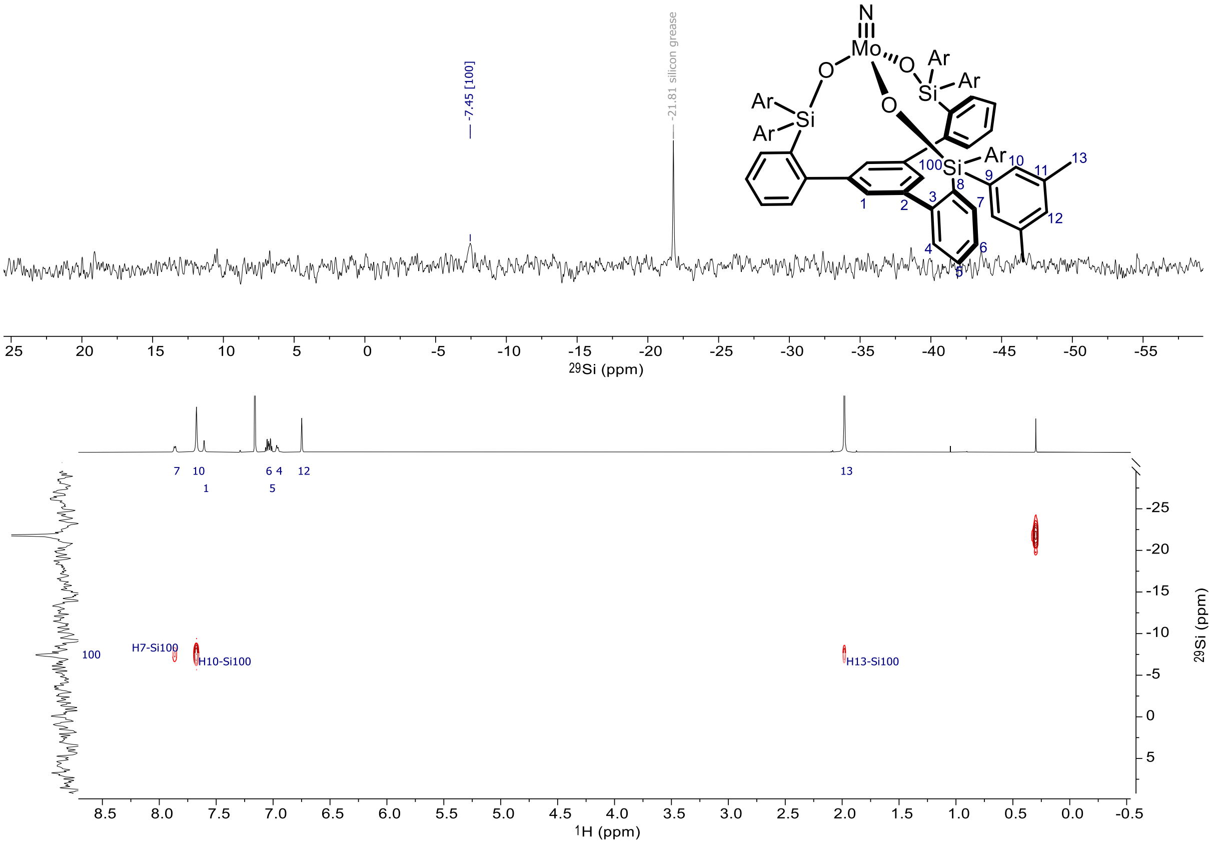

Temperature-dependence of the ^{1}H NMR spectrum of complex 3b: [D$_6$]-benzene, 298 K−323 K, 600 MHz.

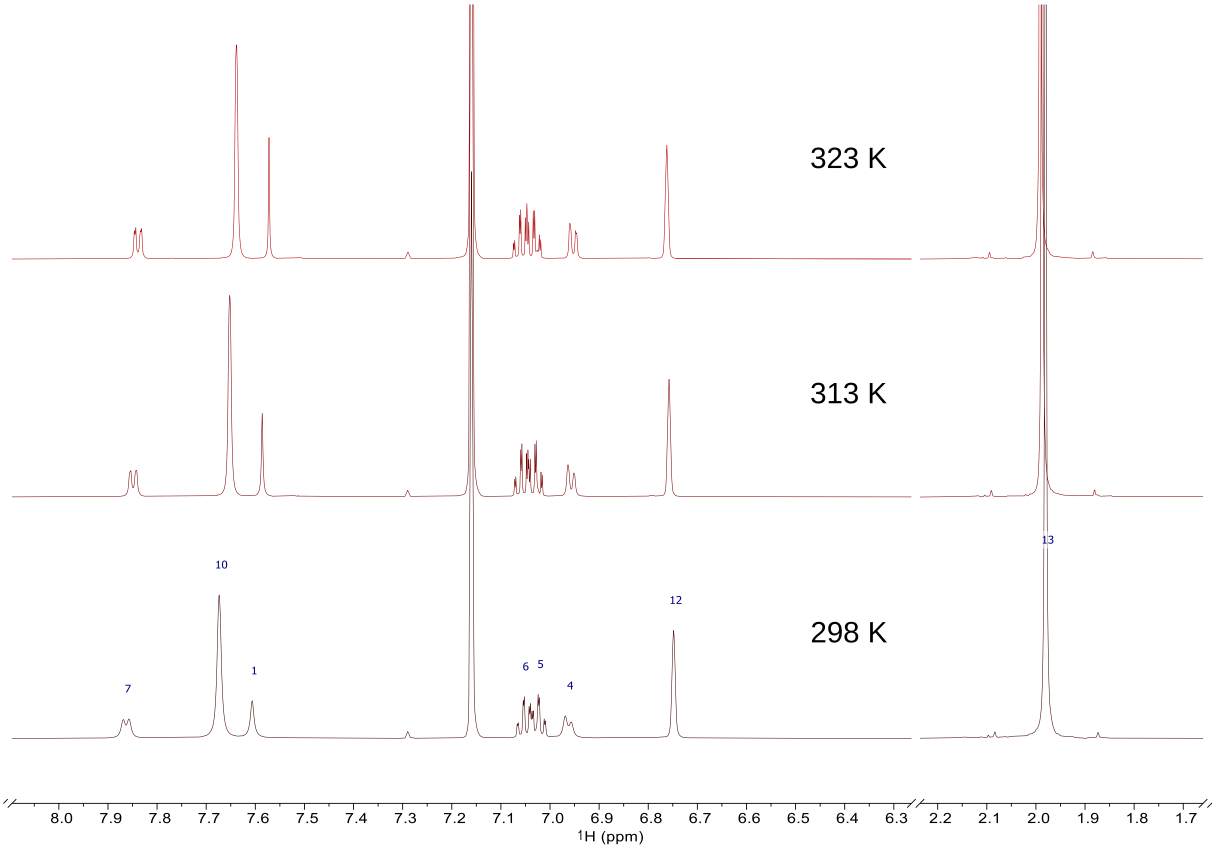

^{95}Mo NMR spectrum of complex 3b: [D$_6$]-benzene, 298 K, 26 MHz.

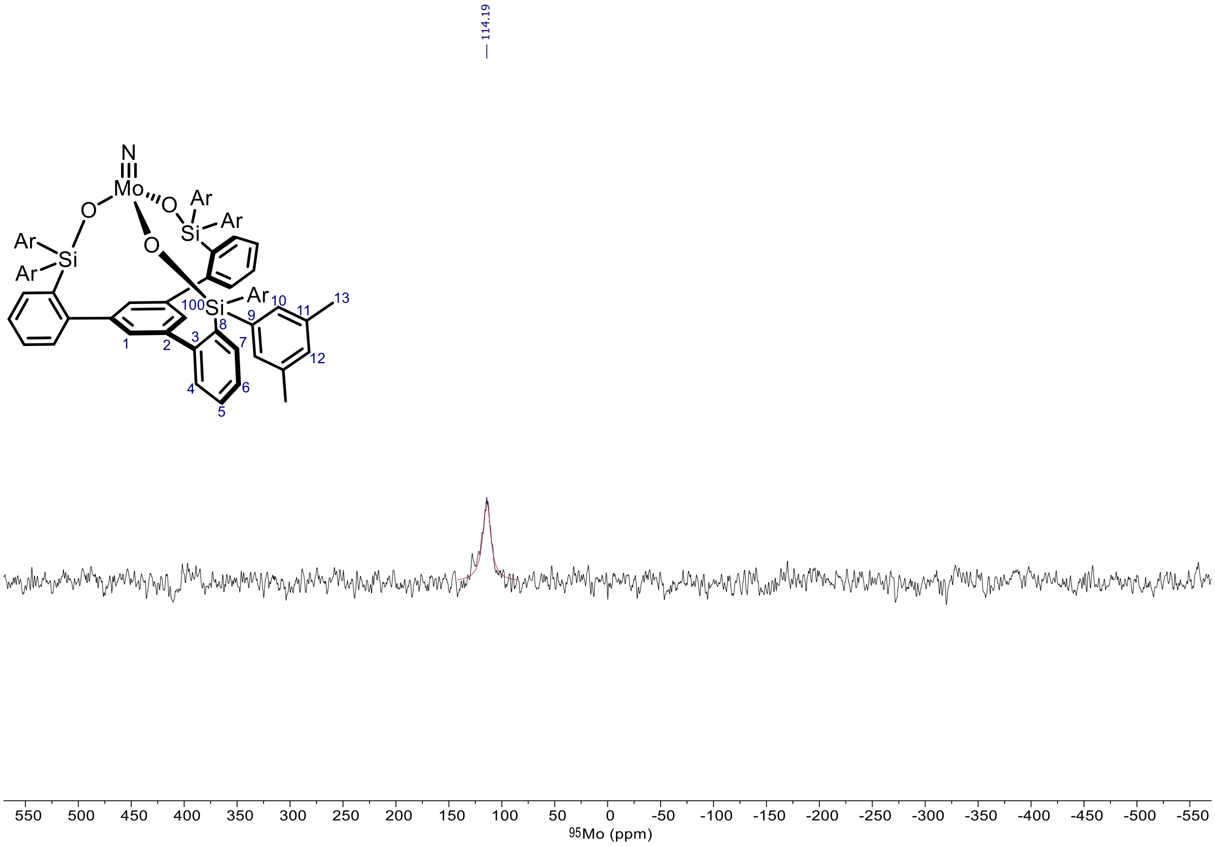

^{95}Mo NMR spectrum of complex 3b: [D$_8$]-toluene, 298 K, 26 MHz.

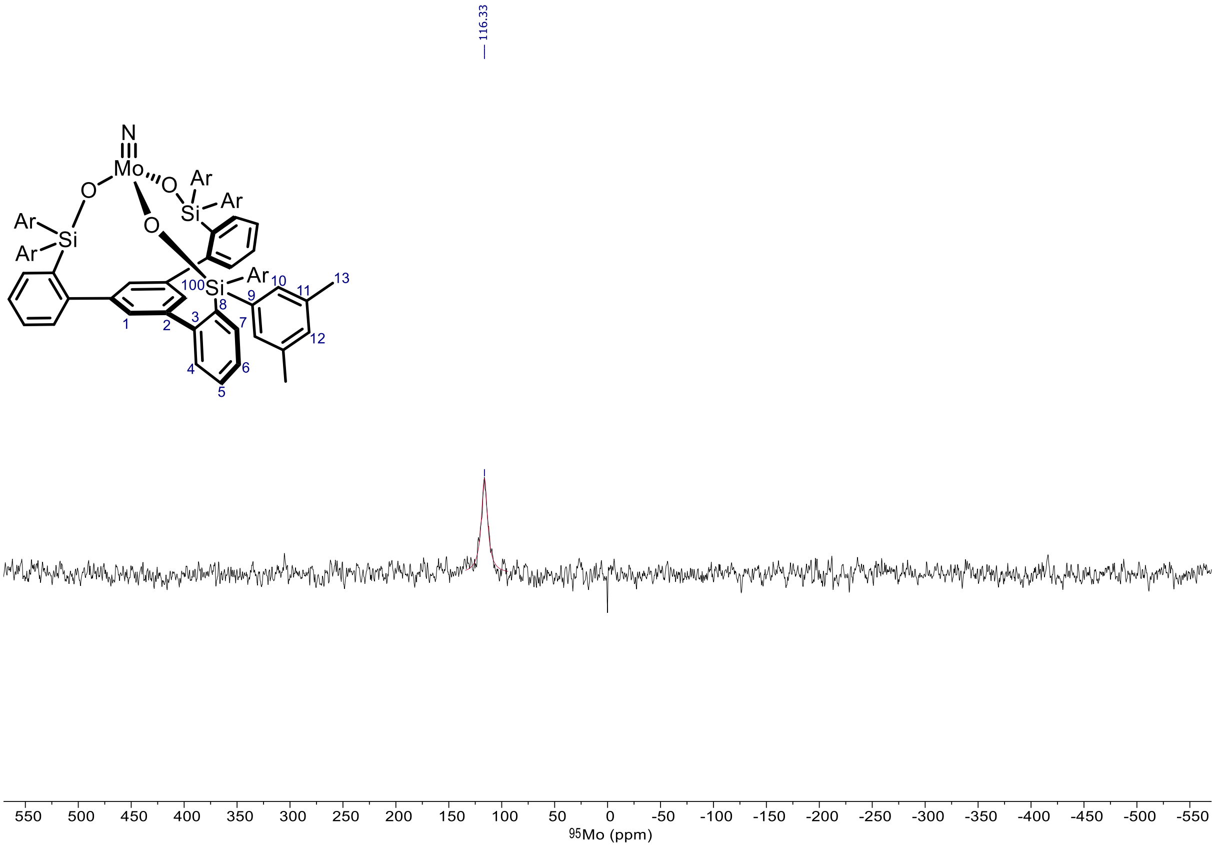

NMR analysis of complex 3c: The two protons at C9 are diastereotopic, but were picked as a multiplet as they are partially overlapping. The ^{95}Mo NMR data was measured at 298 K. The ^{14}N NMR shift was extracted after peak fitting and measured at 333 K to obtain shaper signals.

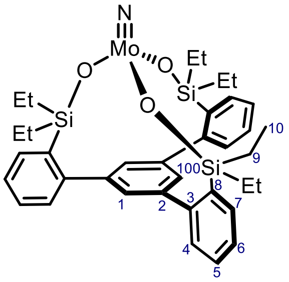

^{1}H NMR spectrum of complex 3c: [D$_8$]-toluene, 298 K, 600 MHz.

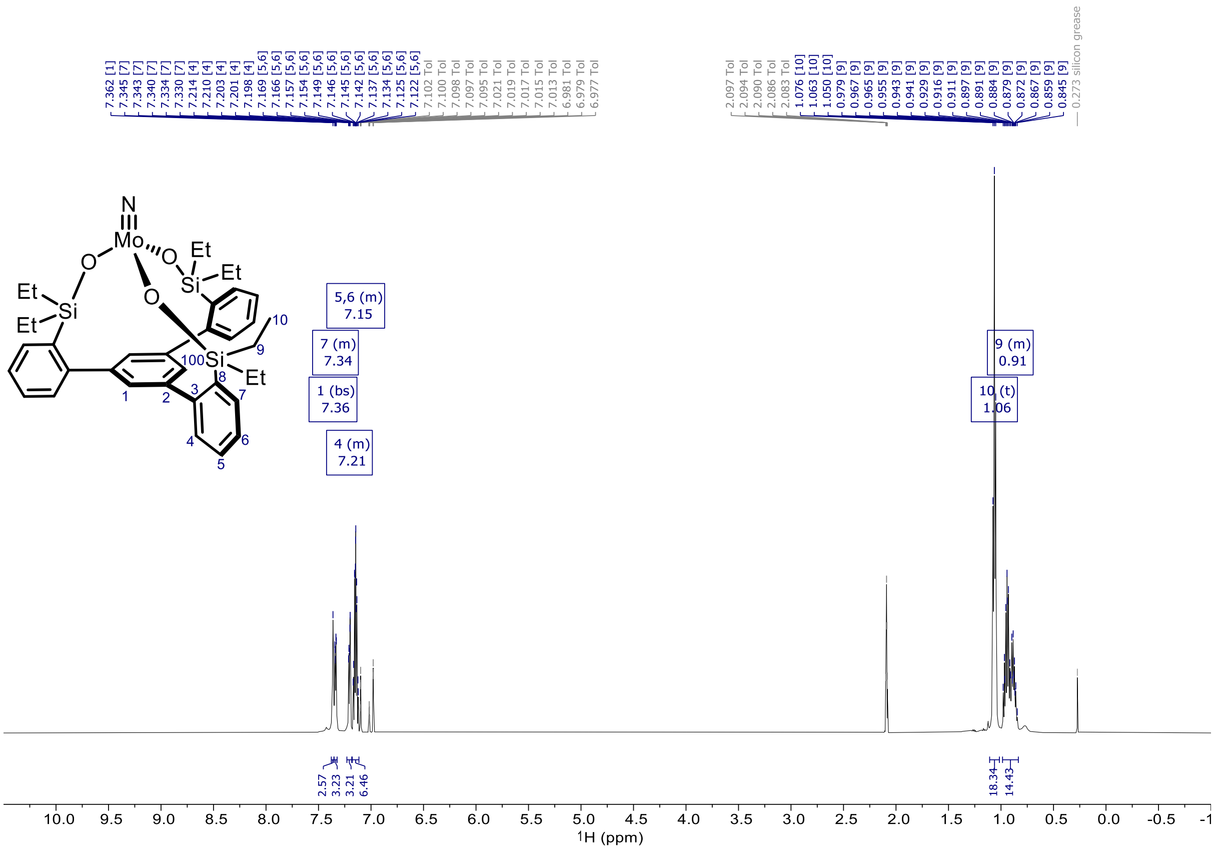

^{13}C NMR spectrum of complex 3c: [D$_8$]-toluene, 298 K, 151 MHz.

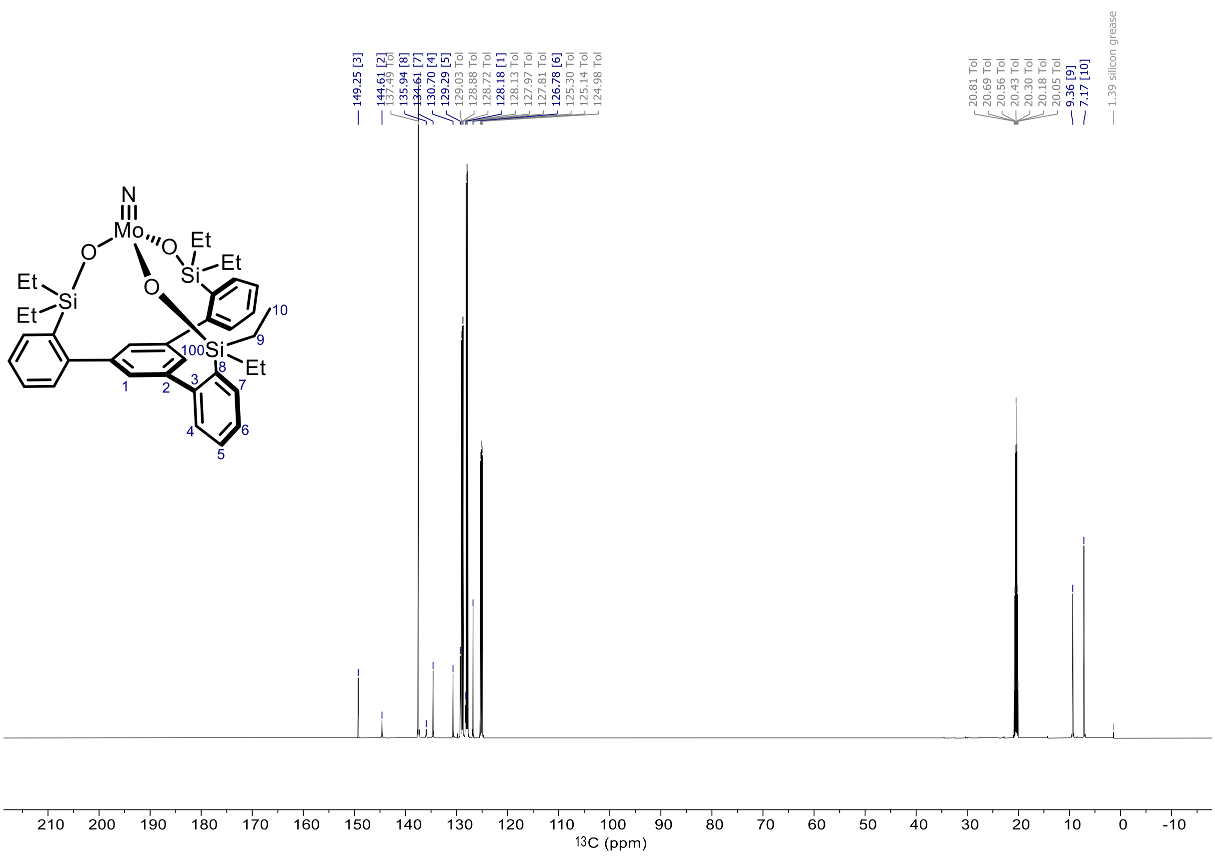

^{1}H-^{13}C HSQC NMR spectrum of complex 3c: [D$_8$]-toluene, 298 K, 600 MHz, 151 MHz.

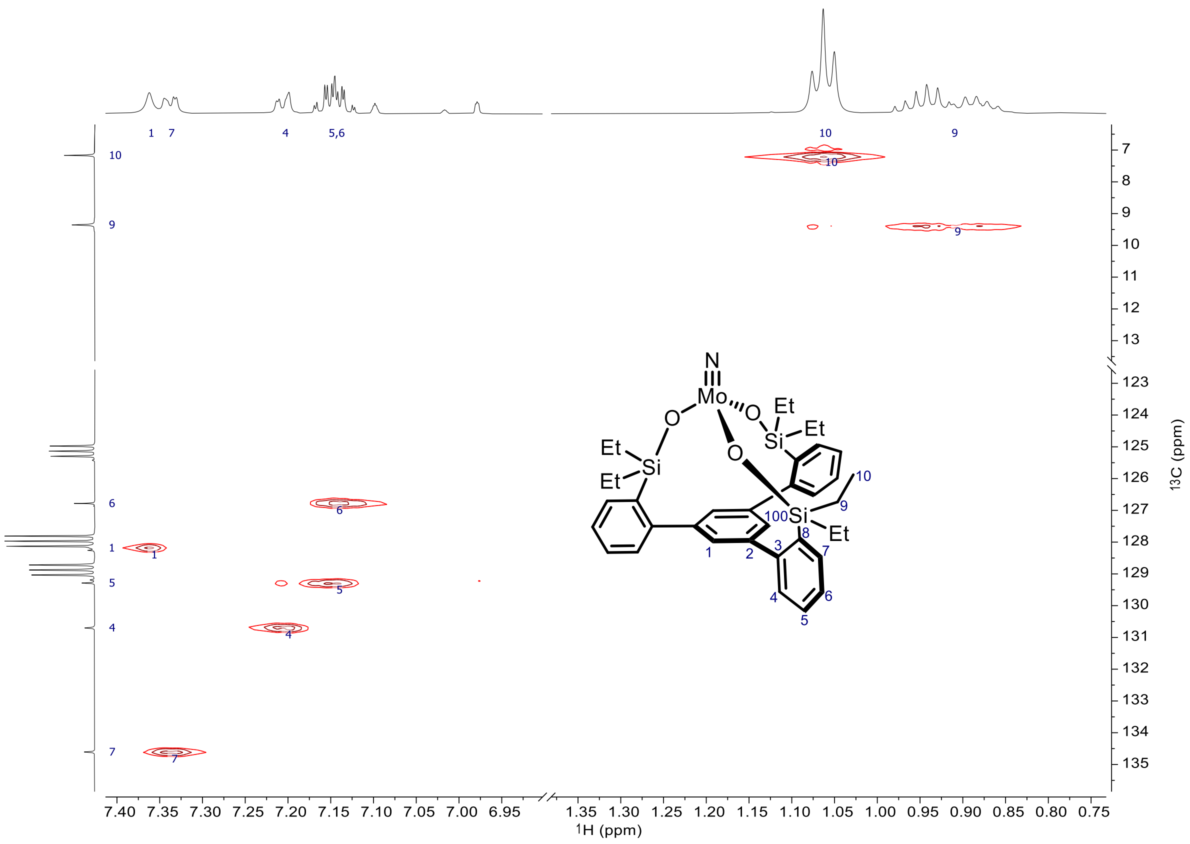

^{1}H-^{13}C HMBC NMR spectrum of complex 3c: [D$_8$]-toluene, 298 K, 600 MHz, 151 MHz.

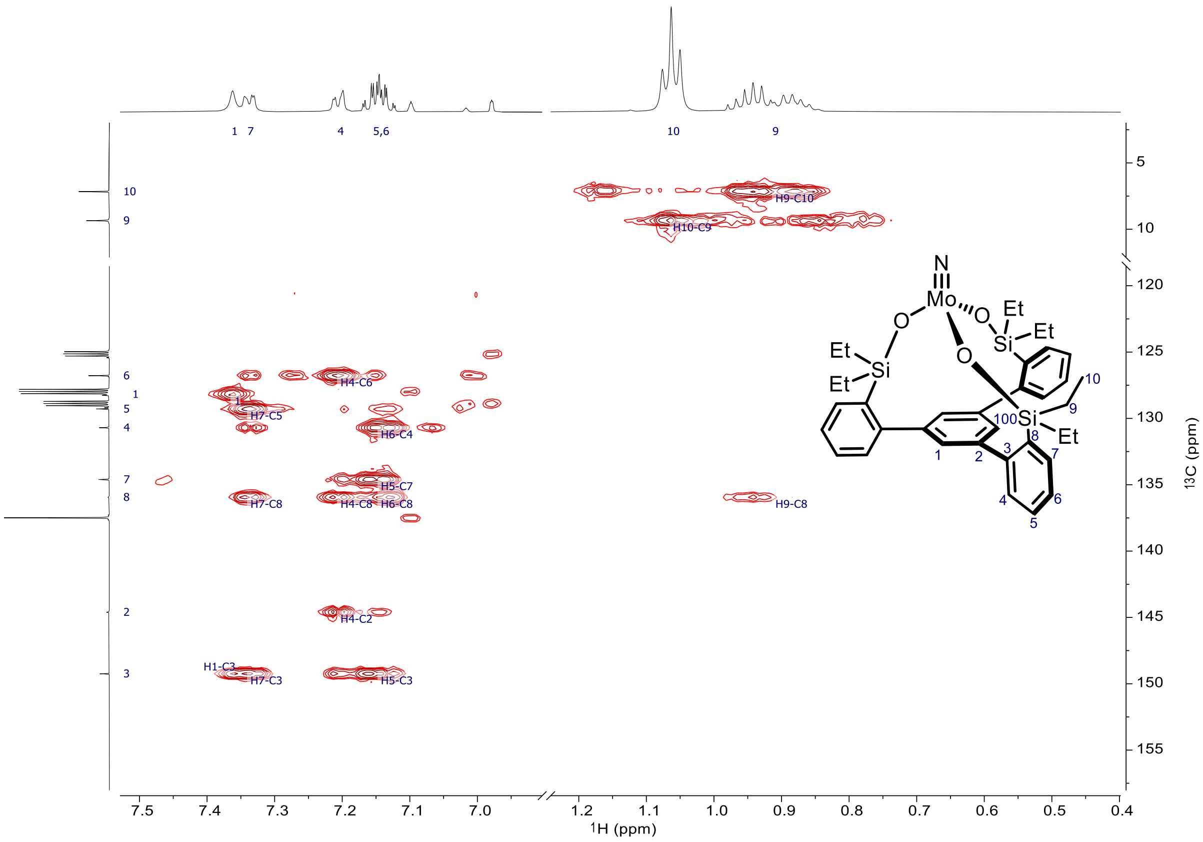

^{1}H-^{1}H COSY NMR spectrum of complex 3c: [D$_8$]-toluene, 298 K, 600 MHz, 600 MHz.

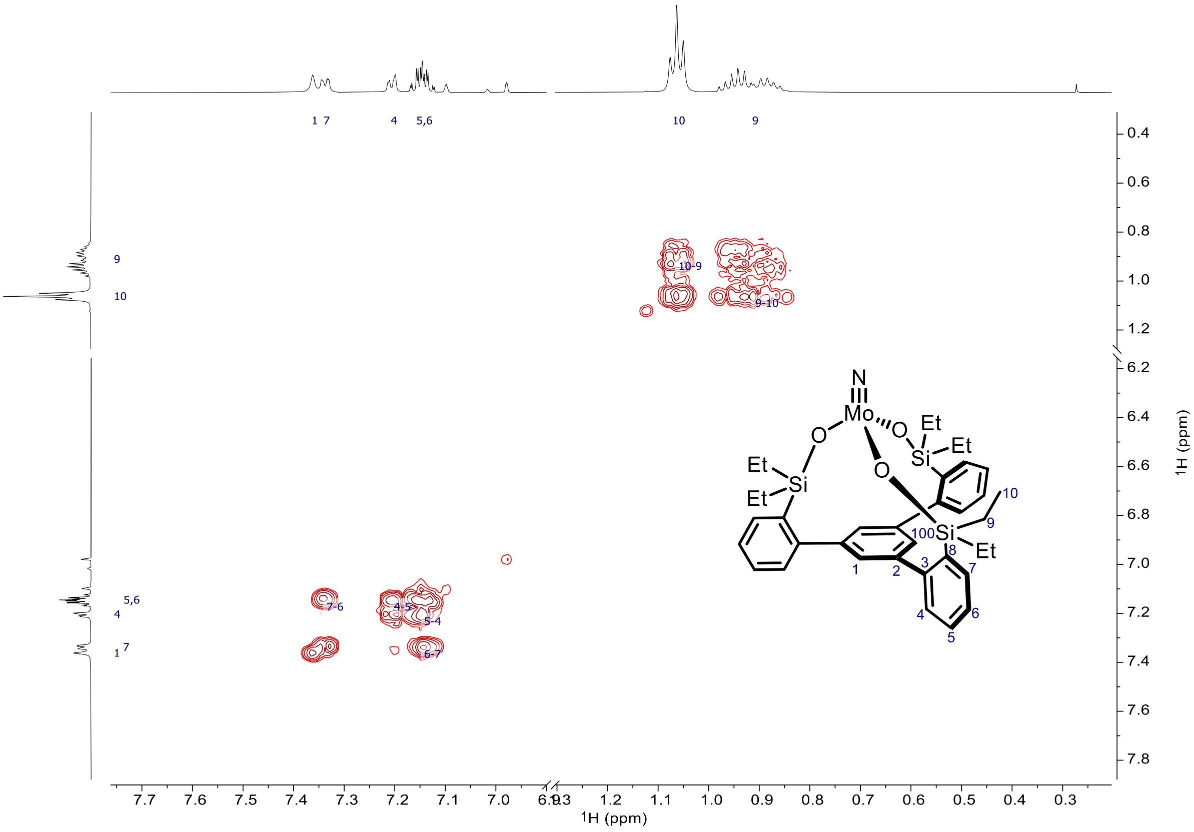

^{29}Si NMR spectrum of complex 3c: [D$_8$]-toluene, 298 K, 119 MHz.

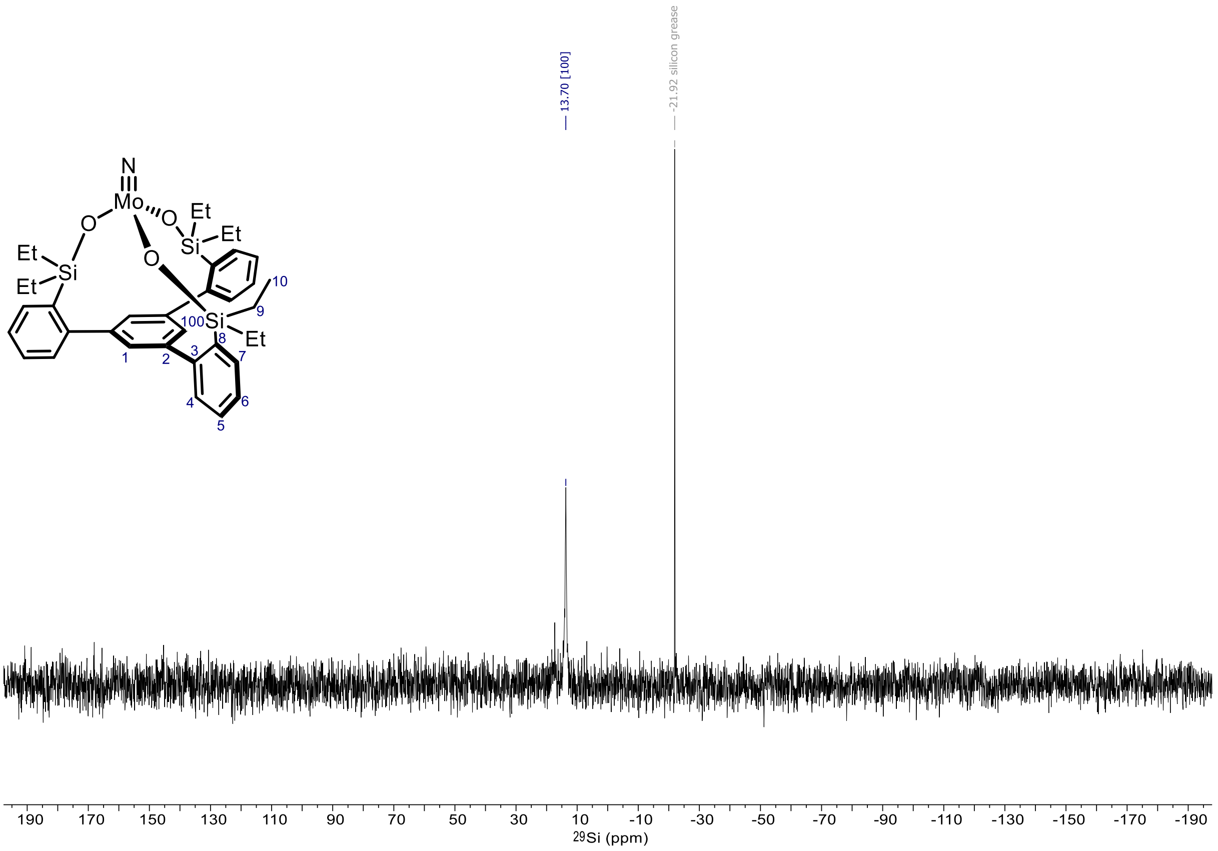

^{1}H-^{29}Si HMBC NMR spectrum of complex 3c: [D$_8$]-toluene, 298 K, 600 MHz, 119 MHz.

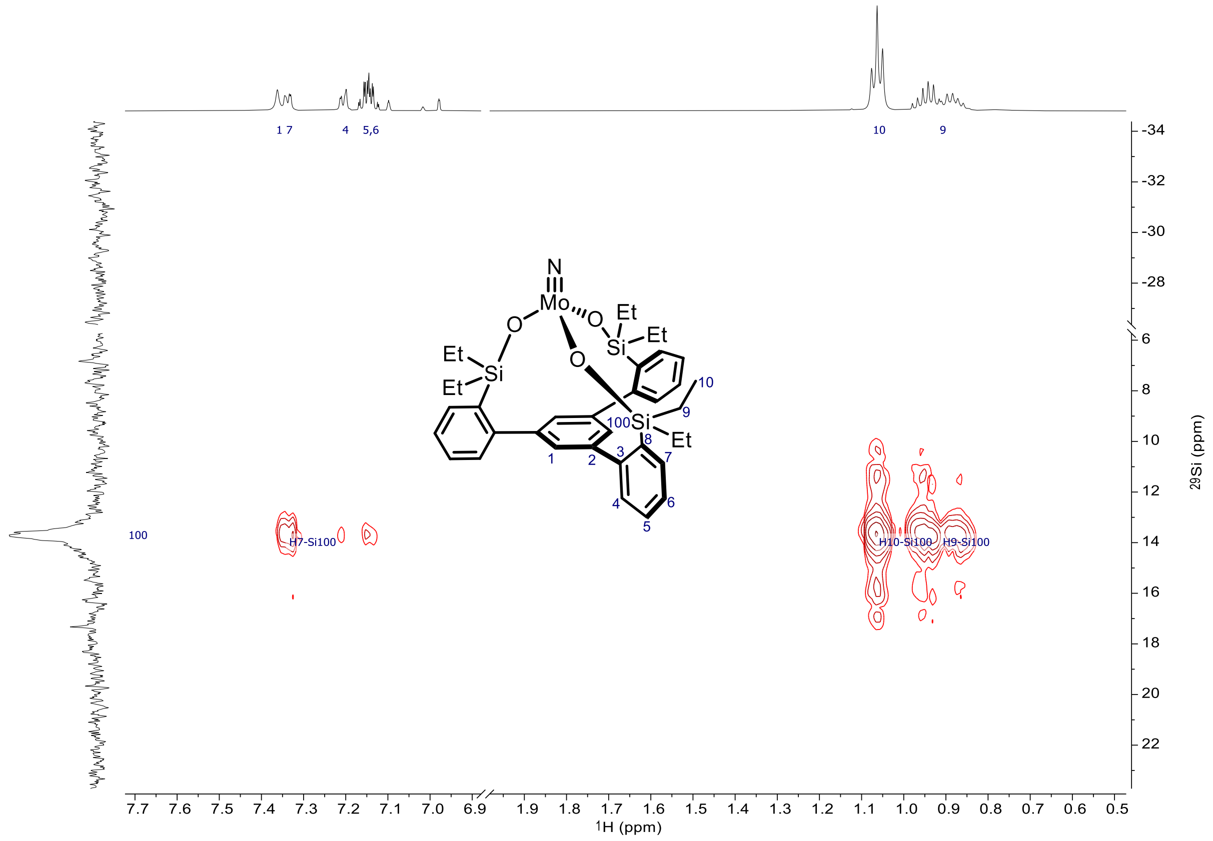

^{13}C DEPT-135 NMR spectrum of complex 3c: [D$_8$]-toluene, 298 K, 151 MHz.

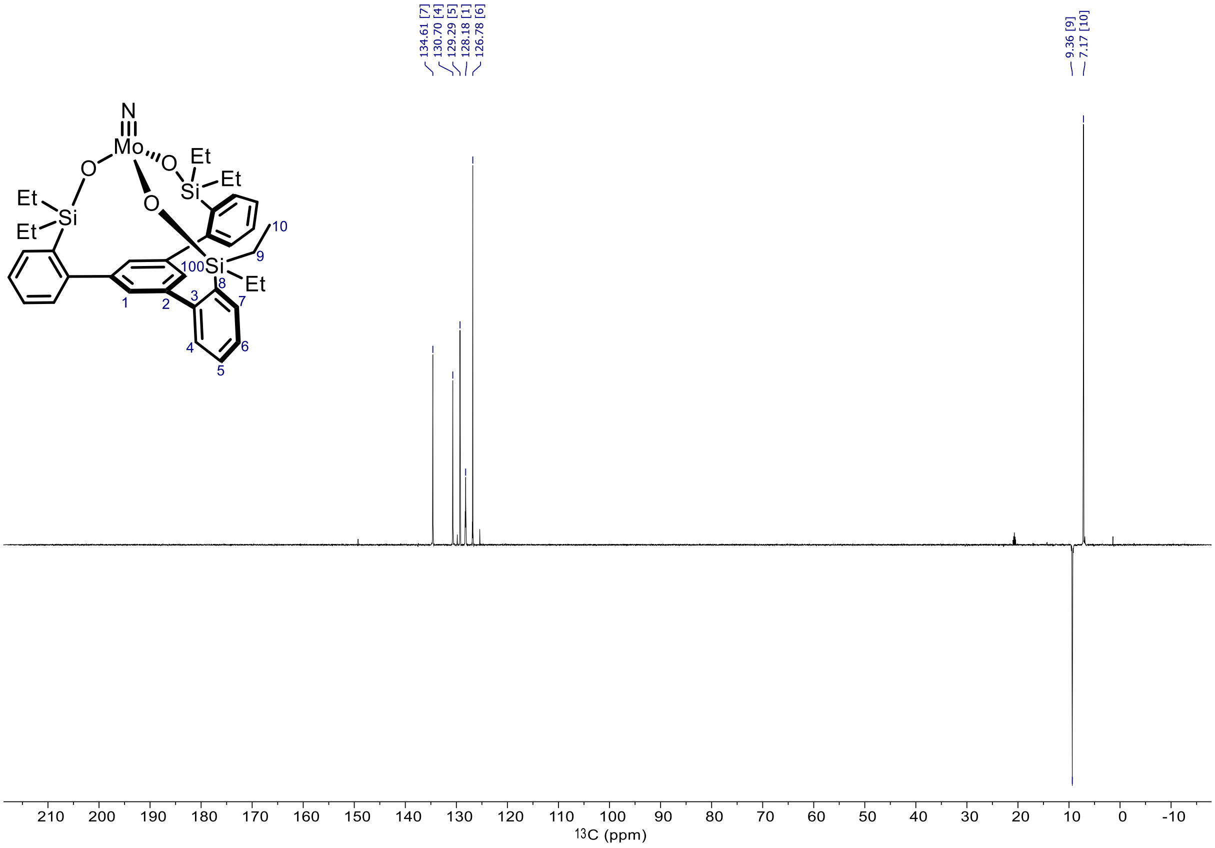

^{95}Mo NMR spectrum of complex 3c: [D$_8$]-toluene, 298 K, 26 MHz.

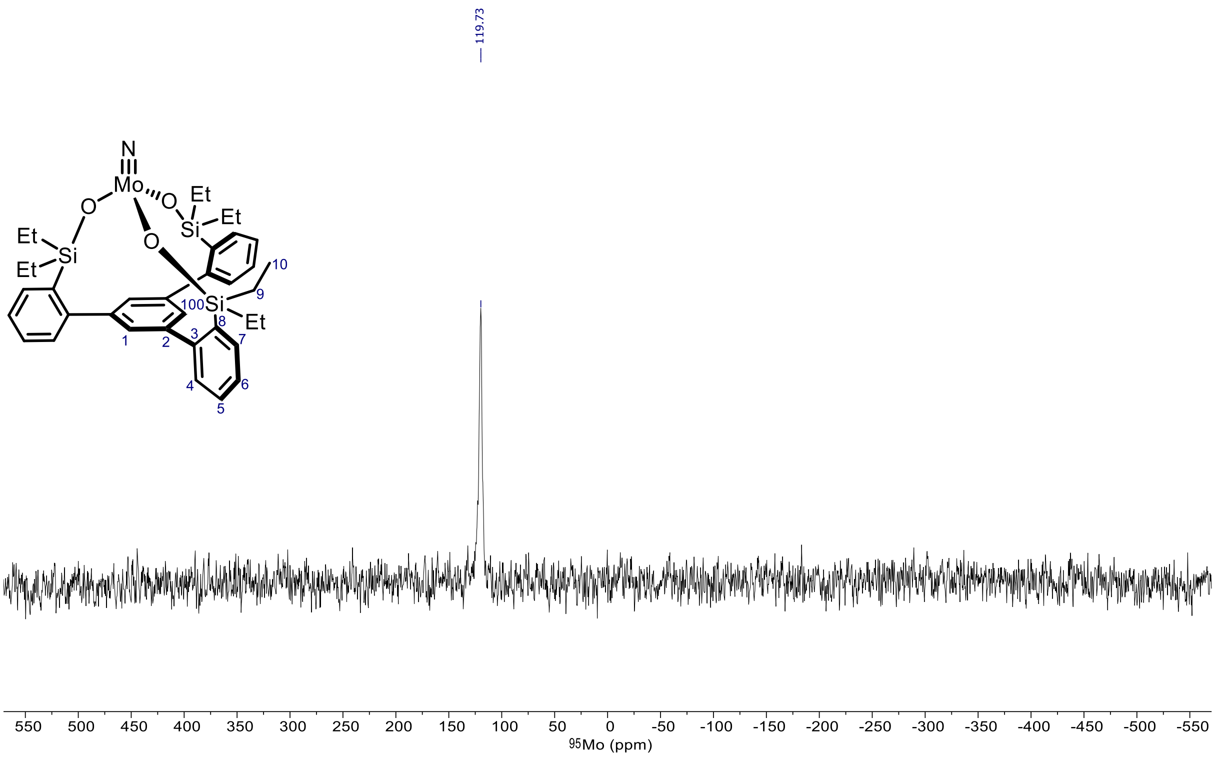

^{14}N NMR spectrum of complex 3c: [D$_8$]-toluene, 333 K, 29 MHz.

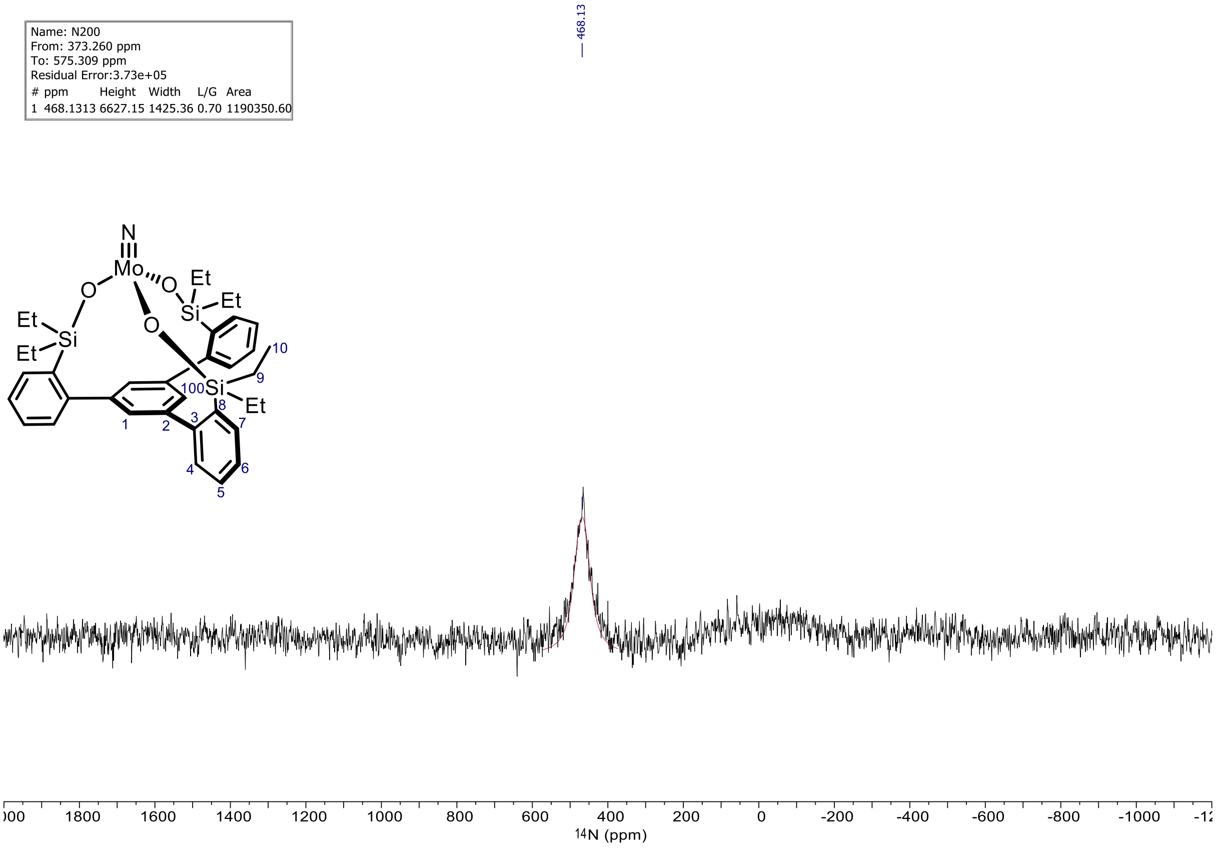

NMR analysis of complex 3d: The two methyl groups C10 and C11 are diastereotopic. H5 and H6 form a complex multiplet. It was difficult to extract their exact chemical shift. Therefore, the average shifts of the multiplet are given. The signal of C5 is overlapping with one of the solvent peaks. The shift was verified *via* ^{13}C DEPT-135 NMR and ^{1}H-^{13}C HSQC NMR experiments. ^{14}N NMR data was measured 333 K and the shift was extracted after peak fitting.

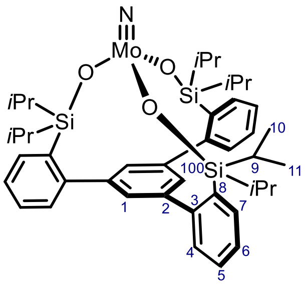

^{1}H NMR spectrum of complex 3d: [D$_8$]-toluene, 298 K, 600 MHz.

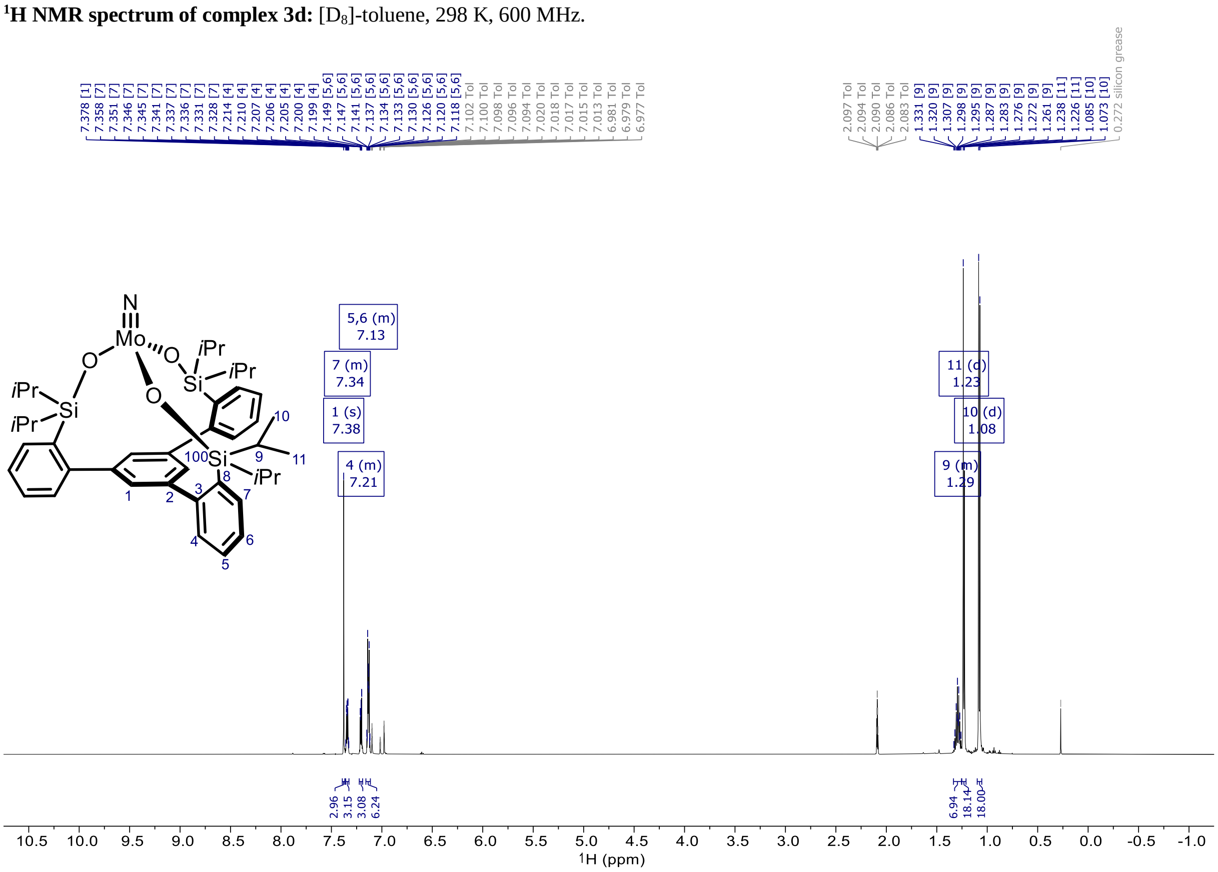

^{13}C NMR spectrum of complex 3d: [D$_8$]-toluene, 298 K, 151 MHz.

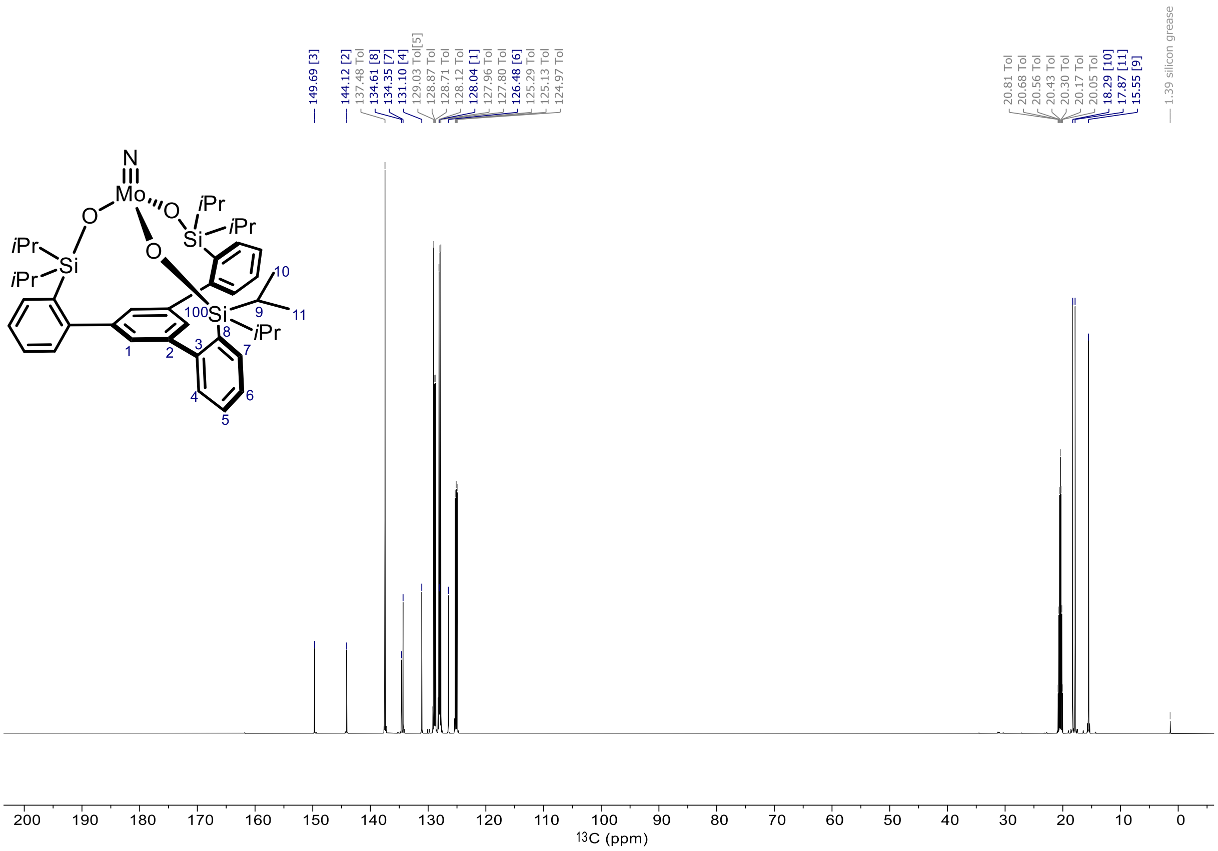

^{1}H-^{13}C HSQC NMR spectrum of complex 3d: [D$_8$]-toluene, 298 K, 600 MHz, 151 MHz.

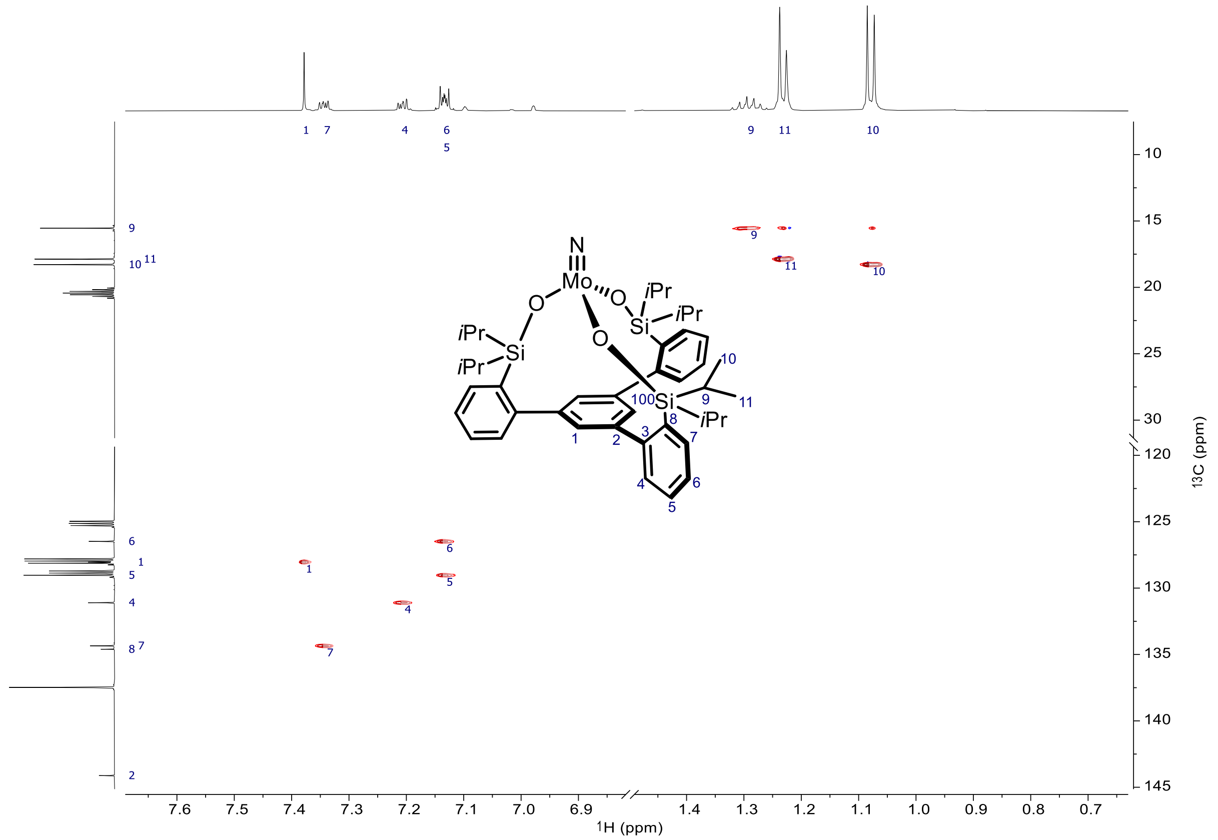

^{1}H-^{13}C HMBC NMR spectrum of complex 3d: [D$_8$]-toluene, 298 K, 600 MHz, 151 MHz.

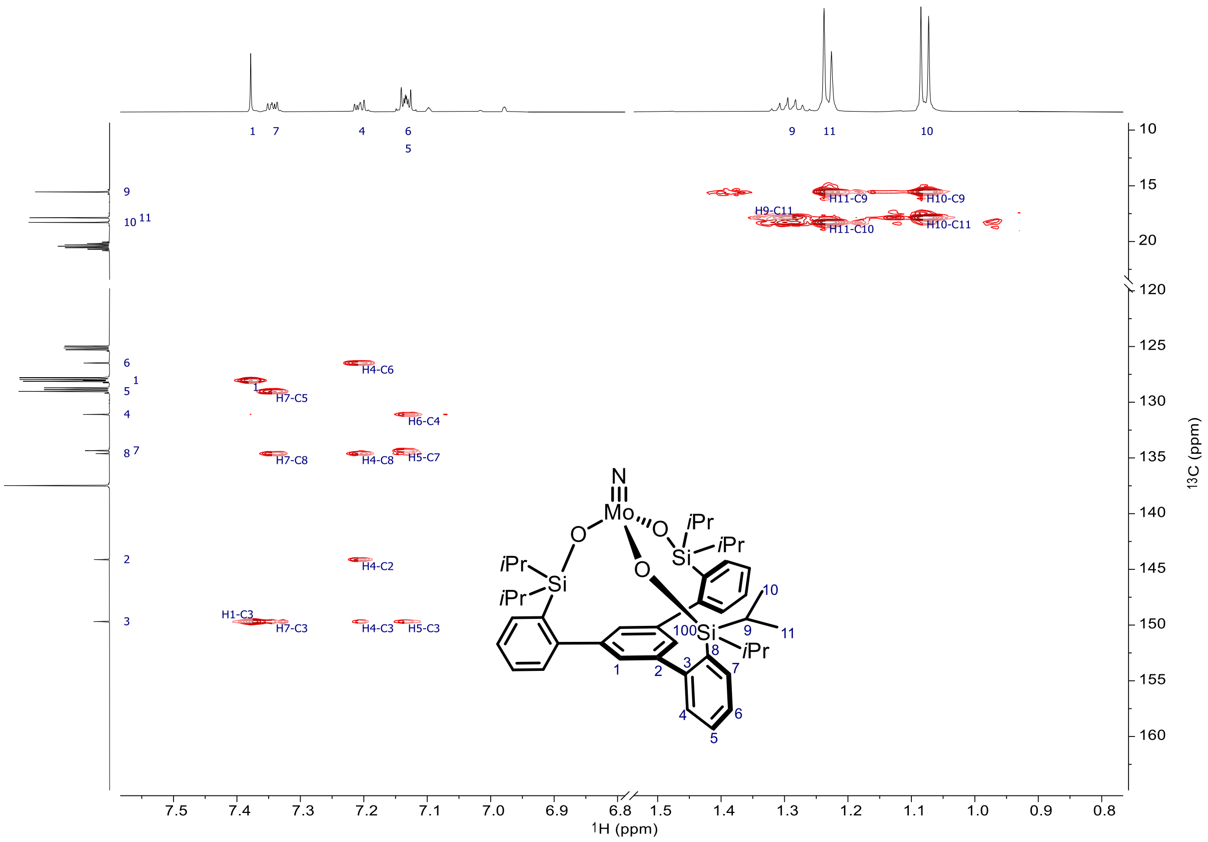

^{1}H-^{1}H COSY NMR spectrum of complex 3d: [D$_8$]-toluene, 298 K, 600 MHz, 600 MHz.

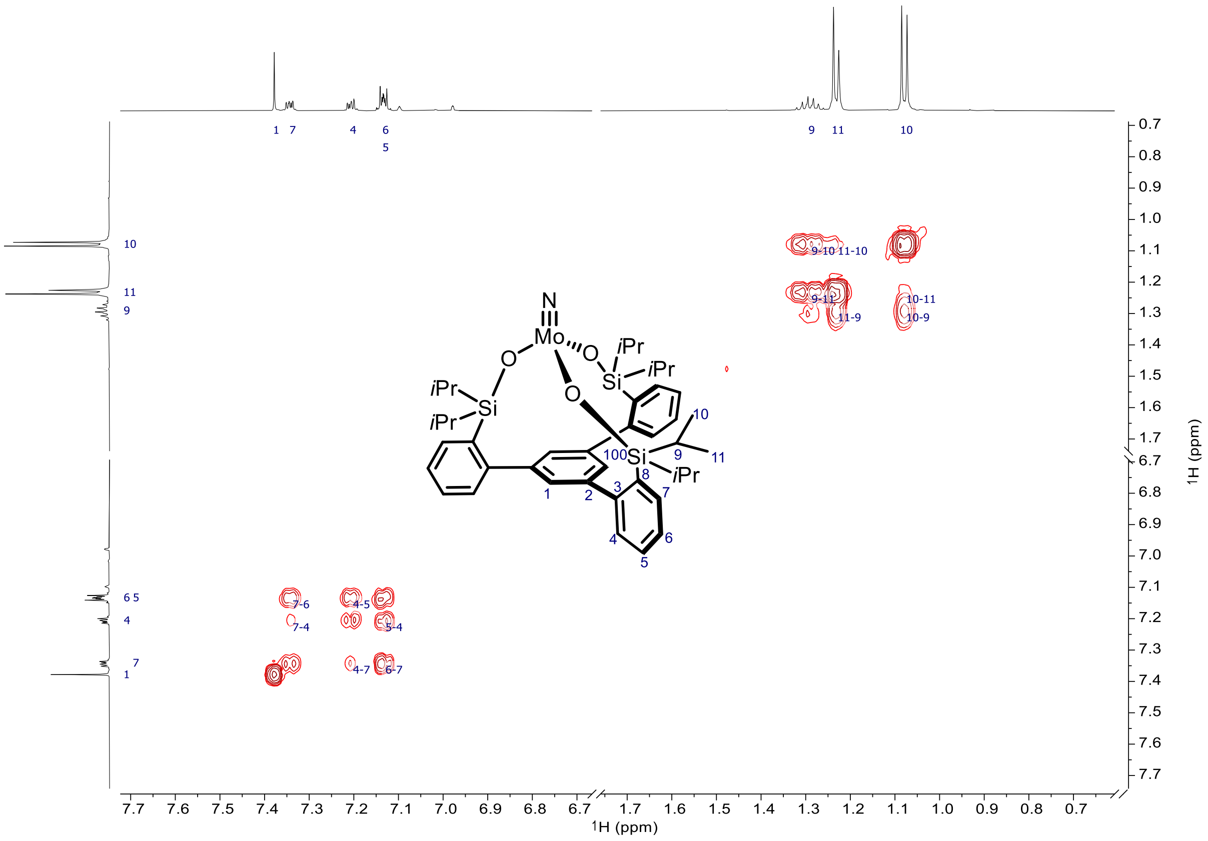

^{29}Si NMR spectrum of complex 3d: [D$_8$]-toluene, 298 K, 119 MHz and **^{1}H-^{29}Si HMBC NMR spectrum:** [D$_8$]-toluene, 298 K, 600 MHz, 119 MHz.

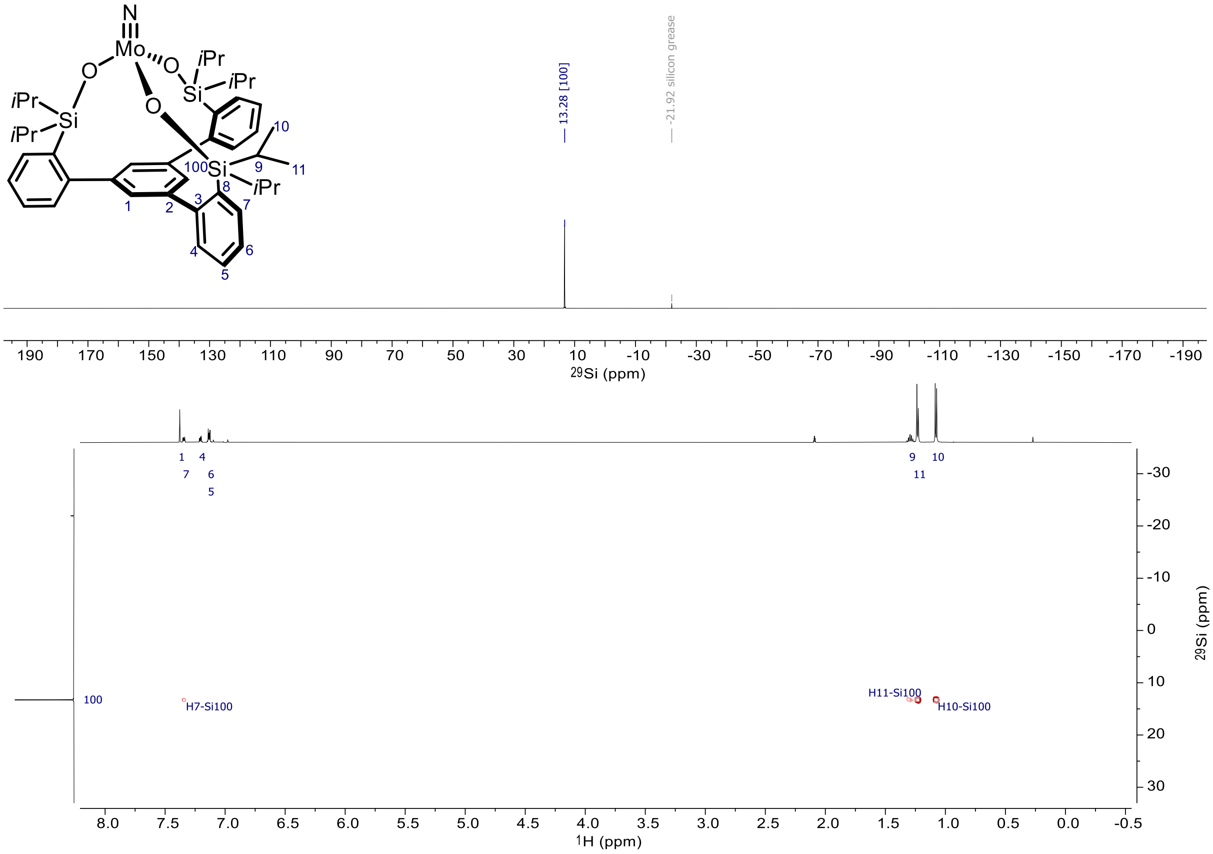

^{13}C DEPT-135 NMR spectrum of complex 3d: [D$_8$]-toluene, 298 K, 151 MHz.

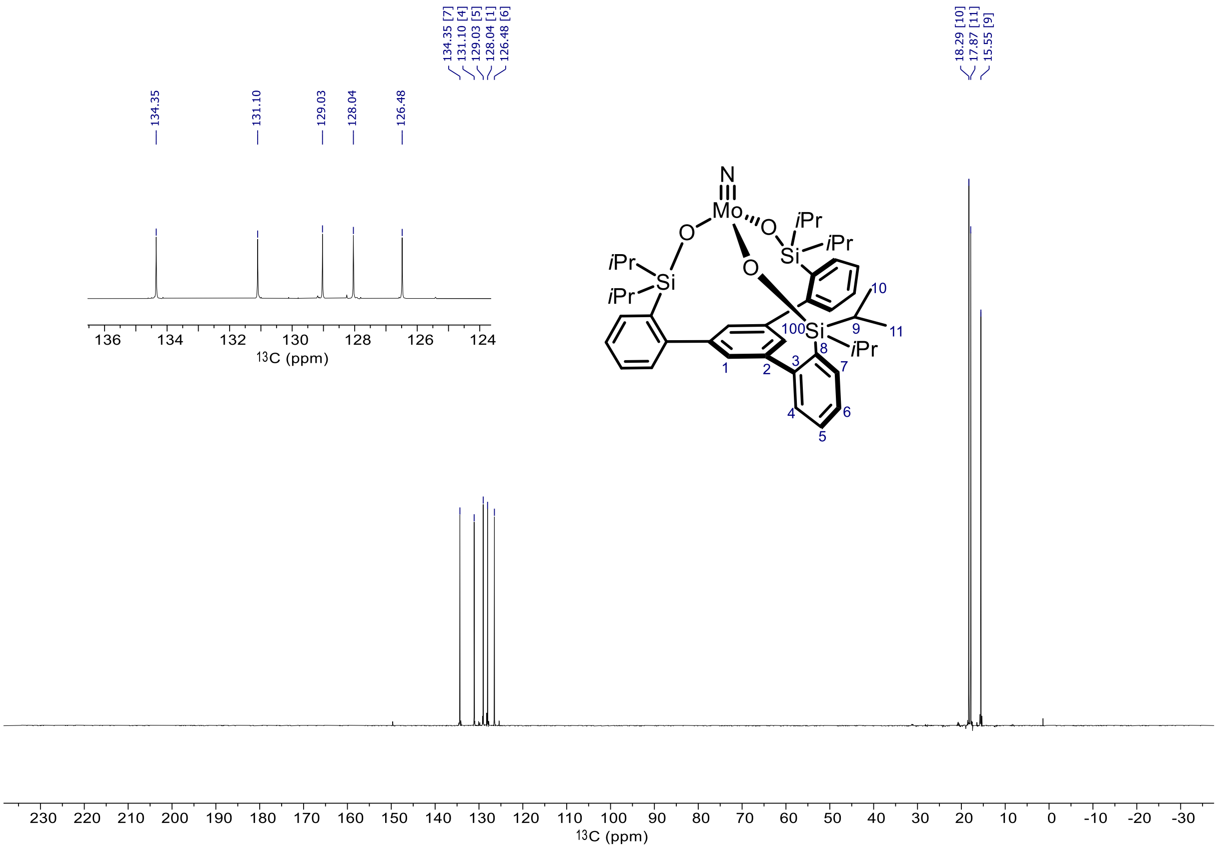

[95]Mo NMR spectrum of complex 3d: [D$_8$]-toluene, 298 K, 26 MHz.

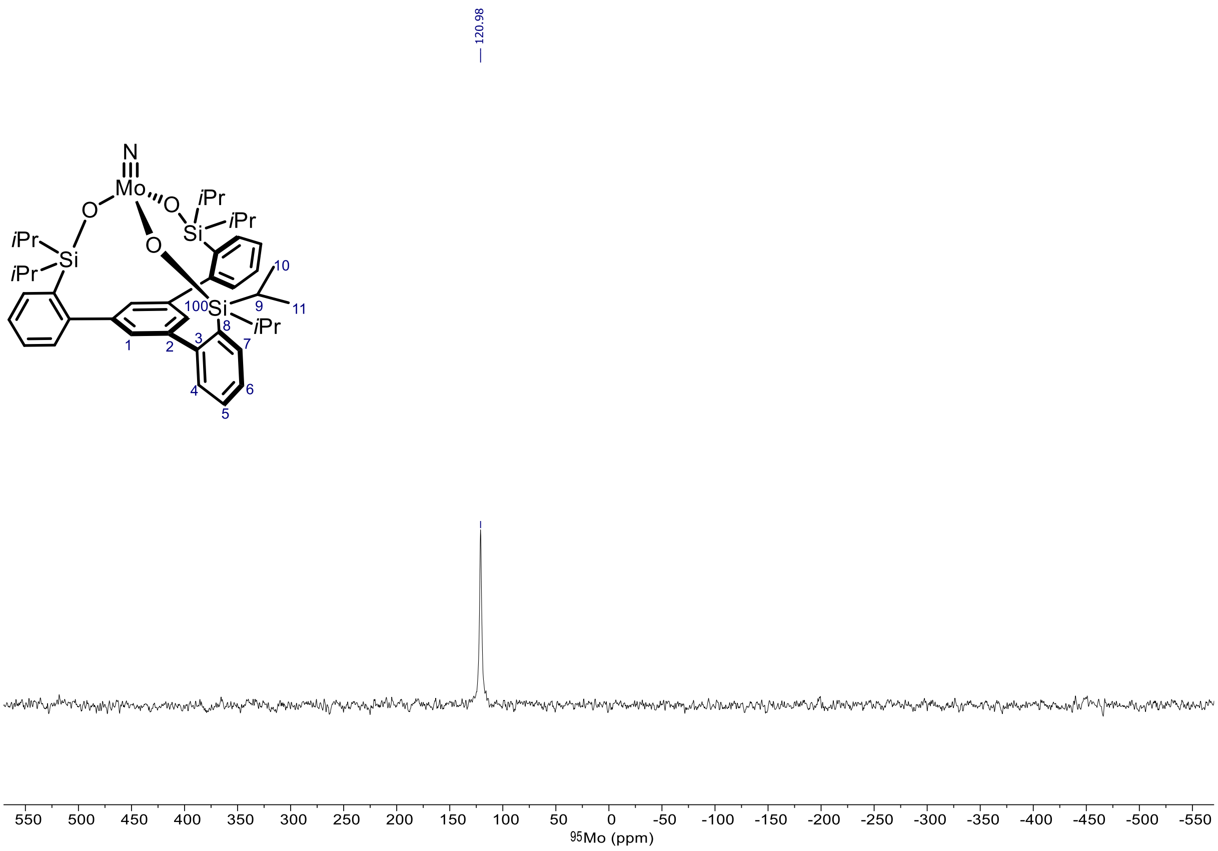

[14]N NMR spectrum of complex 3d: [D$_8$]-toluene, 333 K, 29 MHz.

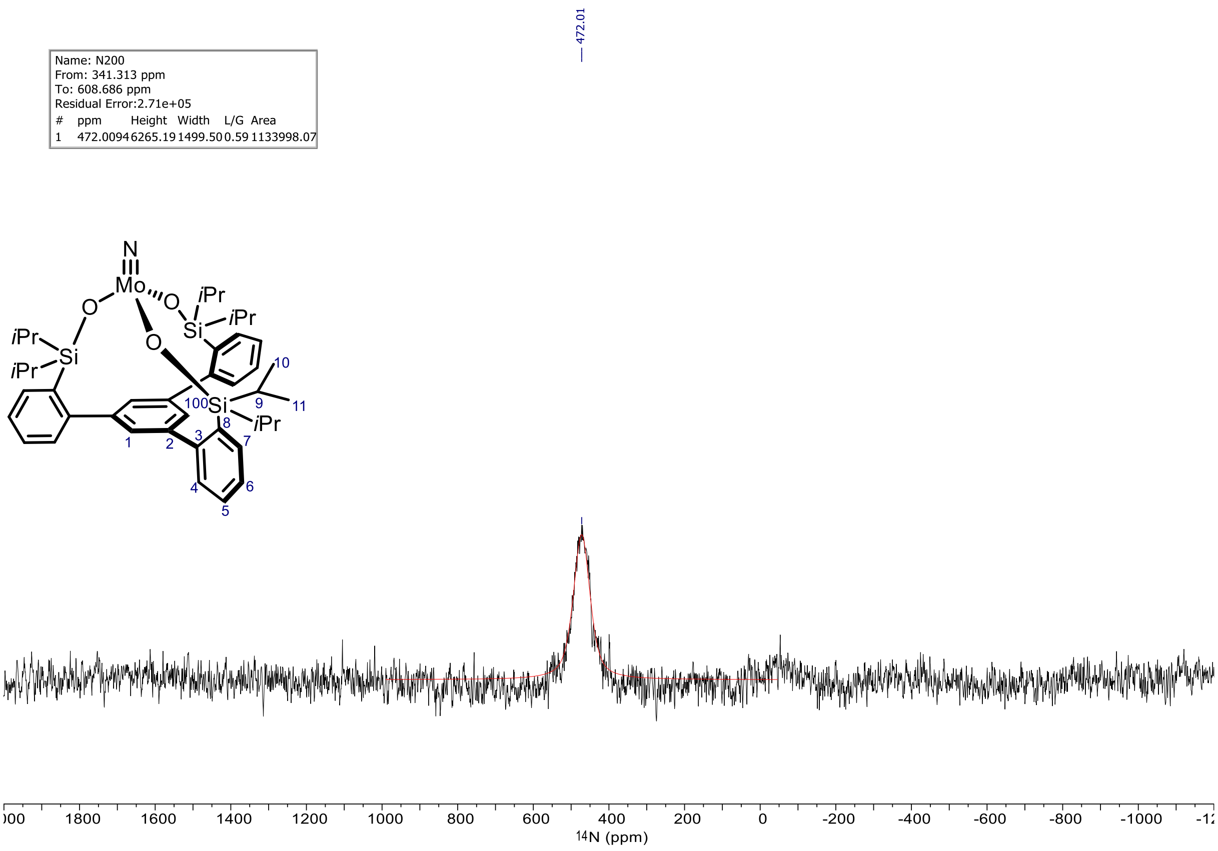

NMR analysis of complex 3e: NMR analysis indicates that the complex is C_3 symmetric. The ^{14}N NMR was measured at 333 K to obtain shaper signals and the shift was extracted after peak fitting.

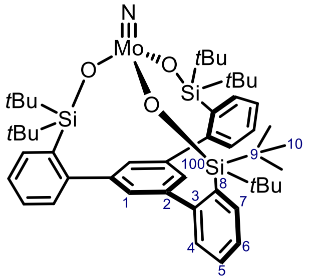

^{1}H NMR spectrum of complex 3e: [D$_8$]-toluene, 298 K, 600 MHz.

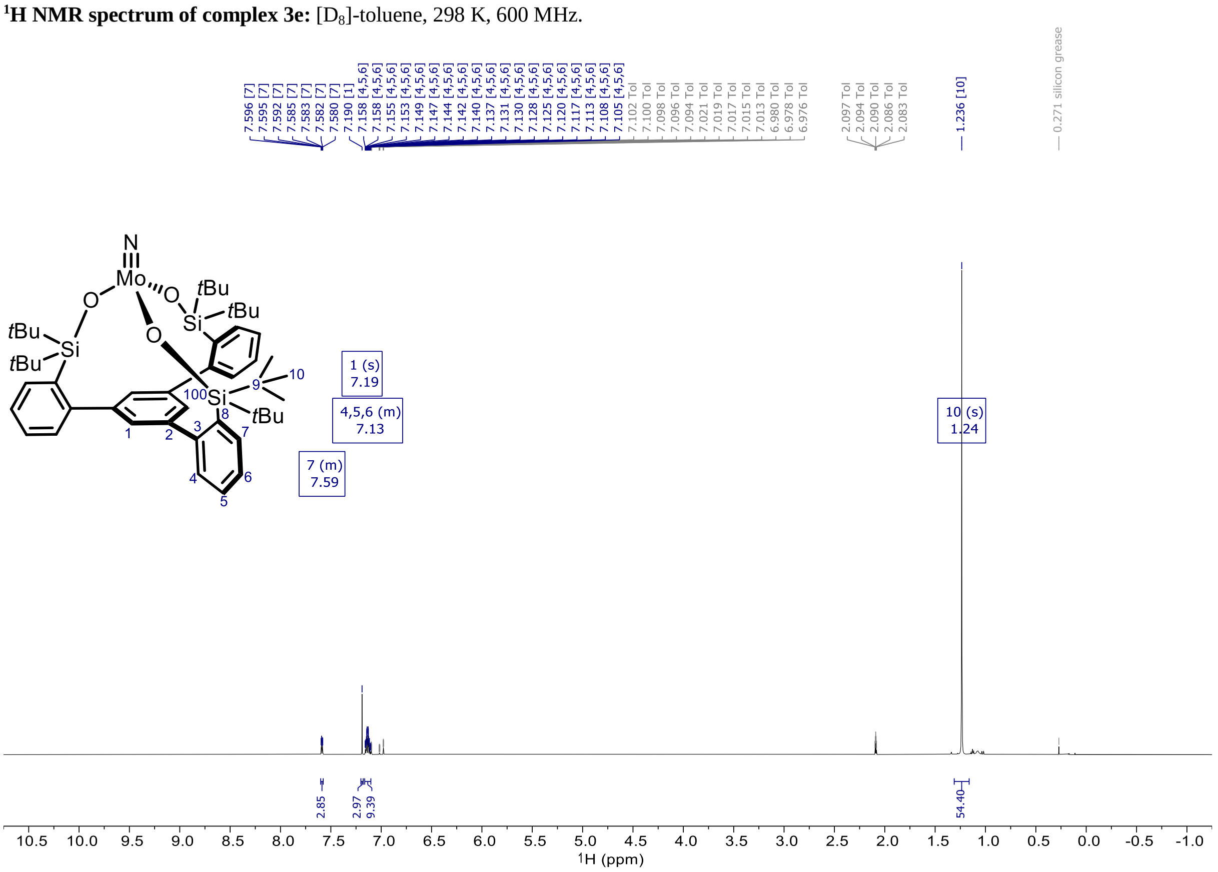

^{13}C NMR spectrum of complex 3e: [D$_8$]-toluene, 298 K, 151 MHz.

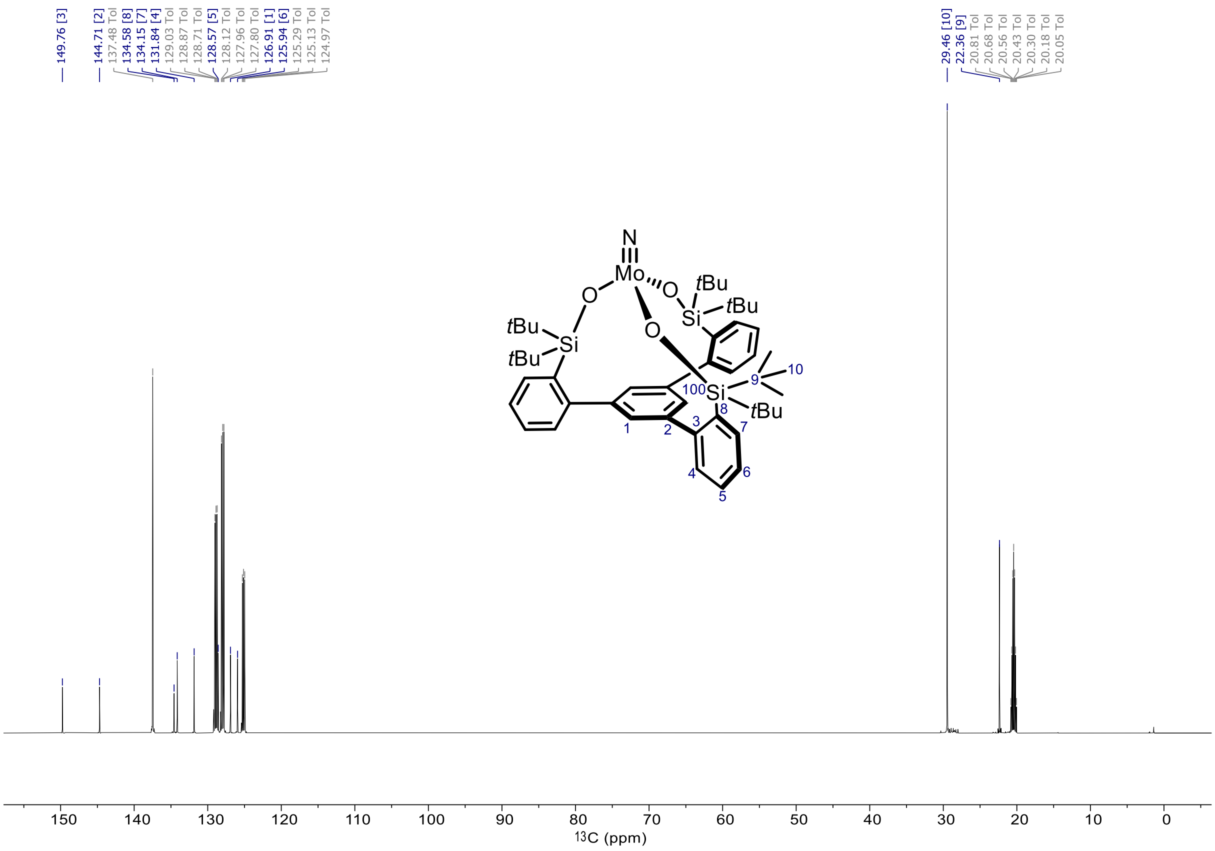

^{1}H-^{13}C HSQC NMR spectrum of complex 3e: [D$_8$]-toluene, 298 K, 600 MHz, 151 MHz.

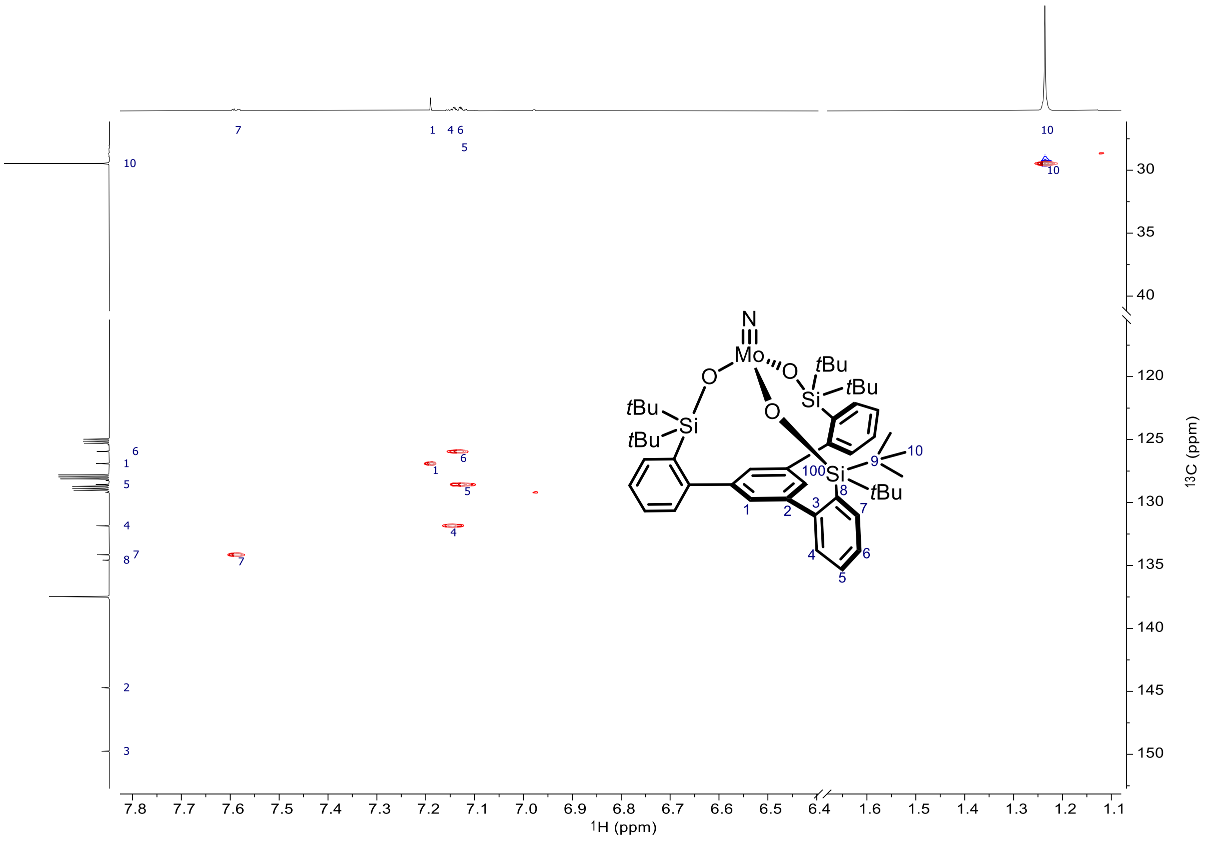

^{1}**H-**^{13}**C HMBC NMR spectrum of complex 3e:** [D$_8$]-toluene, 298 K, 600 MHz, 151 MHz.

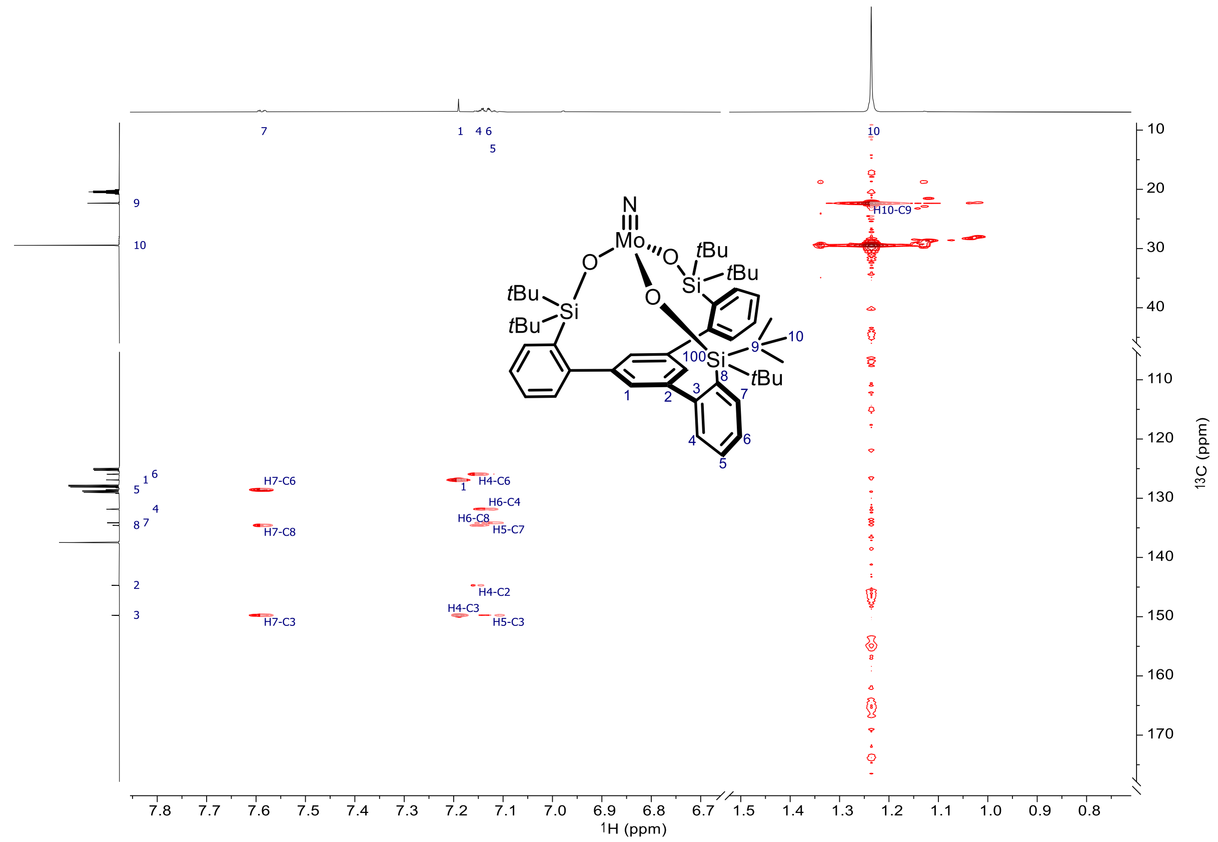

^{1}**H-**^{1}**H COSY NMR spectrum of complex 3e:** [D$_8$]-toluene, 298 K, 600 MHz, 600 MHz.

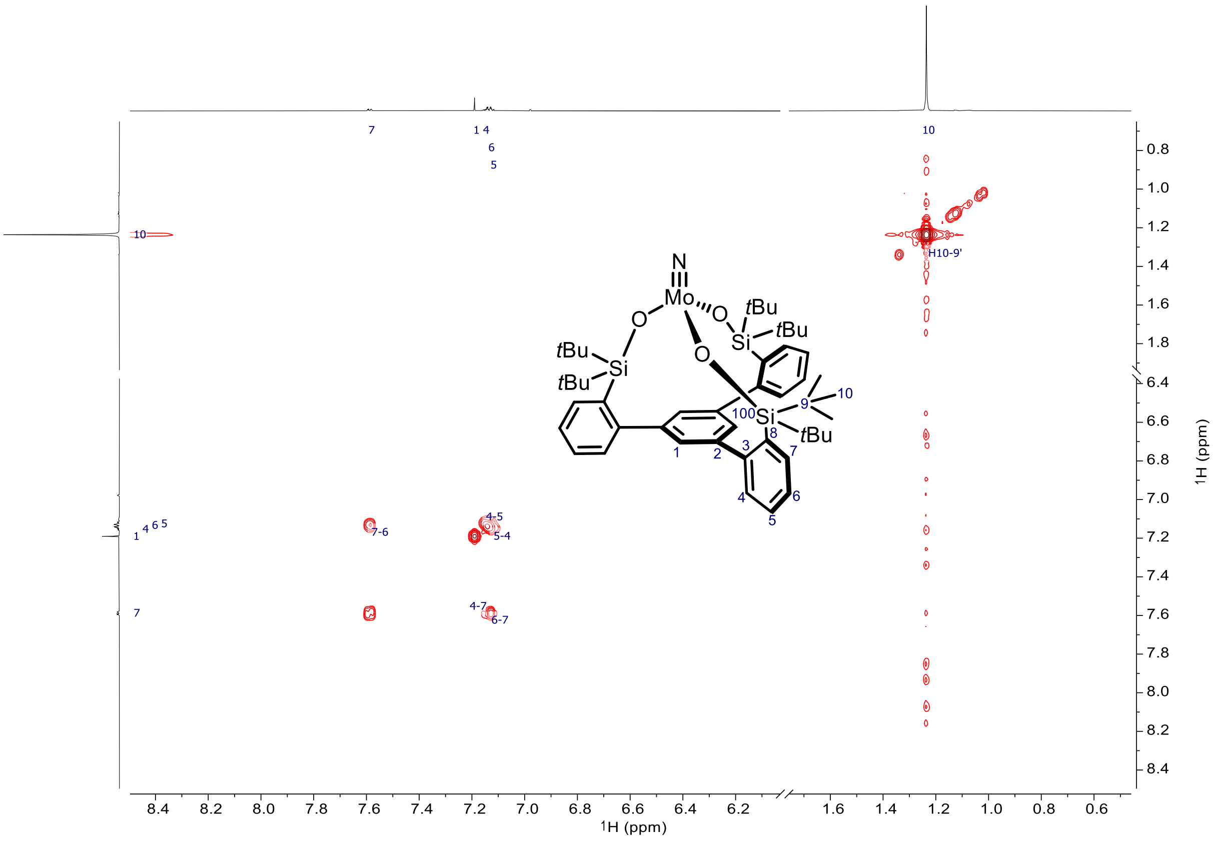

^{1}H-^{1}H NOESY NMR spectrum of complex 3e: [D_8]-toluene, 298 K, 600 MHz, 600 MHz.

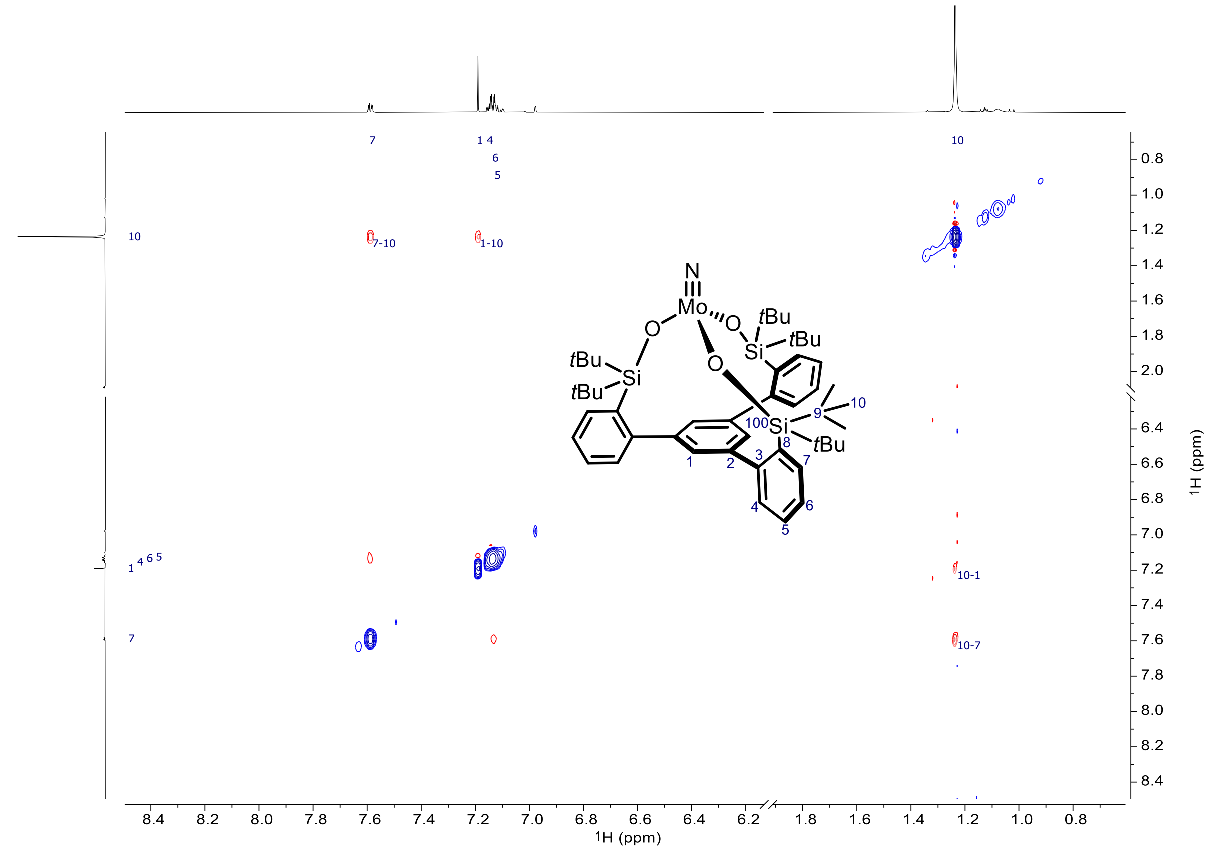

^{29}Si resINEPTrd NMR spectrum of complex 3e: [D_8]-toluene, 298 K, 119 MHz and **^{1}H-^{29}Si HMBC NMR spectrum:** [D_8]-toluene, 298 K, 600 MHz, 119 MHz.

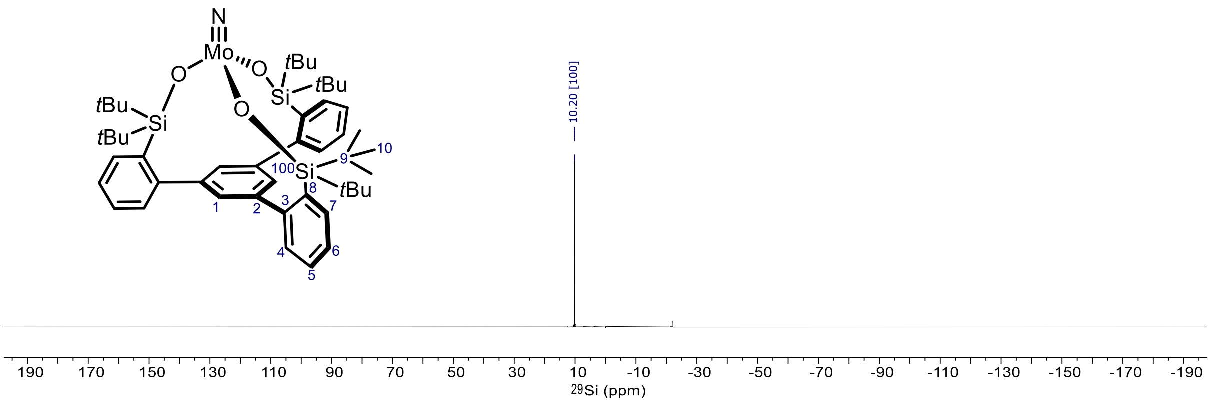

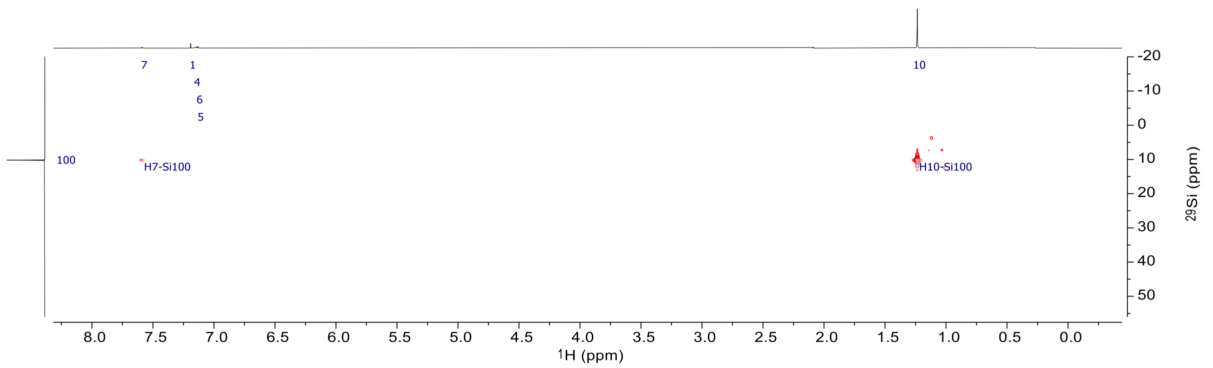

^{13}C DEPT-135 NMR spectrum of complex 3e: [D$_8$]-toluene, 298 K, 151 MHz.

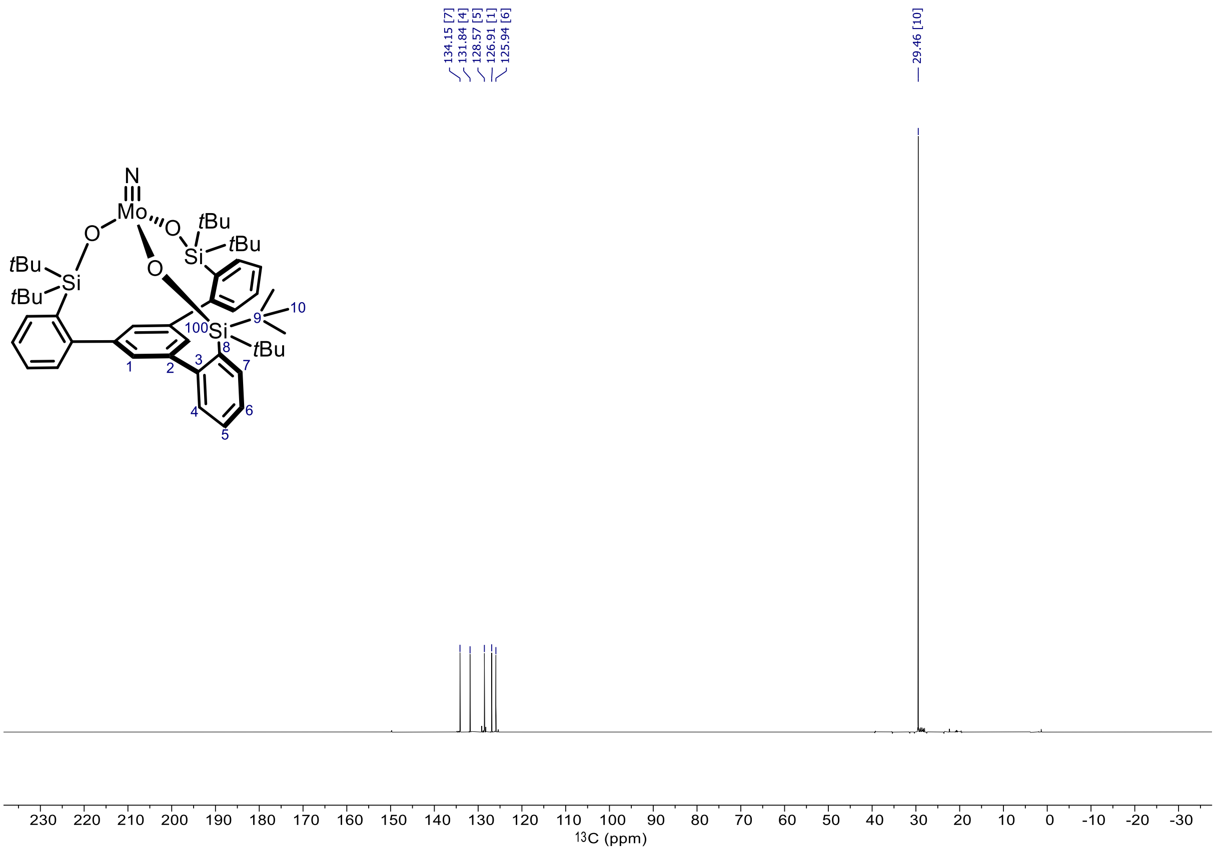

^{95}Mo NMR spectrum of complex 3e: [D$_8$]-toluene, 298 K, 26 MHz.

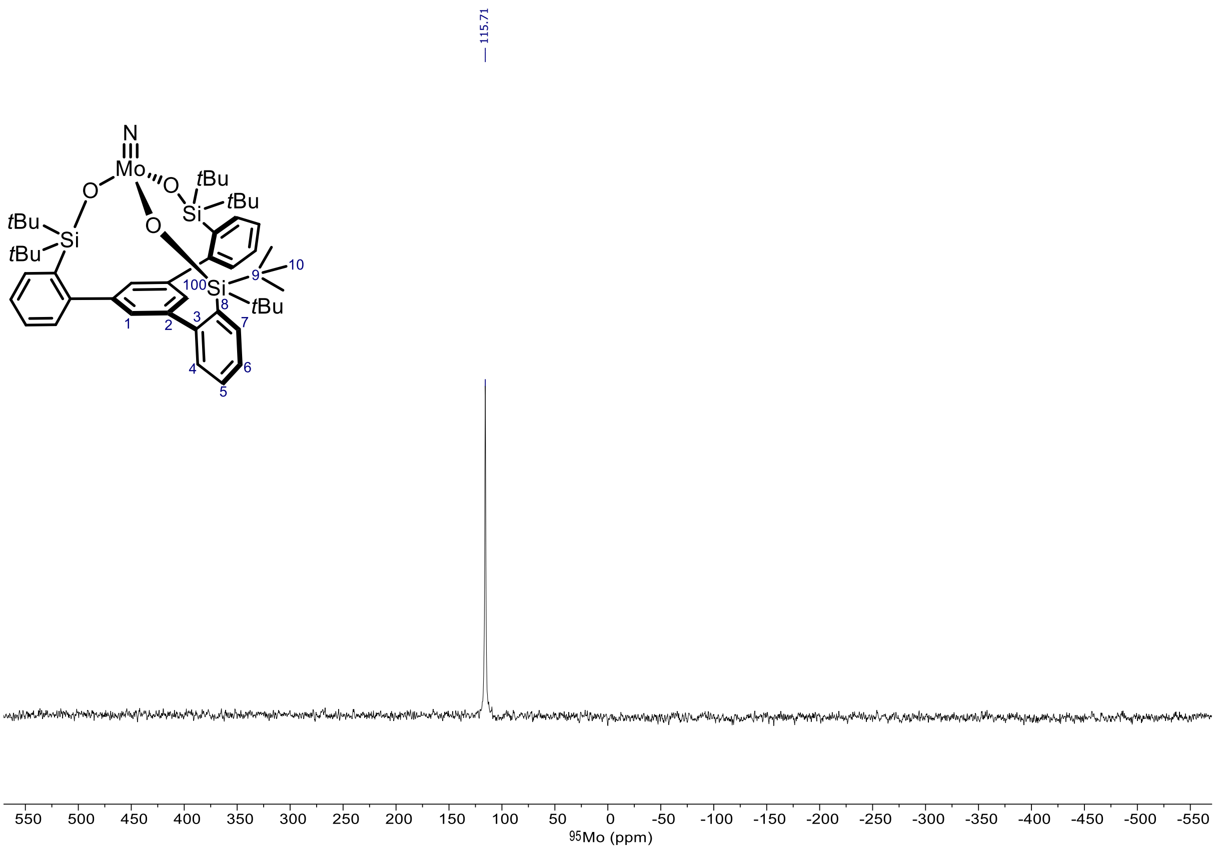

^{14}N NMR spectrum of complex 3e: [D$_8$]-toluene, 333 K, 29 MHz.

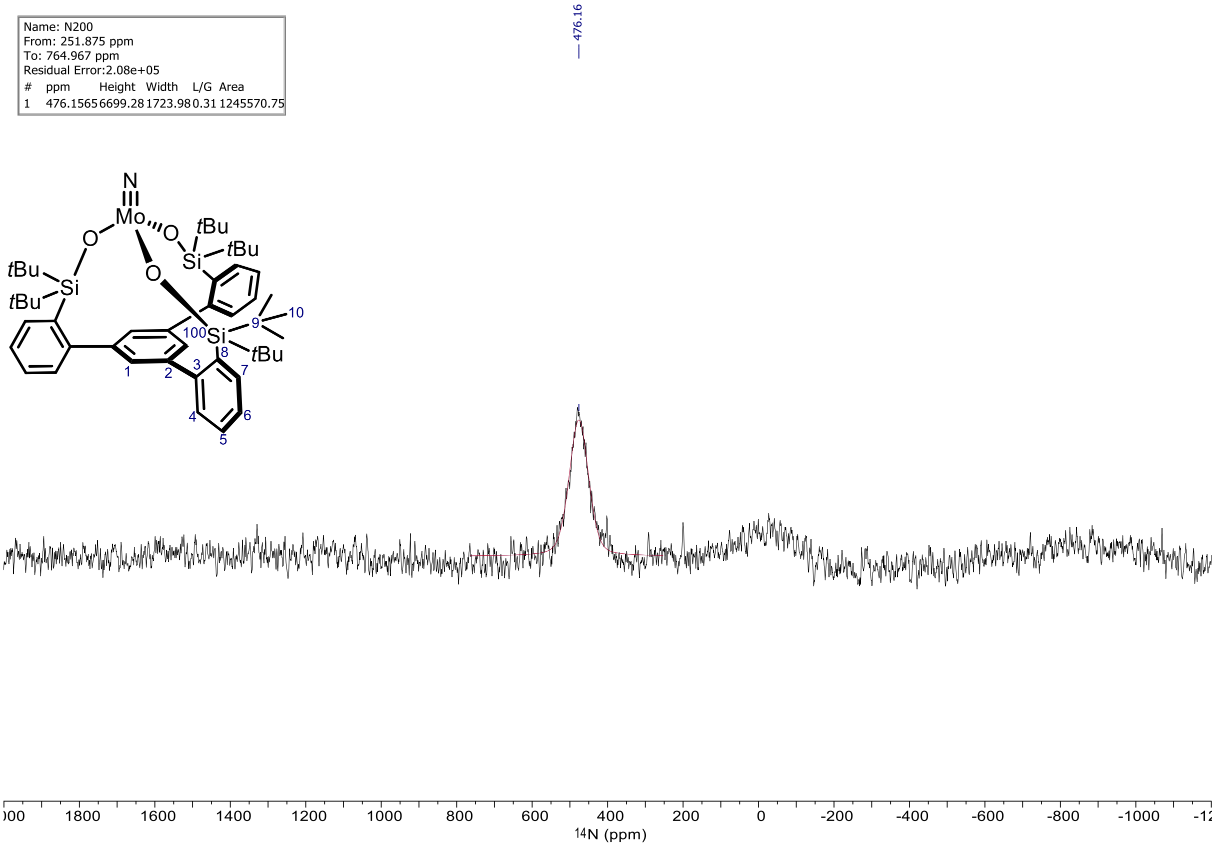

NMR analysis of complex 13a: The lateral phenyl groups are diastereotopic; one of them has an increased rotational barrier. This is also true for the nitrogen-aryl bond of the amide ligands (please see the ^{1}H and ^{13}C NMR spectra at different temperatures). The tripodal ligand framework itself is C_3-symmetric. Multiple atoms have rather unusual chemical shifts, likely due to the paramagnetic nature of the complex. Generally, the closer they are located to the Mo−N−Mo moiety, the stronger the paramagnetic shift. In some cases no HMBC cross peaks were observed, but ^{13}C assignments were done by high temperature 2D NMR data. However, the shift of C13 could not be confirmed with 2D NMR data and was only observed in the 1D ^{13}C NMR spectrum. Due to the high sensitivity of this complex, ^{1}H and ^{13}C NMR spectra always showed small amounts of free aniline HN(*t*Bu)Ar and the terminal nitrido complex [N≡Mo(N(*t*Bu)Ar)$_3$] (**10**) (Ar = 3,5-dimethylphenyl).

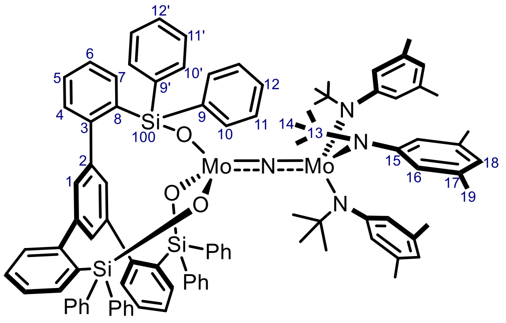

^{1}H NMR spectrum of complex 13a: [D$_6$]-benzene, 298 K, 600 MHz.

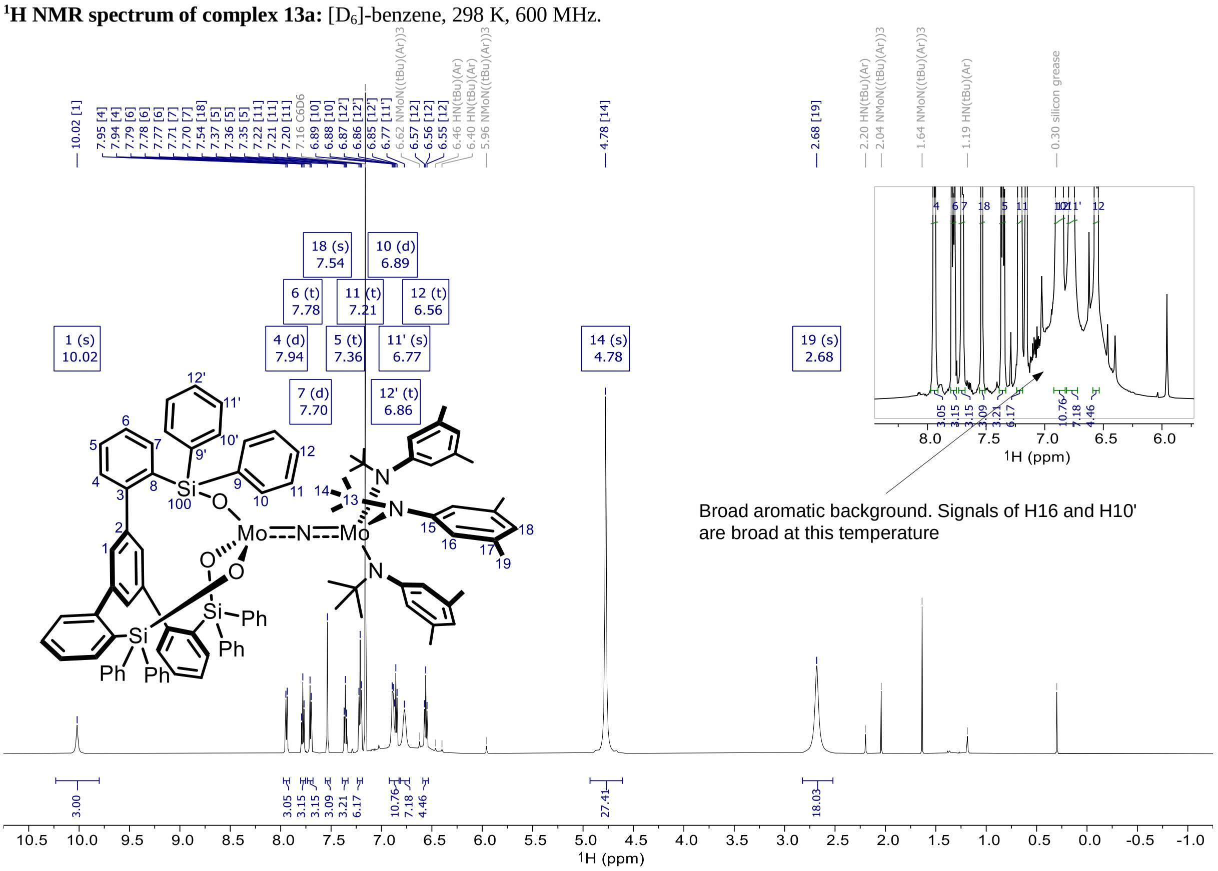

^{13}C NMR spectrum of complex 13a: [D$_6$]-benzene, 298 K, 151 MHz.

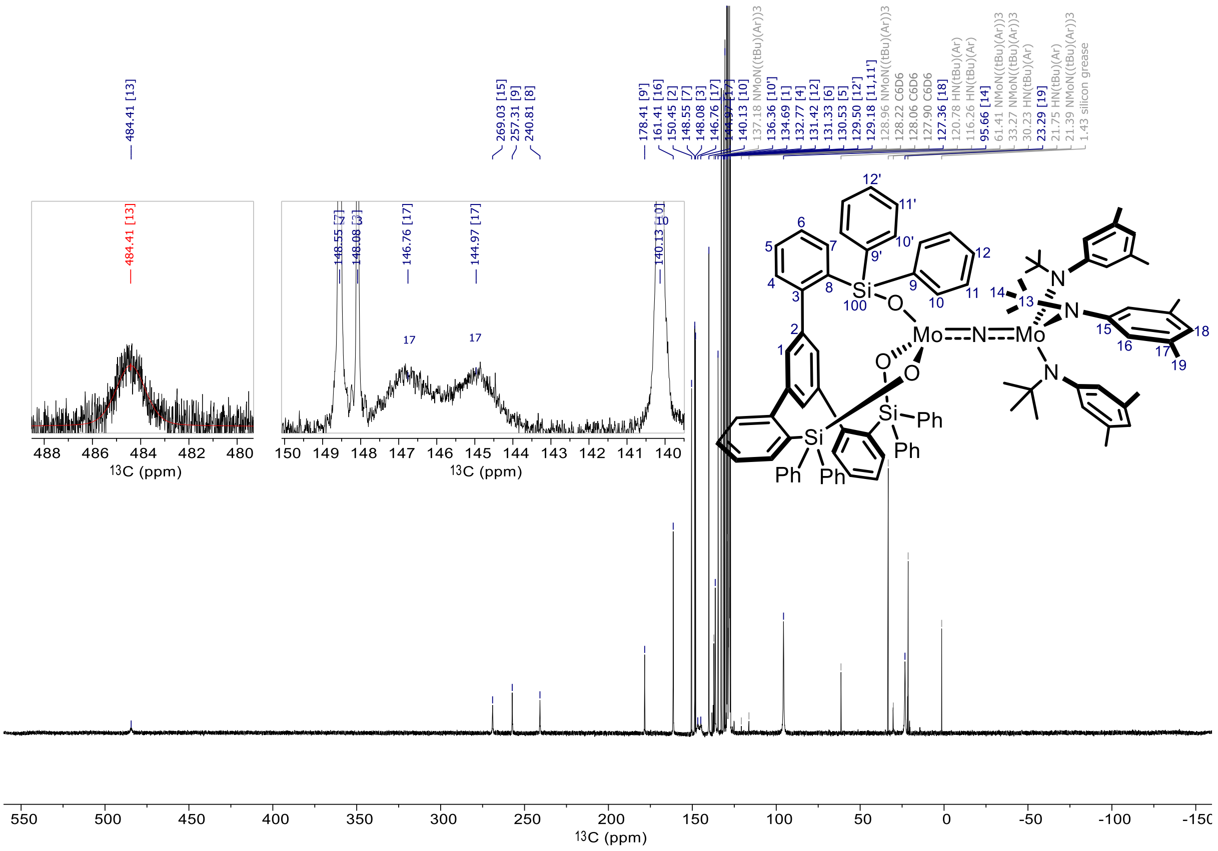

^{1}H-^{13}C HSQC NMR spectrum of complex 13a: [D$_6$]-benzene, 298 K, 600 MHz, 151 MHz.

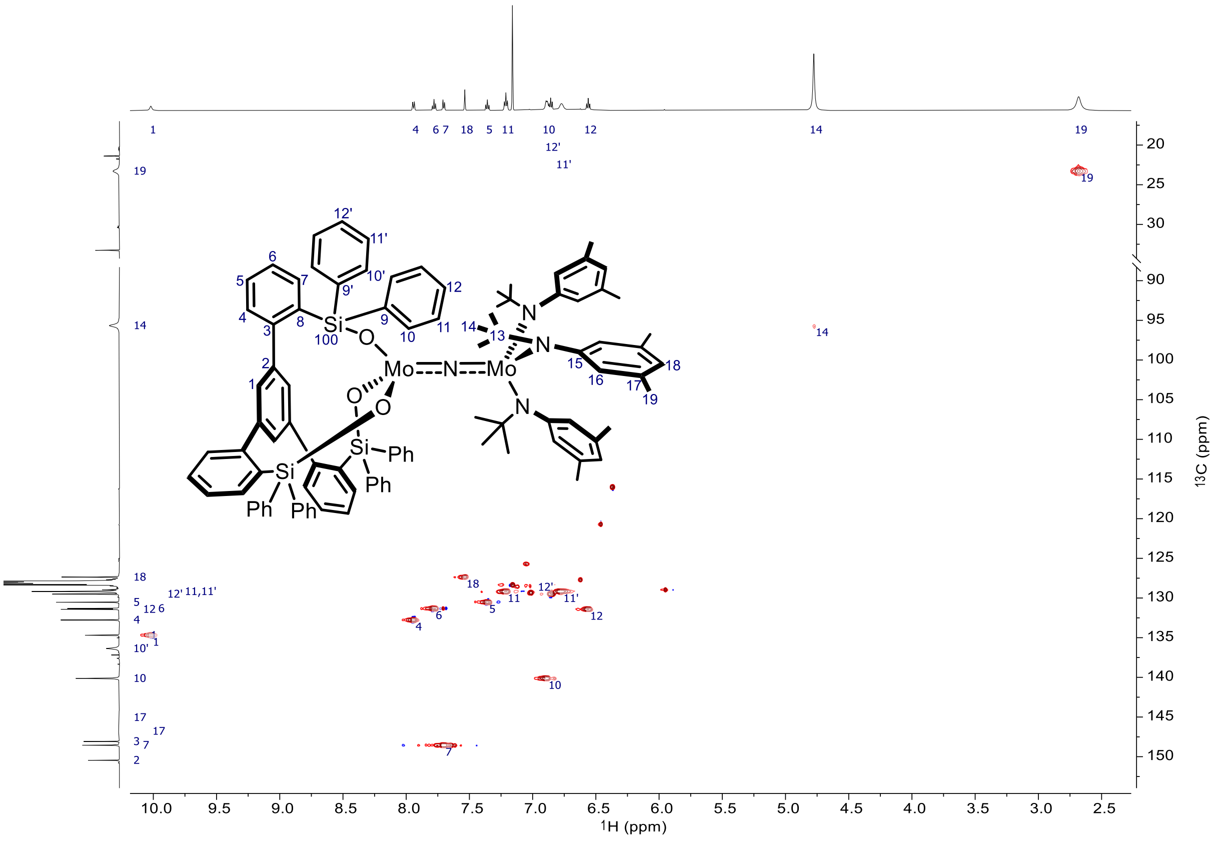

^{1}H-^{13}C HMBC NMR spectrum of complex 13a: [D$_6$]-benzene, 298 K, 600 MHz, 151 MHz.

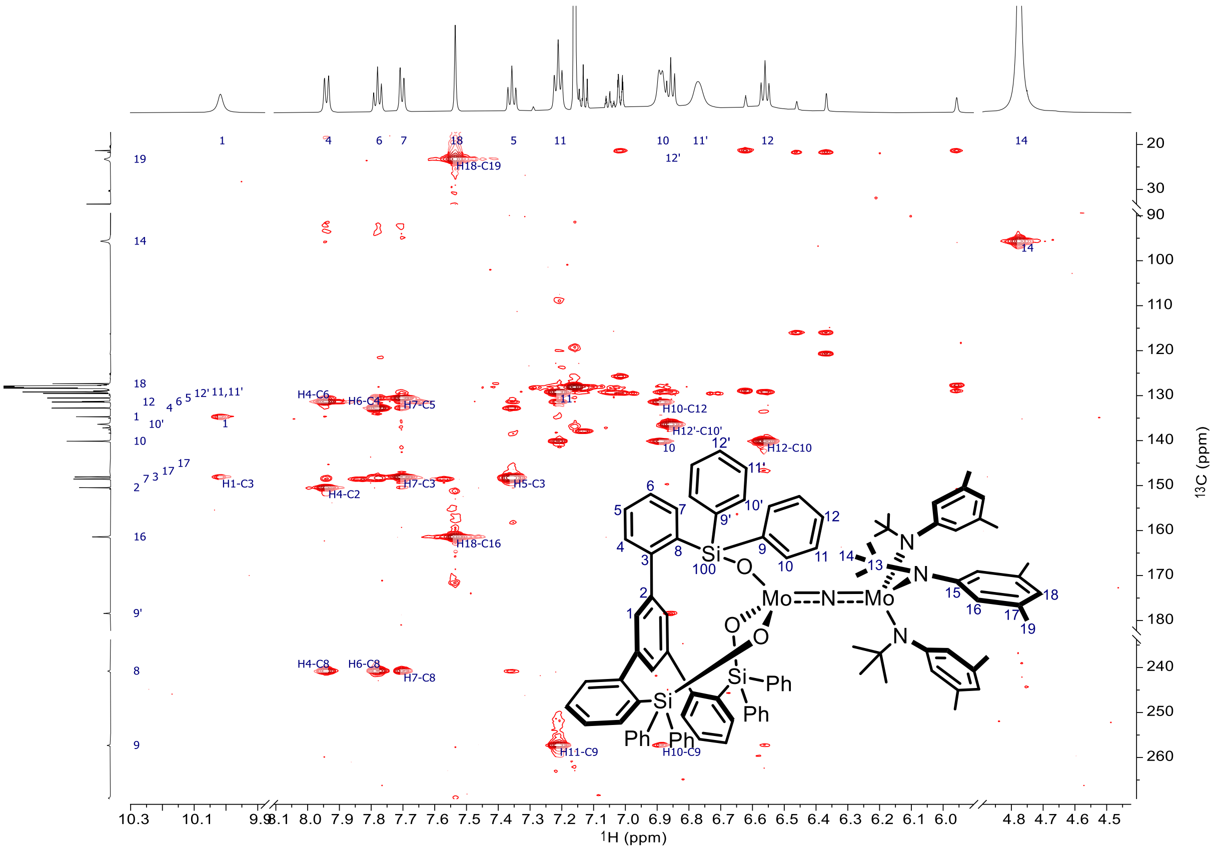

^{1}H-^{1}H COSY NMR spectrum of complex 13a: [D$_6$]-benzene, 298 K, 600 MHz, 600 MHz.

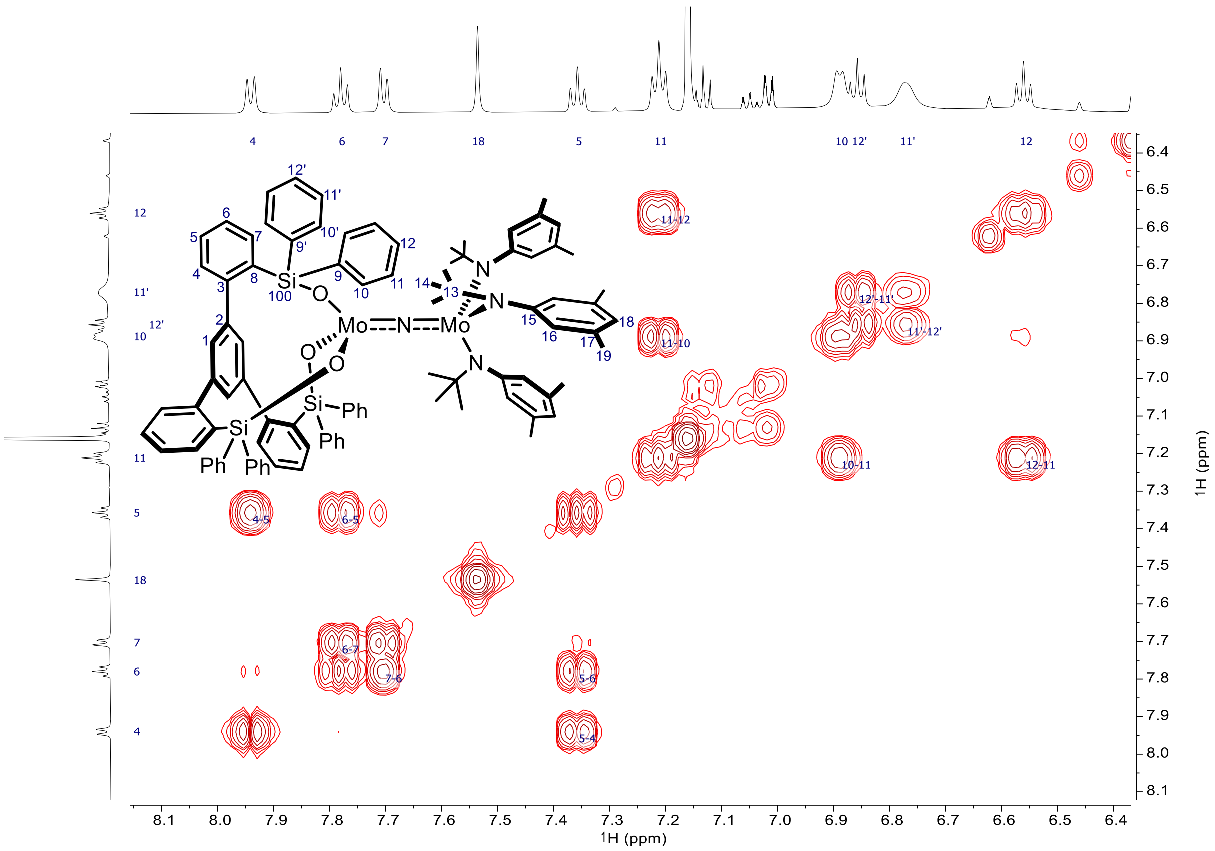

^{1}H-^{1}H ROESY NMR spectrum of complex 13a: [D$_6$]-benzene, 298 K, 600 MHz, 600 MHz.

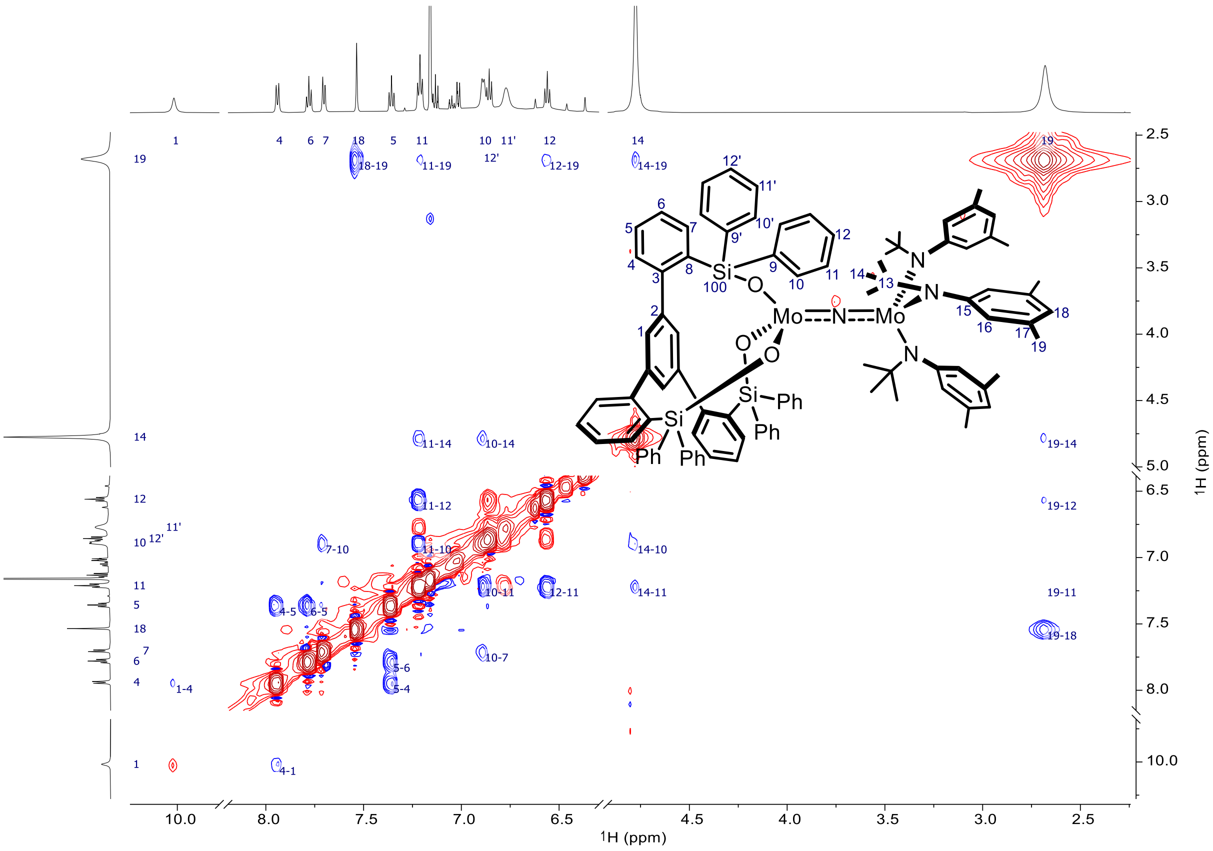

^{29}Si NMR spectrum of complex 13a: [D$_6$]-benzene, 298 K, 119 MHz and **^{1}H-^{29}Si HMBC NMR spectrum:** [D$_6$]-benzene, 298 K, 600 MHz, 119 MHz.

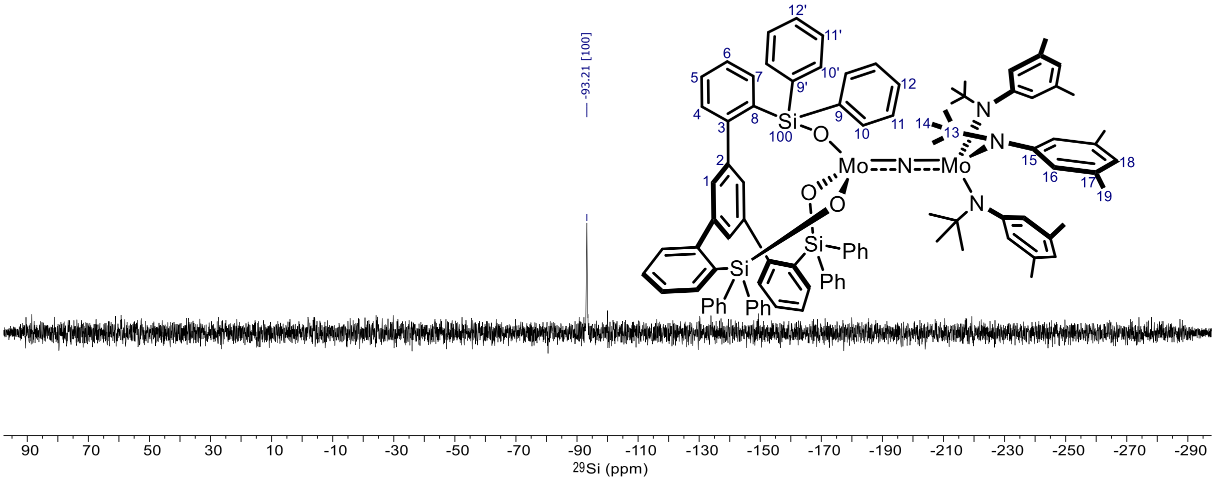

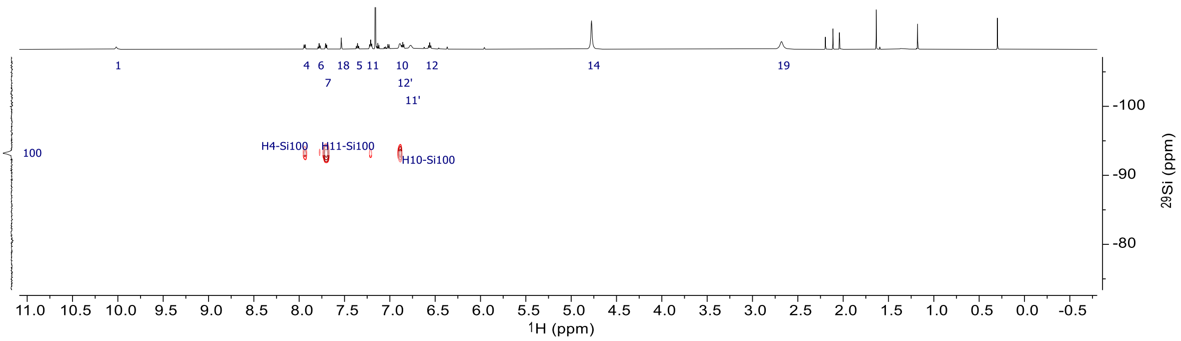

Temperature-dependence of the ^{1}H NMR spectra of complex 13a, selected region: [D$_6$]-benzene, 283 K−343 K, 600 MHz.

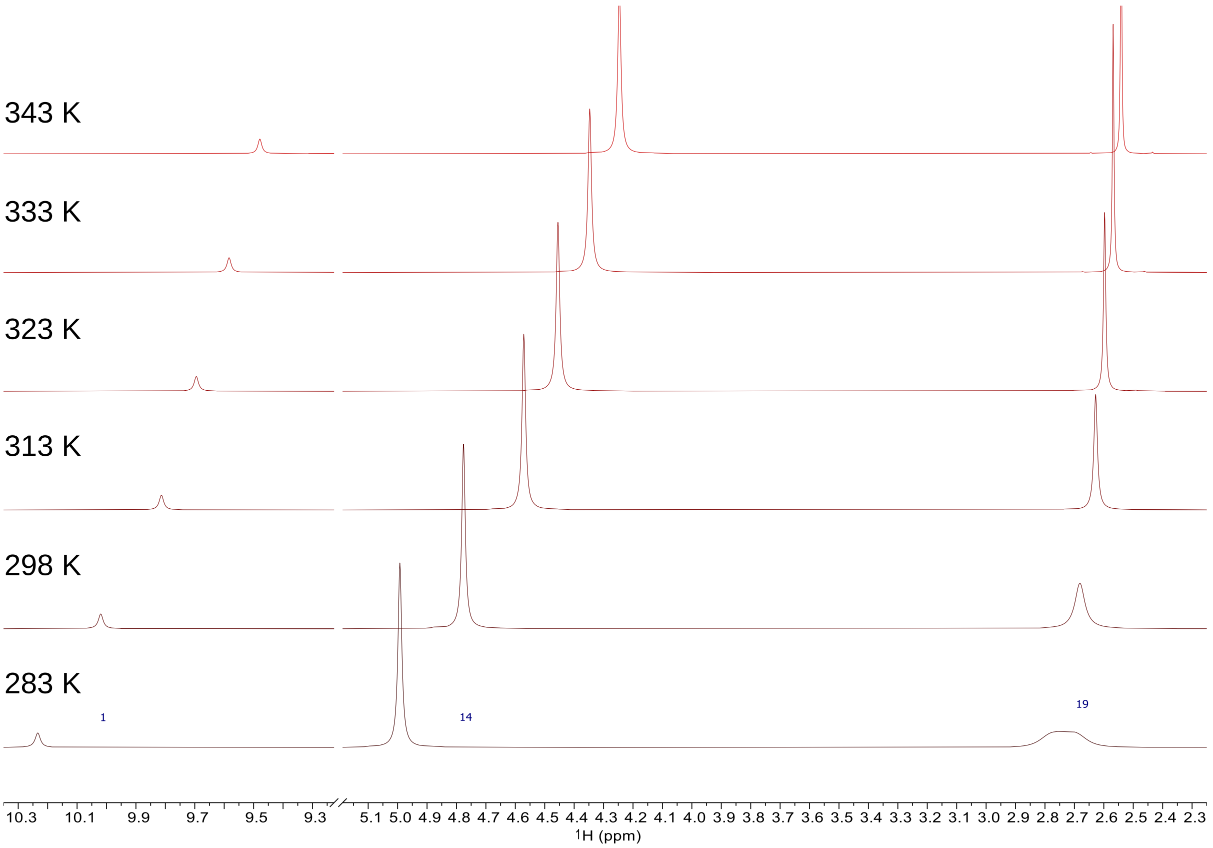

Temperature-dependence of the ^{1}H NMR spectra of complex 13a, selected region: [D$_6$]-benzene, 283 K−343 K, 600 MHz.

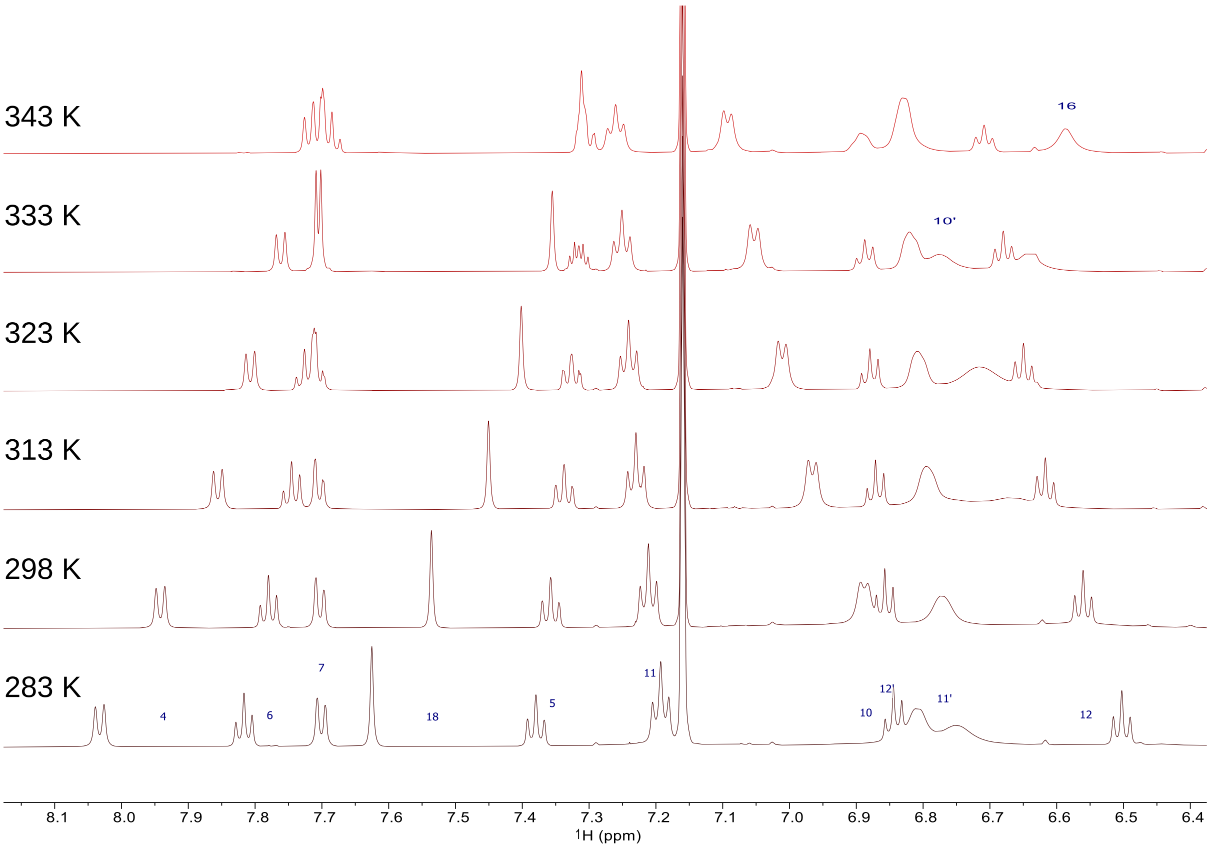

Temperature-dependence of the ^{13}C NMR spectra of complex 13a, selected region: [D$_6$]-benzene, 298 K and 333 K, 600 MHz.

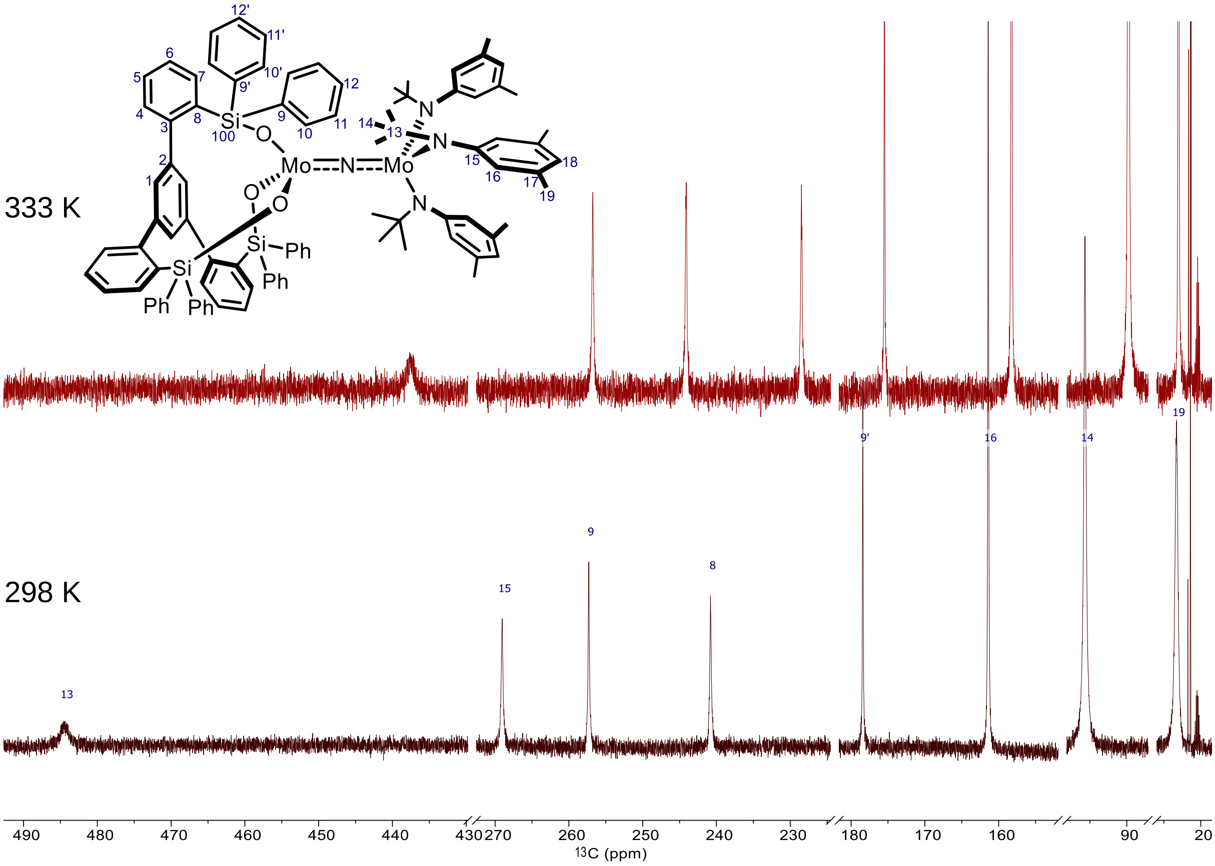

Temperature-dependence of the ^{13}C NMR spectra of complex 13a, selected region: [D$_6$]-benzene, 298 K and 333 K, 600 MHz.

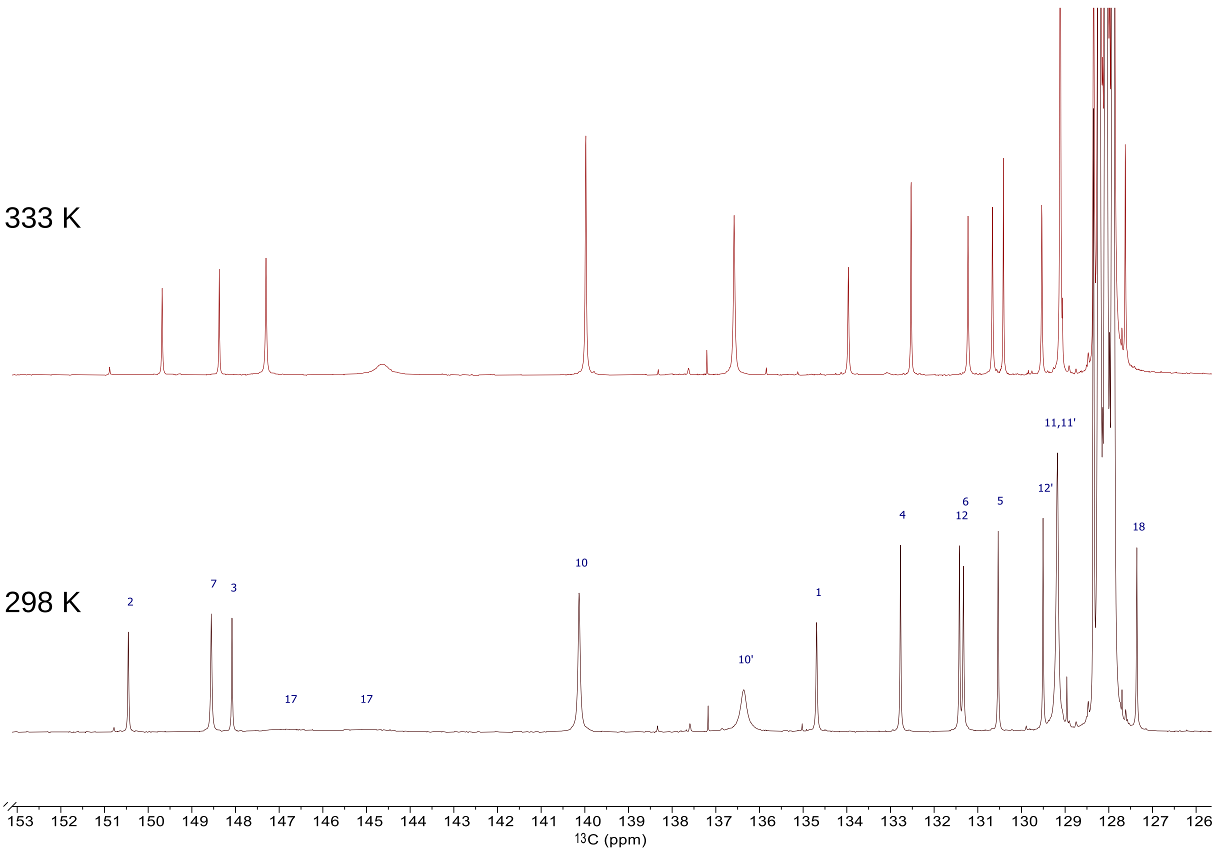

NMR analysis of complex 13b:

Most of the characteristic signals of the tripodal ligand backbone could be assigned. Si100 and C8 show a strong paramagnetic chemical shift compared to the normal chemical shift range. H1 is strongly shifted in the ^{1}H NMR spectrum. The signals of the amide ligands also show a rather large temperature dependence (see VT data), which is typical for paramagnetic species. Additionally, the signals are broadened due to a hindered rotation of the nitrogen-aryl-bond. The lateral aryl groups (H10, H10’, H12, H13, H13’ and H15; H15’, H17, H18, H18’) are diastereotopic. Additionally, the signals of these aryl groups are broadened due to a hindered rotation around the silicon-aryl-bond. Because of the paramagnetism and the hindered rotation a full assignment of all signals was not possible. Due to the high sensitivity of complex **13b**, ^{1}H and ^{13}C NMR spectra showed small amounts of free aniline HN(*t*Bu)(Ar) and terminal nitrido complex [N≡Mo(N(*t*Bu)(Ar))$_3$] (**10**) (Ar = 3,5-dimethylphenyl).

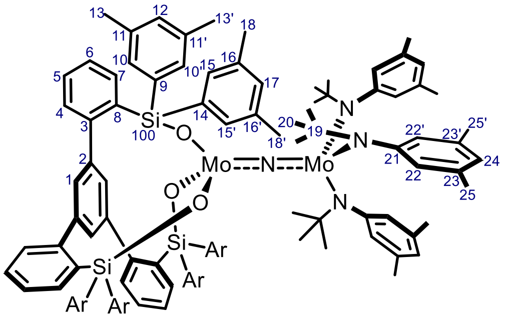

^{1}H NMR spectrum of complex 13b: [D_6]-benzene, 283 K, 600 MHz.

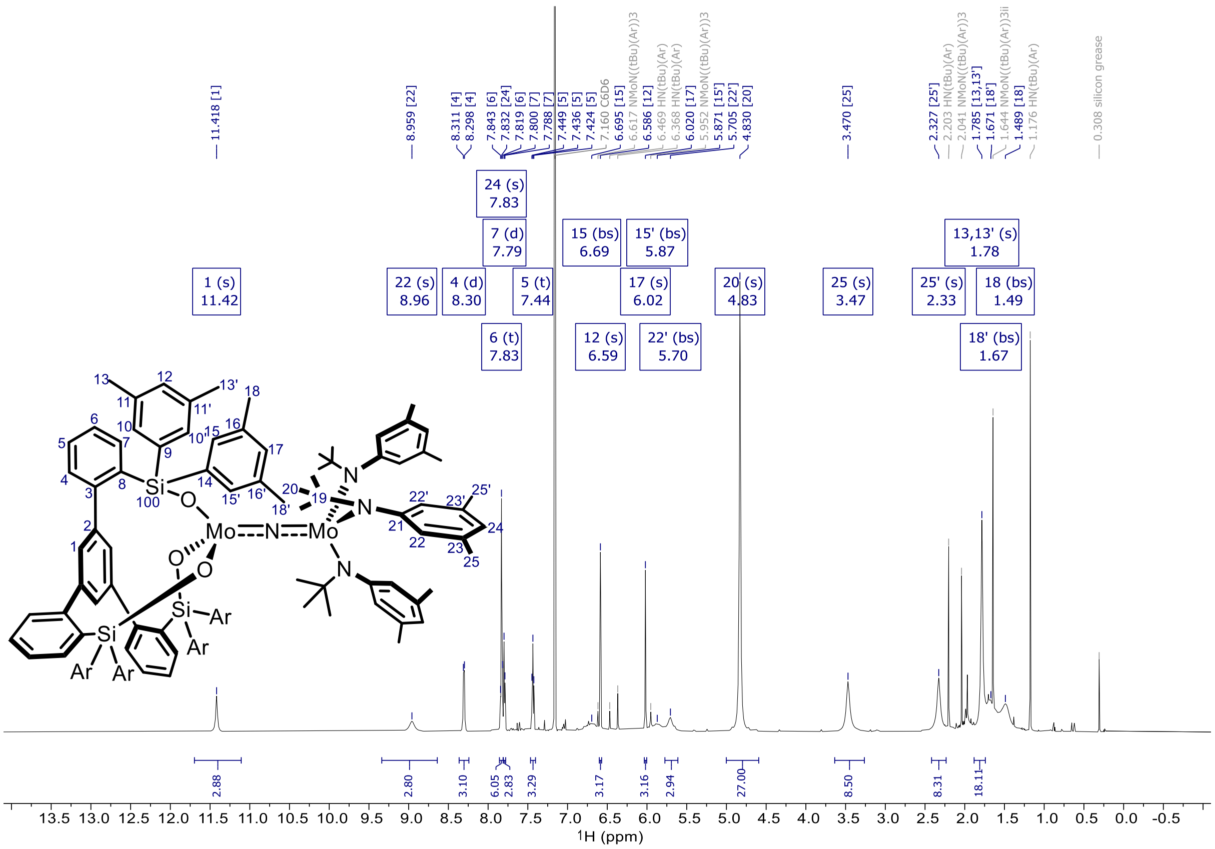

^{13}C NMR spectrum of complex 13b: [D_6]-benzene, 283 K, 151 MHz.

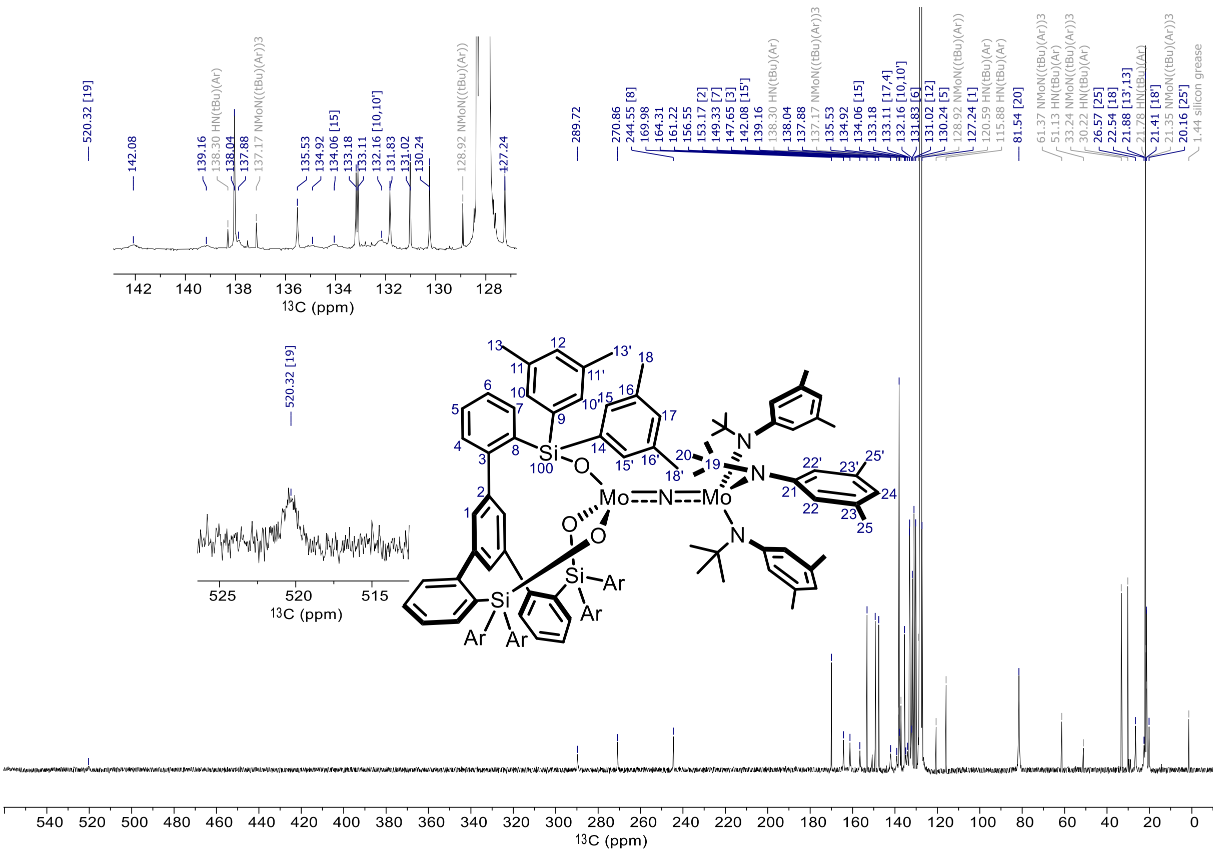

^{1}H-^{13}C HSQC NMR spectrum of complex 13b: [D$_6$]-benzene, 283 K, 600 MHz, 151 MHz.

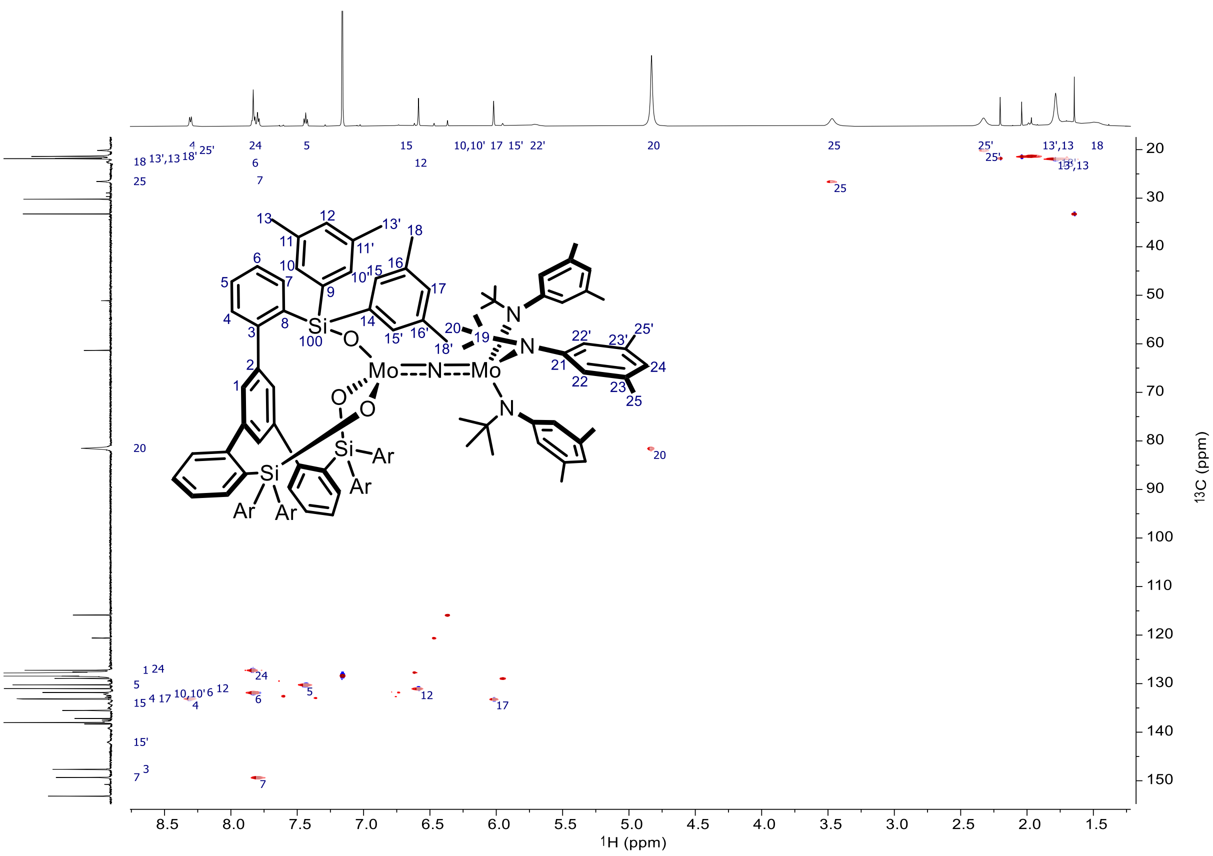

^{1}H-^{13}C HMBC NMR spectrum of complex 13b: [D$_6$]-benzene, 283 K, 600 MHz, 151 MHz.

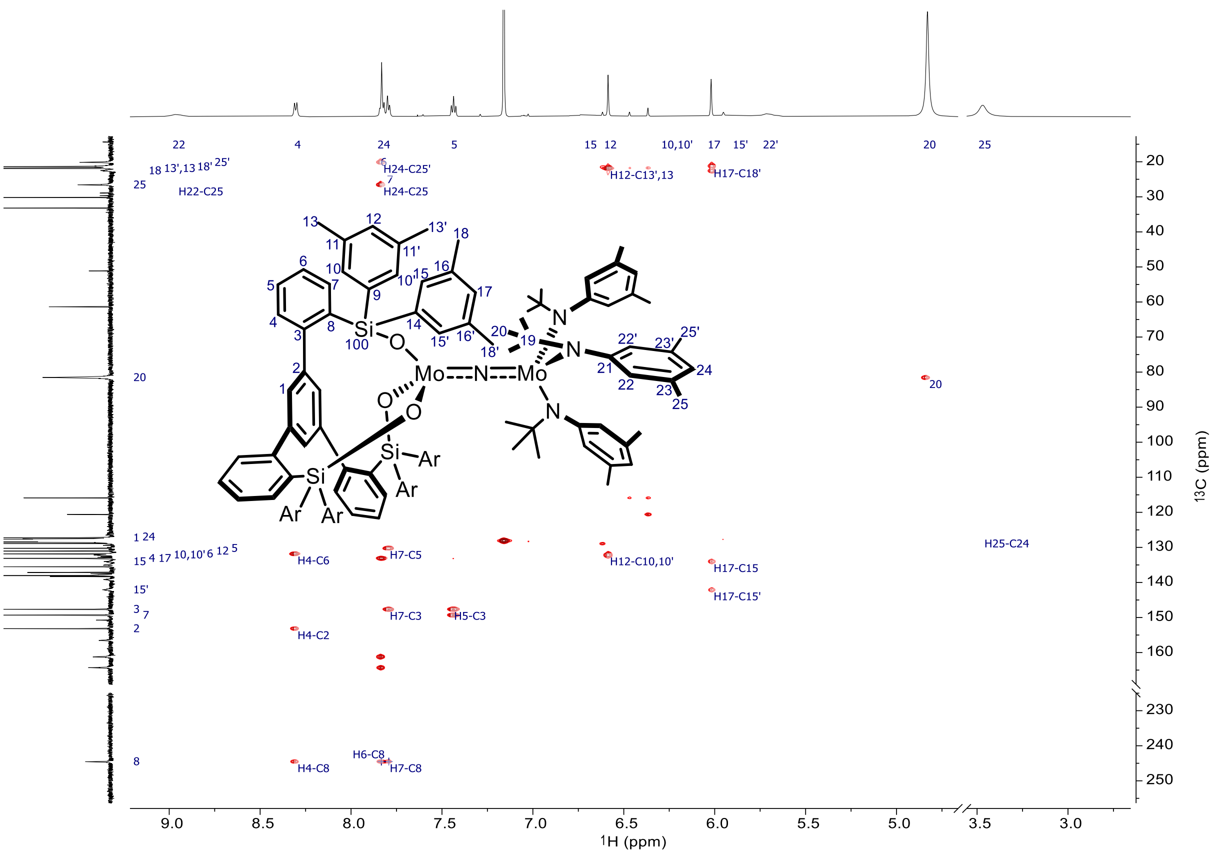

^{1}H-^{1}H COSY NMR spectrum of complex 13b: [D$_6$]-benzene, 283 K, 600 MHz, 600 MHz.

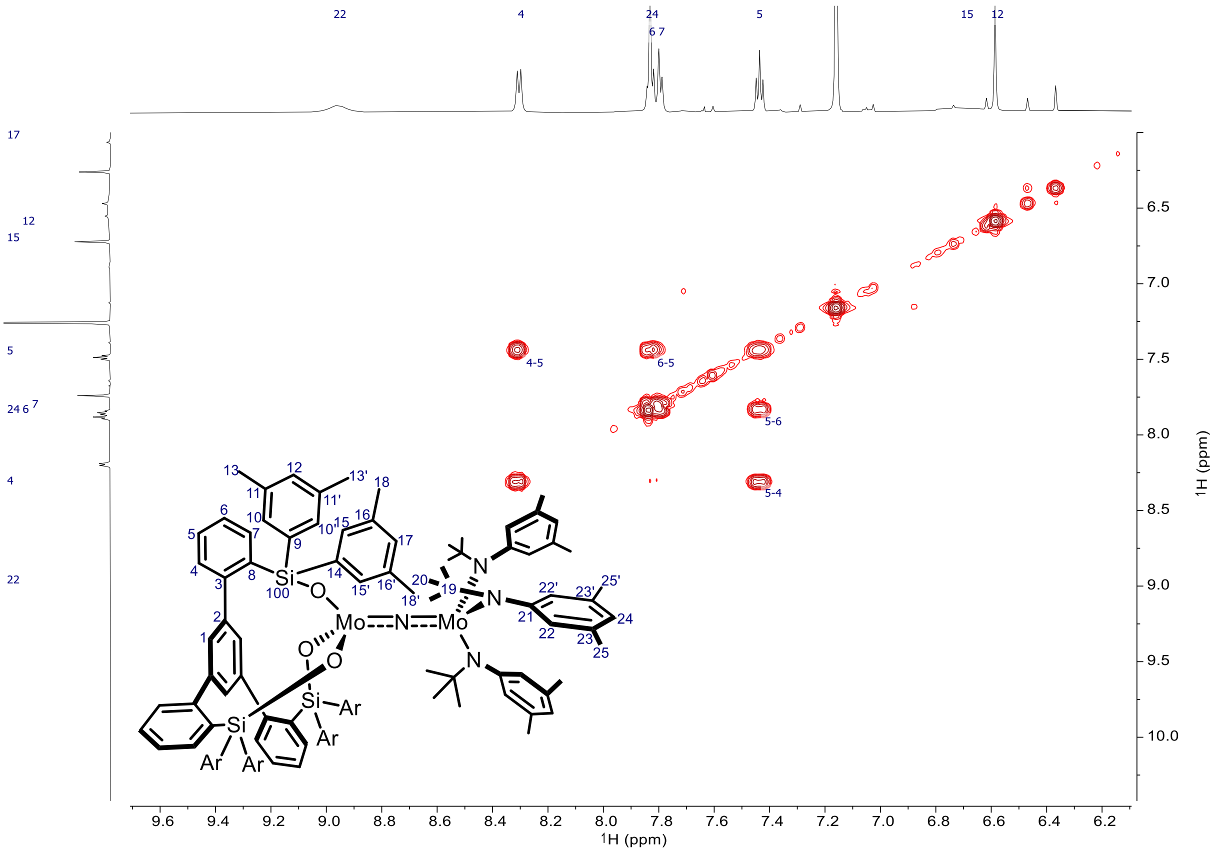

^{1}H-^{1}H ROESY NMR spectrum of complex 13b: [D$_6$]-benzene, 298 K, 600 MHz, 600 MHz.

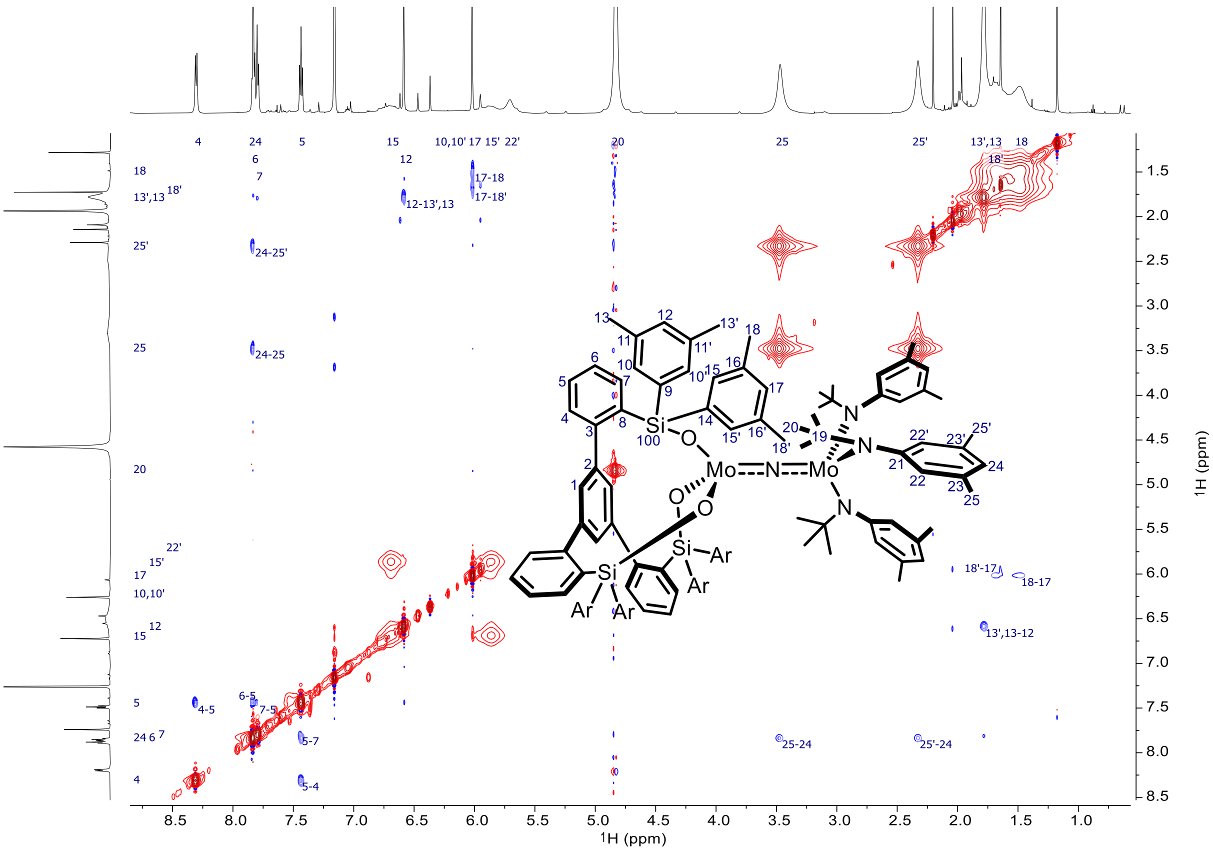

^{1}H-^{29}Si HMBC NMR spectrum of complex 13b: [D_6]-benzene, 283 K, 600 MHz, 119 MHz.

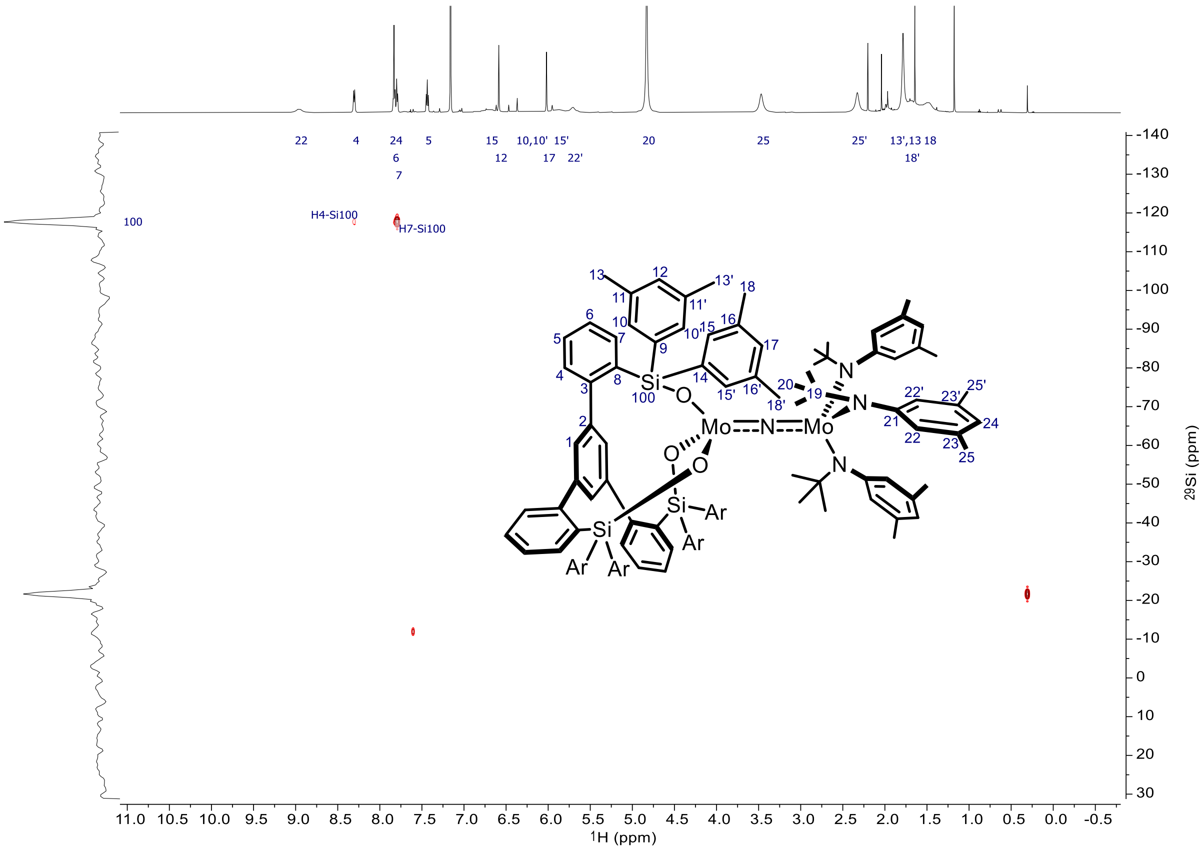

Temperature-dependence of the ^{1}H NMR spectra of complex 13b, selected region: [D_6]-benzene, 283 K−343 K, 600 MHz.

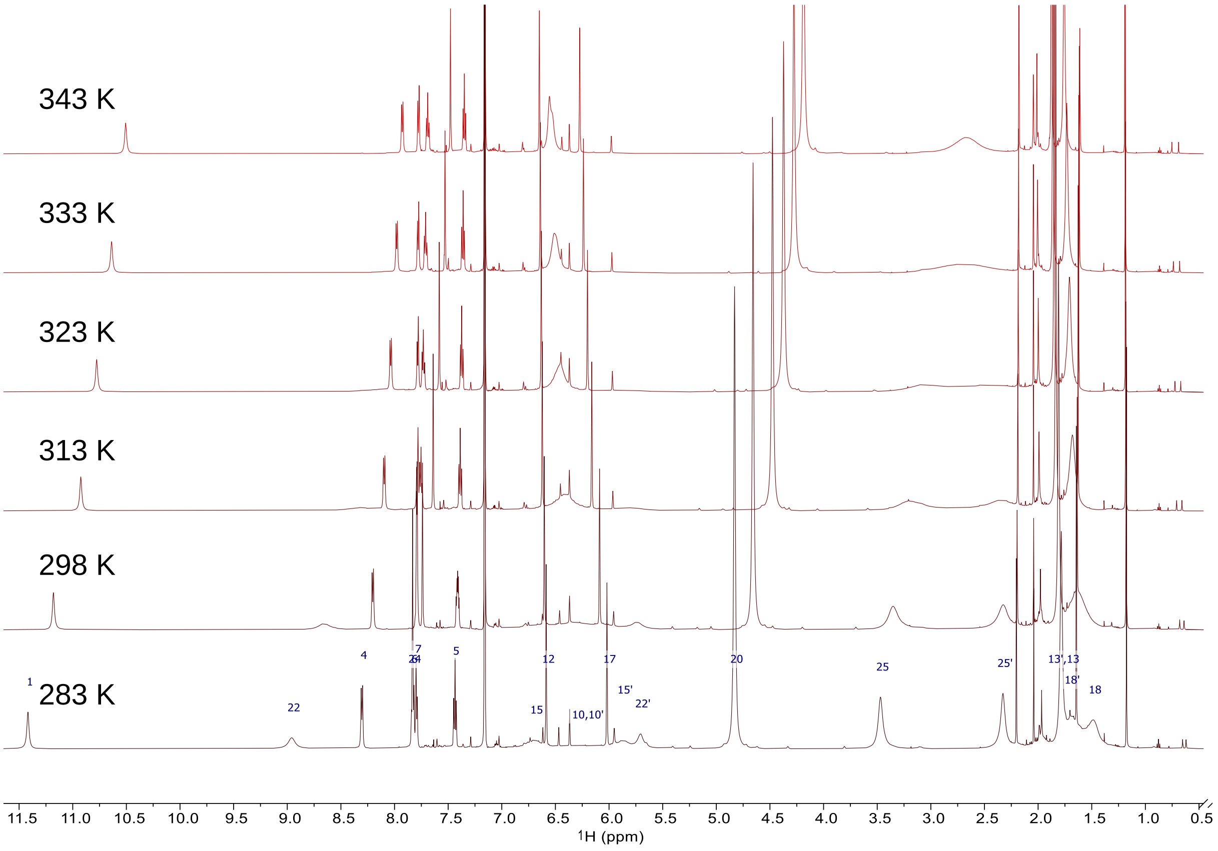

Supplement: Supplementary file 2 — ic4c00762_si_002.pdf [file ic4c00762_si_002.pdf]
