# Supplementary material for: Molybdenum(VI) Nitrido Complexes with Tripodal Silanolate Ligands. Structure and Electronic Character of an Unsymmetrical Dimolybdenum μ-Nitrido Complex Formed by Incomplete Nitrogen Atom Transfer
Source: Inorg Chem. 2024 Apr 25;63(18):8376–89. doi: 10.1021/acs.inorgchem.4c00762 (PMC11080062; doi:10.1021/acs.inorgchem.4c00762)
Supplement: Supplementary file 3 — ic4c00762_si_003.pdf [file ic4c00762_si_003.pdf]

SUPPORTING INFORMATION

Part III

COMPUTATIONAL DATA

Molybdenum(VI) Nitrido Complexes with Tripodal Silanolate Ligands.

Structure and Electronic Character of an Unsymmetrical Dimolybdenum μ-Nitrido Complex Formed by Incomplete Nitrogen Atom Transfer

Daniel Rütter, Maurice van Gastel, Markus Leutzsch, Nils Nöthling, Daniel SantaLucia,[+]

Frank Neese,* and Alois Fürstner*

Max-Planck-Institut für Kohlenforschung, 45470 Mülheim/Ruhr, Germany

[+] *Max-Planck-Institut für Chemische Energiekonversion, 45470 Mülheim/Ruhr, Germany*

Email: fuerstner@kofo.mpg.de; neese@kofo.mpg.de

DFT calculations

The electronic structure of **13**a and its electronic properties, was analyzed using DFT calculations using the B3LYP functional[1, 2] and the def2-TZVP basis set[3] and effective core potentials[4] at the molybdenum atoms.[5] All calculations were carried out with ORCA version 5.0.4.[6-9] The sets of unrestricted natural orbitals and localized quasi-restricted orbitals were constructed using the available functionality within ORCA with the UNO, qro and %loc keywords, respectively. The structures were fully optimized without constraints and confirmed to be minima on the potential energy surface through frequency calculations that showed only one negative modes at –9 cm^{-1} which was considered to be numerical noise. For calculation of the UVVIS spectrum, a TDDFT calculation was carried out using 400 roots. For calculation of the EPR g values,[10] the all-electron basis set x2c-tzvpall[11, 12] and the auxiliary basis set x2c/j[11, 12] was used at the def2-TZVP optimized structure.

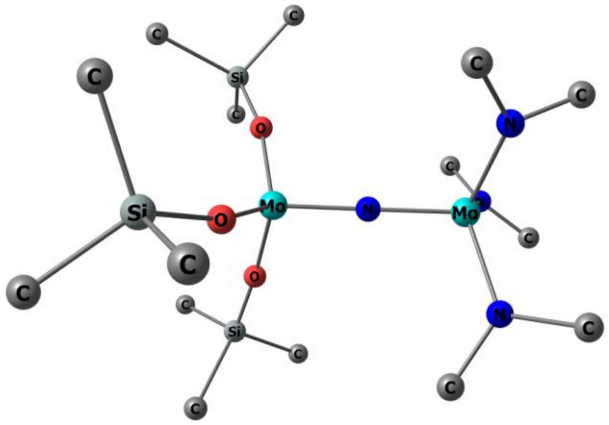

Figure S1. Computed geometric structure of **13a**; the ligand periphery was truncated for clarity.

Table S1. Selected bond distances [Å] and angles [°] of complex **13a**: comparison of the data from the X-ray crystal structure and the geometry optimized computed structure (B3LYP functional, Def2-TZVP basis set).

	SC-XRD	Optimized structure
d($Mo^{[O]}$–μN)	1.838	1.842
d($Mo^{[N]}$–μN)	1.808	1.780
d($Mo^{[O]}$–O)	1.910	1.907
	1.912	1.915
	1.920	1.927
d($Mo^{[N]}$–N)	1.970	1.978
	1.966	1.968
	1.968	1.970
∠($Mo^{[N]}$–μN –$Mo^{[O]}$)	178.6	178.1
∠(μN –$Mo^{[O]}$–O)	102.7	101.9
	102.9	100.3
	102.3	103.0
∠(μN –$Mo^{[N]}$–N)	103.4	105.4
	104.6	105.0
	105.5	105.2

For completeness, an MO scheme using (non-localized) quasi-restricted orbitals is depicted in Figure S2. As expected, the two frontier molecular orbitals concern Mo-centered orbitals at –3.00 eV and –3.55 eV containing the 3 unpaired electrons. Below these, starting at –5.6 eV a plethora of ligand orbitals are found, involving either the ligating nitrogen and oxygen atoms, but also the phenyl moieties.

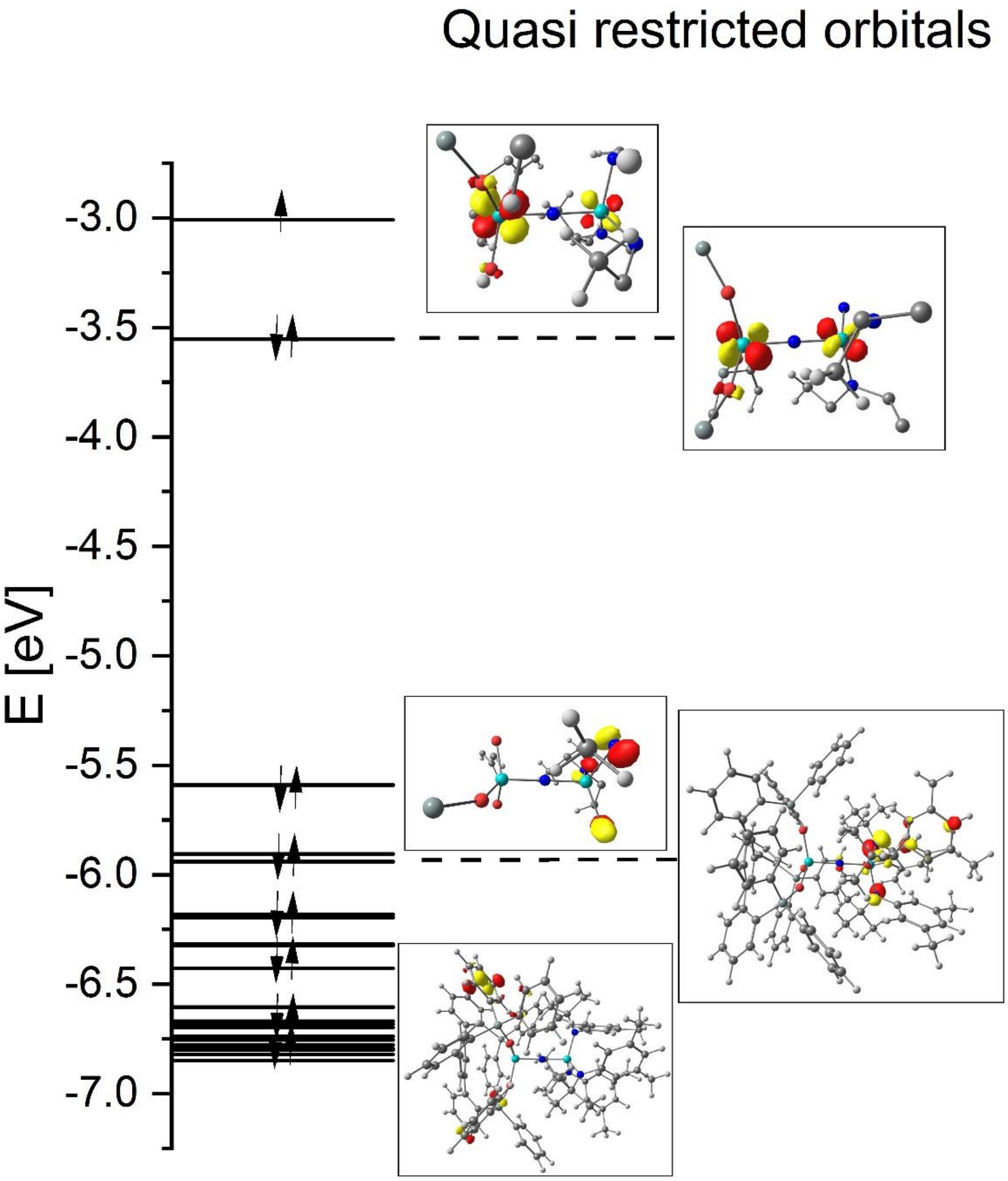

Figure S2. Molecular orbital scheme of **13a** including the frontier orbitals and the closest ligand orbitals. The structures have been truncated in the miniatures with the orbital contours for the purpose of clarity.

Def2-TZVP optimized coordinates [Å] of 13a

Mo	3.45586604664560	14.45721486589789	5.09046206209477
Mo	5.57901159156839	11.52404374112488	5.11317006873042
Si	8.86875144850438	12.70292045413374	4.72979871996492
Si	4.80347250106503	9.48649350468064	2.38319204850919
Si	5.19948629472491	9.93648872517479	8.17412160792142
O	5.43932022616038	10.99377536950186	6.94834458465146
O	7.30248002974735	12.26881628017402	4.68025286170564
O	4.88350115551632	10.51078544230666	3.65547284736519
N	3.61953091481247	15.17700232759893	3.26376972975143
N	4.47656906061412	12.99924946195447	5.11183382479362
N	4.13704728418030	15.59758805354526	6.55569163903166
N	1.63587111949035	13.80924573793881	5.46529475726391
C	3.62608522831284	16.60678206055411	3.27171671894458
C	3.89369313967196	14.50642750696165	1.95733411920439
C	9.07671087639900	14.45008909957510	4.06647876367679
C	6.18788092465487	9.88751295502185	1.19017435627986
C	3.14874678224857	16.42197996965031	7.17342263546953
C	2.43043753361854	17.31095129432856	3.15998272457415
H	1.50692419388322	16.75907388549244	3.05561728752782
C	9.69482892843718	10.18927274693945	3.49467652304075
C	0.14680808266866	14.36616690214194	3.58691233894206
H	0.52746525831281	13.50683705290425	3.05579034201566
C	6.86356986374876	9.34436332588345	8.84190793931612
C	2.91011916312956	17.70951049647613	6.70198330558455
H	3.47098008066324	18.06524960623636	5.84891127589423
C	6.87705019786591	8.13952685069439	5.95628657701473
H	6.16914294784943	7.60877474591261	6.57192726570567
C	5.40679150347946	14.42628683253676	1.74433176900517
H	5.84700043228149	15.41960779800106	1.66969006841125
H	5.64139435508390	13.87721200034649	0.82981197767233
H	5.86906284888119	13.91068346823444	2.58454329947777
C	8.80063510813710	9.55750937602083	5.74267285781121
H	9.58417197013521	10.14325441165655	6.19764888082229
C	6.78223302747597	8.03174455257164	4.56898785426828
C	2.41685523258908	15.95313315117355	8.27004983704438
H	2.58108825908257	14.94208274921662	8.61445864443216
C	3.16562475551180	9.74264879447347	1.50182369226471
C	7.05004011296039	9.34398473929242	10.23067818383438
H	6.22143197161333	9.60325718728352	10.87598778705908
C	0.63322691580432	14.64317999423792	4.87091133344802
C	1.46704945571148	16.75106744602103	8.89520293491377
C	4.11486502124060	8.53335331793117	7.57097597036552
C	-0.78861715235479	15.18910278309173	2.97706316677994
C	7.87267844366686	8.90769445964118	6.55632059636188
C	3.24014621951092	15.27689915164151	0.80344927493177
H	2.16367929350545	15.36540886603197	0.95763405580638
H	3.40988788437217	14.73331584914671	-0.12775805189189
H	3.65537020856908	16.27863076690019	0.69413137401813
C	9.89662476411216	11.58073326587798	3.60931865080698
C	5.47537634903697	15.55639122460500	7.23285023395631
C	0.14483481873738	15.75607570126715	5.54775395191922
H	0.51049370240931	15.96559553527423	6.54174507189506
C	8.62301023904156	14.76582156276891	2.78042824966128
H	8.17759161807123	13.99516755983440	2.16527854826257
C	9.20456388080056	8.72864005796054	8.62379882658006
H	10.04569157860672	8.47798235268016	7.98963815139126
C	7.96912106046290	9.00157008450791	8.03243364520913
C	6.94471739145799	11.05571967079915	1.31943615747991
H	6.73958909232275	11.74200808697671	2.12850709742422
C	8.72452803341266	9.47033879518912	4.35465306842089
C	8.58378737161285	12.49900816419516	7.57161232245381
H	7.52612307883676	12.38980609270353	7.38230349748462
C	9.47173535940740	12.65880495299605	6.50384893617340
C	4.82425267750965	17.31613264902924	3.42159356378951
H	5.74828175281276	16.76639380909667	3.53040656399923
C	-0.80967136877831	16.59346280850998	4.96283672774513
C	1.17919522923783	12.50689011803602	6.04717115911063
C	3.31585361440586	13.09635424357273	1.97733182131747
H	3.73434290766025	12.49998037635509	2.78085022384467
H	3.54703645206836	12.59641859542834	1.03858659388209
H	2.23374805075748	13.11682527271328	2.08864356025926
C	2.41517338142632	18.70742749279113	3.18361622414477
C	-1.26043948359061	16.30296890109724	3.68034340335854
H	-1.99267110011632	16.95347940428411	3.21422947675007

C	8.29216495531284	10.44857069702496	-0.58121867520136
H	9.11423540118590	10.65845876758814	-1.25416026421161
C	7.72348195091340	8.68838678989602	3.77903139707002
H	7.68041070125388	8.59571045439882	2.70553391025893
C	5.71574933709832	7.19834654075456	3.96279975888433
C	10.85675103227502	12.16935248087399	2.77633346657875
H	11.02668063204534	13.23659303237218	2.83579344497030
C	9.65175337002847	15.47628150164640	4.82203488058569
H	10.00806040884115	15.26904484803673	5.82317069105613
C	11.58227734394887	11.42990061753972	1.84928653304520
H	12.31496814537434	11.91905663922741	1.21928031847761
C	6.56306064718677	15.35432144756376	6.18163212125513
H	6.40201903364966	14.45344622197107	5.59821690705716
H	7.53308251173733	15.26573364982780	6.66890359290668
H	6.60162128159729	16.20350102243570	5.50028417858240
C	5.50521315314880	14.41728072547963	8.25023664060838
H	4.75448819601637	14.56925785433983	9.02440994042482
H	6.48288263886230	14.35861326106613	8.73335167006207
H	5.30431270121039	13.46480827678788	7.76468718236944
C	7.98907445772858	11.33421689821904	0.44618239576800
H	8.58283545017267	12.22876012667830	0.58166522838752
C	1.25919698639682	18.04587287588193	8.41069344402534
H	0.51620931720432	18.67739150864917	8.88680087678786
C	4.84722595985507	7.69244046309544	2.96612966796096
C	3.09706395834355	10.07826523285051	0.14620211137410
H	4.00910746731802	10.16864864380199	-0.43048745996429
C	4.86711022921580	11.90304068279969	10.22395261364419
H	5.85054268744882	12.24653129727596	9.93171297603475
C	6.50786400115367	9.00598945736184	0.15064416028669
H	5.94858340899578	8.08380797551370	0.03492788598797
C	9.29225542920972	17.06283540908529	3.04461901620358
H	9.37017900414131	18.06974411035620	2.65328293519677
C	9.76131706289958	16.76934013580814	4.32007497361969
H	10.20534604945685	17.54866484421141	4.92751252736186
C	10.40865012213729	9.45634610467561	2.54504661477016
H	10.23062010075521	8.39142792495481	2.46090866478883
C	8.27718938012483	9.04770157683415	10.81257064749097
H	8.38309451960366	9.05952803343952	11.89028176687534
C	10.39767096842012	12.54664707371896	9.15387818516131
H	10.75499101985863	12.49372452366478	10.17492364444580
C	7.54767235543319	9.28200498256502	-0.73005115028940
H	7.78378819767323	8.58494797596088	-1.52502372136328
C	1.97369261588636	18.53857119142272	7.32266077122707
C	4.30003817322130	10.79609987243562	9.58163626964937
C	9.36475299133801	8.75325769260059	10.00178474749774
H	10.33232668741260	8.53608066875478	10.43706813306351
C	5.76425729209981	16.88425267657708	7.94701923642398
H	5.71457328293796	17.72261552996063	7.25089791602134
H	6.77281091708225	16.84248510782119	8.36191374767705
H	5.06805466398899	17.07286863896944	8.76385159151013
C	9.03826316759877	12.44014091190478	8.88327552343790
H	8.33405915380396	12.28992914142477	9.69169735549093
C	0.66273157672748	16.22745282099496	10.05569263352315
H	-0.40795399512397	16.26514613115195	9.83928357806542
H	0.83078756433801	16.82473024950484	10.95562482263205
H	0.92611876970324	15.19378835382725	10.28126820134774
C	3.61885322199383	19.38668098190631	3.34047235850883
H	3.61511568166320	20.47086910666945	3.37571384617984
C	11.34696406541674	10.06721629112431	1.72493898219221
H	11.89111725128234	9.47987356262816	0.99563769853241
C	-1.25477238467970	14.91363467775794	1.57230839263923
H	-0.78889466319501	15.60766739297585	0.86643114610253
H	-2.33612202569791	15.03551125804243	1.48038148771934
H	-0.99362700979411	13.90170348232164	1.26127118739270
C	3.39170943532553	8.62993828067522	6.37945924929929
H	3.44781405669184	9.52949542900037	5.78287541411621
C	1.87641587850601	10.32203813536030	-0.47578997827867
H	1.84854209744019	10.58617114761716	-1.52593215003045
C	1.11291771914480	19.44953079364586	3.04527664678217
H	0.62289136855138	19.20505749840016	2.09965201617860
H	1.26618755221021	20.52822143330688	3.07971170443884
H	0.41924509504066	19.17648122770763	3.84231775395804
C	10.83743358733048	12.75914766195588	6.79484342389419
H	11.55397732782800	12.86767046386854	5.98763262456886
C	4.83401202621244	18.70358968319616	3.46646201449139
C	1.96359130492755	9.64598380481880	2.21161760937349
H	1.98076712754438	9.38285612483771	3.26091966344655
C	3.03235790692213	10.38014483562161	10.00169057497730

H	2.56435352941379	9.52722218579007	9.52677262719963
C	8.72072368366290	16.05508161118659	2.27318612955266
H	8.34837358347819	16.27566491027839	1.28031999426011
C	0.69550159772427	10.23949966025727	0.25344934052772
H	-0.25545849639951	10.43919299678874	-0.22529830507385
C	3.89456274010965	6.81124801381329	2.43754176990877
H	3.21074244937068	7.16560606239802	1.67764390771070
C	-1.33073239568912	17.78736095853322	5.71627076030033
H	-0.54061207538433	18.52213045890154	5.88030129503048
H	-1.70024886163176	17.49821330031955	6.70253507827236
H	-2.14213314519282	18.27322229632130	5.17377407167795
C	5.57783351864239	5.88409786269337	4.41457140507833
H	6.24376660773758	5.52246596284647	5.18799489512034
C	11.29929625459215	12.70715514676208	8.10551024423616
H	12.36059209248222	12.78414598944545	8.30893397421585
C	4.02075006639067	7.34285833026873	8.30141270104805
H	4.57835184858783	7.23286786564116	9.22530746553601
C	3.77829535049337	5.49777201903284	2.87663501640350
H	3.02811144436095	4.84619343988686	2.44564305800432
C	4.18959222711781	12.58338962304277	11.22734701394923
H	4.64441862794886	13.44646269514792	11.69791272895682
C	1.77459388361065	19.95147614725267	6.84278008376158
H	2.51944791067807	20.61753909945014	7.28864725801685
H	0.78972171340031	20.33185022693799	7.11691549052076
H	1.88706878030372	20.01979525503774	5.76135565553760
C	4.61852487246708	5.03601100592882	3.88021071735414
H	4.53174226635057	4.02036062859882	4.24571805080069
C	0.74139842262080	9.89749352456749	1.60199926211388
H	-0.17398790879696	9.83237193417830	2.17749649851897
C	6.12315897256997	19.45796635103268	3.65683564282384
H	6.05199146862340	20.15161734465265	4.49813823077874
H	6.36831469384502	20.04981208750257	2.77060158341834
H	6.95212768656884	18.77647052468694	3.84581939926514
C	2.92442943134802	12.15858740346154	11.62285653529801
H	2.39246985482851	12.68691646259249	12.40465540639021
C	2.61069041818598	7.57583634497939	5.92215079739512
H	2.08058662569300	7.66394950189965	4.98271124565950
C	3.23734083137697	6.28542844302646	7.85333053663355
H	3.18350423563551	5.36959601019587	8.42935410234729
C	2.34927431795968	11.05027547162637	11.01151174708449
H	1.36620482740471	10.71157904307487	11.31524163717057
C	2.53280160159017	6.39986927559854	6.65841744333255
H	1.93637779799349	5.57147007857631	6.29725477493738
C	-0.26791562245185	12.61081007266329	6.54531619271859
H	-0.37000301887239	13.41244496870921	7.27830618426472
H	-0.54481109430034	11.67056912925592	7.02537706068000
H	-0.96962355219198	12.79813601515209	5.73264590658418
C	2.06518706115035	12.16386362545414	7.24282915894786
H	3.11353323506792	12.11525513607645	6.96535170121796
H	1.77871202225951	11.19395665919098	7.64775904814968
H	1.95431249162802	12.90664936904647	8.03284013298516
C	1.28630647326274	11.40344260929960	4.99340376984070
H	0.62182448691438	11.59584137347583	4.15228094704637
H	1.02030050970680	10.43486672368517	5.42220687854997
H	2.30448746713084	11.34640241650279	4.61372457581365

References

1. Becke, A. D. Density-Functional Exchange-Energy Approximation with Correct Asymptotic-Behavior. *Phys. Rev. A* **1988**, *38*, 3098-3100.
2. Lee, C. T.; Yang, W. T.; Parr, R. G. Development of the Colle-Salvetti Correlation-Energy Formula into a Functional of the Electron-Density. *Phys. Rev. B* **1988**, *37*, 785-789.
3. Weigend, F.; Ahlrichs, R. Balanced Basis Sets of Split Valence, Triple Zeta Valence and Quadruple Zeta Valence Quality for H to Rn: Design and Assessment of Accuracy. *Phys. Chem. Chem. Phys.* **2005**, *7*, 3297-3305.
4. Turbomole GmbH *Turbomole, Available from www.Turbomole.Com*, Turbomole GmbH: Mannheim, 2014.
5. Andrae, D.; Haussermann, U.; Dolg, M.; Stoll, H.; Preuss, H. Energy-Adjusted Abinitio Pseudopotentials for the 2nd and 3rd Row Transition-Elements. *Theoretica Chimica Acta* **1990**, *77*, 123-141.
6. Neese, F. *Orca, an Ab Initio, DFT and Semiempirical SCF-Mo Package*, 3.0.2 R4787; MPI for Chemical Energy Conversion, 2014.
7. Neese, F. Software Update: The Orca Program System, Version 4.0. *Wiley Interdiscip. Rev.: Comput. Mol. Sci.* **2017**, *2*, e1327.
8. Neese, F. The Orca Program System. *Wiley Interdiscip. Rev.: Comput. Mol. Sci.* **2012**, *2*, 73-78.
9. Neese, F. Software Update: The Orca Program System-Version 5.0. *Wiley Interdiscip. Rev.: Comput. Mol. Sci.* **2022**, *12*.
10. Neese, F. Prediction of Electron Paramagnetic Resonance G Values Using Coupled Perturbed Hartree-Fock and Kohn-Sham Theory. *J. Chem. Phys.* **2001**, *115*, 11080-11096.
11. Pollak, P.; Weigend, F. Segmented Contracted Error-Consistent Basis Sets of Double- and Triple-Z Valence Quality for One- and Two-Component Relativistic All-Electron Calculations. *J. Chem. Theory Comput.* **2017**, *13*, 3696-3705.
12. Franzke, Y. J.; Tress, R.; Pazdera, T. M.; Weigend, F. Error-Consistent Segmented Contracted All-Electron Relativistic Basis Sets of Double- and Triple-Zeta Quality for Nmr Shielding Constants. *Phys. Chem. Chem. Phys.* **2019**, *21*, 16658-16664.
