# Supplementary material for: Molybdenum(VI) Nitrido Complexes with Tripodal Silanolate Ligands. Structure and Electronic Character of an Unsymmetrical Dimolybdenum μ-Nitrido Complex Formed by Incomplete Nitrogen Atom Transfer
Source: Inorg Chem. 2024 Apr 25;63(18):8376–89. doi: 10.1021/acs.inorgchem.4c00762 (PMC11080062; doi:10.1021/acs.inorgchem.4c00762)
Supplement: Supplementary file 4 — ic4c00762_si_004.pdf [file ic4c00762_si_004.pdf]

SUPPORTING INFORMATION

Part IV

CRYSTALLOGRAPHIC DATA

Molybdenum(VI) Nitrido Complexes with Tripodal Silanolate Ligands.

Structure and Electronic Character of an Unsymmetrical Dimolybdenum μ-Nitrido Complex Formed by Incomplete Nitrogen Atom Transfer

Daniel Rütter, Maurice van Gastel, Markus Leutzsch, Nils Nöthling, Daniel SantaLucia,[+]

Frank Neese,* and Alois Fürstner*

Max-Planck-Institut für Kohlenforschung, 45470 Mülheim/Ruhr, Germany
[+] *Max-Planck-Institut für Chemische Energiekonversion, 45470 Mülheim/Ruhr, Germany*

Email: fuerstner@kofo.mpg.de; neese@kofo.mpg.de

Table of Contents

Single crystal structure analysis of silane S2 2
Single crystal structure analysis of ligand 7e hydrate · hexamethyldisiloxane solvate 12
Single crystal structure analysis of complex 3d 25
Single crystal structure analysis of complex 3e · diethyl ether solvate 33
Single crystal structure analysis of complex 8 43
Single crystal structure analysis of complex 13a · benzene/pentane solvate 51
Single crystal structure analysis of complex 13b · pentane/toluene solvate 66

Single crystal structure analysis of silane S2

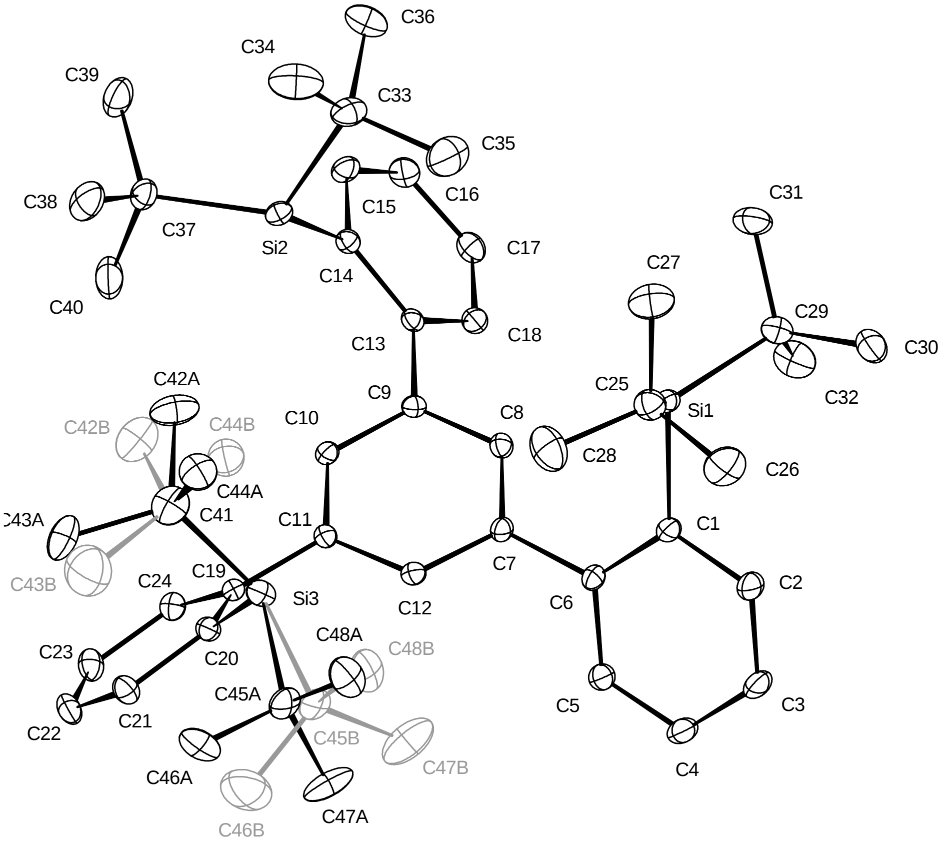

Figure S 1. The molecular structure of silane **S2**; H atoms have been removed for clarity. Main structure shown in black and disordered parts shown in grey.

X-ray Crystal Structure Analysis of Silane S2:

C_{48} H_{72} Si_3, M_r = 733.32 g mol^{-1}, colourless block, crystal size 0.224 x 0.134 x 0.101 mm^3, Triclinic, space group *P*-1 [2], *a* = 13.1547(10) Å, b = 13.1703(10) Å, *c* = 16.8177(13) Å, α = 67.763(4)°, β = 74.469(4)°, γ = 60.595(3)°, *V* = 2337.4(3) Å^3, *T* = 100(2) K, *Z* = 2, D_{calc} = 1.042 g·cm^3, λ = 0.71073 Å, $\mu(Mo\text{-}K_\alpha)$ = 0.131 mm^{-1}, Gaussian absorption correction (T_{min} = 0.971, T_{max} = 0.987), Bruker AXS D8-Venture diffractometer with IµS Diamond Mo-anode X-ray source and PHOTON III detector, 1.857 < θ < 30.508°, 193651 measured reflections, 14245 independent reflections, 12995 reflections with $I > 2\sigma(I)$, R_{int} = 0.0480. The structure was solved by *SHELXT* and refined by full-matrix least-squares (*SHELXL*) against F^2 to R_1 = 0.0519 [$I > 2\sigma(I)$], wR_2 = 0.1132 [all data], 563 parameters and 38 restraints.

Full .cif data for the compound are available under the CCDC number **CCDC-2265450**

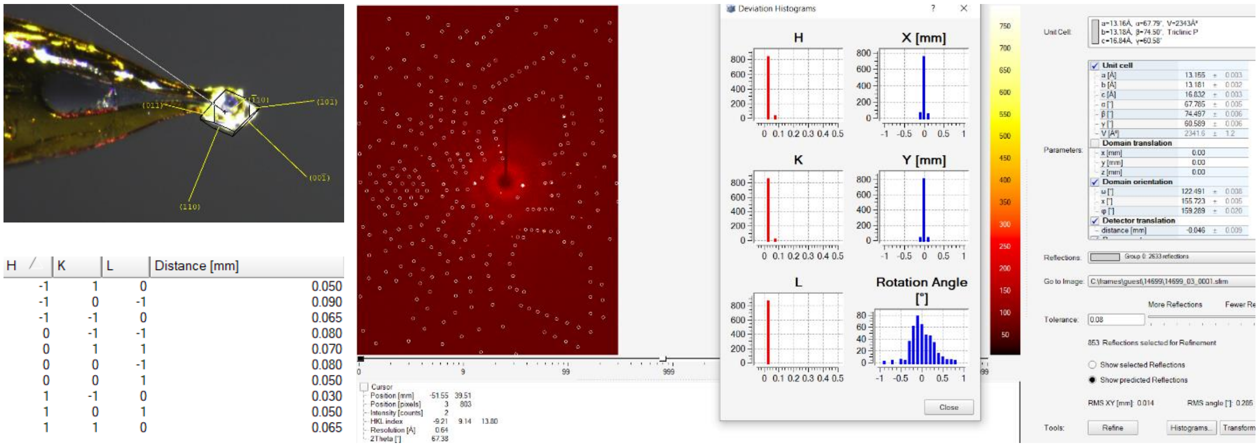

Figure S 2. Crystal faces and unit cell determination/refinement of silane **S2**.

INTENSITY STATISTICS FOR DATASET

Resolution	#Data	#Theory	%Complete	Redundancy	Mean I	Mean I/s	Rmerge	Rsigma
Inf - 2.77	226	229	98.7	18.90	93.16	119.22	0.0215	0.0116
2.77 - 1.86	524	524	100.0	20.19	40.17	101.84	0.0211	0.0071
1.86 - 1.48	752	752	100.0	18.61	18.92	74.13	0.0291	0.0099
1.48 - 1.29	773	773	100.0	19.33	18.86	69.29	0.0295	0.0103
1.29 - 1.17	788	788	100.0	18.92	14.41	59.83	0.0361	0.0123
1.17 - 1.09	699	699	100.0	18.42	11.86	51.08	0.0436	0.0144
1.09 - 1.02	837	837	100.0	17.96	9.66	44.25	0.0546	0.0172
1.02 - 0.97	770	770	100.0	15.88	7.81	36.45	0.0641	0.0210
0.97 - 0.93	720	720	100.0	13.63	6.65	29.30	0.0745	0.0260
0.93 - 0.89	839	839	100.0	12.78	6.26	26.92	0.0808	0.0285
0.89 - 0.86	792	792	100.0	11.95	6.49	26.14	0.0800	0.0293
0.86 - 0.84	529	529	100.0	11.48	5.70	22.73	0.0910	0.0335
0.84 - 0.81	956	956	100.0	11.15	5.06	20.30	0.1011	0.0378
0.81 - 0.79	740	740	100.0	10.65	4.24	17.39	0.1156	0.0441
0.79 - 0.77	774	774	100.0	10.48	3.68	15.78	0.1397	0.0512
0.77 - 0.75	901	901	100.0	10.24	3.43	14.42	0.1500	0.0556
0.75 - 0.74	448	448	100.0	9.91	3.56	14.15	0.1554	0.0562
0.74 - 0.72	1078	1078	100.0	8.50	3.24	11.83	0.1720	0.0690
0.72 - 0.71	535	535	100.0	8.43	2.95	10.72	0.1910	0.0758
0.71 - 0.70	564	564	100.0	8.33	2.89	10.64	0.1958	0.0768
0.70 - 0.69	713	794	89.8	7.09	2.55	9.04	0.2217	0.0950
0.79 - 0.69	5013	5094	98.4	8.99	3.20	12.46	0.1676	0.0664
Inf - 0.69	14958	15042	99.4	13.25	9.82	34.01	0.0482	0.0213

A resolution cut off (SHEL 999 0.7) was applied to exclude poorly determined reflections at high diffraction angles. Both ^{t}Bu groups at Si3 show twofold positional disorders. They were described with fixed occupancies of 60:40% and the ISOR instruction was used to treat thermal ellipsoids. The position of the H atom on Si3 is also affected by the disorder. It was located in the residual density map and freely refined with fixed occupancies of 60:40% (corresponding to the occupancies found for the C atoms). The DFIX command was used to hold the H atoms in position for the final refinement cycles.

Table S 1. Crystal data and structure refinement of silane **S2**.

Identification code	14699	
Empirical formula	$C_{48} H_{72} Si_3$	
Color	colourless	
Formula weight	733.32 g·mol^{-1}	
Temperature	100(2) K	
Wavelength	0.71073 Å	
Crystal system	Triclinic	
Space group	*P*-1, (no. 2)	
Unit cell dimensions	a = 13.1547(10) Å	α= 67.763(4)°.
	b = 13.1703(10) Å	β= 74.469(4)°.
	c = 16.8177(13) Å	γ = 60.595(3)°.
Volume	2337.4(3) Å^3	
Z	2	
Density (calculated)	1.042 Mg·m^{-3}	
Absorption coefficient	0.131 mm^{-1}	
F(000)	804 e	
Crystal size	0.224 x 0.134 x 0.101 mm^3	
θ range for data collection	1.857 to 30.508°.	
Index ranges	-18 ≤ h ≤ 18, -18≤ k ≤ 18, -24 ≤ l ≤ 24	
Reflections collected	193651	
Independent reflections	14245 [R_{int} = 0.0480]	
Reflections with I>2σ(I)	12995	
Completeness to θ = 25.242°	100.0 %	
Absorption correction	Gaussian	
Max. and min. transmission	0.987 and 0.971	
Refinement method	Full-matrix least-squares on F^2	
Data / restraints / parameters	14245 / 38 / 563	
Goodness-of-fit on F^2	1.172	
Final R indices [I>2σ(I)]	R_1 = 0.0519	wR^2 = 0.1106
R indices (all data)	R_1 = 0.0578	wR^2 = 0.1132
Extinction coefficient	n/a	
Largest diff. peak and hole	0.534 and -0.302 e·Å^{-3}	

Table S 2. Bond lengths [Å] and angles [°] of silane **S2**.

Si(1)-H(1)	1.384(18)	Si(1)-C(1)	1.8922(13)
Si(1)-C(25)	1.9061(15)	Si(1)-C(29)	1.9135(14)
Si(2)-H(2)	1.389(19)	Si(2)-C(14)	1.8899(13)
Si(2)-C(33)	1.9066(14)	Si(2)-C(37)	1.9131(14)
Si(3)-H(3A)	1.380(10)	Si(3)-H(3B)	1.382(10)
Si(3)-C(20)	1.8952(13)	Si(3)-C(41)	1.9074(16)
Si(3)-C(45A)	1.903(7)	Si(3)-C(45B)	1.948(10)
C(1)-C(2)	1.4107(17)	C(1)-C(6)	1.4132(17)
C(2)-H(2A)	0.9500	C(2)-C(3)	1.3906(18)
C(3)-H(3)	0.9500	C(3)-C(4)	1.3886(19)
C(4)-H(4)	0.9500	C(4)-C(5)	1.3893(18)
C(5)-H(5)	0.9500	C(5)-C(6)	1.4051(17)
C(6)-C(7)	1.4941(16)	C(7)-C(8)	1.4002(17)
C(7)-C(12)	1.3945(17)	C(8)-H(8)	0.9500
C(8)-C(9)	1.4004(16)	C(9)-C(10)	1.3964(16)
C(9)-C(13)	1.4876(16)	C(10)-H(10)	0.9500
C(10)-C(11)	1.3935(16)	C(11)-C(12)	1.3988(16)
C(11)-C(19)	1.4872(16)	C(12)-H(12)	0.9500
C(13)-C(14)	1.4187(16)	C(13)-C(18)	1.3984(17)
C(14)-C(15)	1.4056(17)	C(15)-H(15)	0.9500
C(15)-C(16)	1.3868(18)	C(16)-H(16)	0.9500
C(16)-C(17)	1.3897(19)	C(17)-H(17)	0.9500
C(17)-C(18)	1.3908(18)	C(18)-H(18)	0.9500
C(19)-C(20)	1.4151(17)	C(19)-C(24)	1.4046(17)
C(20)-C(21)	1.4070(17)	C(21)-H(21)	0.9500
C(21)-C(22)	1.3872(18)	C(22)-H(22)	0.9500
C(22)-C(23)	1.3873(19)	C(23)-H(23)	0.9500
C(23)-C(24)	1.3865(18)	C(24)-H(24)	0.9500
C(25)-C(26)	1.534(2)	C(25)-C(27)	1.535(2)
C(25)-C(28)	1.542(2)	C(26)-H(26A)	0.9800
C(26)-H(26B)	0.9800	C(26)-H(26C)	0.9800
C(27)-H(27A)	0.9800	C(27)-H(27B)	0.9800
C(27)-H(27C)	0.9800	C(28)-H(28A)	0.9800
C(28)-H(28B)	0.9800	C(28)-H(28C)	0.9800

C(29)-C(30)	1.538(2)	C(29)-C(31)	1.5405(19)
C(29)-C(32)	1.537(2)	C(30)-H(30A)	0.9800
C(30)-H(30B)	0.9800	C(30)-H(30C)	0.9800
C(31)-H(31A)	0.9800	C(31)-H(31B)	0.9800
C(31)-H(31C)	0.9800	C(32)-H(32A)	0.9800
C(32)-H(32B)	0.9800	C(32)-H(32C)	0.9800
C(33)-C(34)	1.540(2)	C(33)-C(35)	1.541(2)
C(33)-C(36)	1.533(2)	C(34)-H(34A)	0.9800
C(34)-H(34B)	0.9800	C(34)-H(34C)	0.9800
C(35)-H(35A)	0.9800	C(35)-H(35B)	0.9800
C(35)-H(35C)	0.9800	C(36)-H(36A)	0.9800
C(36)-H(36B)	0.9800	C(36)-H(36C)	0.9800
C(37)-C(38)	1.541(2)	C(37)-C(39)	1.534(2)
C(37)-C(40)	1.535(2)	C(38)-H(38A)	0.9800
C(38)-H(38B)	0.9800	C(38)-H(38C)	0.9800
C(39)-H(39A)	0.9800	C(39)-H(39B)	0.9800
C(39)-H(39C)	0.9800	C(40)-H(40A)	0.9800
C(40)-H(40B)	0.9800	C(40)-H(40C)	0.9800
C(41)-C(42A)	1.613(3)	C(41)-C(42B)	1.403(6)
C(41)-C(43A)	1.542(3)	C(41)-C(43B)	1.593(6)
C(41)-C(44A)	1.519(4)	C(41)-C(44B)	1.569(6)
C(42A)-H(42A)	0.9800	C(42A)-H(42B)	0.9800
C(42A)-H(42C)	0.9800	C(42B)-H(42D)	0.9800
C(42B)-H(42E)	0.9800	C(42B)-H(42F)	0.9800
C(43A)-H(43B)	0.9800	C(43A)-H(43C)	0.9800
C(43A)-H(43A)	0.9800	C(43B)-H(43E)	0.9800
C(43B)-H(43D)	0.9800	C(43B)-H(43F)	0.9800
C(44A)-H(44A)	0.9800	C(44A)-H(44B)	0.9800
C(44A)-H(44C)	0.9800	C(44B)-H(44D)	0.9800
C(44B)-H(44E)	0.9800	C(44B)-H(44F)	0.9800
C(45A)-C(46A)	1.524(5)	C(45A)-C(47A)	1.545(6)
C(45A)-C(48A)	1.594(15)	C(45B)-C(46B)	1.509(10)
C(45B)-C(47B)	1.583(9)	C(45B)-C(48B)	1.45(2)
C(46A)-H(46A)	0.9800	C(46A)-H(46B)	0.9800
C(46A)-H(46C)	0.9800	C(46B)-H(46D)	0.9800
C(46B)-H(46E)	0.9800	C(46B)-H(46F)	0.9800

C(47A)-H(47A)	0.9800	C(47A)-H(47B)	0.9800
C(47A)-H(47C)	0.9800	C(47B)-H(47D)	0.9800
C(47B)-H(47E)	0.9800	C(47B)-H(47F)	0.9800
C(48A)-H(48A)	0.9800	C(48A)-H(48B)	0.9800
C(48A)-H(48C)	0.9800	C(48B)-H(48D)	0.9800
C(48B)-H(48E)	0.9800	C(48B)-H(48F)	0.9800
C(1)-Si(1)-H(1)	110.0(8)	C(1)-Si(1)-C(25)	108.68(6)
C(1)-Si(1)-C(29)	110.73(6)	C(25)-Si(1)-H(1)	104.3(8)
C(25)-Si(1)-C(29)	116.55(6)	C(29)-Si(1)-H(1)	106.3(8)
C(14)-Si(2)-H(2)	111.0(8)	C(14)-Si(2)-C(33)	106.33(6)
C(14)-Si(2)-C(37)	111.53(6)	C(33)-Si(2)-H(2)	104.1(8)
C(33)-Si(2)-C(37)	118.14(6)	C(37)-Si(2)-H(2)	105.5(8)
C(20)-Si(3)-H(3A)	111(2)	C(20)-Si(3)-H(3B)	109(4)
C(20)-Si(3)-C(41)	106.47(6)	C(20)-Si(3)-C(45A)	111.9(2)
C(20)-Si(3)-C(45B)	111.9(3)	C(41)-Si(3)-H(3A)	108(2)
C(41)-Si(3)-H(3B)	98(3)	C(41)-Si(3)-C(45B)	122.9(3)
C(45A)-Si(3)-H(3A)	107(2)	C(45A)-Si(3)-C(41)	112.06(16)
C(45B)-Si(3)-H(3B)	107(3)	C(2)-C(1)-Si(1)	115.90(9)
C(2)-C(1)-C(6)	116.99(11)	C(6)-C(1)-Si(1)	126.88(9)
C(1)-C(2)-H(2A)	118.6	C(3)-C(2)-C(1)	122.71(12)
C(3)-C(2)-H(2A)	118.6	C(2)-C(3)-H(3)	120.2
C(4)-C(3)-C(2)	119.56(12)	C(4)-C(3)-H(3)	120.2
C(3)-C(4)-H(4)	120.4	C(3)-C(4)-C(5)	119.22(12)
C(5)-C(4)-H(4)	120.4	C(4)-C(5)-H(5)	119.2
C(4)-C(5)-C(6)	121.67(12)	C(6)-C(5)-H(5)	119.2
C(1)-C(6)-C(7)	124.17(11)	C(5)-C(6)-C(1)	119.84(11)
C(5)-C(6)-C(7)	115.98(11)	C(8)-C(7)-C(6)	122.55(11)
C(12)-C(7)-C(6)	118.58(11)	C(12)-C(7)-C(8)	118.75(11)
C(7)-C(8)-H(8)	119.7	C(7)-C(8)-C(9)	120.64(11)
C(9)-C(8)-H(8)	119.7	C(8)-C(9)-C(13)	121.27(11)
C(10)-C(9)-C(8)	119.15(11)	C(10)-C(9)-C(13)	119.44(10)
C(9)-C(10)-H(10)	119.3	C(11)-C(10)-C(9)	121.35(11)
C(11)-C(10)-H(10)	119.3	C(10)-C(11)-C(12)	118.36(11)
C(10)-C(11)-C(19)	121.11(10)	C(12)-C(11)-C(19)	120.28(10)
C(7)-C(12)-C(11)	121.73(11)	C(7)-C(12)-H(12)	119.1

C(11)-C(12)-H(12)	119.1	C(14)-C(13)-C(9)	122.30(11)
C(18)-C(13)-C(9)	117.69(11)	C(18)-C(13)-C(14)	119.93(11)
C(13)-C(14)-Si(2)	126.12(9)	C(15)-C(14)-Si(2)	116.13(9)
C(15)-C(14)-C(13)	117.02(11)	C(14)-C(15)-H(15)	118.7
C(16)-C(15)-C(14)	122.64(12)	C(16)-C(15)-H(15)	118.7
C(15)-C(16)-H(16)	120.2	C(15)-C(16)-C(17)	119.53(12)
C(17)-C(16)-H(16)	120.2	C(16)-C(17)-H(17)	120.3
C(16)-C(17)-C(18)	119.41(12)	C(18)-C(17)-H(17)	120.3
C(13)-C(18)-H(18)	119.3	C(17)-C(18)-C(13)	121.32(11)
C(17)-C(18)-H(18)	119.3	C(20)-C(19)-C(11)	123.00(11)
C(24)-C(19)-C(11)	117.29(11)	C(24)-C(19)-C(20)	119.60(11)
C(19)-C(20)-Si(3)	126.69(9)	C(21)-C(20)-Si(3)	115.34(9)
C(21)-C(20)-C(19)	117.22(11)	C(20)-C(21)-H(21)	118.8
C(22)-C(21)-C(20)	122.41(12)	C(22)-C(21)-H(21)	118.8
C(21)-C(22)-H(22)	120.1	C(21)-C(22)-C(23)	119.73(12)
C(23)-C(22)-H(22)	120.1	C(22)-C(23)-H(23)	120.4
C(24)-C(23)-C(22)	119.29(12)	C(24)-C(23)-H(23)	120.4
C(19)-C(24)-H(24)	119.2	C(23)-C(24)-C(19)	121.51(12)
C(23)-C(24)-H(24)	119.2	C(26)-C(25)-Si(1)	114.78(10)
C(26)-C(25)-C(27)	108.63(13)	C(26)-C(25)-C(28)	108.20(13)
C(27)-C(25)-Si(1)	110.35(10)	C(27)-C(25)-C(28)	108.48(13)
C(28)-C(25)-Si(1)	106.19(11)	C(25)-C(26)-H(26A)	109.5
C(25)-C(26)-H(26B)	109.5	C(25)-C(26)-H(26C)	109.5
H(26A)-C(26)-H(26B)	109.5	H(26A)-C(26)-H(26C)	109.5
H(26B)-C(26)-H(26C)	109.5	C(25)-C(27)-H(27A)	109.5
C(25)-C(27)-H(27B)	109.5	C(25)-C(27)-H(27C)	109.5
H(27A)-C(27)-H(27B)	109.5	H(27A)-C(27)-H(27C)	109.5
H(27B)-C(27)-H(27C)	109.5	C(25)-C(28)-H(28A)	109.5
C(25)-C(28)-H(28B)	109.5	C(25)-C(28)-H(28C)	109.5
H(28A)-C(28)-H(28B)	109.5	H(28A)-C(28)-H(28C)	109.5
H(28B)-C(28)-H(28C)	109.5	C(30)-C(29)-Si(1)	114.87(10)
C(30)-C(29)-C(31)	108.20(12)	C(31)-C(29)-Si(1)	108.59(10)
C(32)-C(29)-Si(1)	108.25(10)	C(32)-C(29)-C(30)	108.82(12)
C(32)-C(29)-C(31)	107.92(12)	C(29)-C(30)-H(30A)	109.5
C(29)-C(30)-H(30B)	109.5	C(29)-C(30)-H(30C)	109.5
H(30A)-C(30)-H(30B)	109.5	H(30A)-C(30)-H(30C)	109.5

H(30B)-C(30)-H(30C)	109.5	C(29)-C(31)-H(31A)	109.5
C(29)-C(31)-H(31B)	109.5	C(29)-C(31)-H(31C)	109.5
H(31A)-C(31)-H(31B)	109.5	H(31A)-C(31)-H(31C)	109.5
H(31B)-C(31)-H(31C)	109.5	C(29)-C(32)-H(32A)	109.5
C(29)-C(32)-H(32B)	109.5	C(29)-C(32)-H(32C)	109.5
H(32A)-C(32)-H(32B)	109.5	H(32A)-C(32)-H(32C)	109.5
H(32B)-C(32)-H(32C)	109.5	C(34)-C(33)-Si(2)	110.19(11)
C(34)-C(33)-C(35)	108.84(14)	C(35)-C(33)-Si(2)	105.42(10)
C(36)-C(33)-Si(2)	115.31(10)	C(36)-C(33)-C(34)	108.42(12)
C(36)-C(33)-C(35)	108.48(15)	C(33)-C(34)-H(34A)	109.5
C(33)-C(34)-H(34B)	109.5	C(33)-C(34)-H(34C)	109.5
H(34A)-C(34)-H(34B)	109.5	H(34A)-C(34)-H(34C)	109.5
H(34B)-C(34)-H(34C)	109.5	C(33)-C(35)-H(35A)	109.5
C(33)-C(35)-H(35B)	109.5	C(33)-C(35)-H(35C)	109.5
H(35A)-C(35)-H(35B)	109.5	H(35A)-C(35)-H(35C)	109.5
H(35B)-C(35)-H(35C)	109.5	C(33)-C(36)-H(36A)	109.5
C(33)-C(36)-H(36B)	109.5	C(33)-C(36)-H(36C)	109.5
H(36A)-C(36)-H(36B)	109.5	H(36A)-C(36)-H(36C)	109.5
H(36B)-C(36)-H(36C)	109.5	C(38)-C(37)-Si(2)	109.29(10)
C(39)-C(37)-Si(2)	115.00(10)	C(39)-C(37)-C(38)	108.02(12)
C(39)-C(37)-C(40)	109.05(13)	C(40)-C(37)-Si(2)	107.68(9)
C(40)-C(37)-C(38)	107.57(13)	C(37)-C(38)-H(38A)	109.5
C(37)-C(38)-H(38B)	109.5	C(37)-C(38)-H(38C)	109.5
H(38A)-C(38)-H(38B)	109.5	H(38A)-C(38)-H(38C)	109.5
H(38B)-C(38)-H(38C)	109.5	C(37)-C(39)-H(39A)	109.5
C(37)-C(39)-H(39B)	109.5	C(37)-C(39)-H(39C)	109.5
H(39A)-C(39)-H(39B)	109.5	H(39A)-C(39)-H(39C)	109.5
H(39B)-C(39)-H(39C)	109.5	C(37)-C(40)-H(40A)	109.5
C(37)-C(40)-H(40B)	109.5	C(37)-C(40)-H(40C)	109.5
H(40A)-C(40)-H(40B)	109.5	H(40A)-C(40)-H(40C)	109.5
H(40B)-C(40)-H(40C)	109.5	C(42A)-C(41)-Si(3)	104.79(15)
C(42B)-C(41)-Si(3)	110.0(2)	C(42B)-C(41)-C(43B)	114.7(4)
C(42B)-C(41)-C(44B)	114.3(4)	C(43A)-C(41)-Si(3)	116.72(14)
C(43A)-C(41)-C(42A)	106.2(2)	C(43B)-C(41)-Si(3)	106.4(2)
C(44A)-C(41)-Si(3)	114.32(17)	C(44A)-C(41)-C(42A)	105.8(2)
C(44A)-C(41)-C(43A)	108.1(2)	C(44B)-C(41)-Si(3)	105.5(2)

C(44B)-C(41)-C(43B)	105.3(3)	C(41)-C(42A)-H(42A)	109.5
C(41)-C(42A)-H(42B)	109.5	C(41)-C(42A)-H(42C)	109.5
H(42A)-C(42A)-H(42B)	109.5	H(42A)-C(42A)-H(42C)	109.5
H(42B)-C(42A)-H(42C)	109.5	C(41)-C(42B)-H(42D)	109.5
C(41)-C(42B)-H(42E)	109.5	C(41)-C(42B)-H(42F)	109.5
H(42D)-C(42B)-H(42E)	109.5	H(42D)-C(42B)-H(42F)	109.5
H(42E)-C(42B)-H(42F)	109.5	C(41)-C(43A)-H(43B)	109.5
C(41)-C(43A)-H(43C)	109.5	C(41)-C(43A)-H(43A)	109.5
H(43B)-C(43A)-H(43C)	109.5	H(43B)-C(43A)-H(43A)	109.5
H(43C)-C(43A)-H(43A)	109.5	C(41)-C(43B)-H(43E)	109.5
C(41)-C(43B)-H(43D)	109.5	C(41)-C(43B)-H(43F)	109.5
H(43E)-C(43B)-H(43D)	109.5	H(43E)-C(43B)-H(43F)	109.5
H(43D)-C(43B)-H(43F)	109.5	C(41)-C(44A)-H(44A)	109.5
C(41)-C(44A)-H(44B)	109.5	C(41)-C(44A)-H(44C)	109.5
H(44A)-C(44A)-H(44B)	109.5	H(44A)-C(44A)-H(44C)	109.5
H(44B)-C(44A)-H(44C)	109.5	C(41)-C(44B)-H(44D)	109.5
C(41)-C(44B)-H(44E)	109.5	C(41)-C(44B)-H(44F)	109.5
H(44D)-C(44B)-H(44E)	109.5	H(44D)-C(44B)-H(44F)	109.5
H(44E)-C(44B)-H(44F)	109.5	C(46A)-C(45A)-Si(3)	117.9(3)
C(46A)-C(45A)-C(47A)	108.9(4)	C(46A)-C(45A)-C(48A)	108.3(5)
C(47A)-C(45A)-Si(3)	108.2(4)	C(47A)-C(45A)-C(48A)	107.2(6)
C(48A)-C(45A)-Si(3)	105.8(6)	C(46B)-C(45B)-Si(3)	113.3(5)
C(46B)-C(45B)-C(47B)	106.1(7)	C(47B)-C(45B)-Si(3)	108.9(5)
C(48B)-C(45B)-Si(3)	111.1(11)	C(48B)-C(45B)-C(46B)	110.9(9)
C(48B)-C(45B)-C(47B)	106.1(8)	C(45A)-C(46A)-H(46A)	109.5
C(45A)-C(46A)-H(46B)	109.5	C(45A)-C(46A)-H(46C)	109.5
H(46A)-C(46A)-H(46B)	109.5	H(46A)-C(46A)-H(46C)	109.5
H(46B)-C(46A)-H(46C)	109.5	C(45B)-C(46B)-H(46D)	109.5
C(45B)-C(46B)-H(46E)	109.5	C(45B)-C(46B)-H(46F)	109.5
H(46D)-C(46B)-H(46E)	109.5	H(46D)-C(46B)-H(46F)	109.5
H(46E)-C(46B)-H(46F)	109.5	C(45A)-C(47A)-H(47A)	109.5
C(45A)-C(47A)-H(47B)	109.5	C(45A)-C(47A)-H(47C)	109.5
H(47A)-C(47A)-H(47B)	109.5	H(47A)-C(47A)-H(47C)	109.5
H(47B)-C(47A)-H(47C)	109.5	C(45B)-C(47B)-H(47D)	109.5
C(45B)-C(47B)-H(47E)	109.5	C(45B)-C(47B)-H(47F)	109.5
H(47D)-C(47B)-H(47E)	109.5	H(47D)-C(47B)-H(47F)	109.5

H(47E)-C(47B)-H(47F)	109.5	C(45A)-C(48A)-H(48A)	109.5
C(45A)-C(48A)-H(48B)	109.5	C(45A)-C(48A)-H(48C)	109.5
H(48A)-C(48A)-H(48B)	109.5	H(48A)-C(48A)-H(48C)	109.5
H(48B)-C(48A)-H(48C)	109.5	C(45B)-C(48B)-H(48D)	109.5
C(45B)-C(48B)-H(48E)	109.5	C(45B)-C(48B)-H(48F)	109.5
H(48D)-C(48B)-H(48E)	109.5	H(48D)-C(48B)-H(48F)	109.5
H(48E)-C(48B)-H(48F)	109.5		

Single crystal structure analysis of ligand 7e hydrate · hexamethyldisiloxane solvate

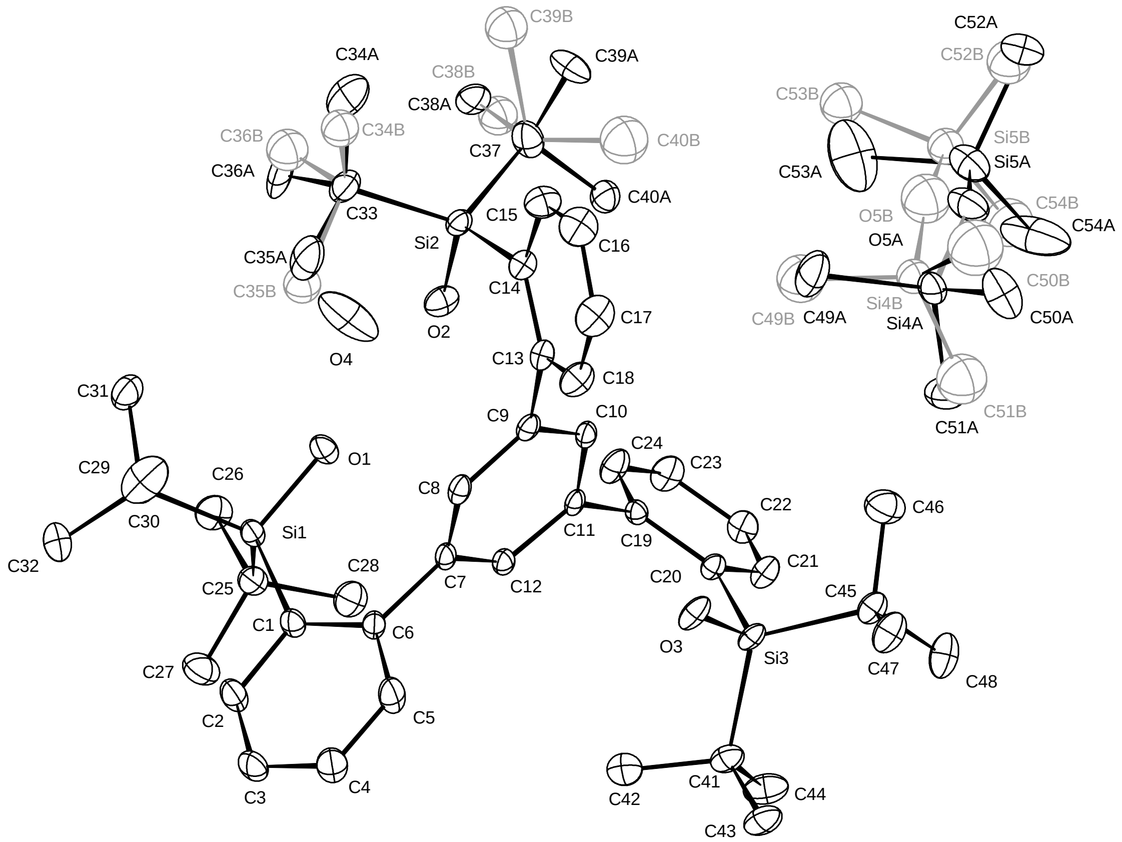

Figure S 3. The molecular structure of ligand **7e hydrate · hexamethyldisiloxane solvate**. H-atoms have been removed for clarity. Main structure shown in black and disordered parts shown in grey.

X-ray Crystal Structure Analysis of Ligand 7e hydrate · hexamethyldisiloxane solvate:
C_{54} H_{92} O_5 Si_5, M_r = 961.72 g mol^{-1}, colourless block, crystal size 0.203 x 0.108 x 0.06 mm^3, Monoclinic, space group $P2_1/c$ [14], a = 23.8929(9) Å, b = 10.0543(3) Å, c = 25.3872(10) Å, β = 102.301(2)°, V = 5958.7(4) Å^3, T = 100(2) K, Z = 4, D_{calc} = 1.072 g·cm^3, λ = 0.71073 Å, μ(*Mo-K$_\alpha$*) = 0.160 mm^{-1}, Gaussian absorption correction ($T_{\min}$ = 0.968, $T_{\max}$ = 0.990), Bruker AXS D8-Venture diffractometer with IµS Diamond Mo-anode X-ray source and PHOTON III detector, 2.017 < θ < 31.506°, 382203 measured reflections, 19830independent reflections, 17147 reflections with $I > 2\sigma(I)$, R_{int} = 0.0761. The structure was solved by *SHELXT* and refined by full-matrix least-squares (*SHELXL*) against F^2 to R_1 = 0.0590 [$I > 2\sigma(I)$], wR_2 = 0.1339 [all data], 691parameters and 0 restraints.

Full .cif data for the compound are available under the CCDC number **CCDC-2265455**

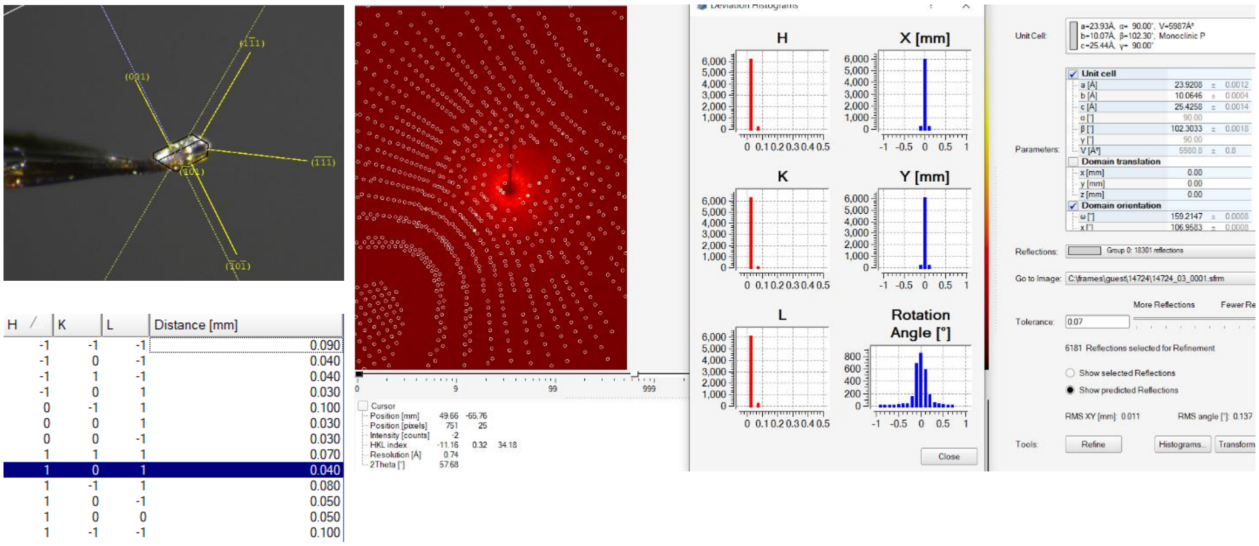

Figure S 4. Crystal faces and unit cell determination/refinement of ligand **7e hydrate · hexamethyldisiloxane solvate**.

INTENSITY STATISTICS FOR DATASET

Resolution	#Data	#Theory	%Complete	Redundancy	Mean I	Mean I/s	Rmerge	Rsigma
Inf - 2.62	408	416	98.1	24.78	56.33	101.28	0.0289	0.0111
2.62 - 1.73	941	941	100.0	26.19	24.63	78.34	0.0324	0.0083
1.73 - 1.36	1377	1377	100.0	25.95	12.90	56.72	0.0451	0.0114
1.36 - 1.18	1413	1413	100.0	26.06	10.54	48.01	0.0534	0.0135
1.18 - 1.07	1367	1367	100.0	25.62	6.12	35.40	0.0792	0.0197
1.07 - 0.99	1398	1398	100.0	24.62	4.34	27.09	0.1056	0.0264
0.99 - 0.93	1390	1390	100.0	21.65	3.87	22.34	0.1198	0.0317
0.93 - 0.89	1156	1156	100.0	20.28	3.42	19.71	0.1311	0.0367
0.89 - 0.85	1345	1345	100.0	19.19	3.08	17.25	0.1506	0.0426
0.85 - 0.81	1665	1665	100.0	17.21	2.43	13.89	0.1832	0.0559
0.81 - 0.78	1442	1442	100.0	15.79	2.24	11.91	0.2030	0.0658
0.78 - 0.76	1126	1126	100.0	14.79	1.89	10.02	0.2326	0.0791
0.76 - 0.74	1222	1222	100.0	14.33	1.62	8.82	0.2675	0.0946
0.74 - 0.72	1333	1333	100.0	11.93	1.46	7.08	0.2933	0.1187
0.72 - 0.70	1558	1558	100.0	11.26	1.42	6.66	0.2988	0.1274
0.70 - 0.68	1710	1710	100.0	10.54	1.21	5.50	0.3524	0.1574
0.68 - 0.67	925	925	100.0	8.12	1.12	4.42	0.3709	0.2016
0.67 - 0.65	2033	2041	99.6	6.34	0.98	3.39	0.4119	0.2762
0.65 - 0.64	1129	1129	100.0	4.92	0.89	2.55	0.4489	0.3580
0.64 - 0.63	1167	1168	99.9	4.24	0.83	2.17	0.4758	0.4243
0.63 - 0.62	848	905	93.7	3.34	0.73	1.77	0.4867	0.5292
0.72 - 0.62	9370	9436	99.3	7.37	1.06	4.02	0.3652	0.2487
Inf - 0.62	26953	27027	99.7	15.81	4.82	19.80	0.0787	0.0397

A resolution cut off (SHEL 999 0.68) was applied to exclude poorly determined reflections at high diffraction angles. Eight reflections were omitted from the data set prior to the final refinement cycles due to high I/σI (> 10).

Two ^{t}Bu groups at Si2 show twofold positional disorders. They were described with fixed occupancies of 80:20% and 75:25%. The ISOR instruction was used to treat the thermal ellipsoids

of C50B C49B C51B C53B C52B C54B. In addition, disorders (twofold positional) were found in the hexamethyldisiloxane solute molecule. The occupancy was refined by applying a free variable (FVAR) and resulted in a final occupancy of 84.4:15.6%. Isotropic atomic displacement parameters were used for the minor moieties.

The structure shows an unusual hydrogen bond driven structure. H-atoms could be localised in the residual electron density map and are partly freely refined. Due to the presence of a water molecule, the inner ligand pockets open up and become more accessible. This geometry is enhanced by the rotation of one phenyl-di(^{t}Bu)silyloxi group to approximately 180°. An -OH is directed towards the central aryl moiety of the canopy ligand. Such unconventional OH···π hydrogen bonds have been reported recently: *J. Org. Chem.* **2020**, 85, 15, 9801–9807 https://doi.org/10.1021/acs.joc.0c01121

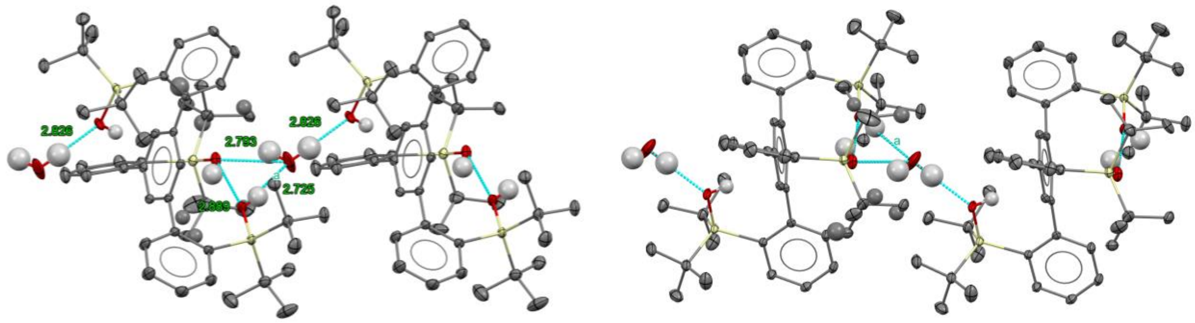

Figure S 5. Hydrogen bonding motif in ligand **7e hydrate · hexamethyldisiloxane solvate** from two different orientations.

Table S 3. Crystal data and structure refinement of compound ligand **7e hydrate · hexamethyldisiloxane solvate**.

Identification code	14724	
Empirical formula	$C_{54} H_{92} O_5 Si_5$	
Color	colourless	
Formula weight	961.72 g·mol^{-1}	
Temperature	100(2) K	
Wavelength	0.71073 Å	
Crystal system	Monoclinic	
Space group	$P2_1/c$, (no. 14)	
Unit cell dimensions	a = 23.8929(9) Å	α= 90°.
	b = 10.0543(3) Å	β= 102.301(2)°.
	c = 25.3872(10) Å	γ = 90°.
Volume	5958.7(4) Å^3	
Z	4	
Density (calculated)	1.072 Mg·m^{-3}	
Absorption coefficient	0.160 mm^{-1}	
F(000)	2104 e	
Crystal size	0.203 x 0.108 x 0.06 mm^3	
θ range for data collection	2.017 to 31.506°.	
Index ranges	-35 ≤ h ≤ 35, -14≤ k ≤ 14, -37 ≤ l ≤ 37	
Reflections collected	382203	
Independent reflections	19830 [R_{int} = 0.0761]	
Reflections with I>2σ(I)	17147	
Completeness to θ = 25.242°	99.9 %	
Absorption correction	Gaussian	
Max. and min. transmission	0.990 and 0.968	
Refinement method	Full-matrix least-squares on F^2	
Data / restraints / parameters	19830 / 0 / 691	
Goodness-of-fit on F^2	1.126	
Final R indices [I>2σ(I)]	R_1 = 0.0590	wR^2 = 0.1285
R indices (all data)	R_1 = 0.0704	wR^2 = 0.1339
Extinction coefficient	n/a	
Largest diff. peak and hole	0.657 and -0.500 e·Å^{-3}	

Table S 4. Bond lengths [Å] and angles [°] of ligand **7e hydrate · hexamethyldisiloxane solvate**.

Si(1)-O(1)	1.6473(11)	Si(1)-C(1)	1.8900(14)
Si(1)-C(25)	1.9079(16)	Si(1)-C(29)	1.9122(16)
Si(2)-O(2)	1.6499(12)	Si(2)-C(14)	1.8943(14)
Si(2)-C(33)	1.8958(16)	Si(2)-C(37)	1.8984(16)
Si(3)-O(3)	1.6623(10)	Si(3)-C(20)	1.8947(13)
Si(3)-C(41)	1.9006(16)	Si(3)-C(45)	1.9060(16)
O(1)-H(1)	0.73(3)	O(2)-H(2)	0.73(3)
O(3)-H(3)	0.8400	C(1)-C(2)	1.409(2)
C(1)-C(6)	1.4074(19)	C(2)-H(2A)	0.9500
C(2)-C(3)	1.387(2)	C(3)-H(3A)	0.9500
C(3)-C(4)	1.383(2)	C(4)-H(4)	0.9500
C(4)-C(5)	1.386(2)	C(5)-H(5)	0.9500
C(5)-C(6)	1.4012(19)	C(6)-C(7)	1.4948(19)
C(7)-C(8)	1.394(2)	C(7)-C(12)	1.3944(18)
C(8)-H(8)	0.9500	C(8)-C(9)	1.3941(19)
C(9)-C(10)	1.4005(18)	C(9)-C(13)	1.4910(19)
C(10)-H(10)	0.9500	C(10)-C(11)	1.3947(19)
C(11)-C(12)	1.3948(18)	C(11)-C(19)	1.4949(18)
C(12)-H(12)	0.9500	C(13)-C(14)	1.408(2)
C(13)-C(18)	1.4027(19)	C(14)-C(15)	1.408(2)
C(15)-H(15)	0.9500	C(15)-C(16)	1.389(2)
C(16)-H(16)	0.9500	C(16)-C(17)	1.382(3)
C(17)-H(17)	0.9500	C(17)-C(18)	1.385(2)
C(18)-H(18)	0.9500	C(19)-C(20)	1.4069(18)
C(19)-C(24)	1.3970(18)	C(20)-C(21)	1.4060(18)
C(21)-H(21)	0.9500	C(21)-C(22)	1.3858(19)
C(22)-H(22)	0.9500	C(22)-C(23)	1.382(2)
C(23)-H(23)	0.9500	C(23)-C(24)	1.387(2)
C(24)-H(24)	0.9500	C(25)-C(26)	1.542(2)
C(25)-C(27)	1.536(2)	C(25)-C(28)	1.536(2)
C(26)-H(26A)	0.9800	C(26)-H(26B)	0.9800
C(26)-H(26C)	0.9800	C(27)-H(27A)	0.9800
C(27)-H(27B)	0.9800	C(27)-H(27C)	0.9800

C(28)-H(28A)	0.9800	C(28)-H(28B)	0.9800
C(28)-H(28C)	0.9800	C(29)-C(30)	1.530(3)
C(29)-C(31)	1.541(2)	C(29)-C(32)	1.546(2)
C(30)-H(30A)	0.9800	C(30)-H(30B)	0.9800
C(30)-H(30C)	0.9800	C(31)-H(31A)	0.9800
C(31)-H(31B)	0.9800	C(31)-H(31C)	0.9800
C(32)-H(32A)	0.9800	C(32)-H(32B)	0.9800
C(32)-H(32C)	0.9800	C(33)-C(34A)	1.533(3)
C(33)-C(34B)	1.557(11)	C(33)-C(35A)	1.499(3)
C(33)-C(35B)	1.688(14)	C(33)-C(36A)	1.560(4)
C(33)-C(36B)	1.406(18)	C(34A)-H(34A)	0.9800
C(34A)-H(34B)	0.9800	C(34A)-H(34C)	0.9800
C(34B)-H(34D)	0.9800	C(34B)-H(34E)	0.9800
C(34B)-H(34F)	0.9800	C(35A)-H(35A)	0.9800
C(35A)-H(35B)	0.9800	C(35A)-H(35C)	0.9800
C(35B)-H(35D)	0.9800	C(35B)-H(35E)	0.9800
C(35B)-H(35F)	0.9800	C(36A)-H(36A)	0.9800
C(36A)-H(36B)	0.9800	C(36A)-H(36C)	0.9800
C(36B)-H(36D)	0.9800	C(36B)-H(36E)	0.9800
C(36B)-H(36F)	0.9800	C(37)-C(38A)	1.547(3)
C(37)-C(38B)	1.370(11)	C(37)-C(39A)	1.488(3)
C(37)-C(39B)	1.890(9)	C(37)-C(40A)	1.590(3)
C(37)-C(40B)	1.474(10)	C(38A)-H(38A)	0.9800
C(38A)-H(38B)	0.9800	C(38A)-H(38C)	0.9800
C(38B)-H(38D)	0.9800	C(38B)-H(38E)	0.9800
C(38B)-H(38F)	0.9800	C(39A)-H(39A)	0.9800
C(39A)-H(39B)	0.9800	C(39A)-H(39C)	0.9800
C(39B)-H(39D)	0.9800	C(39B)-H(39E)	0.9800
C(39B)-H(39F)	0.9800	C(40A)-H(40A)	0.9800
C(40A)-H(40B)	0.9800	C(40A)-H(40C)	0.9800
C(40B)-H(40D)	0.9800	C(40B)-H(40E)	0.9800
C(40B)-H(40F)	0.9800	C(41)-C(42)	1.544(2)
C(41)-C(43)	1.539(2)	C(41)-C(44)	1.536(2)
C(42)-H(42A)	0.9800	C(42)-H(42B)	0.9800
C(42)-H(42C)	0.9800	C(43)-H(43A)	0.9800
C(43)-H(43B)	0.9800	C(43)-H(43C)	0.9800

C(44)-H(44A)	0.9800	C(44)-H(44B)	0.9800
C(44)-H(44C)	0.9800	C(45)-C(46)	1.532(2)
C(45)-C(47)	1.541(2)	C(45)-C(48)	1.539(2)
C(46)-H(46A)	0.9800	C(46)-H(46B)	0.9800
C(46)-H(46C)	0.9800	C(47)-H(47A)	0.9800
C(47)-H(47B)	0.9800	C(47)-H(47C)	0.9800
C(48)-H(48A)	0.9800	C(48)-H(48B)	0.9800
C(48)-H(48C)	0.9800	Si(4A)-O(5A)	1.6382(18)
Si(4A)-C(49A)	1.863(3)	Si(4A)-C(50A)	1.860(3)
Si(4A)-C(51A)	1.869(3)	Si(4B)-O(5B)	1.526(14)
Si(4B)-C(49B)	1.70(2)	Si(4B)-C(50B)	1.92(2)
Si(4B)-C(51B)	1.76(3)	Si(5A)-O(5A)	1.633(2)
Si(5A)-C(52A)	1.877(5)	Si(5A)-C(53A)	1.861(4)
Si(5A)-C(54A)	1.847(4)	Si(5B)-O(5B)	1.684(14)
Si(5B)-C(52B)	1.62(3)	Si(5B)-C(53B)	1.812(16)
Si(5B)-C(54B)	1.92(3)	C(49A)-H(49A)	0.9800
C(49A)-H(49B)	0.9800	C(49A)-H(49C)	0.9800
C(49B)-H(49D)	0.9800	C(49B)-H(49E)	0.9800
C(49B)-H(49F)	0.9800	C(50A)-H(50A)	0.9800
C(50A)-H(50B)	0.9800	C(50A)-H(50C)	0.9800
C(50B)-H(50D)	0.9800	C(50B)-H(50E)	0.9800
C(50B)-H(50F)	0.9800	C(51A)-H(51A)	0.9800
C(51A)-H(51B)	0.9800	C(51A)-H(51C)	0.9800
C(51B)-H(51D)	0.9800	C(51B)-H(51E)	0.9800
C(51B)-H(51F)	0.9800	C(52A)-H(52A)	0.9800
C(52A)-H(52B)	0.9800	C(52A)-H(52C)	0.9800
C(52B)-H(52D)	0.9800	C(52B)-H(52E)	0.9800
C(52B)-H(52F)	0.9800	C(53A)-H(53A)	0.9800
C(53A)-H(53B)	0.9800	C(53A)-H(53C)	0.9800
C(53B)-H(53D)	0.9800	C(53B)-H(53E)	0.9800
C(53B)-H(53F)	0.9800	C(54A)-H(54A)	0.9800
C(54A)-H(54B)	0.9800	C(54A)-H(54C)	0.9800
C(54B)-H(54D)	0.9800	C(54B)-H(54E)	0.9800
C(54B)-H(54F)	0.9800	O(4)-H(4A)	0.90(4)
O(4)-H(4B)	0.89(4)		

O(1)-Si(1)-C(1)	108.68(6)	O(1)-Si(1)-C(25)	107.69(6)
O(1)-Si(1)-C(29)	106.54(7)	C(1)-Si(1)-C(25)	108.38(7)
C(1)-Si(1)-C(29)	109.32(7)	C(25)-Si(1)-C(29)	116.01(7)
O(2)-Si(2)-C(14)	112.77(6)	O(2)-Si(2)-C(33)	106.74(7)
O(2)-Si(2)-C(37)	101.34(8)	C(14)-Si(2)-C(33)	106.61(7)
C(14)-Si(2)-C(37)	111.26(7)	C(33)-Si(2)-C(37)	118.13(8)
O(3)-Si(3)-C(20)	111.61(6)	O(3)-Si(3)-C(41)	106.14(6)
O(3)-Si(3)-C(45)	103.92(6)	C(20)-Si(3)-C(41)	108.16(6)
C(20)-Si(3)-C(45)	110.22(6)	C(41)-Si(3)-C(45)	116.69(7)
Si(1)-O(1)-H(1)	114(2)	Si(2)-O(2)-H(2)	117(2)
Si(3)-O(3)-H(3)	109.5	C(2)-C(1)-Si(1)	115.68(10)
C(6)-C(1)-Si(1)	127.53(10)	C(6)-C(1)-C(2)	116.78(12)
C(1)-C(2)-H(2A)	118.6	C(3)-C(2)-C(1)	122.73(14)
C(3)-C(2)-H(2A)	118.6	C(2)-C(3)-H(3A)	120.3
C(4)-C(3)-C(2)	119.39(14)	C(4)-C(3)-H(3A)	120.3
C(3)-C(4)-H(4)	120.2	C(3)-C(4)-C(5)	119.65(14)
C(5)-C(4)-H(4)	120.2	C(4)-C(5)-H(5)	119.5
C(4)-C(5)-C(6)	121.08(14)	C(6)-C(5)-H(5)	119.5
C(1)-C(6)-C(7)	123.20(12)	C(5)-C(6)-C(1)	120.33(13)
C(5)-C(6)-C(7)	116.43(12)	C(8)-C(7)-C(6)	121.66(12)
C(8)-C(7)-C(12)	118.94(12)	C(12)-C(7)-C(6)	119.31(13)
C(7)-C(8)-H(8)	119.6	C(9)-C(8)-C(7)	120.81(12)
C(9)-C(8)-H(8)	119.6	C(8)-C(9)-C(10)	119.30(13)
C(8)-C(9)-C(13)	120.96(12)	C(10)-C(9)-C(13)	119.72(12)
C(9)-C(10)-H(10)	119.7	C(11)-C(10)-C(9)	120.66(12)
C(11)-C(10)-H(10)	119.7	C(10)-C(11)-C(12)	118.91(12)
C(10)-C(11)-C(19)	121.44(12)	C(12)-C(11)-C(19)	119.65(12)
C(7)-C(12)-C(11)	121.31(13)	C(7)-C(12)-H(12)	119.3
C(11)-C(12)-H(12)	119.3	C(14)-C(13)-C(9)	122.27(12)
C(18)-C(13)-C(9)	117.44(13)	C(18)-C(13)-C(14)	120.29(13)
C(13)-C(14)-Si(2)	125.71(11)	C(15)-C(14)-Si(2)	116.93(11)
C(15)-C(14)-C(13)	116.86(13)	C(14)-C(15)-H(15)	118.8
C(16)-C(15)-C(14)	122.45(15)	C(16)-C(15)-H(15)	118.8
C(15)-C(16)-H(16)	120.1	C(17)-C(16)-C(15)	119.73(15)
C(17)-C(16)-H(16)	120.1	C(16)-C(17)-H(17)	120.3
C(16)-C(17)-C(18)	119.47(15)	C(18)-C(17)-H(17)	120.3

C(13)-C(18)-H(18)	119.4	C(17)-C(18)-C(13)	121.17(15)
C(17)-C(18)-H(18)	119.4	C(20)-C(19)-C(11)	122.24(11)
C(24)-C(19)-C(11)	117.37(11)	C(24)-C(19)-C(20)	120.37(12)
C(19)-C(20)-Si(3)	126.61(10)	C(21)-C(20)-Si(3)	116.48(10)
C(21)-C(20)-C(19)	116.88(12)	C(20)-C(21)-H(21)	118.7
C(22)-C(21)-C(20)	122.58(13)	C(22)-C(21)-H(21)	118.7
C(21)-C(22)-H(22)	120.2	C(23)-C(22)-C(21)	119.52(13)
C(23)-C(22)-H(22)	120.2	C(22)-C(23)-H(23)	120.2
C(22)-C(23)-C(24)	119.57(13)	C(24)-C(23)-H(23)	120.2
C(19)-C(24)-H(24)	119.5	C(23)-C(24)-C(19)	121.07(13)
C(23)-C(24)-H(24)	119.5	C(26)-C(25)-Si(1)	110.75(11)
C(27)-C(25)-Si(1)	113.83(11)	C(27)-C(25)-C(26)	108.54(13)
C(27)-C(25)-C(28)	107.47(13)	C(28)-C(25)-Si(1)	108.03(10)
C(28)-C(25)-C(26)	108.02(13)	C(25)-C(26)-H(26A)	109.5
C(25)-C(26)-H(26B)	109.5	C(25)-C(26)-H(26C)	109.5
H(26A)-C(26)-H(26B)	109.5	H(26A)-C(26)-H(26C)	109.5
H(26B)-C(26)-H(26C)	109.5	C(25)-C(27)-H(27A)	109.5
C(25)-C(27)-H(27B)	109.5	C(25)-C(27)-H(27C)	109.5
H(27A)-C(27)-H(27B)	109.5	H(27A)-C(27)-H(27C)	109.5
H(27B)-C(27)-H(27C)	109.5	C(25)-C(28)-H(28A)	109.5
C(25)-C(28)-H(28B)	109.5	C(25)-C(28)-H(28C)	109.5
H(28A)-C(28)-H(28B)	109.5	H(28A)-C(28)-H(28C)	109.5
H(28B)-C(28)-H(28C)	109.5	C(30)-C(29)-Si(1)	108.47(12)
C(30)-C(29)-C(31)	107.81(15)	C(30)-C(29)-C(32)	108.78(17)
C(31)-C(29)-Si(1)	109.21(11)	C(31)-C(29)-C(32)	106.87(14)
C(32)-C(29)-Si(1)	115.45(13)	C(29)-C(30)-H(30A)	109.5
C(29)-C(30)-H(30B)	109.5	C(29)-C(30)-H(30C)	109.5
H(30A)-C(30)-H(30B)	109.5	H(30A)-C(30)-H(30C)	109.5
H(30B)-C(30)-H(30C)	109.5	C(29)-C(31)-H(31A)	109.5
C(29)-C(31)-H(31B)	109.5	C(29)-C(31)-H(31C)	109.5
H(31A)-C(31)-H(31B)	109.5	H(31A)-C(31)-H(31C)	109.5
H(31B)-C(31)-H(31C)	109.5	C(29)-C(32)-H(32A)	109.5
C(29)-C(32)-H(32B)	109.5	C(29)-C(32)-H(32C)	109.5
H(32A)-C(32)-H(32B)	109.5	H(32A)-C(32)-H(32C)	109.5
H(32B)-C(32)-H(32C)	109.5	C(34A)-C(33)-Si(2)	112.60(15)
C(34A)-C(33)-C(36A)	106.8(2)	C(34B)-C(33)-Si(2)	118.4(4)

C(34B)-C(33)-C(35B)	101.8(6)	C(35A)-C(33)-Si(2)	109.10(14)
C(35A)-C(33)-C(34A)	110.8(3)	C(35A)-C(33)-C(36A)	108.0(2)
C(35B)-C(33)-Si(2)	101.2(5)	C(36A)-C(33)-Si(2)	109.44(16)
C(36B)-C(33)-Si(2)	115.7(8)	C(36B)-C(33)-C(34B)	109.1(8)
C(36B)-C(33)-C(35B)	108.9(9)	C(33)-C(34A)-H(34A)	109.5
C(33)-C(34A)-H(34B)	109.5	C(33)-C(34A)-H(34C)	109.5
H(34A)-C(34A)-H(34B)	109.5	H(34A)-C(34A)-H(34C)	109.5
H(34B)-C(34A)-H(34C)	109.5	C(33)-C(34B)-H(34D)	109.5
C(33)-C(34B)-H(34E)	109.5	C(33)-C(34B)-H(34F)	109.5
H(34D)-C(34B)-H(34E)	109.5	H(34D)-C(34B)-H(34F)	109.5
H(34E)-C(34B)-H(34F)	109.5	C(33)-C(35A)-H(35A)	109.5
C(33)-C(35A)-H(35B)	109.5	C(33)-C(35A)-H(35C)	109.5
H(35A)-C(35A)-H(35B)	109.5	H(35A)-C(35A)-H(35C)	109.5
H(35B)-C(35A)-H(35C)	109.5	C(33)-C(35B)-H(35D)	109.5
C(33)-C(35B)-H(35E)	109.5	C(33)-C(35B)-H(35F)	109.5
H(35D)-C(35B)-H(35E)	109.5	H(35D)-C(35B)-H(35F)	109.5
H(35E)-C(35B)-H(35F)	109.5	C(33)-C(36A)-H(36A)	109.5
C(33)-C(36A)-H(36B)	109.5	C(33)-C(36A)-H(36C)	109.5
H(36A)-C(36A)-H(36B)	109.5	H(36A)-C(36A)-H(36C)	109.5
H(36B)-C(36A)-H(36C)	109.5	C(33)-C(36B)-H(36D)	109.5
C(33)-C(36B)-H(36E)	109.5	C(33)-C(36B)-H(36F)	109.5
H(36D)-C(36B)-H(36E)	109.5	H(36D)-C(36B)-H(36F)	109.5
H(36E)-C(36B)-H(36F)	109.5	C(38A)-C(37)-Si(2)	111.20(14)
C(38A)-C(37)-C(40A)	105.86(18)	C(38B)-C(37)-Si(2)	117.8(5)
C(38B)-C(37)-C(39B)	96.6(5)	C(38B)-C(37)-C(40B)	121.0(6)
C(39A)-C(37)-Si(2)	115.78(14)	C(39A)-C(37)-C(38A)	110.33(18)
C(39A)-C(37)-C(40A)	107.90(17)	C(39B)-C(37)-Si(2)	105.0(3)
C(40A)-C(37)-Si(2)	105.11(12)	C(40B)-C(37)-Si(2)	114.5(4)
C(40B)-C(37)-C(39B)	94.6(5)	C(37)-C(38A)-H(38A)	109.5
C(37)-C(38A)-H(38B)	109.5	C(37)-C(38A)-H(38C)	109.5
H(38A)-C(38A)-H(38B)	109.5	H(38A)-C(38A)-H(38C)	109.5
H(38B)-C(38A)-H(38C)	109.5	C(37)-C(38B)-H(38D)	109.5
C(37)-C(38B)-H(38E)	109.5	C(37)-C(38B)-H(38F)	109.5
H(38D)-C(38B)-H(38E)	109.5	H(38D)-C(38B)-H(38F)	109.5
H(38E)-C(38B)-H(38F)	109.5	C(37)-C(39A)-H(39A)	109.5
C(37)-C(39A)-H(39B)	109.5	C(37)-C(39A)-H(39C)	109.5

H(39A)-C(39A)-H(39B)	109.5	H(39A)-C(39A)-H(39C)	109.5
H(39B)-C(39A)-H(39C)	109.5	C(37)-C(39B)-H(39D)	109.5
C(37)-C(39B)-H(39E)	109.5	C(37)-C(39B)-H(39F)	109.5
H(39D)-C(39B)-H(39E)	109.5	H(39D)-C(39B)-H(39F)	109.5
H(39E)-C(39B)-H(39F)	109.5	C(37)-C(40A)-H(40A)	109.5
C(37)-C(40A)-H(40B)	109.5	C(37)-C(40A)-H(40C)	109.5
H(40A)-C(40A)-H(40B)	109.5	H(40A)-C(40A)-H(40C)	109.5
H(40B)-C(40A)-H(40C)	109.5	C(37)-C(40B)-H(40D)	109.5
C(37)-C(40B)-H(40E)	109.5	C(37)-C(40B)-H(40F)	109.5
H(40D)-C(40B)-H(40E)	109.5	H(40D)-C(40B)-H(40F)	109.5
H(40E)-C(40B)-H(40F)	109.5	C(42)-C(41)-Si(3)	107.41(11)
C(43)-C(41)-Si(3)	111.05(11)	C(43)-C(41)-C(42)	108.19(13)
C(44)-C(41)-Si(3)	113.05(11)	C(44)-C(41)-C(42)	107.81(15)
C(44)-C(41)-C(43)	109.16(13)	C(41)-C(42)-H(42A)	109.5
C(41)-C(42)-H(42B)	109.5	C(41)-C(42)-H(42C)	109.5
H(42A)-C(42)-H(42B)	109.5	H(42A)-C(42)-H(42C)	109.5
H(42B)-C(42)-H(42C)	109.5	C(41)-C(43)-H(43A)	109.5
C(41)-C(43)-H(43B)	109.5	C(41)-C(43)-H(43C)	109.5
H(43A)-C(43)-H(43B)	109.5	H(43A)-C(43)-H(43C)	109.5
H(43B)-C(43)-H(43C)	109.5	C(41)-C(44)-H(44A)	109.5
C(41)-C(44)-H(44B)	109.5	C(41)-C(44)-H(44C)	109.5
H(44A)-C(44)-H(44B)	109.5	H(44A)-C(44)-H(44C)	109.5
H(44B)-C(44)-H(44C)	109.5	C(46)-C(45)-Si(3)	108.50(11)
C(46)-C(45)-C(47)	107.64(14)	C(46)-C(45)-C(48)	108.81(15)
C(47)-C(45)-Si(3)	109.23(11)	C(48)-C(45)-Si(3)	114.88(11)
C(48)-C(45)-C(47)	107.55(12)	C(45)-C(46)-H(46A)	109.5
C(45)-C(46)-H(46B)	109.5	C(45)-C(46)-H(46C)	109.5
H(46A)-C(46)-H(46B)	109.5	H(46A)-C(46)-H(46C)	109.5
H(46B)-C(46)-H(46C)	109.5	C(45)-C(47)-H(47A)	109.5
C(45)-C(47)-H(47B)	109.5	C(45)-C(47)-H(47C)	109.5
H(47A)-C(47)-H(47B)	109.5	H(47A)-C(47)-H(47C)	109.5
H(47B)-C(47)-H(47C)	109.5	C(45)-C(48)-H(48A)	109.5
C(45)-C(48)-H(48B)	109.5	C(45)-C(48)-H(48C)	109.5
H(48A)-C(48)-H(48B)	109.5	H(48A)-C(48)-H(48C)	109.5
H(48B)-C(48)-H(48C)	109.5	O(5A)-Si(4A)-C(49A)	111.74(12)
O(5A)-Si(4A)-C(50A)	107.89(12)	O(5A)-Si(4A)-C(51A)	108.18(13)

C(49A)-Si(4A)-C(51A)	109.41(16)	C(50A)-Si(4A)-C(49A)	109.74(14)
C(50A)-Si(4A)-C(51A)	109.85(18)	O(5B)-Si(4B)-C(49B)	102.0(10)
O(5B)-Si(4B)-C(50B)	108.3(9)	O(5B)-Si(4B)-C(51B)	116.5(12)
C(49B)-Si(4B)-C(50B)	112.2(10)	C(49B)-Si(4B)-C(51B)	114.9(12)
C(51B)-Si(4B)-C(50B)	103.1(12)	O(5A)-Si(5A)-C(52A)	107.55(15)
O(5A)-Si(5A)-C(53A)	108.76(14)	O(5A)-Si(5A)-C(54A)	110.24(19)
C(53A)-Si(5A)-C(52A)	110.9(2)	C(54A)-Si(5A)-C(52A)	109.8(2)
C(54A)-Si(5A)-C(53A)	109.6(2)	O(5B)-Si(5B)-C(53B)	109.4(7)
O(5B)-Si(5B)-C(54B)	111.1(9)	C(52B)-Si(5B)-O(5B)	105.6(14)
C(52B)-Si(5B)-C(53B)	108.4(14)	C(52B)-Si(5B)-C(54B)	108.5(16)
C(53B)-Si(5B)-C(54B)	113.6(9)	Si(5A)-O(5A)-Si(4A)	143.92(12)
Si(4B)-O(5B)-Si(5B)	158.0(10)	Si(4A)-C(49A)-H(49A)	109.5
Si(4A)-C(49A)-H(49B)	109.5	Si(4A)-C(49A)-H(49C)	109.5
H(49A)-C(49A)-H(49B)	109.5	H(49A)-C(49A)-H(49C)	109.5
H(49B)-C(49A)-H(49C)	109.5	Si(4B)-C(49B)-H(49D)	109.5
Si(4B)-C(49B)-H(49E)	109.5	Si(4B)-C(49B)-H(49F)	109.5
H(49D)-C(49B)-H(49E)	109.5	H(49D)-C(49B)-H(49F)	109.5
H(49E)-C(49B)-H(49F)	109.5	Si(4A)-C(50A)-H(50A)	109.5
Si(4A)-C(50A)-H(50B)	109.5	Si(4A)-C(50A)-H(50C)	109.5
H(50A)-C(50A)-H(50B)	109.5	H(50A)-C(50A)-H(50C)	109.5
H(50B)-C(50A)-H(50C)	109.5	Si(4B)-C(50B)-H(50D)	109.5
Si(4B)-C(50B)-H(50E)	109.5	Si(4B)-C(50B)-H(50F)	109.5
H(50D)-C(50B)-H(50E)	109.5	H(50D)-C(50B)-H(50F)	109.5
H(50E)-C(50B)-H(50F)	109.5	Si(4A)-C(51A)-H(51A)	109.5
Si(4A)-C(51A)-H(51B)	109.5	Si(4A)-C(51A)-H(51C)	109.5
H(51A)-C(51A)-H(51B)	109.5	H(51A)-C(51A)-H(51C)	109.5
H(51B)-C(51A)-H(51C)	109.5	Si(4B)-C(51B)-H(51D)	109.5
Si(4B)-C(51B)-H(51E)	109.5	Si(4B)-C(51B)-H(51F)	109.5
H(51D)-C(51B)-H(51E)	109.5	H(51D)-C(51B)-H(51F)	109.5
H(51E)-C(51B)-H(51F)	109.5	Si(5A)-C(52A)-H(52A)	109.5
Si(5A)-C(52A)-H(52B)	109.5	Si(5A)-C(52A)-H(52C)	109.5
H(52A)-C(52A)-H(52B)	109.5	H(52A)-C(52A)-H(52C)	109.5
H(52B)-C(52A)-H(52C)	109.5	Si(5B)-C(52B)-H(52D)	109.5
Si(5B)-C(52B)-H(52E)	109.5	Si(5B)-C(52B)-H(52F)	109.5
H(52D)-C(52B)-H(52E)	109.5	H(52D)-C(52B)-H(52F)	109.5
H(52E)-C(52B)-H(52F)	109.5	Si(5A)-C(53A)-H(53A)	109.5

Si(5A)-C(53A)-H(53B)	109.5	Si(5A)-C(53A)-H(53C)	109.5
H(53A)-C(53A)-H(53B)	109.5	H(53A)-C(53A)-H(53C)	109.5
H(53B)-C(53A)-H(53C)	109.5	Si(5B)-C(53B)-H(53D)	109.5
Si(5B)-C(53B)-H(53E)	109.5	Si(5B)-C(53B)-H(53F)	109.5
H(53D)-C(53B)-H(53E)	109.5	H(53D)-C(53B)-H(53F)	109.5
H(53E)-C(53B)-H(53F)	109.5	Si(5A)-C(54A)-H(54A)	109.5
Si(5A)-C(54A)-H(54B)	109.5	Si(5A)-C(54A)-H(54C)	109.5
H(54A)-C(54A)-H(54B)	109.5	H(54A)-C(54A)-H(54C)	109.5
H(54B)-C(54A)-H(54C)	109.5	Si(5B)-C(54B)-H(54D)	109.5
Si(5B)-C(54B)-H(54E)	109.5	Si(5B)-C(54B)-H(54F)	109.5
H(54D)-C(54B)-H(54E)	109.5	H(54D)-C(54B)-H(54F)	109.5
H(54E)-C(54B)-H(54F)	109.5	H(4A)-O(4)-H(4B)	111(3)

Single crystal structure analysis of complex 3d

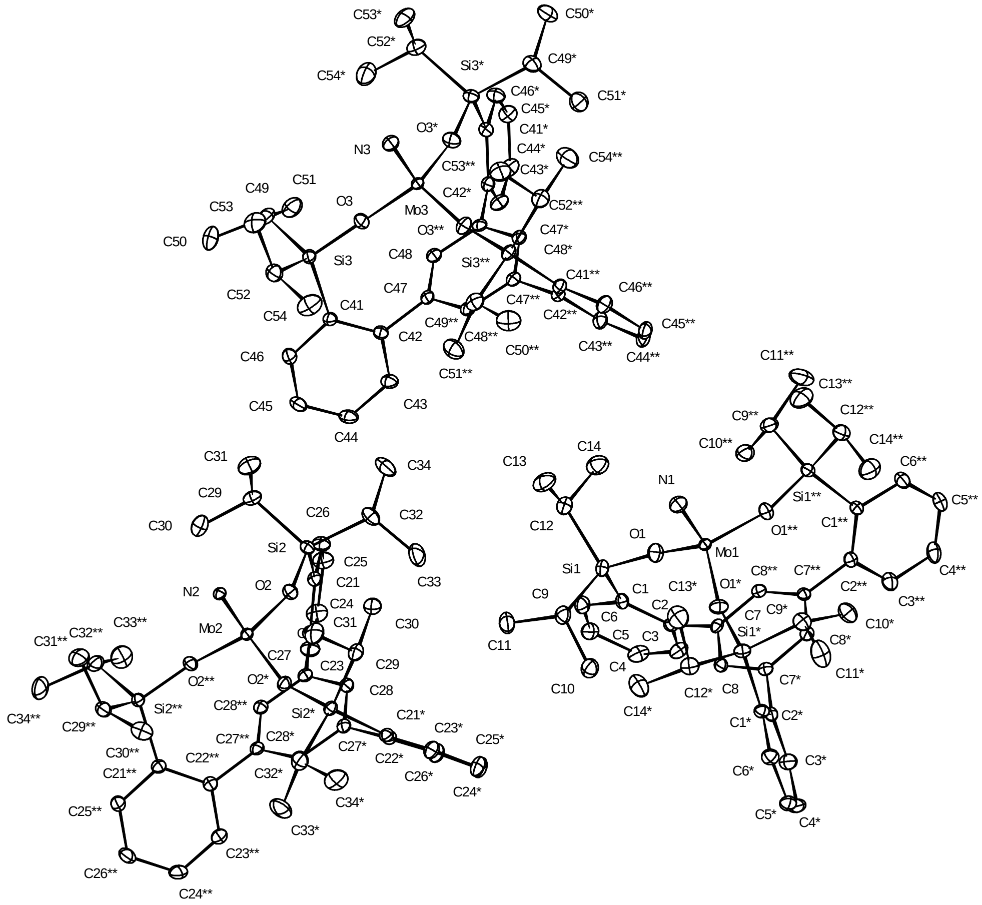

Figure S 6. The molecular structure of complex **3d**. H atoms have been removed for clarity.

X-ray Crystal Structure Analysis of Complex 3d:

C_{42} H_{57} Mo N O_3 Si_3, M_r = 804.09 g mol^{-1}, colourless plate, crystal size 0.101 x 0.064 x 0.017 mm^3, Trigonal, space group *P*3*c*1 [158], *a* = 20.9695(9) Å, *c* = 16.4975(10) Å, *V* = 6282.4(7) Å^3, *T* = 100(2) K, *Z* = 6, D_{calc} = 1.275 g·cm^3, λ = 0.71073 Å, μ(*Mo-K$_\alpha$*) = 0.436 mm^{-1}, Gaussian absorption correction ($T_{\min}$ = 0.95533, $T_{\max}$ = 0.99219), Bruker-AXS Kappa Mach3 with APEX-II detector and IµS microfocus Mo-anode X-ray source, 1.121 < θ < 31.524°, 206695 measured reflections, 13714 independent reflections, 11831 reflections with $I > 2\sigma(I)$, R_{int} = 0.0471. The structure was solved by *SHELXT* and refined by full-matrix least-squares (*SHELXL*) against F^2 to R_1 = 0.0253 [$I > 2\sigma(I)$], wR_2 = 0.0607 [all data], 463 parameters and 1 restraints.

Full .cif data for the compound are available under the CCDC number **CCDC-2265451**

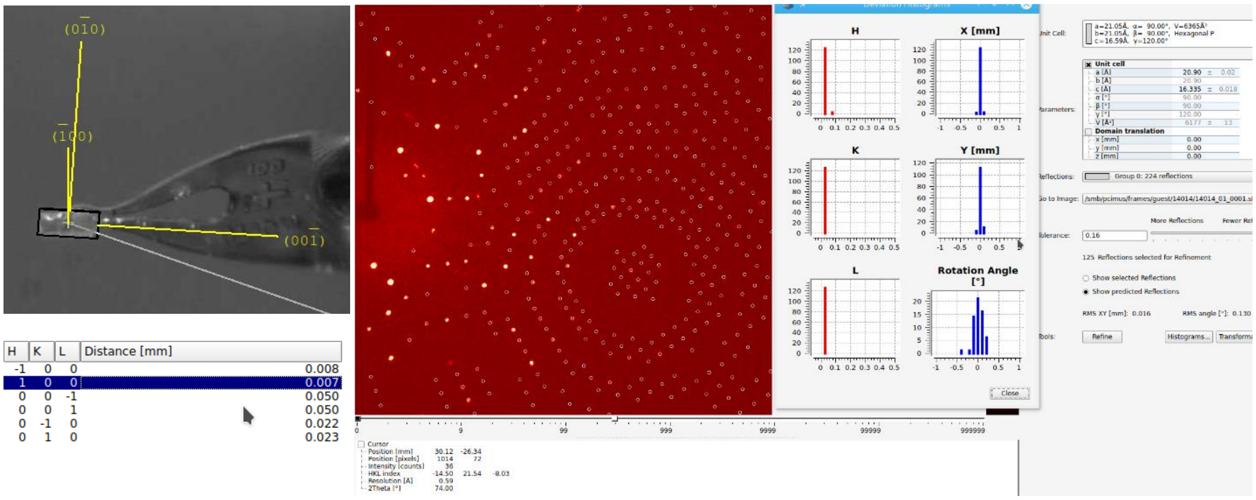

Figure S 7. Crystal faces and unit cell determination/refinement of complex **3d**.

INTENSITY STATISTICS FOR DATASET

Resolution	#Data	#Theory	%Complete	Redundancy	Mean I	Mean I/s	Rmerge	Rsigma
Inf - 2.97	114	114	100.0	41.68	87.72	110.81	0.0204	0.0065
2.97 - 1.94	259	259	100.0	48.96	37.57	111.79	0.0276	0.0064
1.94 - 1.51	374	374	100.0	51.67	29.50	96.07	0.0298	0.0065
1.51 - 1.31	373	373	100.0	51.16	24.47	88.06	0.0338	0.0070
1.31 - 1.18	387	387	100.0	51.00	17.18	73.05	0.0443	0.0083
1.18 - 1.09	395	395	100.0	46.28	17.40	67.99	0.0482	0.0096
1.09 - 1.03	331	331	100.0	37.13	12.70	47.56	0.0564	0.0125
1.03 - 0.97	436	436	100.0	30.74	10.23	38.69	0.0692	0.0163
0.97 - 0.93	344	344	100.0	26.91	9.14	33.03	0.0747	0.0189
0.93 - 0.89	400	400	100.0	24.11	9.12	30.13	0.0807	0.0209
0.89 - 0.86	366	366	100.0	22.34	9.63	28.37	0.0766	0.0218
0.86 - 0.83	402	402	100.0	22.16	7.81	24.47	0.0913	0.0253
0.83 - 0.81	321	321	100.0	21.00	7.68	22.89	0.0999	0.0281
0.81 - 0.79	333	333	100.0	20.88	8.42	24.23	0.0953	0.0268
0.79 - 0.77	384	384	100.0	20.06	5.70	18.27	0.1209	0.0364
0.77 - 0.75	401	401	100.0	19.57	5.21	16.39	0.1331	0.0409
0.75 - 0.73	485	485	100.0	18.83	5.48	16.55	0.1347	0.0417
0.73 - 0.72	233	233	100.0	18.21	5.26	15.61	0.1396	0.0448
0.72 - 0.70	535	535	100.0	18.03	4.45	13.48	0.1582	0.0524
0.70 - 0.69	314	314	100.0	17.41	3.78	11.38	0.1826	0.0630
0.69 - 0.68	258	329	78.4	5.98	4.89	7.76	0.1601	0.1121
0.78 - 0.68	2428	2499	97.2	16.96	4.86	14.16	0.1460	0.0536
Inf - 0.68	7445	7516	99.1	28.64	12.60	39.65	0.0490	0.0172

Table S 5. Crystal data and structure refinement of complex **3d**.

Identification code	14014	
Empirical formula	C_{42} H_{57} Mo N O_3 Si_3	
Color	colourless	
Formula weight	804.09 g·mol^{-1}	
Temperature	100(2) K	
Wavelength	0.71073 Å	
Crystal system	Trigonal	
Space group	*P*3*c*1, (no. 158)	
Unit cell dimensions	a = 20.9695(9) Å	α= 90°.
	b = 20.9695(9) Å	β= 90°.
	c = 16.4975(10) Å	γ = 120°.
Volume	6282.4(7) Å^3	
Z	6	
Density (calculated)	1.275 Mg·m^{-3}	
Absorption coefficient	0.436 mm^{-1}	
F(000)	2544 e	
Crystal size	0.101 x 0.064 x 0.017 mm^3	
θ range for data collection	1.121 to 31.524°.	
Index ranges	-30 ≤ h ≤ 30, -30≤ k ≤ 30, -24 ≤ l ≤ 24	
Reflections collected	206695	
Independent reflections	13714 [R_{int} = 0.0471]	
Reflections with I>2σ(I)	11831	
Completeness to θ = 25.242°	100.0 %	
Absorption correction	Gaussian	
Max. and min. transmission	0.99219 and 0.95533	
Refinement method	Full-matrix least-squares on F^2	
Data / restraints / parameters	13714 / 1 / 463	
Goodness-of-fit on F^2	1.029	
Final R indices [I>2σ(I)]	R_1 = 0.0253	wR^2 = 0.0560
R indices (all data)	R_1 = 0.0363	wR^2 = 0.0607
Absolute structure parameter	-0.013(6)	
Extinction coefficient	n/a	
Largest diff. peak and hole	0.421 and -0.357 e·Å^{-3}	

Table S 6. Bond lengths [Å] and angles [°] of complex **3d**.

Mo(1)-O(1)#1	1.8584(15)	Mo(1)-O(1)#2	1.8584(15)
Mo(1)-O(1)	1.8584(15)	Mo(1)-N(1)	1.644(3)
Si(1)-O(1)	1.6427(16)	Si(1)-C(1)	1.876(2)
Si(1)-C(9)	1.874(2)	Si(1)-C(12)	1.883(2)
C(1)-C(2)	1.407(3)	C(1)-C(6)	1.411(3)
C(2)-C(3)	1.399(3)	C(2)-C(7)	1.502(3)
C(3)-H(3)	0.9500	C(3)-C(4)	1.387(3)
C(4)-H(4)	0.9500	C(4)-C(5)	1.381(3)
C(5)-H(5)	0.9500	C(5)-C(6)	1.380(3)
C(6)-H(6)	0.9500	C(7)-C(8)#2	1.397(3)
C(7)-C(8)	1.394(3)	C(8)-H(8)	0.9500
C(9)-H(9)	1.0000	C(9)-C(10)	1.524(3)
C(9)-C(11)	1.532(3)	C(10)-H(10A)	0.9800
C(10)-H(10B)	0.9800	C(10)-H(10C)	0.9800
C(11)-H(11A)	0.9800	C(11)-H(11B)	0.9800
C(11)-H(11C)	0.9800	C(12)-H(12)	1.0000
C(12)-C(13)	1.538(3)	C(12)-C(14)	1.532(3)
C(13)-H(13A)	0.9800	C(13)-H(13B)	0.9800
C(13)-H(13C)	0.9800	C(14)-H(14A)	0.9800
C(14)-H(14B)	0.9800	C(14)-H(14C)	0.9800
Mo(2)-O(2)	1.8703(15)	Mo(2)-O(2)#3	1.8703(15)
Mo(2)-O(2)#4	1.8703(15)	Mo(2)-N(2)	1.664(3)
Si(2)-O(2)	1.6456(16)	Si(2)-C(21)	1.890(2)
Si(2)-C(29)	1.881(2)	Si(2)-C(32)	1.880(2)
C(21)-C(22)	1.412(3)	C(21)-C(26)	1.401(3)
C(22)-C(23)	1.400(3)	C(22)-C(27)	1.489(3)
C(23)-H(23)	0.9500	C(23)-C(24)	1.383(3)
C(24)-H(24)	0.9500	C(24)-C(25)	1.382(3)
C(25)-H(25)	0.9500	C(25)-C(26)	1.392(3)
C(26)-H(26)	0.9500	C(27)-C(28)	1.399(3)
C(27)-C(28)#3	1.388(3)	C(28)-H(28)	0.9500
C(29)-H(29)	1.0000	C(29)-C(30)	1.532(3)
C(29)-C(31)	1.536(3)	C(30)-H(30A)	0.9800
C(30)-H(30B)	0.9800	C(30)-H(30C)	0.9800

C(31)-H(31A)	0.9800	C(31)-H(31B)	0.9800
C(31)-H(31C)	0.9800	C(32)-H(32)	1.0000
C(32)-C(33)	1.530(4)	C(32)-C(34)	1.546(3)
C(33)-H(33A)	0.9800	C(33)-H(33B)	0.9800
C(33)-H(33C)	0.9800	C(34)-H(34A)	0.9800
C(34)-H(34B)	0.9800	C(34)-H(34C)	0.9800
Mo(3)-O(3)	1.8671(15)	Mo(3)-O(3)#5	1.8672(15)
Mo(3)-O(3)#6	1.8671(15)	Mo(3)-N(3)	1.651(3)
Si(3)-O(3)	1.6427(16)	Si(3)-C(41)	1.879(2)
Si(3)-C(49)	1.882(2)	Si(3)-C(52)	1.880(2)
C(41)-C(42)	1.407(3)	C(41)-C(46)	1.410(3)
C(42)-C(43)	1.401(3)	C(42)-C(47)	1.487(3)
C(43)-H(43)	0.9500	C(43)-C(44)	1.384(3)
C(44)-H(44)	0.9500	C(44)-C(45)	1.384(3)
C(45)-H(45)	0.9500	C(45)-C(46)	1.386(3)
C(46)-H(46)	0.9500	C(47)-C(48)#6	1.393(3)
C(47)-C(48)	1.395(3)	C(48)-H(48)	0.9500
C(49)-H(49)	1.0000	C(49)-C(50)	1.533(3)
C(49)-C(51)	1.531(3)	C(50)-H(50A)	0.9800
C(50)-H(50B)	0.9800	C(50)-H(50C)	0.9800
C(51)-H(51A)	0.9800	C(51)-H(51B)	0.9800
C(51)-H(51C)	0.9800	C(52)-H(52)	1.0000
C(52)-C(53)	1.534(3)	C(52)-C(54)	1.539(4)
C(53)-H(53A)	0.9800	C(53)-H(53B)	0.9800
C(53)-H(53C)	0.9800	C(54)-H(54A)	0.9800
C(54)-H(54B)	0.9800	C(54)-H(54C)	0.9800

O(1)#2-Mo(1)-O(1)	112.64(4)	O(1)#1-Mo(1)-O(1)	112.64(4)
O(1)#1-Mo(1)-O(1)#2	112.64(4)	N(1)-Mo(1)-O(1)#2	106.08(5)
N(1)-Mo(1)-O(1)#1	106.08(5)	N(1)-Mo(1)-O(1)	106.08(5)
O(1)-Si(1)-C(1)	109.01(9)	O(1)-Si(1)-C(9)	107.51(10)
O(1)-Si(1)-C(12)	108.05(9)	C(1)-Si(1)-C(12)	111.42(10)
C(9)-Si(1)-C(1)	109.55(10)	C(9)-Si(1)-C(12)	111.19(10)
Si(1)-O(1)-Mo(1)	163.70(10)	C(2)-C(1)-Si(1)	126.71(15)
C(2)-C(1)-C(6)	117.73(19)	C(6)-C(1)-Si(1)	115.44(16)
C(1)-C(2)-C(7)	124.43(18)	C(3)-C(2)-C(1)	119.72(19)

C(3)-C(2)-C(7)	115.84(18)	C(2)-C(3)-H(3)	119.6
C(4)-C(3)-C(2)	120.9(2)	C(4)-C(3)-H(3)	119.6
C(3)-C(4)-H(4)	119.9	C(5)-C(4)-C(3)	120.2(2)
C(5)-C(4)-H(4)	119.9	C(4)-C(5)-H(5)	120.3
C(6)-C(5)-C(4)	119.5(2)	C(6)-C(5)-H(5)	120.3
C(1)-C(6)-H(6)	119.0	C(5)-C(6)-C(1)	122.0(2)
C(5)-C(6)-H(6)	119.0	C(8)-C(7)-C(2)	120.43(18)
C(8)#2-C(7)-C(2)	120.13(18)	C(8)-C(7)-C(8)#2	119.1(2)
C(7)-C(8)-C(7)#1	120.9(2)	C(7)#1-C(8)-H(8)	119.5
C(7)-C(8)-H(8)	119.5	Si(1)-C(9)-H(9)	106.7
C(10)-C(9)-Si(1)	113.38(15)	C(10)-C(9)-H(9)	106.7
C(10)-C(9)-C(11)	110.3(2)	C(11)-C(9)-Si(1)	112.48(17)
C(11)-C(9)-H(9)	106.7	C(9)-C(10)-H(10A)	109.5
C(9)-C(10)-H(10B)	109.5	C(9)-C(10)-H(10C)	109.5
H(10A)-C(10)-H(10B)	109.5	H(10A)-C(10)-H(10C)	109.5
H(10B)-C(10)-H(10C)	109.5	C(9)-C(11)-H(11A)	109.5
C(9)-C(11)-H(11B)	109.5	C(9)-C(11)-H(11C)	109.5
H(11A)-C(11)-H(11B)	109.5	H(11A)-C(11)-H(11C)	109.5
H(11B)-C(11)-H(11C)	109.5	Si(1)-C(12)-H(12)	108.1
C(13)-C(12)-Si(1)	110.52(17)	C(13)-C(12)-H(12)	108.1
C(14)-C(12)-Si(1)	112.12(16)	C(14)-C(12)-H(12)	108.1
C(14)-C(12)-C(13)	109.8(2)	C(12)-C(13)-H(13A)	109.5
C(12)-C(13)-H(13B)	109.5	C(12)-C(13)-H(13C)	109.5
H(13A)-C(13)-H(13B)	109.5	H(13A)-C(13)-H(13C)	109.5
H(13B)-C(13)-H(13C)	109.5	C(12)-C(14)-H(14A)	109.5
C(12)-C(14)-H(14B)	109.5	C(12)-C(14)-H(14C)	109.5
H(14A)-C(14)-H(14B)	109.5	H(14A)-C(14)-H(14C)	109.5
H(14B)-C(14)-H(14C)	109.5	O(2)#4-Mo(2)-O(2)#3	113.16(4)
O(2)#3-Mo(2)-O(2)	113.16(4)	O(2)#4-Mo(2)-O(2)	113.16(4)
N(2)-Mo(2)-O(2)	105.47(5)	N(2)-Mo(2)-O(2)#3	105.47(5)
N(2)-Mo(2)-O(2)#4	105.47(5)	O(2)-Si(2)-C(21)	110.08(9)
O(2)-Si(2)-C(29)	106.63(9)	O(2)-Si(2)-C(32)	107.95(9)
C(29)-Si(2)-C(21)	109.00(10)	C(32)-Si(2)-C(21)	111.58(10)
C(32)-Si(2)-C(29)	111.48(10)	Si(2)-O(2)-Mo(2)	159.28(10)
C(22)-C(21)-Si(2)	127.49(15)	C(26)-C(21)-Si(2)	115.26(15)
C(26)-C(21)-C(22)	117.02(18)	C(21)-C(22)-C(27)	123.36(18)

C(23)-C(22)-C(21)	120.07(19)	C(23)-C(22)-C(27)	116.55(18)
C(22)-C(23)-H(23)	119.4	C(24)-C(23)-C(22)	121.3(2)
C(24)-C(23)-H(23)	119.4	C(23)-C(24)-H(24)	120.2
C(25)-C(24)-C(23)	119.6(2)	C(25)-C(24)-H(24)	120.2
C(24)-C(25)-H(25)	120.3	C(24)-C(25)-C(26)	119.5(2)
C(26)-C(25)-H(25)	120.3	C(21)-C(26)-H(26)	118.7
C(25)-C(26)-C(21)	122.5(2)	C(25)-C(26)-H(26)	118.7
C(28)-C(27)-C(22)	120.50(18)	C(28)#3-C(27)-C(22)	120.20(18)
C(28)#3-C(27)-C(28)	119.2(2)	C(27)#4-C(28)-C(27)	120.8(2)
C(27)#4-C(28)-H(28)	119.6	C(27)-C(28)-H(28)	119.6
Si(2)-C(29)-H(29)	108.4	C(30)-C(29)-Si(2)	110.03(15)
C(30)-C(29)-H(29)	108.4	C(30)-C(29)-C(31)	109.91(19)
C(31)-C(29)-Si(2)	111.55(16)	C(31)-C(29)-H(29)	108.4
C(29)-C(30)-H(30A)	109.5	C(29)-C(30)-H(30B)	109.5
C(29)-C(30)-H(30C)	109.5	H(30A)-C(30)-H(30B)	109.5
H(30A)-C(30)-H(30C)	109.5	H(30B)-C(30)-H(30C)	109.5
C(29)-C(31)-H(31A)	109.5	C(29)-C(31)-H(31B)	109.5
C(29)-C(31)-H(31C)	109.5	H(31A)-C(31)-H(31B)	109.5
H(31A)-C(31)-H(31C)	109.5	H(31B)-C(31)-H(31C)	109.5
Si(2)-C(32)-H(32)	106.8	C(33)-C(32)-Si(2)	112.99(16)
C(33)-C(32)-H(32)	106.8	C(33)-C(32)-C(34)	109.9(2)
C(34)-C(32)-Si(2)	112.99(17)	C(34)-C(32)-H(32)	106.8
C(32)-C(33)-H(33A)	109.5	C(32)-C(33)-H(33B)	109.5
C(32)-C(33)-H(33C)	109.5	H(33A)-C(33)-H(33B)	109.5
H(33A)-C(33)-H(33C)	109.5	H(33B)-C(33)-H(33C)	109.5
C(32)-C(34)-H(34A)	109.5	C(32)-C(34)-H(34B)	109.5
C(32)-C(34)-H(34C)	109.5	H(34A)-C(34)-H(34B)	109.5
H(34A)-C(34)-H(34C)	109.5	H(34B)-C(34)-H(34C)	109.5
O(3)#6-Mo(3)-O(3)	113.20(4)	O(3)#5-Mo(3)-O(3)	113.19(4)
O(3)#5-Mo(3)-O(3)#6	113.19(4)	N(3)-Mo(3)-O(3)	105.43(5)
N(3)-Mo(3)-O(3)#6	105.43(5)	N(3)-Mo(3)-O(3)#5	105.43(5)
O(3)-Si(3)-C(41)	109.71(9)	O(3)-Si(3)-C(49)	107.56(9)
O(3)-Si(3)-C(52)	108.17(9)	C(41)-Si(3)-C(49)	110.08(10)
C(41)-Si(3)-C(52)	109.64(10)	C(52)-Si(3)-C(49)	111.62(10)
Si(3)-O(3)-Mo(3)	169.46(11)	C(42)-C(41)-Si(3)	127.50(15)
C(42)-C(41)-C(46)	117.35(18)	C(46)-C(41)-Si(3)	115.14(15)

C(41)-C(42)-C(47)	123.97(18)	C(43)-C(42)-C(41)	120.09(18)
C(43)-C(42)-C(47)	115.93(17)	C(42)-C(43)-H(43)	119.5
C(44)-C(43)-C(42)	120.91(19)	C(44)-C(43)-H(43)	119.5
C(43)-C(44)-H(44)	120.0	C(45)-C(44)-C(43)	119.97(19)
C(45)-C(44)-H(44)	120.0	C(44)-C(45)-H(45)	120.3
C(44)-C(45)-C(46)	119.5(2)	C(46)-C(45)-H(45)	120.3
C(41)-C(46)-H(46)	118.9	C(45)-C(46)-C(41)	122.2(2)
C(45)-C(46)-H(46)	118.9	C(48)#6-C(47)-C(42)	120.05(19)
C(48)-C(47)-C(42)	120.49(18)	C(48)#6-C(47)-C(48)	119.2(2)
C(47)#5-C(48)-C(47)	120.8(2)	C(47)#5-C(48)-H(48)	119.6
C(47)-C(48)-H(48)	119.6	Si(3)-C(49)-H(49)	106.7
C(50)-C(49)-Si(3)	113.60(17)	C(50)-C(49)-H(49)	106.7
C(51)-C(49)-Si(3)	112.32(15)	C(51)-C(49)-H(49)	106.7
C(51)-C(49)-C(50)	110.4(2)	C(49)-C(50)-H(50A)	109.5
C(49)-C(50)-H(50B)	109.5	C(49)-C(50)-H(50C)	109.5
H(50A)-C(50)-H(50B)	109.5	H(50A)-C(50)-H(50C)	109.5
H(50B)-C(50)-H(50C)	109.5	C(49)-C(51)-H(51A)	109.5
C(49)-C(51)-H(51B)	109.5	C(49)-C(51)-H(51C)	109.5
H(51A)-C(51)-H(51B)	109.5	H(51A)-C(51)-H(51C)	109.5
H(51B)-C(51)-H(51C)	109.5	Si(3)-C(52)-H(52)	108.0
C(53)-C(52)-Si(3)	110.86(16)	C(53)-C(52)-H(52)	108.0
C(53)-C(52)-C(54)	110.5(2)	C(54)-C(52)-Si(3)	111.43(16)
C(54)-C(52)-H(52)	108.0	C(52)-C(53)-H(53A)	109.5
C(52)-C(53)-H(53B)	109.5	C(52)-C(53)-H(53C)	109.5
H(53A)-C(53)-H(53B)	109.5	H(53A)-C(53)-H(53C)	109.5
H(53B)-C(53)-H(53C)	109.5	C(52)-C(54)-H(54A)	109.5
C(52)-C(54)-H(54B)	109.5	C(52)-C(54)-H(54C)	109.5
H(54A)-C(54)-H(54B)	109.5	H(54A)-C(54)-H(54C)	109.5

Symmetry transformations used to generate equivalent atoms:
#1 -x+y+1,-x+2,z   #2 -y+2,x-y+1,z   #3 -x+y,-x+1,z
#4 -y+1,x-y+1,z   #5 -y+1,x-y,z   #6 -x+y+1,-x+1,z

Single crystal structure analysis of complex 3e · diethyl ether solvate

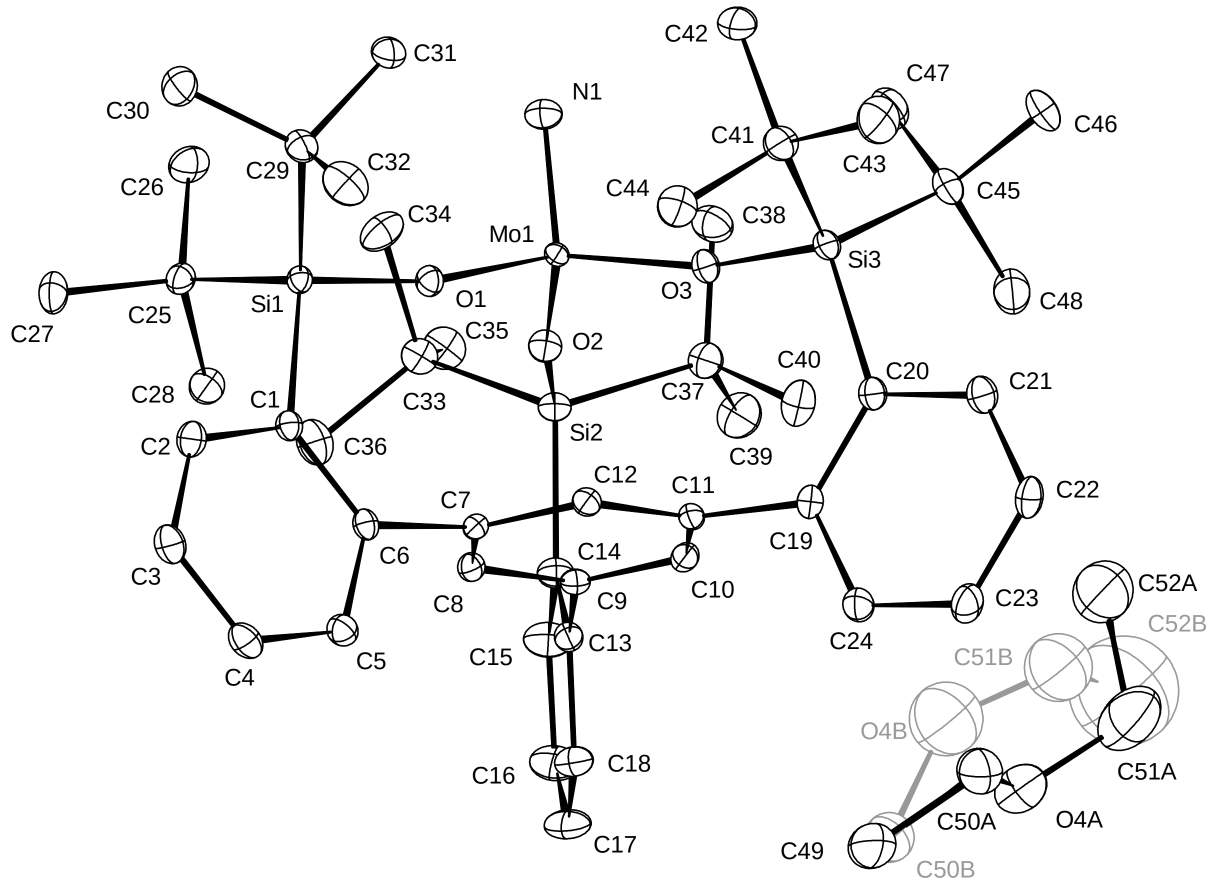

Figure S 8. The molecular structure of complex **3e · diethyl ether solvate**. H atoms have been removed for clarity. Main structure shown in black and disordered parts shown in grey.

X-ray Crystal Structure Analysis of Complex 3e · diethyl ether solvate:

C_{52} H_{79} Mo N O_4 Si_3, M_r = 962.37g mol^{-1}, yellow prism, crystal size 0.132 x 0.131 x 0.102 mm^3, Monoclinic, space group *C*2/*c* [15], *a* = 17.9114(13) Å, b = 15.0179(10) Å, *c* = 38.283(3) Å, β = 90.702(3)°, *V* = 10297.1(12) Å^3, *T* = 100(2) K, *Z* = 8, D_{calc} = 1.242 g·cm^3, λ = 0.71073 Å, $\mu(Mo\text{-}K_\alpha)$ = 0.367 mm^{-1}, Gaussian absorption correction ($T_{\min}$ = 0.96327, $T_{\max}$ = 0.97654), Bruker-AXS Kappa Mach3 with APEX-II detector and IµS microfocus Mo-anode X-ray source, 1.064 < θ < 30.998°, 307116 measured reflections, 16549 reflections, 15724 reflections with $I > 2\sigma(I)$, R_{int} = 0.0463. The structure was solved by *SHELXT* and refined by full-matrix least-squares (*SHELXL*) against F^2 to R_1 = 0.0375 [$I > 2\sigma(I)$], wR_2 = 0.0862 [all data], 588 and 2 restraints.

Full .cif data for the compound are available under the CCDC number **CCDC-2265453**

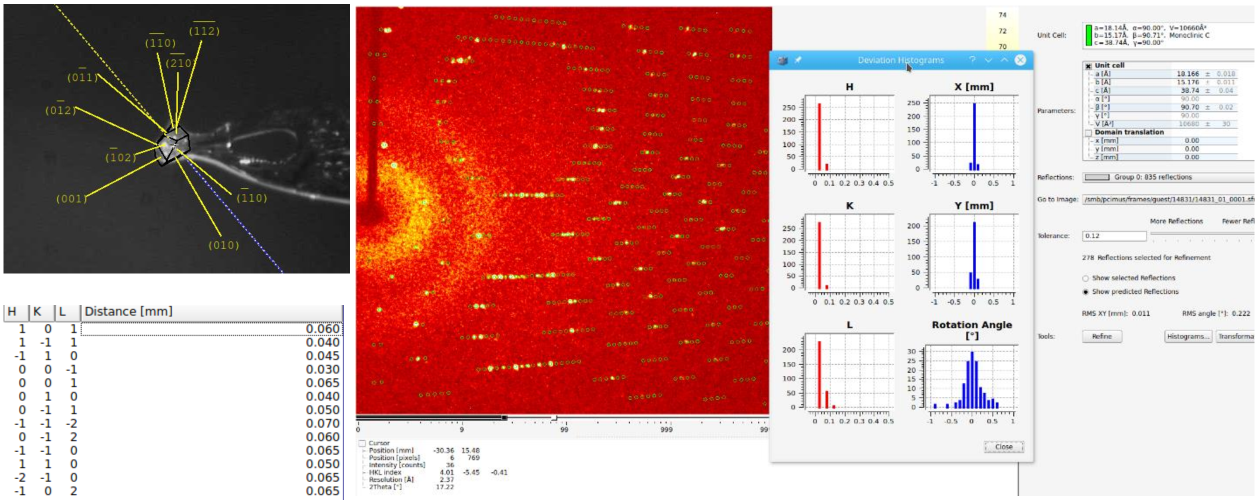

Figure S 9. Crystal faces and unit cell determination/refinement of complex **3e · diethyl ether solvate**.

INTENSITY STATISTICS FOR DATASET

Resolution	#Data	#Theory	%Complete	Redundancy	Mean I	Mean I/s	Rmerge	Rsigma
Inf - 2.87	257	258	99.6	20.74	74.37	108.67	0.0241	0.0068
2.87 - 1.90	604	604	100.0	26.43	31.45	101.44	0.0264	0.0069
1.90 - 1.50	856	856	100.0	27.76	23.86	92.08	0.0284	0.0074
1.50 - 1.31	840	840	100.0	27.85	20.58	82.73	0.0315	0.0082
1.31 - 1.19	838	840	99.8	27.17	15.32	69.54	0.0374	0.0096
1.19 - 1.10	888	888	100.0	25.83	13.69	62.24	0.0418	0.0110
1.10 - 1.03	913	913	100.0	21.78	10.91	49.97	0.0491	0.0139
1.03 - 0.98	800	800	100.0	19.45	9.74	43.16	0.0555	0.0164
0.98 - 0.94	806	806	100.0	18.12	8.37	37.14	0.0620	0.0189
0.94 - 0.90	895	895	100.0	16.66	8.03	34.01	0.0652	0.0211
0.90 - 0.87	858	858	100.0	15.59	7.72	31.04	0.0693	0.0232
0.87 - 0.84	898	898	100.0	15.40	7.77	30.23	0.0712	0.0240
0.84 - 0.82	724	724	100.0	14.83	7.47	28.67	0.0731	0.0258
0.82 - 0.79	1188	1188	100.0	14.33	6.83	26.25	0.0800	0.0288
0.79 - 0.77	905	905	100.0	13.90	5.88	23.05	0.0908	0.0332
0.77 - 0.76	494	494	100.0	13.81	5.11	20.65	0.1001	0.0371
0.76 - 0.74	1045	1045	100.0	13.25	4.94	19.44	0.1066	0.0398
0.74 - 0.72	1163	1163	100.0	12.91	4.68	17.95	0.1121	0.0431
0.72 - 0.71	642	642	100.0	12.63	4.66	17.05	0.1160	0.0447
0.71 - 0.70	673	673	100.0	12.40	4.38	16.34	0.1200	0.0478
0.70 - 0.69	672	672	100.0	12.30	4.06	15.02	0.1246	0.0512
0.79 - 0.69	5594	5594	100.0	13.04	4.85	18.64	0.1079	0.0415
Inf - 0.69	16959	16962	100.0	18.11	11.01	41.37	0.0459	0.0170

One reflection (OMIT 11 3 1) was omitted from the data set prior to the final refinement cycles because of high I/σI > 10. The crystal was twinned and it was refined accordingly (TWIN -1 0 0 0 -1 0 0.052 0 1) with a final BASF of 0.05925. A twofold positionally disordered diethyl ether solute is present in the asymmetric unit. It was refined with a fixed occupancy of 80:20% and the DFIX instruction was used to treat distances between C51B O4B and C51B C52B. Isotropic atomic displacement parameters were used for the minor constituents.

Table S 7. Crystal data and structure refinement of complex **3e · diethyl ether solvate**.

Identification code	14831	
Empirical formula	$C_{52} H_{79} Mo N O_4 Si_3$	
Color	yellow	
Formula weight	962.37 g·mol^{-1}	
Temperature	100(2) K	
Wavelength	0.71073 Å	
Crystal system	Monoclinic	
Space group	*C*2/*c*, (no. 15)	
Unit cell dimensions	a = 17.9114(13) Å	α= 90°.
	b = 15.0179(10) Å	β= 90.702(3)°.
	c = 38.283(3) Å	γ = 90°.
Volume	10297.1(12) Å^3	
Z	8	
Density (calculated)	1.242 Mg·m^{-3}	
Absorption coefficient	0.367 mm^{-1}	
F(000)	4112 e	
Crystal size	0.132 x 0.131 x 0.102 mm^3	
θ range for data collection	1.064 to 30.998°.	
Index ranges	-25 ≤ h ≤ 25, -21≤ k ≤ 21, -55 ≤ l ≤ 55	
Reflections collected	307116	
Independent reflections	16549 [R_{int} = 0.0463]	
Reflections with I>2σ(I)	15724	
Completeness to θ = 25.242°	100.0 %	
Absorption correction	Gaussian	
Max. and min. transmission	0.97654 and 0.96327	
Refinement method	Full-matrix least-squares on F^2	
Data / restraints / parameters	16549 / 2 / 588	
Goodness-of-fit on F^2	1.134	
Final R indices [I>2σ(I)]	R_1 = 0.0375	wR^2 = 0.0850
R indices (all data)	R_1 = 0.0398	wR^2 = 0.0862
Extinction coefficient	n/a	
Largest diff. peak and hole	1.258 and -0.549 e·Å^{-3}	

Table S 8. Bond lengths [Å] and angles [°] of complex **3e · diethyl ether solvate**.

Mo(1)-O(1)	1.8585(13)	Mo(1)-O(2)	1.8640(13)
Mo(1)-O(3)	1.8695(13)	Mo(1)-N(1)	1.6497(16)
Si(1)-O(1)	1.6534(13)	Si(1)-C(1)	1.8792(18)
Si(1)-C(25)	1.9042(19)	Si(1)-C(29)	1.9099(19)
Si(2)-O(2)	1.6513(14)	Si(2)-C(14)	1.8819(19)
Si(2)-C(33)	1.9061(19)	Si(2)-C(37)	1.899(2)
Si(3)-O(3)	1.6504(14)	Si(3)-C(20)	1.8844(19)
Si(3)-C(41)	1.901(2)	Si(3)-C(45)	1.912(2)
C(1)-C(2)	1.410(2)	C(1)-C(6)	1.414(2)
C(2)-H(2)	0.9500	C(2)-C(3)	1.388(3)
C(3)-H(3)	0.9500	C(3)-C(4)	1.386(3)
C(4)-H(4)	0.9500	C(4)-C(5)	1.390(2)
C(5)-H(5)	0.9500	C(5)-C(6)	1.399(2)
C(6)-C(7)	1.502(2)	C(7)-C(8)	1.395(2)
C(7)-C(12)	1.399(2)	C(8)-H(8)	0.9500
C(8)-C(9)	1.399(2)	C(9)-C(10)	1.397(2)
C(9)-C(13)	1.502(2)	C(10)-H(10)	0.9500
C(10)-C(11)	1.395(2)	C(11)-C(12)	1.398(2)
C(11)-C(19)	1.502(2)	C(12)-H(12)	0.9500
C(13)-C(14)	1.410(3)	C(13)-C(18)	1.401(2)
C(14)-C(15)	1.411(3)	C(15)-H(15)	0.9500
C(15)-C(16)	1.387(3)	C(16)-H(16)	0.9500
C(16)-C(17)	1.385(3)	C(17)-H(17)	0.9500
C(17)-C(18)	1.387(3)	C(18)-H(18)	0.9500
C(19)-C(20)	1.409(3)	C(19)-C(24)	1.398(3)
C(20)-C(21)	1.410(2)	C(21)-H(21)	0.9500
C(21)-C(22)	1.388(3)	C(22)-H(22)	0.9500
C(22)-C(23)	1.384(3)	C(23)-H(23)	0.9500
C(23)-C(24)	1.388(3)	C(24)-H(24)	0.9500
C(25)-C(26)	1.536(3)	C(25)-C(27)	1.534(3)
C(25)-C(28)	1.538(3)	C(26)-H(26A)	0.9800
C(26)-H(26B)	0.9800	C(26)-H(26C)	0.9800
C(27)-H(27A)	0.9800	C(27)-H(27B)	0.9800
C(27)-H(27C)	0.9800	C(28)-H(28A)	0.9800

C(28)-H(28B)	0.9800	C(28)-H(28C)	0.9800
C(29)-C(30)	1.535(3)	C(29)-C(31)	1.540(3)
C(29)-C(32)	1.536(3)	C(30)-H(30A)	0.9800
C(30)-H(30B)	0.9800	C(30)-H(30C)	0.9800
C(31)-H(31A)	0.9800	C(31)-H(31B)	0.9800
C(31)-H(31C)	0.9800	C(32)-H(32A)	0.9800
C(32)-H(32B)	0.9800	C(32)-H(32C)	0.9800
C(33)-C(34)	1.542(3)	C(33)-C(35)	1.538(3)
C(33)-C(36)	1.537(3)	C(34)-H(34A)	0.9800
C(34)-H(34B)	0.9800	C(34)-H(34C)	0.9800
C(35)-H(35A)	0.9800	C(35)-H(35B)	0.9800
C(35)-H(35C)	0.9800	C(36)-H(36A)	0.9800
C(36)-H(36B)	0.9800	C(36)-H(36C)	0.9800
C(37)-C(38)	1.538(3)	C(37)-C(39)	1.541(3)
C(37)-C(40)	1.537(3)	C(38)-H(38A)	0.9800
C(38)-H(38B)	0.9800	C(38)-H(38C)	0.9800
C(39)-H(39A)	0.9800	C(39)-H(39B)	0.9800
C(39)-H(39C)	0.9800	C(40)-H(40A)	0.9800
C(40)-H(40B)	0.9800	C(40)-H(40C)	0.9800
C(41)-C(42)	1.537(3)	C(41)-C(43)	1.538(3)
C(41)-C(44)	1.542(3)	C(42)-H(42A)	0.9800
C(42)-H(42B)	0.9800	C(42)-H(42C)	0.9800
C(43)-H(43A)	0.9800	C(43)-H(43B)	0.9800
C(43)-H(43C)	0.9800	C(44)-H(44A)	0.9800
C(44)-H(44B)	0.9800	C(44)-H(44C)	0.9800
C(45)-C(46)	1.538(3)	C(45)-C(47)	1.544(3)
C(45)-C(48)	1.537(3)	C(46)-H(46A)	0.9800
C(46)-H(46B)	0.9800	C(46)-H(46C)	0.9800
C(47)-H(47A)	0.9800	C(47)-H(47B)	0.9800
C(47)-H(47C)	0.9800	C(48)-H(48A)	0.9800
C(48)-H(48B)	0.9800	C(48)-H(48C)	0.9800
O(4A)-C(50A)	1.414(4)	O(4A)-C(51A)	1.473(5)
O(4B)-C(50B)	1.54(2)	O(4B)-C(51B)	1.387(10)
C(49)-H(49A)	0.9800	C(49)-H(49B)	0.9800
C(49)-H(49C)	0.9800	C(49)-H(49D)	0.9800
C(49)-H(49E)	0.9800	C(49)-H(49F)	0.9800

C(49)-C(50A)	1.493(4)	C(49)-C(50B)	1.189(14)
C(50A)-H(50A)	0.9900	C(50A)-H(50B)	0.9900
C(50B)-H(50C)	0.9900	C(50B)-H(50D)	0.9900
C(51A)-H(51A)	0.9900	C(51A)-H(51B)	0.9900
C(51A)-C(52A)	1.470(5)	C(51B)-H(51C)	0.9900
C(51B)-H(51D)	0.9900	C(51B)-C(52B)	1.5000(11)
C(52A)-H(52A)	0.9800	C(52A)-H(52B)	0.9800
C(52A)-H(52C)	0.9800	C(52B)-H(52D)	0.9800
C(52B)-H(52E)	0.9800	C(52B)-H(52F)	0.9800
O(1)-Mo(1)-O(2)	111.97(6)	O(1)-Mo(1)-O(3)	113.80(6)
O(2)-Mo(1)-O(3)	112.91(6)	N(1)-Mo(1)-O(1)	105.14(7)
N(1)-Mo(1)-O(2)	106.32(7)	N(1)-Mo(1)-O(3)	105.89(7)
O(1)-Si(1)-C(1)	106.06(7)	O(1)-Si(1)-C(25)	106.26(8)
O(1)-Si(1)-C(29)	106.89(8)	C(1)-Si(1)-C(25)	107.80(8)
C(1)-Si(1)-C(29)	112.72(8)	C(25)-Si(1)-C(29)	116.43(8)
O(2)-Si(2)-C(14)	107.59(8)	O(2)-Si(2)-C(33)	107.81(8)
O(2)-Si(2)-C(37)	105.45(8)	C(14)-Si(2)-C(33)	111.28(9)
C(14)-Si(2)-C(37)	108.10(9)	C(37)-Si(2)-C(33)	116.17(9)
O(3)-Si(3)-C(20)	107.69(8)	O(3)-Si(3)-C(41)	106.00(8)
O(3)-Si(3)-C(45)	107.32(8)	C(20)-Si(3)-C(41)	108.77(9)
C(20)-Si(3)-C(45)	109.98(9)	C(41)-Si(3)-C(45)	116.68(9)
Si(1)-O(1)-Mo(1)	165.60(8)	Si(2)-O(2)-Mo(1)	168.14(9)
Si(3)-O(3)-Mo(1)	165.60(9)	C(2)-C(1)-Si(1)	116.28(13)
C(2)-C(1)-C(6)	117.40(16)	C(6)-C(1)-Si(1)	126.24(13)
C(1)-C(2)-H(2)	118.9	C(3)-C(2)-C(1)	122.28(16)
C(3)-C(2)-H(2)	118.9	C(2)-C(3)-H(3)	120.2
C(4)-C(3)-C(2)	119.66(17)	C(4)-C(3)-H(3)	120.2
C(3)-C(4)-H(4)	120.3	C(3)-C(4)-C(5)	119.41(17)
C(5)-C(4)-H(4)	120.3	C(4)-C(5)-H(5)	119.2
C(4)-C(5)-C(6)	121.59(16)	C(6)-C(5)-H(5)	119.2
C(1)-C(6)-C(7)	125.40(15)	C(5)-C(6)-C(1)	119.66(16)
C(5)-C(6)-C(7)	114.92(15)	C(8)-C(7)-C(6)	121.02(15)
C(8)-C(7)-C(12)	118.87(15)	C(12)-C(7)-C(6)	119.67(15)
C(7)-C(8)-H(8)	119.3	C(7)-C(8)-C(9)	121.30(16)
C(9)-C(8)-H(8)	119.3	C(8)-C(9)-C(13)	119.92(16)

C(10)-C(9)-C(8)	118.76(16)	C(10)-C(9)-C(13)	120.93(16)
C(9)-C(10)-H(10)	119.5	C(11)-C(10)-C(9)	120.99(16)
C(11)-C(10)-H(10)	119.5	C(10)-C(11)-C(12)	119.26(16)
C(10)-C(11)-C(19)	120.63(16)	C(12)-C(11)-C(19)	119.77(16)
C(7)-C(12)-H(12)	119.6	C(11)-C(12)-C(7)	120.81(16)
C(11)-C(12)-H(12)	119.6	C(14)-C(13)-C(9)	125.01(16)
C(18)-C(13)-C(9)	115.72(16)	C(18)-C(13)-C(14)	119.27(17)
C(13)-C(14)-Si(2)	127.44(14)	C(13)-C(14)-C(15)	117.27(17)
C(15)-C(14)-Si(2)	115.21(14)	C(14)-C(15)-H(15)	118.5
C(16)-C(15)-C(14)	122.94(19)	C(16)-C(15)-H(15)	118.5
C(15)-C(16)-H(16)	120.5	C(17)-C(16)-C(15)	118.92(19)
C(17)-C(16)-H(16)	120.5	C(16)-C(17)-H(17)	120.2
C(16)-C(17)-C(18)	119.66(18)	C(18)-C(17)-H(17)	120.2
C(13)-C(18)-H(18)	119.0	C(17)-C(18)-C(13)	121.93(18)
C(17)-C(18)-H(18)	119.0	C(20)-C(19)-C(11)	124.61(16)
C(24)-C(19)-C(11)	115.76(16)	C(24)-C(19)-C(20)	119.62(17)
C(19)-C(20)-Si(3)	127.10(14)	C(19)-C(20)-C(21)	117.36(17)
C(21)-C(20)-Si(3)	115.53(14)	C(20)-C(21)-H(21)	118.7
C(22)-C(21)-C(20)	122.51(18)	C(22)-C(21)-H(21)	118.7
C(21)-C(22)-H(22)	120.4	C(23)-C(22)-C(21)	119.25(18)
C(23)-C(22)-H(22)	120.4	C(22)-C(23)-H(23)	120.2
C(22)-C(23)-C(24)	119.64(18)	C(24)-C(23)-H(23)	120.2
C(19)-C(24)-H(24)	119.2	C(23)-C(24)-C(19)	121.60(18)
C(23)-C(24)-H(24)	119.2	C(26)-C(25)-Si(1)	110.78(13)
C(26)-C(25)-C(28)	108.56(16)	C(27)-C(25)-Si(1)	113.78(13)
C(27)-C(25)-C(26)	109.45(16)	C(27)-C(25)-C(28)	107.65(16)
C(28)-C(25)-Si(1)	106.42(12)	C(25)-C(26)-H(26A)	109.5
C(25)-C(26)-H(26B)	109.5	C(25)-C(26)-H(26C)	109.5
H(26A)-C(26)-H(26B)	109.5	H(26A)-C(26)-H(26C)	109.5
H(26B)-C(26)-H(26C)	109.5	C(25)-C(27)-H(27A)	109.5
C(25)-C(27)-H(27B)	109.5	C(25)-C(27)-H(27C)	109.5
H(27A)-C(27)-H(27B)	109.5	H(27A)-C(27)-H(27C)	109.5
H(27B)-C(27)-H(27C)	109.5	C(25)-C(28)-H(28A)	109.5
C(25)-C(28)-H(28B)	109.5	C(25)-C(28)-H(28C)	109.5
H(28A)-C(28)-H(28B)	109.5	H(28A)-C(28)-H(28C)	109.5
H(28B)-C(28)-H(28C)	109.5	C(30)-C(29)-Si(1)	113.84(13)

C(30)-C(29)-C(31)	107.84(16)	C(30)-C(29)-C(32)	107.67(17)
C(31)-C(29)-Si(1)	108.42(12)	C(32)-C(29)-Si(1)	110.59(14)
C(32)-C(29)-C(31)	108.32(16)	C(29)-C(30)-H(30A)	109.5
C(29)-C(30)-H(30B)	109.5	C(29)-C(30)-H(30C)	109.5
H(30A)-C(30)-H(30B)	109.5	H(30A)-C(30)-H(30C)	109.5
H(30B)-C(30)-H(30C)	109.5	C(29)-C(31)-H(31A)	109.5
C(29)-C(31)-H(31B)	109.5	C(29)-C(31)-H(31C)	109.5
H(31A)-C(31)-H(31B)	109.5	H(31A)-C(31)-H(31C)	109.5
H(31B)-C(31)-H(31C)	109.5	C(29)-C(32)-H(32A)	109.5
C(29)-C(32)-H(32B)	109.5	C(29)-C(32)-H(32C)	109.5
H(32A)-C(32)-H(32B)	109.5	H(32A)-C(32)-H(32C)	109.5
H(32B)-C(32)-H(32C)	109.5	C(34)-C(33)-Si(2)	108.80(13)
C(35)-C(33)-Si(2)	113.15(14)	C(35)-C(33)-C(34)	107.82(16)
C(36)-C(33)-Si(2)	110.37(13)	C(36)-C(33)-C(34)	108.50(17)
C(36)-C(33)-C(35)	108.07(16)	C(33)-C(34)-H(34A)	109.5
C(33)-C(34)-H(34B)	109.5	C(33)-C(34)-H(34C)	109.5
H(34A)-C(34)-H(34B)	109.5	H(34A)-C(34)-H(34C)	109.5
H(34B)-C(34)-H(34C)	109.5	C(33)-C(35)-H(35A)	109.5
C(33)-C(35)-H(35B)	109.5	C(33)-C(35)-H(35C)	109.5
H(35A)-C(35)-H(35B)	109.5	H(35A)-C(35)-H(35C)	109.5
H(35B)-C(35)-H(35C)	109.5	C(33)-C(36)-H(36A)	109.5
C(33)-C(36)-H(36B)	109.5	C(33)-C(36)-H(36C)	109.5
H(36A)-C(36)-H(36B)	109.5	H(36A)-C(36)-H(36C)	109.5
H(36B)-C(36)-H(36C)	109.5	C(38)-C(37)-Si(2)	110.03(13)
C(38)-C(37)-C(39)	109.03(17)	C(39)-C(37)-Si(2)	114.93(15)
C(40)-C(37)-Si(2)	106.35(14)	C(40)-C(37)-C(38)	108.89(17)
C(40)-C(37)-C(39)	107.42(17)	C(37)-C(38)-H(38A)	109.5
C(37)-C(38)-H(38B)	109.5	C(37)-C(38)-H(38C)	109.5
H(38A)-C(38)-H(38B)	109.5	H(38A)-C(38)-H(38C)	109.5
H(38B)-C(38)-H(38C)	109.5	C(37)-C(39)-H(39A)	109.5
C(37)-C(39)-H(39B)	109.5	C(37)-C(39)-H(39C)	109.5
H(39A)-C(39)-H(39B)	109.5	H(39A)-C(39)-H(39C)	109.5
H(39B)-C(39)-H(39C)	109.5	C(37)-C(40)-H(40A)	109.5
C(37)-C(40)-H(40B)	109.5	C(37)-C(40)-H(40C)	109.5
H(40A)-C(40)-H(40B)	109.5	H(40A)-C(40)-H(40C)	109.5
H(40B)-C(40)-H(40C)	109.5	C(42)-C(41)-Si(3)	109.85(14)

C(42)-C(41)-C(43)	108.80(17)	C(42)-C(41)-C(44)	109.49(17)
C(43)-C(41)-Si(3)	115.02(14)	C(43)-C(41)-C(44)	106.98(18)
C(44)-C(41)-Si(3)	106.55(13)	C(41)-C(42)-H(42A)	109.5
C(41)-C(42)-H(42B)	109.5	C(41)-C(42)-H(42C)	109.5
H(42A)-C(42)-H(42B)	109.5	H(42A)-C(42)-H(42C)	109.5
H(42B)-C(42)-H(42C)	109.5	C(41)-C(43)-H(43A)	109.5
C(41)-C(43)-H(43B)	109.5	C(41)-C(43)-H(43C)	109.5
H(43A)-C(43)-H(43B)	109.5	H(43A)-C(43)-H(43C)	109.5
H(43B)-C(43)-H(43C)	109.5	C(41)-C(44)-H(44A)	109.5
C(41)-C(44)-H(44B)	109.5	C(41)-C(44)-H(44C)	109.5
H(44A)-C(44)-H(44B)	109.5	H(44A)-C(44)-H(44C)	109.5
H(44B)-C(44)-H(44C)	109.5	C(46)-C(45)-Si(3)	113.69(16)
C(46)-C(45)-C(47)	107.89(18)	C(47)-C(45)-Si(3)	109.03(14)
C(48)-C(45)-Si(3)	109.74(14)	C(48)-C(45)-C(46)	107.70(17)
C(48)-C(45)-C(47)	108.66(19)	C(45)-C(46)-H(46A)	109.5
C(45)-C(46)-H(46B)	109.5	C(45)-C(46)-H(46C)	109.5
H(46A)-C(46)-H(46B)	109.5	H(46A)-C(46)-H(46C)	109.5
H(46B)-C(46)-H(46C)	109.5	C(45)-C(47)-H(47A)	109.5
C(45)-C(47)-H(47B)	109.5	C(45)-C(47)-H(47C)	109.5
H(47A)-C(47)-H(47B)	109.5	H(47A)-C(47)-H(47C)	109.5
H(47B)-C(47)-H(47C)	109.5	C(45)-C(48)-H(48A)	109.5
C(45)-C(48)-H(48B)	109.5	C(45)-C(48)-H(48C)	109.5
H(48A)-C(48)-H(48B)	109.5	H(48A)-C(48)-H(48C)	109.5
H(48B)-C(48)-H(48C)	109.5	C(50A)-O(4A)-C(51A)	110.8(3)
C(51B)-O(4B)-C(50B)	136(2)	H(49A)-C(49)-H(49B)	109.5
H(49A)-C(49)-H(49C)	109.5	H(49B)-C(49)-H(49C)	109.5
H(49D)-C(49)-H(49E)	109.5	H(49D)-C(49)-H(49F)	109.5
H(49E)-C(49)-H(49F)	109.5	C(50A)-C(49)-H(49A)	109.5
C(50A)-C(49)-H(49B)	109.5	C(50A)-C(49)-H(49C)	109.5
C(50B)-C(49)-H(49D)	109.5	C(50B)-C(49)-H(49E)	109.5
C(50B)-C(49)-H(49F)	109.5	O(4A)-C(50A)-C(49)	110.0(3)
O(4A)-C(50A)-H(50A)	109.7	O(4A)-C(50A)-H(50B)	109.7
C(49)-C(50A)-H(50A)	109.7	C(49)-C(50A)-H(50B)	109.7
H(50A)-C(50A)-H(50B)	108.2	O(4B)-C(50B)-H(50C)	108.7
O(4B)-C(50B)-H(50D)	108.7	C(49)-C(50B)-O(4B)	114.4(12)
C(49)-C(50B)-H(50C)	108.7	C(49)-C(50B)-H(50D)	108.7

H(50C)-C(50B)-H(50D)	107.6	O(4A)-C(51A)-H(51A)	109.1
O(4A)-C(51A)-H(51B)	109.1	H(51A)-C(51A)-H(51B)	107.9
C(52A)-C(51A)-O(4A)	112.3(3)	C(52A)-C(51A)-H(51A)	109.1
C(52A)-C(51A)-H(51B)	109.1	O(4B)-C(51B)-H(51C)	109.8
O(4B)-C(51B)-H(51D)	109.8	O(4B)-C(51B)-C(52B)	109(3)
H(51C)-C(51B)-H(51D)	108.2	C(52B)-C(51B)-H(51C)	109.8
C(52B)-C(51B)-H(51D)	109.8	C(51A)-C(52A)-H(52A)	109.5
C(51A)-C(52A)-H(52B)	109.5	C(51A)-C(52A)-H(52C)	109.5
H(52A)-C(52A)-H(52B)	109.5	H(52A)-C(52A)-H(52C)	109.5
H(52B)-C(52A)-H(52C)	109.5	C(51B)-C(52B)-H(52D)	109.5
C(51B)-C(52B)-H(52E)	109.5	C(51B)-C(52B)-H(52F)	109.5
H(52D)-C(52B)-H(52E)	109.5	H(52D)-C(52B)-H(52F)	109.5
H(52E)-C(52B)-H(52F)	109.5		

Single crystal structure analysis of complex 8

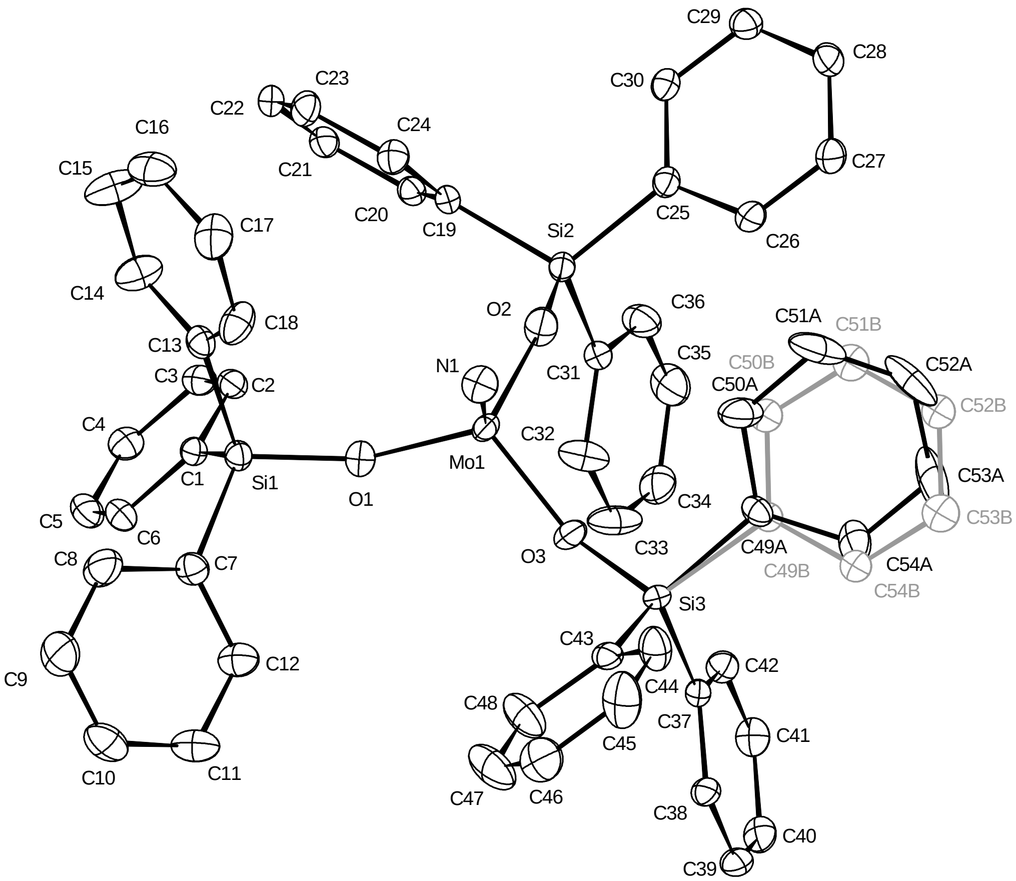

Figure S 10. The molecular structure of complex **8**. H atoms have been removed for clarity. Main structure shown in black and disordered parts shown in grey.

X-ray Crystal Structure Analysis of Complex 8:

C_{54} H_{45} Mo N O_3 Si_3, M_r = 936.12 g mol^{-1}, pale yellow prism, crystal size 0.094 x 0.080 x 0.041 mm^3, Triclinic, space group *P*-1 [2], *a* = 9.8873(6) Å, b = 13.6273(8) Å, *c* = 18.3370(11) Å, α = 71.713(2)°, β = 81.406(2)°, γ = 78.354(2)°, *V* = 2287.7(2) Å^3, *T* = 100(2) K, *Z* = 2, D_{calc} = 1.359 g·cm^3, λ = 0.71073 Å, μ(*Mo-K*$_\alpha$) = 0.411 mm^{-1}, Gaussian absorption correction ($T_{\min}$ = 0.97152, $T_{\max}$ = 0.98858), Bruker-AXS Kappa Mach3 with APEX-II detector and IμS microfocus Mo-anode X-ray source, 1.175 < θ < 32.576°, 112568 measured reflections, 16644 independent reflections, 14361 reflections with $I > 2\sigma(I)$, R_{int} = 0.0406. The structure was solved by *SHELXT* and refined by full-matrix least-squares (*SHELXL*) against F^2 to R_1 = 0.0442 [$I > 2\sigma(I)$], wR_2 = 0.1078 [all data], 571 parameters and 0 restraints.

Full .cif data for the compound are available under the CCDC number **CCDC-2265449**

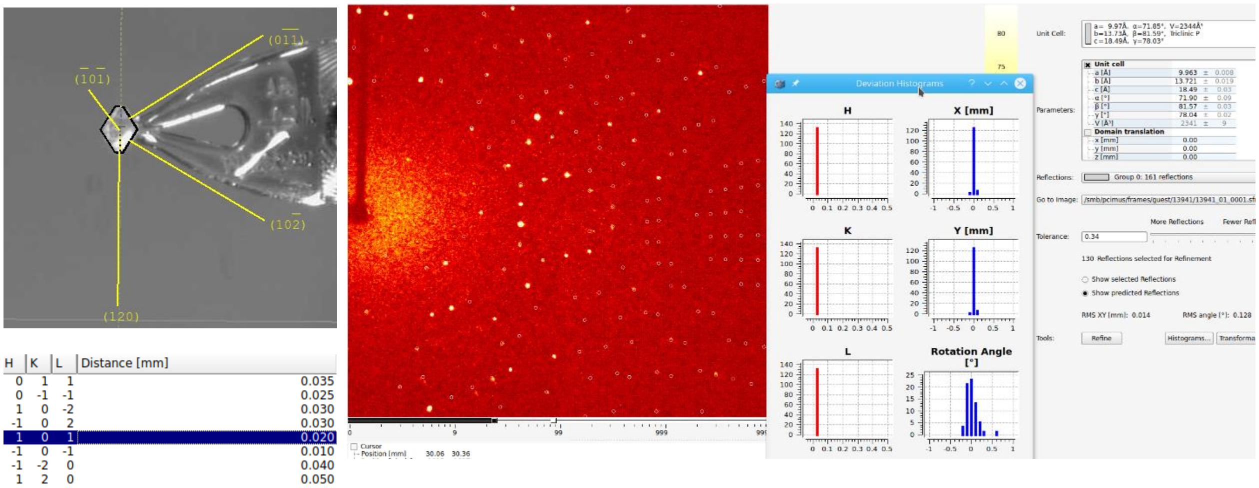

Figure S 11. Crystal faces and unit cell determination/refinement of complex **8**.

INTENSITY STATISTICS FOR DATASET

Resolution	#Data	#Theory	%Complete	Redundancy	Mean I	Mean I/s	Rmerge	Rsigma
Inf - 2.42	334	334	100.0	10.82	75.11	90.08	0.0204	0.0088
2.42 - 1.62	788	788	100.0	11.87	30.94	77.27	0.0237	0.0098
1.62 - 1.29	1116	1116	100.0	11.90	23.04	68.23	0.0269	0.0110
1.29 - 1.12	1178	1178	100.0	11.29	14.68	52.30	0.0341	0.0136
1.12 - 1.02	1099	1099	100.0	8.87	10.95	38.38	0.0443	0.0194
1.02 - 0.95	1083	1083	100.0	7.25	8.78	29.38	0.0518	0.0254
0.95 - 0.89	1191	1191	100.0	6.21	7.23	22.86	0.0614	0.0324
0.89 - 0.85	1019	1019	100.0	5.69	6.41	19.53	0.0711	0.0385
0.85 - 0.81	1212	1212	100.0	5.43	5.98	17.95	0.0759	0.0436
0.81 - 0.78	1105	1105	100.0	5.20	5.02	15.11	0.0866	0.0523
0.78 - 0.75	1242	1242	100.0	5.01	4.52	13.30	0.0974	0.0606
0.75 - 0.73	963	963	100.0	4.75	3.71	10.97	0.1157	0.0761
0.73 - 0.71	1051	1051	100.0	4.61	3.22	9.62	0.1341	0.0888
0.71 - 0.69	1203	1204	99.9	4.53	2.86	8.43	0.1481	0.1009
0.69 - 0.67	1347	1349	99.9	4.31	2.88	7.94	0.1509	0.1083
0.67 - 0.66	713	717	99.4	4.16	2.46	6.90	0.1723	0.1304
0.66 - 0.64	1631	1642	99.3	4.07	2.27	6.28	0.1826	0.1420
0.64 - 0.63	851	862	98.7	3.87	2.06	5.65	0.2099	0.1627
0.63 - 0.62	924	938	98.5	3.82	1.88	5.05	0.2295	0.1810
0.62 - 0.61	1001	1014	98.7	3.73	1.64	4.50	0.2514	0.2111
0.61 - 0.60	1064	1094	97.3	3.57	1.46	3.81	0.2835	0.2497
0.70 - 0.60	8134	8220	99.0	3.99	2.18	6.00	0.1919	0.1518
Inf - 0.60	22115	22201	99.6	6.03	7.87	21.68	0.0449	0.0365

A resolution cut off (SHEL 999 0.66) was applied to exclude poorly determined reflections at high diffraction angles. Several disordered parts can be found. One phenyl ring has a twofold positional disorder and was described with fixed occupancies of 70:30%. Isotropic atomic displacement parameters were used for the minor parts of the disorder.

Table S 9. Crystal data and structure refinement of complex **8**.

Identification code	13941	
Empirical formula	$C_{54} H_{45} Mo N O_3 Si_3$	
Color	light yellow	
Formula weight	936.12 g·mol^{-1}	
Temperature	100(2) K	
Wavelength	0.71073 Å	
Crystal system	Triclinic	
Space group	*P*-1, (no. 2)	
Unit cell dimensions	a = 9.8873(6) Å	α= 71.713(2)°.
	b = 13.6273(8) Å	β= 81.406(2)°.
	c = 18.3370(11) Å	γ = 78.354(2)°.
Volume	2287.7(2) Å^3	
Z	2	
Density (calculated)	1.359 Mg·m^{-3}	
Absorption coefficient	0.411 mm^{-1}	
F(000)	968 e	
Crystal size	0.094 x 0.080 x 0.041 mm^3	
θ range for data collection	1.175 to 32.576°.	
Index ranges	-14 ≤ h ≤ 14, -20≤ k ≤ 20, -27 ≤ l ≤ 27	
Reflections collected	112568	
Independent reflections	16644 [R_{int} = 0.0406]	
Reflections with I>2σ(I)	14361	
Completeness to θ = 25.242°	100.0 %	
Absorption correction	Gaussian	
Max. and min. transmission	0.98858 and 0.97152	
Refinement method	Full-matrix least-squares on F^2	
Data / restraints / parameters	16644 / 0 / 571	
Goodness-of-fit on F^2	1.074	
Final R indices [I>2σ(I)]	R_1 = 0.0442	wR^2 = 0.1033
R indices (all data)	R_1 = 0.0539	wR^2 = 0.1078
Extinction coefficient	n/a	
Largest diff. peak and hole	1.858 and -1.689 e·Å^{-3}	

Table S 10. Bond lengths [Å] and angles [°] of complex **8**.

Mo(1)-O(1)	1.8494(14)	Mo(1)-O(2)	1.8676(14)
Mo(1)-O(3)	1.8670(13)	Mo(1)-N(1)	1.6530(16)
Si(1)-O(1)	1.6658(15)	Si(1)-C(1)	1.8642(18)
Si(1)-C(7)	1.8569(19)	Si(1)-C(13)	1.8648(19)
Si(2)-O(2)	1.6430(14)	Si(2)-C(19)	1.8573(17)
Si(2)-C(25)	1.8555(19)	Si(2)-C(31)	1.8673(18)
Si(3)-O(3)	1.6521(14)	Si(3)-C(37)	1.8629(17)
Si(3)-C(43)	1.8590(18)	Si(3)-C(49A)	1.846(4)
Si(3)-C(49B)	1.905(5)	C(1)-C(2)	1.400(2)
C(1)-C(6)	1.398(2)	C(2)-H(2)	0.9500
C(2)-C(3)	1.388(3)	C(3)-H(3)	0.9500
C(3)-C(4)	1.392(3)	C(4)-H(4)	0.9500
C(4)-C(5)	1.379(3)	C(5)-H(5)	0.9500
C(5)-C(6)	1.391(3)	C(6)-H(6)	0.9500
C(7)-C(8)	1.392(3)	C(7)-C(12)	1.402(3)
C(8)-H(8)	0.9500	C(8)-C(9)	1.389(3)
C(9)-H(9)	0.9500	C(9)-C(10)	1.372(4)
C(10)-H(10)	0.9500	C(10)-C(11)	1.382(3)
C(11)-H(11)	0.9500	C(11)-C(12)	1.393(3)
C(12)-H(12)	0.9500	C(13)-C(14)	1.388(3)
C(13)-C(18)	1.398(3)	C(14)-H(14)	0.9500
C(14)-C(15)	1.391(3)	C(15)-H(15)	0.9500
C(15)-C(16)	1.381(3)	C(16)-H(16)	0.9500
C(16)-C(17)	1.379(3)	C(17)-H(17)	0.9500
C(17)-C(18)	1.392(3)	C(18)-H(18)	0.9500
C(19)-C(20)	1.399(2)	C(19)-C(24)	1.400(2)
C(20)-H(20)	0.9500	C(20)-C(21)	1.389(2)
C(21)-H(21)	0.9500	C(21)-C(22)	1.390(3)
C(22)-H(22)	0.9500	C(22)-C(23)	1.386(3)
C(23)-H(23)	0.9500	C(23)-C(24)	1.387(3)
C(24)-H(24)	0.9500	C(25)-C(26)	1.400(2)
C(25)-C(30)	1.401(2)	C(26)-H(26)	0.9500
C(26)-C(27)	1.385(3)	C(27)-H(27)	0.9500
C(27)-C(28)	1.388(3)	C(28)-H(28)	0.9500

C(28)-C(29)	1.385(3)	C(29)-H(29)	0.9500
C(29)-C(30)	1.389(3)	C(30)-H(30)	0.9500
C(31)-C(32)	1.390(3)	C(31)-C(36)	1.403(3)
C(32)-H(32)	0.9500	C(32)-C(33)	1.394(3)
C(33)-H(33)	0.9500	C(33)-C(34)	1.377(3)
C(34)-H(34)	0.9500	C(34)-C(35)	1.389(3)
C(35)-H(35)	0.9500	C(35)-C(36)	1.389(3)
C(36)-H(36)	0.9500	C(37)-C(38)	1.397(2)
C(37)-C(42)	1.404(2)	C(38)-H(38)	0.9500
C(38)-C(39)	1.392(2)	C(39)-H(39)	0.9500
C(39)-C(40)	1.382(3)	C(40)-H(40)	0.9500
C(40)-C(41)	1.386(3)	C(41)-H(41)	0.9500
C(41)-C(42)	1.390(3)	C(42)-H(42)	0.9500
C(43)-C(44)	1.393(3)	C(43)-C(48)	1.398(3)
C(44)-H(44)	0.9500	C(44)-C(45)	1.391(3)
C(45)-H(45)	0.9500	C(45)-C(46)	1.372(4)
C(46)-H(46)	0.9500	C(46)-C(47)	1.375(4)
C(47)-H(47)	0.9500	C(47)-C(48)	1.391(3)
C(48)-H(48)	0.9500	C(49A)-C(50A)	1.395(5)
C(49A)-C(54A)	1.394(7)	C(50A)-H(50A)	0.9500
C(50A)-C(51A)	1.388(5)	C(50B)-H(50B)	0.9500
C(50B)-C(49B)	1.3900	C(50B)-C(51B)	1.3900
C(49B)-C(54B)	1.3900	C(54B)-H(54B)	0.9500
C(54B)-C(53B)	1.3900	C(53B)-H(53B)	0.9500
C(53B)-C(52B)	1.3900	C(52B)-H(52B)	0.9500
C(52B)-C(51B)	1.3900	C(51B)-H(51B)	0.9500
C(51A)-H(51A)	0.9500	C(51A)-C(52A)	1.354(7)
C(52A)-H(52A)	0.9500	C(52A)-C(53A)	1.389(8)
C(53A)-H(53A)	0.9500	C(53A)-C(54A)	1.390(5)
C(54A)-H(54A)	0.9500		
O(1)-Mo(1)-O(2)	112.48(6)	O(1)-Mo(1)-O(3)	113.06(6)
O(3)-Mo(1)-O(2)	109.14(6)	N(1)-Mo(1)-O(1)	108.76(8)
N(1)-Mo(1)-O(2)	105.09(8)	N(1)-Mo(1)-O(3)	107.91(7)
O(1)-Si(1)-C(1)	106.41(8)	O(1)-Si(1)-C(7)	109.49(8)
O(1)-Si(1)-C(13)	108.52(8)	C(1)-Si(1)-C(13)	110.01(8)

C(7)-Si(1)-C(1)	109.43(8)	C(7)-Si(1)-C(13)	112.78(8)
O(2)-Si(2)-C(19)	106.60(8)	O(2)-Si(2)-C(25)	108.14(8)
O(2)-Si(2)-C(31)	109.47(8)	C(19)-Si(2)-C(31)	112.63(8)
C(25)-Si(2)-C(19)	111.73(8)	C(25)-Si(2)-C(31)	108.16(8)
O(3)-Si(3)-C(37)	105.75(8)	O(3)-Si(3)-C(43)	110.68(8)
O(3)-Si(3)-C(49A)	107.77(18)	O(3)-Si(3)-C(49B)	111.4(2)
C(37)-Si(3)-C(49B)	106.7(3)	C(43)-Si(3)-C(37)	109.85(8)
C(43)-Si(3)-C(49B)	112.2(3)	C(49A)-Si(3)-C(37)	111.4(2)
C(49A)-Si(3)-C(43)	111.26(15)	Si(1)-O(1)-Mo(1)	154.64(9)
Si(2)-O(2)-Mo(1)	153.62(10)	Si(3)-O(3)-Mo(1)	148.06(9)
C(2)-C(1)-Si(1)	120.96(13)	C(6)-C(1)-Si(1)	121.49(13)
C(6)-C(1)-C(2)	117.55(16)	C(1)-C(2)-H(2)	119.4
C(3)-C(2)-C(1)	121.19(17)	C(3)-C(2)-H(2)	119.4
C(2)-C(3)-H(3)	120.0	C(2)-C(3)-C(4)	120.09(17)
C(4)-C(3)-H(3)	120.0	C(3)-C(4)-H(4)	120.2
C(5)-C(4)-C(3)	119.65(17)	C(5)-C(4)-H(4)	120.2
C(4)-C(5)-H(5)	119.9	C(4)-C(5)-C(6)	120.16(18)
C(6)-C(5)-H(5)	119.9	C(1)-C(6)-H(6)	119.3
C(5)-C(6)-C(1)	121.34(17)	C(5)-C(6)-H(6)	119.3
C(8)-C(7)-Si(1)	120.00(15)	C(8)-C(7)-C(12)	117.32(18)
C(12)-C(7)-Si(1)	122.17(15)	C(7)-C(8)-H(8)	119.2
C(9)-C(8)-C(7)	121.6(2)	C(9)-C(8)-H(8)	119.2
C(8)-C(9)-H(9)	119.9	C(10)-C(9)-C(8)	120.1(2)
C(10)-C(9)-H(9)	119.9	C(9)-C(10)-H(10)	120.0
C(9)-C(10)-C(11)	119.9(2)	C(11)-C(10)-H(10)	120.0
C(10)-C(11)-H(11)	119.9	C(10)-C(11)-C(12)	120.1(2)
C(12)-C(11)-H(11)	119.9	C(7)-C(12)-H(12)	119.5
C(11)-C(12)-C(7)	120.9(2)	C(11)-C(12)-H(12)	119.5
C(14)-C(13)-Si(1)	122.24(14)	C(14)-C(13)-C(18)	117.81(18)
C(18)-C(13)-Si(1)	119.93(15)	C(13)-C(14)-H(14)	119.4
C(13)-C(14)-C(15)	121.2(2)	C(15)-C(14)-H(14)	119.4
C(14)-C(15)-H(15)	119.9	C(16)-C(15)-C(14)	120.2(2)
C(16)-C(15)-H(15)	119.9	C(15)-C(16)-H(16)	120.2
C(17)-C(16)-C(15)	119.7(2)	C(17)-C(16)-H(16)	120.2
C(16)-C(17)-H(17)	119.9	C(16)-C(17)-C(18)	120.1(2)
C(18)-C(17)-H(17)	119.9	C(13)-C(18)-H(18)	119.5

C(17)-C(18)-C(13)	121.0(2)	C(17)-C(18)-H(18)	119.5
C(20)-C(19)-Si(2)	122.73(13)	C(20)-C(19)-C(24)	117.97(16)
C(24)-C(19)-Si(2)	119.29(13)	C(19)-C(20)-H(20)	119.5
C(21)-C(20)-C(19)	120.99(16)	C(21)-C(20)-H(20)	119.5
C(20)-C(21)-H(21)	120.0	C(20)-C(21)-C(22)	120.08(17)
C(22)-C(21)-H(21)	120.0	C(21)-C(22)-H(22)	120.1
C(23)-C(22)-C(21)	119.78(17)	C(23)-C(22)-H(22)	120.1
C(22)-C(23)-H(23)	120.0	C(22)-C(23)-C(24)	120.02(17)
C(24)-C(23)-H(23)	120.0	C(19)-C(24)-H(24)	119.4
C(23)-C(24)-C(19)	121.16(17)	C(23)-C(24)-H(24)	119.4
C(26)-C(25)-Si(2)	119.41(14)	C(26)-C(25)-C(30)	118.09(17)
C(30)-C(25)-Si(2)	122.48(13)	C(25)-C(26)-H(26)	119.5
C(27)-C(26)-C(25)	120.99(18)	C(27)-C(26)-H(26)	119.5
C(26)-C(27)-H(27)	120.0	C(26)-C(27)-C(28)	119.92(18)
C(28)-C(27)-H(27)	120.0	C(27)-C(28)-H(28)	119.9
C(29)-C(28)-C(27)	120.22(18)	C(29)-C(28)-H(28)	119.9
C(28)-C(29)-H(29)	120.1	C(28)-C(29)-C(30)	119.77(18)
C(30)-C(29)-H(29)	120.1	C(25)-C(30)-H(30)	119.5
C(29)-C(30)-C(25)	121.02(16)	C(29)-C(30)-H(30)	119.5
C(32)-C(31)-Si(2)	122.88(15)	C(32)-C(31)-C(36)	117.27(18)
C(36)-C(31)-Si(2)	119.85(14)	C(31)-C(32)-H(32)	119.2
C(31)-C(32)-C(33)	121.6(2)	C(33)-C(32)-H(32)	119.2
C(32)-C(33)-H(33)	120.0	C(34)-C(33)-C(32)	119.9(2)
C(34)-C(33)-H(33)	120.0	C(33)-C(34)-H(34)	120.0
C(33)-C(34)-C(35)	120.0(2)	C(35)-C(34)-H(34)	120.0
C(34)-C(35)-H(35)	120.2	C(34)-C(35)-C(36)	119.7(2)
C(36)-C(35)-H(35)	120.2	C(31)-C(36)-H(36)	119.2
C(35)-C(36)-C(31)	121.51(19)	C(35)-C(36)-H(36)	119.2
C(38)-C(37)-Si(3)	122.52(13)	C(38)-C(37)-C(42)	117.53(16)
C(42)-C(37)-Si(3)	119.96(13)	C(37)-C(38)-H(38)	119.3
C(39)-C(38)-C(37)	121.46(16)	C(39)-C(38)-H(38)	119.3
C(38)-C(39)-H(39)	120.1	C(40)-C(39)-C(38)	119.85(17)
C(40)-C(39)-H(39)	120.1	C(39)-C(40)-H(40)	120.0
C(39)-C(40)-C(41)	120.03(17)	C(41)-C(40)-H(40)	120.0
C(40)-C(41)-H(41)	120.0	C(40)-C(41)-C(42)	120.01(17)
C(42)-C(41)-H(41)	120.0	C(37)-C(42)-H(42)	119.4

C(41)-C(42)-C(37)	121.11(17)	C(41)-C(42)-H(42)	119.4
C(44)-C(43)-Si(3)	121.99(15)	C(44)-C(43)-C(48)	117.70(18)
C(48)-C(43)-Si(3)	120.15(15)	C(43)-C(44)-H(44)	119.3
C(45)-C(44)-C(43)	121.3(2)	C(45)-C(44)-H(44)	119.3
C(44)-C(45)-H(45)	120.1	C(46)-C(45)-C(44)	119.8(2)
C(46)-C(45)-H(45)	120.1	C(45)-C(46)-H(46)	119.9
C(45)-C(46)-C(47)	120.2(2)	C(47)-C(46)-H(46)	119.9
C(46)-C(47)-H(47)	119.9	C(46)-C(47)-C(48)	120.2(2)
C(48)-C(47)-H(47)	119.9	C(43)-C(48)-H(48)	119.7
C(47)-C(48)-C(43)	120.7(2)	C(47)-C(48)-H(48)	119.7
C(50A)-C(49A)-Si(3)	122.5(4)	C(54A)-C(49A)-Si(3)	121.1(4)
C(54A)-C(49A)-C(50A)	116.4(3)	C(49A)-C(50A)-H(50A)	119.2
C(51A)-C(50A)-C(49A)	121.7(4)	C(51A)-C(50A)-H(50A)	119.2
C(49B)-C(50B)-H(50B)	120.0	C(49B)-C(50B)-C(51B)	120.0
C(51B)-C(50B)-H(50B)	120.0	C(50B)-C(49B)-Si(3)	123.7(4)
C(50B)-C(49B)-C(54B)	120.0	C(54B)-C(49B)-Si(3)	116.3(4)
C(49B)-C(54B)-H(54B)	120.0	C(53B)-C(54B)-C(49B)	120.0
C(53B)-C(54B)-H(54B)	120.0	C(54B)-C(53B)-H(53B)	120.0
C(52B)-C(53B)-C(54B)	120.0	C(52B)-C(53B)-H(53B)	120.0
C(53B)-C(52B)-H(52B)	120.0	C(53B)-C(52B)-C(51B)	120.0
C(51B)-C(52B)-H(52B)	120.0	C(50B)-C(51B)-H(51B)	120.0
C(52B)-C(51B)-C(50B)	120.0	C(52B)-C(51B)-H(51B)	120.0
C(50A)-C(51A)-H(51A)	120.0	C(52A)-C(51A)-C(50A)	120.0(4)
C(52A)-C(51A)-H(51A)	120.0	C(51A)-C(52A)-H(52A)	119.5
C(51A)-C(52A)-C(53A)	120.9(3)	C(53A)-C(52A)-H(52A)	119.5
C(52A)-C(53A)-H(53A)	120.8	C(52A)-C(53A)-C(54A)	118.3(4)
C(54A)-C(53A)-H(53A)	120.8	C(49A)-C(54A)-H(54A)	118.8
C(53A)-C(54A)-C(49A)	122.5(5)	C(53A)-C(54A)-H(54A)	118.8

Single crystal structure analysis of complex 13a · benzene/pentane solvate

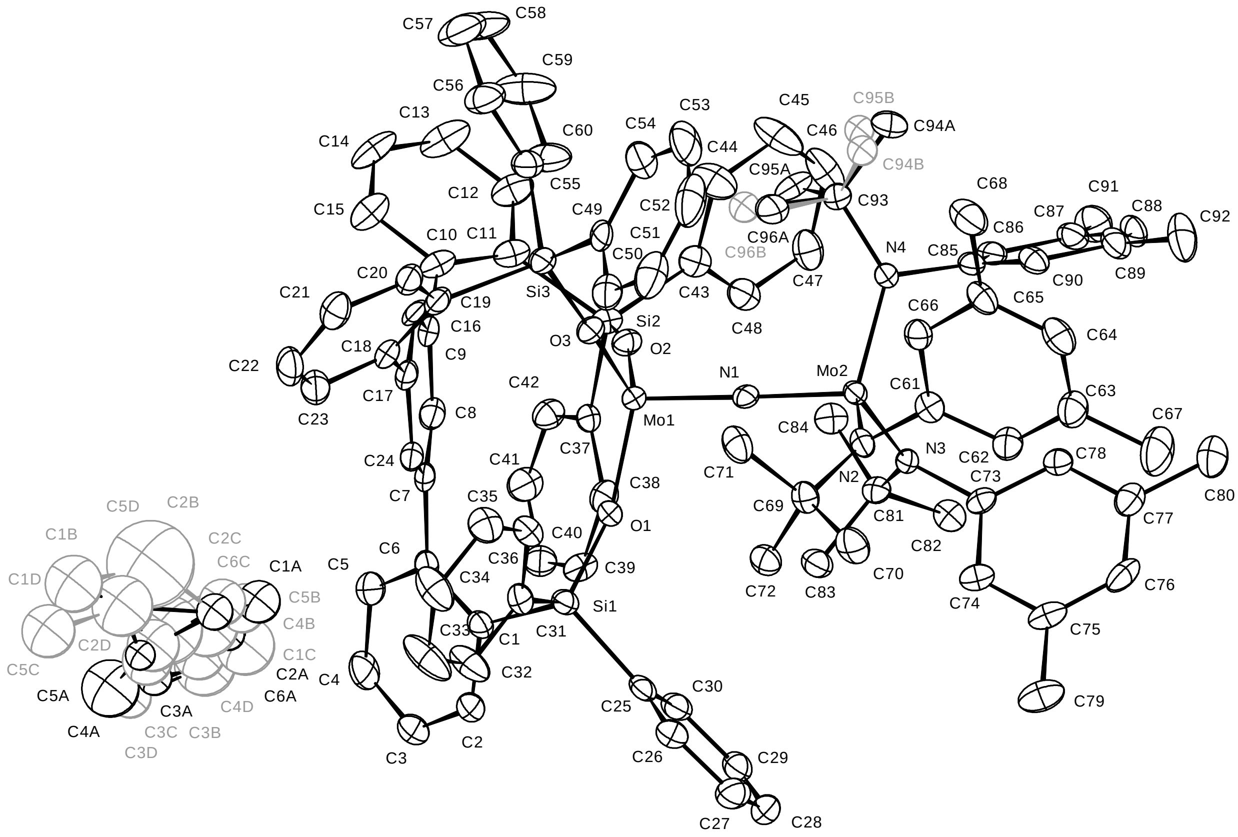

Figure S 12. The molecular structure of complex **13a · benzene/pentane solvate**. H atoms have been removed for clarity. Main structure shown in black and disordered parts shown in grey.

X-ray Crystal Structure Analysis of Complex 13a · benzene/pentane solvate:

C_{203} H_{216} Mo_4 N_8 O_6 Si_6, M_r = 3416.13 g mol^{-1}, orange plate, crystal size 0.061 x 0.061 x 0.009 mm^3, Monoclinic, space group $P2_1/c$ [14], a = 14.885(3) Å, b = 28.731(7) Å, c = 20.650(5) Å, β = 91.244(12)°, V = 8829(4) Å^3, T = 100(2) K, Z = 2, D_{calc} = 1.285 g·cm^3, λ = 0.71073 Å, μ(Mo-K_α) = 0.378 mm^{-1}, Gaussian absorption correction ($T_{\min}$ = 0.97863, $T_{\max}$ = 0.99810), Bruker-AXS Kappa Mach3 with APEX-II detector and IμS microfocus Mo-anode X-ray source, 1.215 < θ < 26.372°, 161323 measured reflections, 17891 independent reflections, 10974 reflections with $I > 2\sigma(I)$, R_{int} = 0.1752. The structure was solved by *SHELXT* and refined by full-matrix least-squares (*SHELXL*) against F^2 to R_1 = 0.0506 [$I > 2\sigma(I)$], wR_2 = 0.1018 [all data], 1071 parameters and 40 restraints.

Full .cif data for the compound are available under the CCDC number **CCDC-2265452**

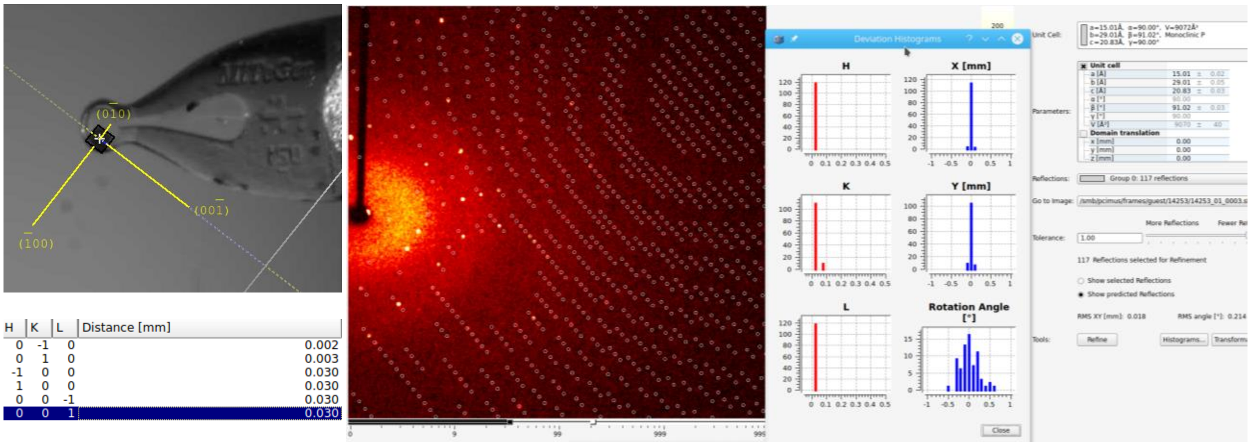

Figure S 13. Crystal faces and unit cell determination/refinement of complex **13a · benzene/pentane solvate**.

INTENSITY STATISTICS FOR DATASET

Resolution	#Data	#Theory	%Complete	Redundancy	Mean I	Mean I/s	Rmerge	Rsigma
Inf - 3.31	276	282	97.9	13.06	81.84	42.40	0.0394	0.0154
3.31 - 2.19	641	652	98.3	13.86	40.48	32.32	0.0606	0.0206
2.19 - 1.73	922	944	97.7	14.16	27.73	25.54	0.0848	0.0276
1.73 - 1.50	952	982	96.9	14.09	21.81	21.95	0.1039	0.0330
1.50 - 1.36	921	942	97.8	14.08	19.32	19.56	0.1219	0.0386
1.36 - 1.27	854	878	97.3	14.00	14.91	15.73	0.1546	0.0492
1.27 - 1.19	980	994	98.6	13.60	11.37	12.03	0.2002	0.0664
1.19 - 1.13	932	944	98.7	13.31	12.52	11.93	0.2001	0.0654
1.13 - 1.08	930	940	98.9	11.35	11.18	9.84	0.2288	0.0830
1.08 - 1.04	883	894	98.8	8.93	9.51	7.32	0.2680	0.1133
1.04 - 1.00	1039	1046	99.3	7.66	7.31	5.56	0.3184	0.1568
1.00 - 0.97	906	915	99.0	6.75	6.65	4.70	0.3463	0.1862
0.97 - 0.94	987	994	99.3	6.08	6.40	4.34	0.3607	0.2028
0.94 - 0.92	759	759	100.0	5.45	5.36	3.55	0.4006	0.2539
0.92 - 0.90	812	820	99.0	5.00	6.15	3.72	0.3555	0.2369
0.90 - 0.88	899	904	99.4	4.74	5.70	3.45	0.3751	0.2630
0.88 - 0.86	988	988	100.0	4.61	5.06	2.97	0.4119	0.3046
0.86 - 0.84	1076	1080	99.6	4.48	5.19	2.97	0.4056	0.3095
0.84 - 0.82	1191	1192	99.9	4.39	4.53	2.57	0.4605	0.3688
0.82 - 0.81	641	641	100.0	4.26	3.89	2.12	0.5142	0.4421
0.81 - 0.80	664	665	99.8	4.25	3.96	2.12	0.5034	0.4365
0.90 - 0.80	5459	5470	99.8	4.47	4.80	2.76	0.4319	0.3370
Inf - 0.80	18253	18456	98.9	8.84	12.18	9.97	0.1501	0.0970

A terminal ^{t}Bu group was found to be rotationally disordered over two positions. The atomic occupancy was fixed to be 70:30%. The two solute molecules (benzene and pentane) show positional and substitutional disorder. The occupancies of the solute molecules have been described using fixed occupancies of 25% for each of the four components. Isotropic atomic displacement parameters were used for the minor components and the solute molecules. Several restraints were used to model the disordered solute molecules.

Table S 11. Crystal data and structure refinement of complex **13a · benzene/pentane solvate**.

Identification code	14253	
Empirical formula	$C_{203} H_{216} Mo_4 N_8 O_6 Si_6$	
Color	orange	
Formula weight	3416.13 g·mol^{-1}	
Temperature	100(2) K	
Wavelength	0.71073 Å	
Crystal system	Monoclinic	
Space group	$P2_1/c$, (no. 14)	
Unit cell dimensions	a = 14.885(3) Å	α= 90°.
	b = 28.731(7) Å	β= 91.244(12)°.
	c = 20.650(5) Å	γ = 90°.
Volume	8829(4) Å^3	
Z	2	
Density (calculated)	1.285 Mg·m^{-3}	
Absorption coefficient	0.378 mm^{-1}	
F(000)	3580 e	
Crystal size	0.061 x 0.061 x 0.009 mm^3	
θ range for data collection	1.215 to 26.372°.	
Index ranges	-18 ≤ h ≤ 18, -35≤ k ≤ 35, -25 ≤ l ≤ 25	
Reflections collected	161323	
Independent reflections	17891 [R_{int} = 0.1752]	
Reflections with I>2σ(I)	10974	
Completeness to θ = 25.242°	99.0 %	
Absorption correction	Gaussian	
Max. and min. transmission	0.99810 and 0.97863	
Refinement method	Full-matrix least-squares on F^2	
Data / restraints / parameters	17891 / 40 / 1071	
Goodness-of-fit on F^2	1.005	
Final R indices [I>2σ(I)]	R_1 = 0.0506	wR^2 = 0.0849
R indices (all data)	R_1 = 0.1118	wR^2 = 0.1018
Extinction coefficient	n/a	
Largest diff. peak and hole	0.605 and -0.822 e·Å^{-3}	

Table S 12. Bond lengths [Å] and angles [°] of complex **13a · benzene/pentane solvate**.

Mo(1)-O(1)	1.910(2)	Mo(1)-O(2)	1.912(2)
Mo(1)-O(3)	1.920(2)	Mo(1)-N(1)	1.838(3)
Mo(2)-N(1)	1.808(3)	Mo(2)-N(2)	1.970(3)
Mo(2)-N(3)	1.966(3)	Mo(2)-N(4)	1.968(3)
Si(1)-O(1)	1.635(2)	Si(1)-C(1)	1.888(4)
Si(1)-C(25)	1.886(4)	Si(1)-C(31)	1.873(4)
Si(2)-O(2)	1.631(3)	Si(2)-C(11)	1.892(4)
Si(2)-C(37)	1.873(4)	Si(2)-C(43)	1.882(4)
Si(3)-O(3)	1.624(2)	Si(3)-C(19)	1.894(4)
Si(3)-C(49)	1.880(4)	Si(3)-C(55)	1.873(4)
N(2)-C(61)	1.451(4)	N(2)-C(69)	1.504(4)
N(3)-C(73)	1.448(4)	N(3)-C(81)	1.504(4)
N(4)-C(85)	1.443(4)	N(4)-C(93)	1.509(4)
C(1)-C(2)	1.413(5)	C(1)-C(6)	1.415(5)
C(2)-H(2)	0.9500	C(2)-C(3)	1.388(5)
C(3)-H(3)	0.9500	C(3)-C(4)	1.373(5)
C(4)-H(4)	0.9500	C(4)-C(5)	1.387(5)
C(5)-H(5)	0.9500	C(5)-C(6)	1.400(5)
C(6)-C(7)	1.494(5)	C(7)-C(8)	1.392(5)
C(7)-C(24)	1.392(5)	C(8)-H(8)	0.9500
C(8)-C(9)	1.390(5)	C(9)-C(10)	1.502(5)
C(9)-C(16)	1.391(5)	C(10)-C(11)	1.415(5)
C(10)-C(15)	1.403(5)	C(11)-C(12)	1.400(5)
C(12)-H(12)	0.9500	C(12)-C(13)	1.387(5)
C(13)-H(13)	0.9500	C(13)-C(14)	1.376(6)
C(14)-H(14)	0.9500	C(14)-C(15)	1.386(6)
C(15)-H(15)	0.9500	C(16)-H(16)	0.9500
C(16)-C(17)	1.395(5)	C(17)-C(18)	1.496(5)
C(17)-C(24)	1.394(5)	C(18)-C(19)	1.415(5)
C(18)-C(23)	1.394(5)	C(19)-C(20)	1.408(5)
C(20)-H(20)	0.9500	C(20)-C(21)	1.394(5)
C(21)-H(21)	0.9500	C(21)-C(22)	1.381(5)
C(22)-H(22)	0.9500	C(22)-C(23)	1.387(5)
C(23)-H(23)	0.9500	C(24)-H(24)	0.9500

C(25)-C(26)	1.395(5)	C(25)-C(30)	1.403(5)
C(26)-H(26)	0.9500	C(26)-C(27)	1.386(5)
C(27)-H(27)	0.9500	C(27)-C(28)	1.371(6)
C(28)-H(28)	0.9500	C(28)-C(29)	1.380(5)
C(29)-H(29)	0.9500	C(29)-C(30)	1.386(5)
C(30)-H(30)	0.9500	C(31)-C(32)	1.396(5)
C(31)-C(36)	1.388(5)	C(32)-H(32)	0.9500
C(32)-C(33)	1.396(6)	C(33)-H(33)	0.9500
C(33)-C(34)	1.371(6)	C(34)-H(34)	0.9500
C(34)-C(35)	1.374(5)	C(35)-H(35)	0.9500
C(35)-C(36)	1.381(5)	C(36)-H(36)	0.9500
C(37)-C(38)	1.393(5)	C(37)-C(42)	1.402(5)
C(38)-H(38)	0.9500	C(38)-C(39)	1.390(5)
C(39)-H(39)	0.9500	C(39)-C(40)	1.380(5)
C(40)-H(40)	0.9500	C(40)-C(41)	1.378(5)
C(41)-H(41)	0.9500	C(41)-C(42)	1.381(5)
C(42)-H(42)	0.9500	C(43)-C(44)	1.393(5)
C(43)-C(48)	1.398(5)	C(44)-H(44)	0.9500
C(44)-C(45)	1.385(6)	C(45)-H(45)	0.9500
C(45)-C(46)	1.379(6)	C(46)-H(46)	0.9500
C(46)-C(47)	1.377(6)	C(47)-H(47)	0.9500
C(47)-C(48)	1.382(5)	C(48)-H(48)	0.9500
C(49)-C(50)	1.397(5)	C(49)-C(54)	1.399(5)
C(50)-H(50)	0.9500	C(50)-C(51)	1.387(5)
C(51)-H(51)	0.9500	C(51)-C(52)	1.371(6)
C(52)-H(52)	0.9500	C(52)-C(53)	1.366(7)
C(53)-H(53)	0.9500	C(53)-C(54)	1.390(6)
C(54)-H(54)	0.9500	C(55)-C(56)	1.384(5)
C(55)-C(60)	1.400(6)	C(56)-H(56)	0.9500
C(56)-C(57)	1.380(6)	C(57)-H(57)	0.9500
C(57)-C(58)	1.385(7)	C(58)-H(58)	0.9500
C(58)-C(59)	1.366(6)	C(59)-H(59)	0.9500
C(59)-C(60)	1.394(6)	C(60)-H(60)	0.9500
C(61)-C(62)	1.394(5)	C(61)-C(66)	1.387(5)
C(62)-H(62)	0.9500	C(62)-C(63)	1.400(5)
C(63)-C(64)	1.395(5)	C(63)-C(67)	1.522(5)

C(64)-H(64)	0.9500	C(64)-C(65)	1.383(5)
C(65)-C(66)	1.392(5)	C(65)-C(68)	1.500(5)
C(66)-H(66)	0.9500	C(67)-H(67A)	0.9800
C(67)-H(67B)	0.9800	C(67)-H(67C)	0.9800
C(68)-H(68A)	0.9800	C(68)-H(68B)	0.9800
C(68)-H(68C)	0.9800	C(69)-C(70)	1.531(5)
C(69)-C(71)	1.537(5)	C(69)-C(72)	1.530(5)
C(70)-H(70A)	0.9800	C(70)-H(70B)	0.9800
C(70)-H(70C)	0.9800	C(71)-H(71A)	0.9800
C(71)-H(71B)	0.9800	C(71)-H(71C)	0.9800
C(72)-H(72A)	0.9800	C(72)-H(72B)	0.9800
C(72)-H(72C)	0.9800	C(73)-C(74)	1.395(5)
C(73)-C(78)	1.395(5)	C(74)-H(74)	0.9500
C(74)-C(75)	1.395(5)	C(75)-C(76)	1.390(5)
C(75)-C(79)	1.502(5)	C(76)-H(76)	0.9500
C(76)-C(77)	1.386(5)	C(77)-C(78)	1.392(5)
C(77)-C(80)	1.511(5)	C(78)-H(78)	0.9500
C(79)-H(79A)	0.9800	C(79)-H(79B)	0.9800
C(79)-H(79C)	0.9800	C(80)-H(80A)	0.9800
C(80)-H(80B)	0.9800	C(80)-H(80C)	0.9800
C(81)-C(82)	1.532(5)	C(81)-C(83)	1.533(5)
C(81)-C(84)	1.530(5)	C(82)-H(82A)	0.9800
C(82)-H(82B)	0.9800	C(82)-H(82C)	0.9800
C(83)-H(83A)	0.9800	C(83)-H(83B)	0.9800
C(83)-H(83C)	0.9800	C(84)-H(84A)	0.9800
C(84)-H(84B)	0.9800	C(84)-H(84C)	0.9800
C(85)-C(86)	1.397(5)	C(85)-C(90)	1.388(5)
C(86)-H(86)	0.9500	C(86)-C(87)	1.389(5)
C(87)-C(88)	1.394(5)	C(87)-C(91)	1.503(5)
C(88)-H(88)	0.9500	C(88)-C(89)	1.390(5)
C(89)-C(90)	1.399(5)	C(89)-C(92)	1.512(5)
C(90)-H(90)	0.9500	C(91)-H(91A)	0.9800
C(91)-H(91B)	0.9800	C(91)-H(91C)	0.9800
C(92)-H(92A)	0.9800	C(92)-H(92B)	0.9800
C(92)-H(92C)	0.9800	C(93)-C(94A)	1.490(6)
C(93)-C(94B)	1.560(12)	C(93)-C(95A)	1.488(7)

C(93)-C(95B)	1.655(12)	C(93)-C(96A)	1.549(7)
C(93)-C(96B)	1.510(12)	C(94A)-H(94A)	0.9800
C(94A)-H(94B)	0.9800	C(94A)-H(94C)	0.9800
C(94B)-H(94D)	0.9800	C(94B)-H(94E)	0.9800
C(94B)-H(94F)	0.9800	C(95A)-H(95A)	0.9800
C(95A)-H(95B)	0.9800	C(95A)-H(95C)	0.9800
C(95B)-H(95D)	0.9800	C(95B)-H(95E)	0.9800
C(95B)-H(95F)	0.9800	C(96A)-H(96A)	0.9800
C(96A)-H(96B)	0.9800	C(96A)-H(96C)	0.9800
C(96B)-H(96D)	0.9800	C(96B)-H(96E)	0.9800
C(96B)-H(96F)	0.9800	C(1B)-H	0.9800
C(1B)-HA	0.9800	C(1B)-HB	0.9800
C(1B)-C(2B)	1.492(13)	C(1D)-HC	0.9800
C(1D)-HD	0.9800	C(1D)-HE	0.9800
C(1D)-C(2D)	1.514(13)	C(2B)-HF	0.9900
C(2B)-HG	0.9900	C(2B)-C(3B)	1.492(14)
C(2D)-HH	0.9900	C(2D)-HI	0.9900
C(2D)-C(3D)	1.510(13)	C(3B)-HJ	0.9900
C(3B)-HK	0.9900	C(3B)-C(4B)	1.477(13)
C(3D)-HL	0.9900	C(3D)-HM	0.9900
C(3D)-C(4D)	1.510(13)	C(4B)-HN	0.9900
C(4B)-HO	0.9900	C(4B)-C(5B)	1.485(13)
C(4D)-HP	0.9900	C(4D)-HQ	0.9900
C(4D)-C(5D)	1.527(13)	C(5B)-HR	0.9800
C(5B)-HS	0.9800	C(5B)-HT	0.9800
C(5D)-HU	0.9800	C(5D)-HV	0.9800
C(5D)-HW	0.9800	C(6A)-HX	0.9500
C(6A)-C(5A)	1.3900	C(6A)-C(1A)	1.3900
C(5A)-HY	0.9500	C(5A)-C(4A)	1.3900
C(4A)-HZ	0.9500	C(4A)-C(3A)	1.3900
C(3A)-H(1)	0.9500	C(3A)-C(2A)	1.3900
C(2A)-H(6)	0.9500	C(2A)-C(1A)	1.3900
C(1A)-H(7)	0.9500	C(6C)-H(9)	0.9500
C(6C)-C(5C)	1.3900	C(6C)-C(1C)	1.3900
C(5C)-H(10)	0.9500	C(5C)-C(4C)	1.3900
C(4C)-H(11)	0.9500	C(4C)-C(3C)	1.3900

C(3C)-H(17)	0.9500	C(3C)-C(2C)	1.3900
C(2C)-H(18)	0.9500	C(2C)-C(1C)	1.3900
C(1C)-H(19)	0.9500		

O(1)-Mo(1)-O(2)	115.37(10)	O(1)-Mo(1)-O(3)	120.05(10)
O(2)-Mo(1)-O(3)	110.55(10)	N(1)-Mo(1)-O(1)	102.73(11)
N(1)-Mo(1)-O(2)	102.93(11)	N(1)-Mo(1)-O(3)	102.30(11)
N(1)-Mo(2)-N(2)	103.35(12)	N(1)-Mo(2)-N(3)	104.58(12)
N(1)-Mo(2)-N(4)	105.53(12)	N(3)-Mo(2)-N(2)	112.49(12)
N(3)-Mo(2)-N(4)	112.17(12)	N(4)-Mo(2)-N(2)	117.21(12)
O(1)-Si(1)-C(1)	111.44(14)	O(1)-Si(1)-C(25)	109.58(14)
O(1)-Si(1)-C(31)	108.79(15)	C(25)-Si(1)-C(1)	106.52(16)
C(31)-Si(1)-C(1)	109.61(16)	C(31)-Si(1)-C(25)	110.90(17)
O(2)-Si(2)-C(11)	111.88(15)	O(2)-Si(2)-C(37)	109.95(14)
O(2)-Si(2)-C(43)	110.01(15)	C(37)-Si(2)-C(11)	108.14(16)
C(37)-Si(2)-C(43)	110.17(16)	C(43)-Si(2)-C(11)	106.63(17)
O(3)-Si(3)-C(19)	110.55(15)	O(3)-Si(3)-C(49)	110.83(15)
O(3)-Si(3)-C(55)	110.41(16)	C(49)-Si(3)-C(19)	106.25(17)
C(55)-Si(3)-C(19)	109.32(16)	C(55)-Si(3)-C(49)	109.39(18)
Si(1)-O(1)-Mo(1)	160.80(15)	Si(2)-O(2)-Mo(1)	162.78(15)
Si(3)-O(3)-Mo(1)	165.55(16)	Mo(2)-N(1)-Mo(1)	178.56(18)
C(61)-N(2)-Mo(2)	110.9(2)	C(61)-N(2)-C(69)	114.5(3)
C(69)-N(2)-Mo(2)	133.5(2)	C(73)-N(3)-Mo(2)	111.2(2)
C(73)-N(3)-C(81)	116.1(3)	C(81)-N(3)-Mo(2)	131.8(2)
C(85)-N(4)-Mo(2)	113.3(2)	C(85)-N(4)-C(93)	113.9(3)
C(93)-N(4)-Mo(2)	132.5(2)	C(2)-C(1)-Si(1)	117.5(3)
C(2)-C(1)-C(6)	116.9(3)	C(6)-C(1)-Si(1)	125.6(3)
C(1)-C(2)-H(2)	119.0	C(3)-C(2)-C(1)	121.9(4)
C(3)-C(2)-H(2)	119.0	C(2)-C(3)-H(3)	119.9
C(4)-C(3)-C(2)	120.2(4)	C(4)-C(3)-H(3)	119.9
C(3)-C(4)-H(4)	120.2	C(3)-C(4)-C(5)	119.7(4)
C(5)-C(4)-H(4)	120.2	C(4)-C(5)-H(5)	119.5
C(4)-C(5)-C(6)	121.0(4)	C(6)-C(5)-H(5)	119.5
C(1)-C(6)-C(7)	122.5(3)	C(5)-C(6)-C(1)	120.2(3)
C(5)-C(6)-C(7)	117.3(3)	C(8)-C(7)-C(6)	120.2(3)
C(24)-C(7)-C(6)	121.1(3)	C(24)-C(7)-C(8)	118.6(3)

C(7)-C(8)-H(8)	119.4	C(9)-C(8)-C(7)	121.1(3)
C(9)-C(8)-H(8)	119.4	C(8)-C(9)-C(10)	120.0(3)
C(8)-C(9)-C(16)	119.0(3)	C(16)-C(9)-C(10)	121.0(3)
C(11)-C(10)-C(9)	121.9(3)	C(15)-C(10)-C(9)	118.3(4)
C(15)-C(10)-C(11)	119.8(4)	C(10)-C(11)-Si(2)	124.6(3)
C(12)-C(11)-Si(2)	118.4(3)	C(12)-C(11)-C(10)	117.0(3)
C(11)-C(12)-H(12)	118.6	C(13)-C(12)-C(11)	122.8(4)
C(13)-C(12)-H(12)	118.6	C(12)-C(13)-H(13)	120.4
C(14)-C(13)-C(12)	119.3(4)	C(14)-C(13)-H(13)	120.4
C(13)-C(14)-H(14)	119.9	C(13)-C(14)-C(15)	120.2(4)
C(15)-C(14)-H(14)	119.9	C(10)-C(15)-H(15)	119.6
C(14)-C(15)-C(10)	120.9(4)	C(14)-C(15)-H(15)	119.6
C(9)-C(16)-H(16)	119.3	C(9)-C(16)-C(17)	121.4(3)
C(17)-C(16)-H(16)	119.3	C(16)-C(17)-C(18)	120.8(3)
C(24)-C(17)-C(16)	118.1(3)	C(24)-C(17)-C(18)	120.9(3)
C(19)-C(18)-C(17)	122.2(3)	C(23)-C(18)-C(17)	118.2(3)
C(23)-C(18)-C(19)	119.7(3)	C(18)-C(19)-Si(3)	124.3(3)
C(20)-C(19)-Si(3)	118.5(3)	C(20)-C(19)-C(18)	117.2(3)
C(19)-C(20)-H(20)	118.8	C(21)-C(20)-C(19)	122.3(3)
C(21)-C(20)-H(20)	118.8	C(20)-C(21)-H(21)	120.4
C(22)-C(21)-C(20)	119.3(4)	C(22)-C(21)-H(21)	120.4
C(21)-C(22)-H(22)	120.1	C(21)-C(22)-C(23)	119.8(4)
C(23)-C(22)-H(22)	120.1	C(18)-C(23)-H(23)	119.2
C(22)-C(23)-C(18)	121.6(4)	C(22)-C(23)-H(23)	119.2
C(7)-C(24)-C(17)	121.8(3)	C(7)-C(24)-H(24)	119.1
C(17)-C(24)-H(24)	119.1	C(26)-C(25)-Si(1)	123.3(3)
C(26)-C(25)-C(30)	116.9(3)	C(30)-C(25)-Si(1)	119.7(3)
C(25)-C(26)-H(26)	119.4	C(27)-C(26)-C(25)	121.3(4)
C(27)-C(26)-H(26)	119.4	C(26)-C(27)-H(27)	119.7
C(28)-C(27)-C(26)	120.5(4)	C(28)-C(27)-H(27)	119.7
C(27)-C(28)-H(28)	120.1	C(27)-C(28)-C(29)	119.8(4)
C(29)-C(28)-H(28)	120.1	C(28)-C(29)-H(29)	120.1
C(28)-C(29)-C(30)	119.8(4)	C(30)-C(29)-H(29)	120.1
C(25)-C(30)-H(30)	119.2	C(29)-C(30)-C(25)	121.6(4)
C(29)-C(30)-H(30)	119.2	C(32)-C(31)-Si(1)	119.3(3)
C(36)-C(31)-Si(1)	123.1(3)	C(36)-C(31)-C(32)	117.2(4)

C(31)-C(32)-H(32)	119.9	C(31)-C(32)-C(33)	120.2(4)
C(33)-C(32)-H(32)	119.9	C(32)-C(33)-H(33)	119.6
C(34)-C(33)-C(32)	120.8(4)	C(34)-C(33)-H(33)	119.6
C(33)-C(34)-H(34)	120.1	C(33)-C(34)-C(35)	119.8(4)
C(35)-C(34)-H(34)	120.1	C(34)-C(35)-H(35)	120.3
C(34)-C(35)-C(36)	119.4(4)	C(36)-C(35)-H(35)	120.3
C(31)-C(36)-H(36)	118.8	C(35)-C(36)-C(31)	122.5(3)
C(35)-C(36)-H(36)	118.8	C(38)-C(37)-Si(2)	123.2(3)
C(38)-C(37)-C(42)	117.1(3)	C(42)-C(37)-Si(2)	119.3(3)
C(37)-C(38)-H(38)	119.5	C(39)-C(38)-C(37)	121.0(4)
C(39)-C(38)-H(38)	119.5	C(38)-C(39)-H(39)	119.9
C(40)-C(39)-C(38)	120.2(4)	C(40)-C(39)-H(39)	119.9
C(39)-C(40)-H(40)	119.9	C(41)-C(40)-C(39)	120.1(4)
C(41)-C(40)-H(40)	119.9	C(40)-C(41)-H(41)	120.3
C(40)-C(41)-C(42)	119.4(4)	C(42)-C(41)-H(41)	120.3
C(37)-C(42)-H(42)	118.9	C(41)-C(42)-C(37)	122.1(4)
C(41)-C(42)-H(42)	118.9	C(44)-C(43)-Si(2)	121.7(3)
C(44)-C(43)-C(48)	116.5(3)	C(48)-C(43)-Si(2)	121.8(3)
C(43)-C(44)-H(44)	118.8	C(45)-C(44)-C(43)	122.4(4)
C(45)-C(44)-H(44)	118.8	C(44)-C(45)-H(45)	120.4
C(46)-C(45)-C(44)	119.3(4)	C(46)-C(45)-H(45)	120.4
C(45)-C(46)-H(46)	120.0	C(47)-C(46)-C(45)	120.0(4)
C(47)-C(46)-H(46)	120.0	C(46)-C(47)-H(47)	119.9
C(46)-C(47)-C(48)	120.1(4)	C(48)-C(47)-H(47)	119.9
C(43)-C(48)-H(48)	119.2	C(47)-C(48)-C(43)	121.7(4)
C(47)-C(48)-H(48)	119.2	C(50)-C(49)-Si(3)	121.5(3)
C(50)-C(49)-C(54)	116.1(4)	C(54)-C(49)-Si(3)	122.4(3)
C(49)-C(50)-H(50)	118.8	C(51)-C(50)-C(49)	122.4(4)
C(51)-C(50)-H(50)	118.8	C(50)-C(51)-H(51)	120.0
C(52)-C(51)-C(50)	119.9(5)	C(52)-C(51)-H(51)	120.0
C(51)-C(52)-H(52)	120.3	C(53)-C(52)-C(51)	119.4(4)
C(53)-C(52)-H(52)	120.3	C(52)-C(53)-H(53)	119.5
C(52)-C(53)-C(54)	121.0(4)	C(54)-C(53)-H(53)	119.5
C(49)-C(54)-H(54)	119.4	C(53)-C(54)-C(49)	121.2(5)
C(53)-C(54)-H(54)	119.4	C(56)-C(55)-Si(3)	119.3(3)
C(56)-C(55)-C(60)	116.7(4)	C(60)-C(55)-Si(3)	123.5(3)

C(55)-C(56)-H(56)	118.8	C(57)-C(56)-C(55)	122.5(4)
C(57)-C(56)-H(56)	118.8	C(56)-C(57)-H(57)	120.3
C(56)-C(57)-C(58)	119.4(4)	C(58)-C(57)-H(57)	120.3
C(57)-C(58)-H(58)	119.9	C(59)-C(58)-C(57)	120.1(4)
C(59)-C(58)-H(58)	119.9	C(58)-C(59)-H(59)	120.0
C(58)-C(59)-C(60)	119.9(4)	C(60)-C(59)-H(59)	120.0
C(55)-C(60)-H(60)	119.3	C(59)-C(60)-C(55)	121.3(4)
C(59)-C(60)-H(60)	119.3	C(62)-C(61)-N(2)	120.2(3)
C(66)-C(61)-N(2)	120.8(3)	C(66)-C(61)-C(62)	119.0(3)
C(61)-C(62)-H(62)	119.8	C(61)-C(62)-C(63)	120.5(3)
C(63)-C(62)-H(62)	119.8	C(62)-C(63)-C(67)	120.3(4)
C(64)-C(63)-C(62)	118.7(4)	C(64)-C(63)-C(67)	121.0(4)
C(63)-C(64)-H(64)	119.1	C(65)-C(64)-C(63)	121.8(4)
C(65)-C(64)-H(64)	119.1	C(64)-C(65)-C(66)	118.2(3)
C(64)-C(65)-C(68)	121.6(3)	C(66)-C(65)-C(68)	120.2(4)
C(61)-C(66)-C(65)	121.8(3)	C(61)-C(66)-H(66)	119.1
C(65)-C(66)-H(66)	119.1	C(63)-C(67)-H(67A)	109.5
C(63)-C(67)-H(67B)	109.5	C(63)-C(67)-H(67C)	109.5
H(67A)-C(67)-H(67B)	109.5	H(67A)-C(67)-H(67C)	109.5
H(67B)-C(67)-H(67C)	109.5	C(65)-C(68)-H(68A)	109.5
C(65)-C(68)-H(68B)	109.5	C(65)-C(68)-H(68C)	109.5
H(68A)-C(68)-H(68B)	109.5	H(68A)-C(68)-H(68C)	109.5
H(68B)-C(68)-H(68C)	109.5	N(2)-C(69)-C(70)	111.3(3)
N(2)-C(69)-C(71)	109.7(3)	N(2)-C(69)-C(72)	108.4(3)
C(70)-C(69)-C(71)	110.0(3)	C(72)-C(69)-C(70)	107.9(3)
C(72)-C(69)-C(71)	109.6(3)	C(69)-C(70)-H(70A)	109.5
C(69)-C(70)-H(70B)	109.5	C(69)-C(70)-H(70C)	109.5
H(70A)-C(70)-H(70B)	109.5	H(70A)-C(70)-H(70C)	109.5
H(70B)-C(70)-H(70C)	109.5	C(69)-C(71)-H(71A)	109.5
C(69)-C(71)-H(71B)	109.5	C(69)-C(71)-H(71C)	109.5
H(71A)-C(71)-H(71B)	109.5	H(71A)-C(71)-H(71C)	109.5
H(71B)-C(71)-H(71C)	109.5	C(69)-C(72)-H(72A)	109.5
C(69)-C(72)-H(72B)	109.5	C(69)-C(72)-H(72C)	109.5
H(72A)-C(72)-H(72B)	109.5	H(72A)-C(72)-H(72C)	109.5
H(72B)-C(72)-H(72C)	109.5	C(74)-C(73)-N(3)	120.5(3)
C(78)-C(73)-N(3)	120.3(3)	C(78)-C(73)-C(74)	119.2(3)

C(73)-C(74)-H(74)	119.4	C(75)-C(74)-C(73)	121.3(4)
C(75)-C(74)-H(74)	119.4	C(74)-C(75)-C(79)	120.7(4)
C(76)-C(75)-C(74)	117.9(3)	C(76)-C(75)-C(79)	121.4(4)
C(75)-C(76)-H(76)	118.8	C(77)-C(76)-C(75)	122.3(4)
C(77)-C(76)-H(76)	118.8	C(76)-C(77)-C(78)	118.7(3)
C(76)-C(77)-C(80)	121.0(4)	C(78)-C(77)-C(80)	120.2(3)
C(73)-C(78)-H(78)	119.7	C(77)-C(78)-C(73)	120.6(3)
C(77)-C(78)-H(78)	119.7	C(75)-C(79)-H(79A)	109.5
C(75)-C(79)-H(79B)	109.5	C(75)-C(79)-H(79C)	109.5
H(79A)-C(79)-H(79B)	109.5	H(79A)-C(79)-H(79C)	109.5
H(79B)-C(79)-H(79C)	109.5	C(77)-C(80)-H(80A)	109.5
C(77)-C(80)-H(80B)	109.5	C(77)-C(80)-H(80C)	109.5
H(80A)-C(80)-H(80B)	109.5	H(80A)-C(80)-H(80C)	109.5
H(80B)-C(80)-H(80C)	109.5	N(3)-C(81)-C(82)	110.2(3)
N(3)-C(81)-C(83)	110.2(3)	N(3)-C(81)-C(84)	108.3(3)
C(82)-C(81)-C(83)	109.9(3)	C(84)-C(81)-C(82)	108.5(3)
C(84)-C(81)-C(83)	109.7(3)	C(81)-C(82)-H(82A)	109.5
C(81)-C(82)-H(82B)	109.5	C(81)-C(82)-H(82C)	109.5
H(82A)-C(82)-H(82B)	109.5	H(82A)-C(82)-H(82C)	109.5
H(82B)-C(82)-H(82C)	109.5	C(81)-C(83)-H(83A)	109.5
C(81)-C(83)-H(83B)	109.5	C(81)-C(83)-H(83C)	109.5
H(83A)-C(83)-H(83B)	109.5	H(83A)-C(83)-H(83C)	109.5
H(83B)-C(83)-H(83C)	109.5	C(81)-C(84)-H(84A)	109.5
C(81)-C(84)-H(84B)	109.5	C(81)-C(84)-H(84C)	109.5
H(84A)-C(84)-H(84B)	109.5	H(84A)-C(84)-H(84C)	109.5
H(84B)-C(84)-H(84C)	109.5	C(86)-C(85)-N(4)	120.9(3)
C(90)-C(85)-N(4)	120.2(3)	C(90)-C(85)-C(86)	118.9(3)
C(85)-C(86)-H(86)	119.3	C(87)-C(86)-C(85)	121.4(3)
C(87)-C(86)-H(86)	119.3	C(86)-C(87)-C(88)	118.3(3)
C(86)-C(87)-C(91)	120.6(3)	C(88)-C(87)-C(91)	121.1(3)
C(87)-C(88)-H(88)	119.1	C(89)-C(88)-C(87)	121.8(3)
C(89)-C(88)-H(88)	119.1	C(88)-C(89)-C(90)	118.5(3)
C(88)-C(89)-C(92)	121.5(3)	C(90)-C(89)-C(92)	120.0(4)
C(85)-C(90)-C(89)	121.0(3)	C(85)-C(90)-H(90)	119.5
C(89)-C(90)-H(90)	119.5	C(87)-C(91)-H(91A)	109.5
C(87)-C(91)-H(91B)	109.5	C(87)-C(91)-H(91C)	109.5

H(91A)-C(91)-H(91B)	109.5	H(91A)-C(91)-H(91C)	109.5
H(91B)-C(91)-H(91C)	109.5	C(89)-C(92)-H(92A)	109.5
C(89)-C(92)-H(92B)	109.5	C(89)-C(92)-H(92C)	109.5
H(92A)-C(92)-H(92B)	109.5	H(92A)-C(92)-H(92C)	109.5
H(92B)-C(92)-H(92C)	109.5	N(4)-C(93)-C(94B)	108.7(5)
N(4)-C(93)-C(95B)	112.9(5)	N(4)-C(93)-C(96A)	107.0(3)
N(4)-C(93)-C(96B)	115.0(5)	C(94A)-C(93)-N(4)	112.5(3)
C(94A)-C(93)-C(96A)	107.3(4)	C(94B)-C(93)-C(95B)	104.5(7)
C(95A)-C(93)-N(4)	106.7(3)	C(95A)-C(93)-C(94A)	114.4(4)
C(95A)-C(93)-C(96A)	108.6(4)	C(96B)-C(93)-C(94B)	110.2(7)
C(96B)-C(93)-C(95B)	105.1(7)	C(93)-C(94A)-H(94A)	109.5
C(93)-C(94A)-H(94B)	109.5	C(93)-C(94A)-H(94C)	109.5
H(94A)-C(94A)-H(94B)	109.5	H(94A)-C(94A)-H(94C)	109.5
H(94B)-C(94A)-H(94C)	109.5	C(93)-C(94B)-H(94D)	109.5
C(93)-C(94B)-H(94E)	109.5	C(93)-C(94B)-H(94F)	109.5
H(94D)-C(94B)-H(94E)	109.5	H(94D)-C(94B)-H(94F)	109.5
H(94E)-C(94B)-H(94F)	109.5	C(93)-C(95A)-H(95A)	109.5
C(93)-C(95A)-H(95B)	109.5	C(93)-C(95A)-H(95C)	109.5
H(95A)-C(95A)-H(95B)	109.5	H(95A)-C(95A)-H(95C)	109.5
H(95B)-C(95A)-H(95C)	109.5	C(93)-C(95B)-H(95D)	109.5
C(93)-C(95B)-H(95E)	109.5	C(93)-C(95B)-H(95F)	109.5
H(95D)-C(95B)-H(95E)	109.5	H(95D)-C(95B)-H(95F)	109.5
H(95E)-C(95B)-H(95F)	109.5	C(93)-C(96A)-H(96A)	109.5
C(93)-C(96A)-H(96B)	109.5	C(93)-C(96A)-H(96C)	109.5
H(96A)-C(96A)-H(96B)	109.5	H(96A)-C(96A)-H(96C)	109.5
H(96B)-C(96A)-H(96C)	109.5	C(93)-C(96B)-H(96D)	109.5
C(93)-C(96B)-H(96E)	109.5	C(93)-C(96B)-H(96F)	109.5
H(96D)-C(96B)-H(96E)	109.5	H(96D)-C(96B)-H(96F)	109.5
H(96E)-C(96B)-H(96F)	109.5	H-C(1B)-HA	109.5
H-C(1B)-HB	109.5	HA-C(1B)-HB	109.5
C(2B)-C(1B)-H	109.5	C(2B)-C(1B)-HA	109.5
C(2B)-C(1B)-HB	109.5	HC-C(1D)-HD	109.5
HC-C(1D)-HE	109.5	HD-C(1D)-HE	109.5
C(2D)-C(1D)-HC	109.5	C(2D)-C(1D)-HD	109.5
C(2D)-C(1D)-HE	109.5	C(1B)-C(2B)-HF	109.6
C(1B)-C(2B)-HG	109.6	C(1B)-C(2B)-C(3B)	110.2(18)

HF-C(2B)-HG	108.1	C(3B)-C(2B)-HF	109.6
C(3B)-C(2B)-HG	109.6	C(1D)-C(2D)-HH	107.7
C(1D)-C(2D)-HI	107.7	HH-C(2D)-HI	107.1
C(3D)-C(2D)-C(1D)	118.6(18)	C(3D)-C(2D)-HH	107.7
C(3D)-C(2D)-HI	107.7	C(2B)-C(3B)-HJ	109.3
C(2B)-C(3B)-HK	109.3	HJ-C(3B)-HK	107.9
C(4B)-C(3B)-C(2B)	112(2)	C(4B)-C(3B)-HJ	109.3
C(4B)-C(3B)-HK	109.3	C(2D)-C(3D)-HL	107.8
C(2D)-C(3D)-HM	107.8	C(2D)-C(3D)-C(4D)	118.2(19)
HL-C(3D)-HM	107.1	C(4D)-C(3D)-HL	107.8
C(4D)-C(3D)-HM	107.8	C(3B)-C(4B)-HN	108.2
C(3B)-C(4B)-HO	108.2	C(3B)-C(4B)-C(5B)	116.3(19)
HN-C(4B)-HO	107.4	C(5B)-C(4B)-HN	108.2
C(5B)-C(4B)-HO	108.2	C(3D)-C(4D)-HP	108.3
C(3D)-C(4D)-HQ	108.3	C(3D)-C(4D)-C(5D)	115.9(18)
HP-C(4D)-HQ	107.4	C(5D)-C(4D)-HP	108.3
C(5D)-C(4D)-HQ	108.3	C(4B)-C(5B)-HR	109.5
C(4B)-C(5B)-HS	109.5	C(4B)-C(5B)-HT	109.5
HR-C(5B)-HS	109.5	HR-C(5B)-HT	109.5
HS-C(5B)-HT	109.5	C(4D)-C(5D)-HU	109.5
C(4D)-C(5D)-HV	109.5	C(4D)-C(5D)-HW	109.5
HU-C(5D)-HV	109.5	HU-C(5D)-HW	109.5
HV-C(5D)-HW	109.5	C(5A)-C(6A)-HX	120.0
C(5A)-C(6A)-C(1A)	120.0	C(1A)-C(6A)-HX	120.0
C(6A)-C(5A)-HY	120.0	C(4A)-C(5A)-C(6A)	120.0
C(4A)-C(5A)-HY	120.0	C(5A)-C(4A)-HZ	120.0
C(5A)-C(4A)-C(3A)	120.0	C(3A)-C(4A)-HZ	120.0
C(4A)-C(3A)-H(1)	120.0	C(4A)-C(3A)-C(2A)	120.0
C(2A)-C(3A)-H(1)	120.0	C(3A)-C(2A)-H(6)	120.0
C(1A)-C(2A)-C(3A)	120.0	C(1A)-C(2A)-H(6)	120.0
C(6A)-C(1A)-H(7)	120.0	C(2A)-C(1A)-C(6A)	120.0
C(2A)-C(1A)-H(7)	120.0	C(5C)-C(6C)-H(9)	120.0
C(5C)-C(6C)-C(1C)	120.0	C(1C)-C(6C)-H(9)	120.0
C(6C)-C(5C)-H(10)	120.0	C(4C)-C(5C)-C(6C)	120.0
C(4C)-C(5C)-H(10)	120.0	C(5C)-C(4C)-H(11)	120.0
C(5C)-C(4C)-C(3C)	120.0	C(3C)-C(4C)-H(11)	120.0

C(4C)-C(3C)-H(17)	120.0	C(2C)-C(3C)-C(4C)	120.0
C(2C)-C(3C)-H(17)	120.0	C(3C)-C(2C)-H(18)	120.0
C(3C)-C(2C)-C(1C)	120.0	C(1C)-C(2C)-H(18)	120.0
C(6C)-C(1C)-H(19)	120.0	C(2C)-C(1C)-C(6C)	120.0
C(2C)-C(1C)-H(19)	120.0		

Single crystal structure analysis of complex 13b · pentane/toluene solvate

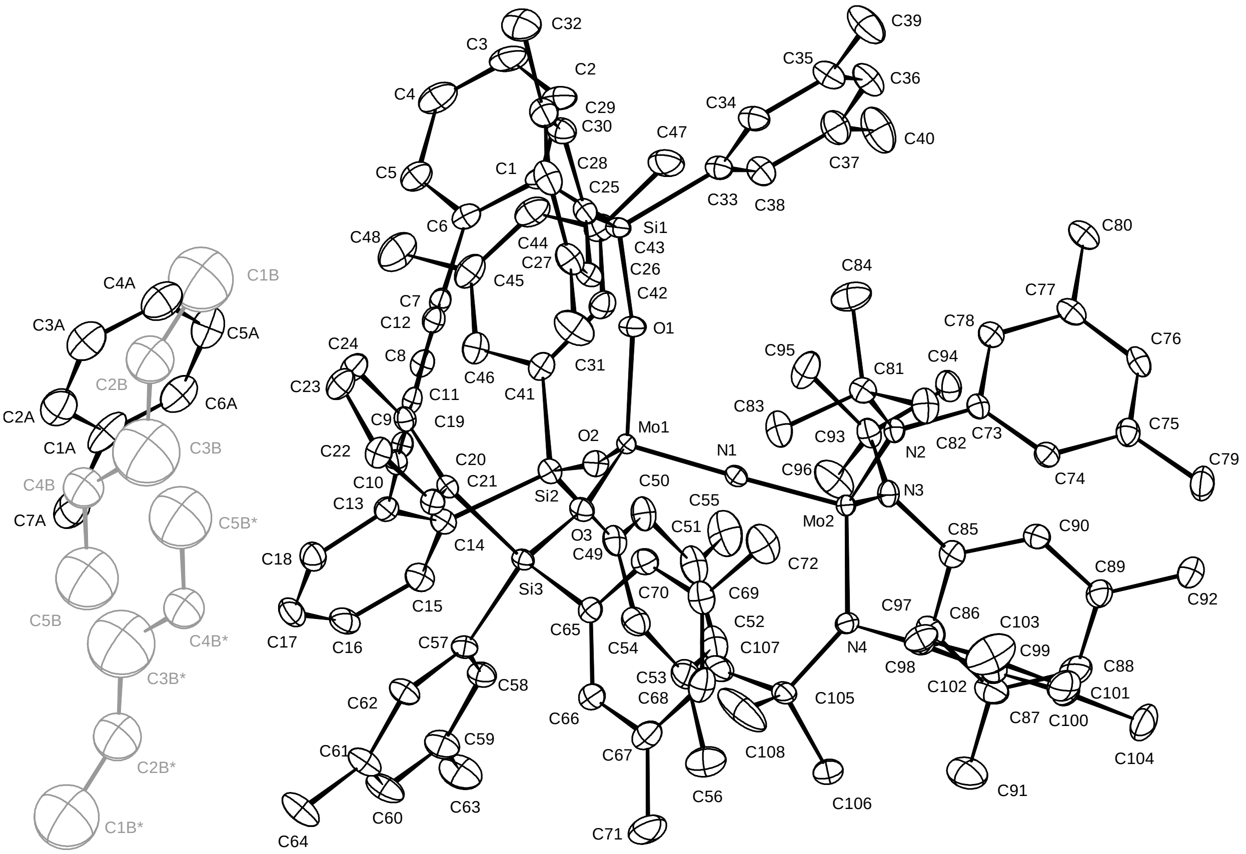

Figure S 14. The molecular structure of complex **13b · pentane/toluene solvate**. H atoms have been removed for clarity. Main structure shown in black and disordered parts shown in grey.

X-ray Crystal Structure Analysis of complex 13b · pentane/toluene solvate:

C_{458} H_{528} Mo_8 N_{16} O_{12} Si_{12}, M_r = 7553.53 g mol^{-1}, green plate, crystal size 0.197 x 0.143 x 0.04 mm^3, Monoclinic, space group $P2_1/n$ [14], a = 14.8017(7) Å, b = 29.0993(15) Å, c = 23.9781(12) Å, β = 92.440(2)°, V = 10318.5(9) Å^3, T = 100(2) K, Z = 1, D_{calc} = 1.216 g·cm^3, λ = 0.71073 Å, $\mu(Mo\text{-}K_\alpha)$ = 0.330 mm^{-1}, Gaussian absorption correction ($T_{\min}$ = 0.95295, $T_{\max}$ = 0.99184), Bruker-AXS Kappa Mach3 with APEX-II detector and IµS microfocus Mo-anode X-ray source, 1.101 < θ < 30.999°, 348764 measured reflections, 32908 independent reflections, 23936 reflections with $I > 2\sigma(I)$, R_{int} = 0.0847. The structure was solved by *SHELXT* and refined by full-matrix least-squares (*SHELXL*) against F^2 to R_1 = 0.0398 [$I > 2\sigma(I)$], wR_2 = 0.0933 [all data], 1194 parameters and 20 restraints.

Full .cif data for the compound are available under the CCDC number **CCDC-2265454**

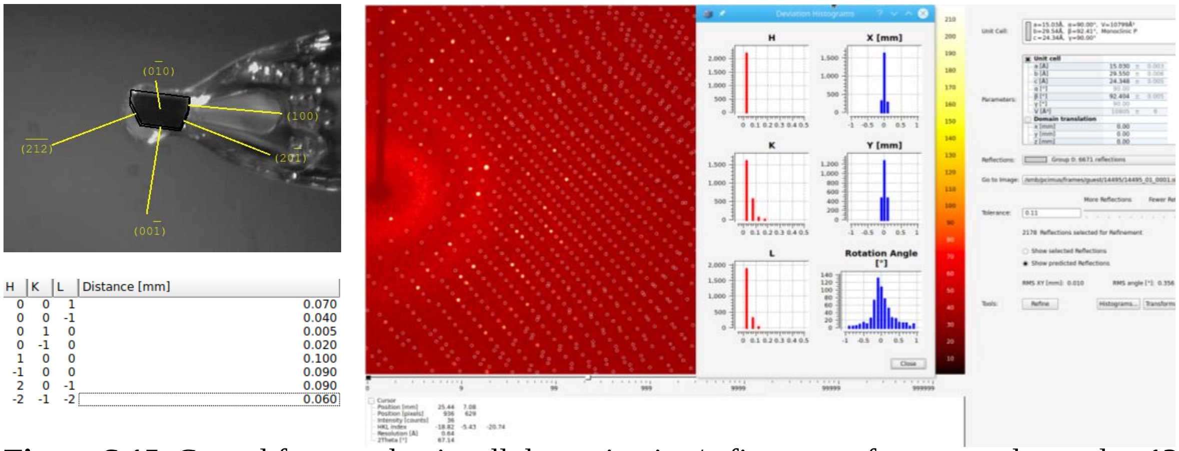

Figure S 15. Crystal faces and unit cell determination/refinement of compound complex **13b · pentane/toluene solvate**.

INTENSITY STATISTICS FOR DATASET

Resolution	#Data	#Theory	%Complete	Redundancy	Mean I	Mean I/s	Rmerge	Rsigma
Inf - 2.41	818	820	99.8	24.67	41.47	77.84	0.0241	0.0088
2.41 - 1.60	1928	1928	100.0	28.44	18.53	59.74	0.0428	0.0113
1.60 - 1.27	2708	2708	100.0	28.76	12.92	47.37	0.0605	0.0148
1.27 - 1.11	2668	2668	100.0	27.40	9.02	33.78	0.0863	0.0214
1.11 - 1.00	2952	2952	100.0	21.86	7.34	23.65	0.1102	0.0319
1.00 - 0.93	2663	2663	100.0	18.35	5.38	16.14	0.1510	0.0479
0.93 - 0.87	3012	3012	100.0	16.32	5.11	13.89	0.1632	0.0567
0.87 - 0.83	2520	2520	100.0	15.29	4.48	11.66	0.1902	0.0688
0.83 - 0.79	3078	3078	100.0	14.58	4.00	9.80	0.2187	0.0817
0.79 - 0.76	2736	2736	100.0	13.91	3.13	7.66	0.2710	0.1077
0.76 - 0.74	2040	2040	100.0	13.56	2.76	6.69	0.3107	0.1272
0.74 - 0.71	3574	3574	100.0	12.95	2.61	6.06	0.3360	0.1431
0.71 - 0.69	2755	2755	100.0	12.32	2.29	5.10	0.3828	0.1747
0.69 - 0.67	3030	3030	100.0	12.01	2.06	4.49	0.4237	0.2031
0.67 - 0.66	1698	1700	99.9	11.51	1.81	3.75	0.4857	0.2429
0.66 - 0.64	3647	3653	99.8	11.26	1.55	3.21	0.5351	0.2919
0.64 - 0.63	2011	2026	99.3	10.84	1.42	2.82	0.5825	0.3339
0.63 - 0.62	2134	2145	99.5	10.70	1.26	2.52	0.6234	0.3854
0.62 - 0.60	4667	4720	98.9	10.15	1.10	2.13	0.6787	0.4668
0.60 - 0.59	2548	2600	98.0	6.41	0.81	1.09	0.7972	0.9926
0.59 - 0.58	842	1729	48.7	1.07	0.71	0.39	1.0137	2.2884
0.82 - 0.80	298	298	100.0	8.86	5.98	10.49	0.1807	0.0911
0.68 - 0.58	19113	20139	94.9	9.46	1.31	2.55	0.5872	0.4326
Inf - 0.58	54029	55057	98.1	15.01	4.85	13.65	0.1306	0.0814

A resolution cut off (SHEL 999 0.69) was applied to exclude poorly determined reflections at high diffraction angles. Three reflection were omitted (OMIT: 1 0 1; 1 3 2 and 1 0 5) from the data set prior to the final refinement cycles because of high I/σI (> 10). Two disordered solute molecules are present in the unit cell sharing approximately the same positions. The DSR tool as a plugin in Olex2 was used to describe the disorder in combination with several restraints. Isotropic atomic displacement parameters were used for the pentane solute molecule. An additional solvent molecule could not be properly refined, so a solvent mask (SQUEEZE routine in Olex2) was

applied to exclude the remaining residual electron density. This procedure resulted in a structure model with a void volume of 693 Å³, corresponding to 6.7% of the unit cell volume (probe radius 1.2 Å and grid spacing approx. 0.7 Å using the CCDC software program Mercury).

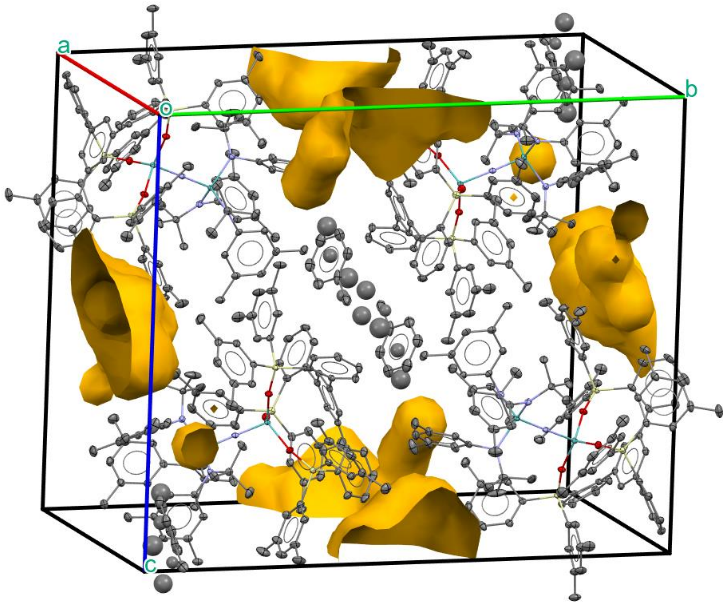

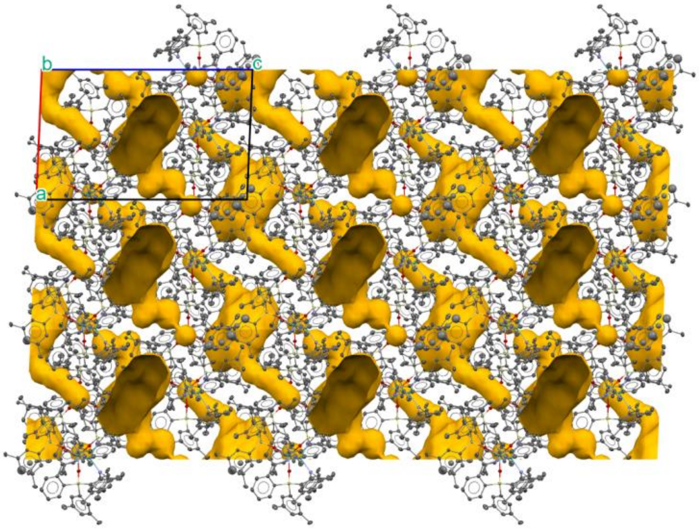

Figure S 16. Solvent accessible voids (yellow surface) in the structure of complex **13b · pentane/toluene solvate** in the unit cell in a random orientation (top) and along the crystallographic b axis in 3x3x3 packing extension (bottom).

Table S 13. Crystal data and structure refinement of complex **13b · pentane/toluene solvate**.

Identification code	14495	
Empirical formula	C_{458} H_{528} Mo_8 N_{16} O_{12} Si_{12}	
Color	green	
Formula weight	7553.53 g·mol^{-1}	
Temperature	100(2) K	
Wavelength	0.71073 Å	
Crystal system	Monoclinic	
Space group	$P2_1/n$, (no. 14)	
Unit cell dimensions	a = 14.8017(7) Å	α= 90°.
	b = 29.0993(15) Å	β= 92.440(2)°.
	c = 23.9781(12) Å	γ = 90°.
Volume	10318.5(9) Å^3	
Z	1	
Density (calculated)	1.216 Mg·m^{-3}	
Absorption coefficient	0.330 mm^{-1}	
F(000)	3988 e	
Crystal size	0.197 x 0.143 x 0.04 mm^3	
θ range for data collection	1.101 to 30.999°.	
Index ranges	-21 ≤ h ≤ 21, -42≤ k ≤ 42, -34 ≤ l ≤ 34	
Reflections collected	348764	
Independent reflections	32908 [R_{int} = 0.0847]	
Reflections with I>2σ(I)	23936	
Completeness to θ = 25.242°	100.0 %	
Absorption correction	Gaussian	
Max. and min. transmission	0.99184 and 0.95295	
Refinement method	Full-matrix least-squares on F^2	
Data / restraints / parameters	32908 / 20 / 1194	
Goodness-of-fit on F^2	1.019	
Final R indices [I>2σ(I)]	R_1 = 0.0398	wR^2 = 0.0830
R indices (all data)	R_1 = 0.0687	wR^2 = 0.0933
Extinction coefficient	n/a	
Largest diff. peak and hole	0.533 and -0.672 e·Å^{-3}	

Table S 14. Bond lengths [Å] and angles [°] of complex **13b · pentane/toluene solvate**.

Mo(1)-O(1)	1.9174(13)	Mo(1)-O(2)	1.8996(12)
Mo(1)-O(3)	1.9088(12)	Mo(1)-N(1)	1.8253(15)
Mo(2)-N(1)	1.8077(15)	Mo(2)-N(2)	1.9690(15)
Mo(2)-N(3)	1.9788(14)	Mo(2)-N(4)	1.9632(15)
Si(1)-O(1)	1.6248(14)	Si(1)-C(1)	1.894(2)
Si(1)-C(25)	1.8709(18)	Si(1)-C(33)	1.882(2)
Si(2)-O(2)	1.6291(13)	Si(2)-C(14)	1.882(2)
Si(2)-C(41)	1.8692(19)	Si(2)-C(49)	1.8734(19)
Si(3)-O(3)	1.6306(13)	Si(3)-C(20)	1.8806(18)
Si(3)-C(57)	1.8652(19)	Si(3)-C(65)	1.8803(19)
N(2)-C(73)	1.440(2)	N(2)-C(81)	1.502(2)
N(3)-C(85)	1.442(2)	N(3)-C(93)	1.497(2)
N(4)-C(97)	1.447(2)	N(4)-C(105)	1.501(2)
C(1)-C(2)	1.408(3)	C(1)-C(6)	1.407(3)
C(2)-H(2)	0.9500	C(2)-C(3)	1.384(3)
C(3)-H(3)	0.9500	C(3)-C(4)	1.376(3)
C(4)-H(4)	0.9500	C(4)-C(5)	1.386(3)
C(5)-H(5)	0.9500	C(5)-C(6)	1.400(3)
C(6)-C(7)	1.485(3)	C(7)-C(8)	1.394(3)
C(7)-C(12)	1.397(2)	C(8)-H(8)	0.9500
C(8)-C(9)	1.391(3)	C(9)-C(10)	1.394(2)
C(9)-C(13)	1.488(3)	C(10)-H(10)	0.9500
C(10)-C(11)	1.397(3)	C(11)-C(12)	1.392(3)
C(11)-C(19)	1.490(2)	C(12)-H(12)	0.9500
C(13)-C(14)	1.413(3)	C(13)-C(18)	1.394(3)
C(14)-C(15)	1.401(3)	C(15)-H(15)	0.9500
C(15)-C(16)	1.382(3)	C(16)-H(16)	0.9500
C(16)-C(17)	1.390(3)	C(17)-H(17)	0.9500
C(17)-C(18)	1.382(3)	C(18)-H(18)	0.9500
C(19)-C(20)	1.409(2)	C(19)-C(24)	1.392(3)
C(20)-C(21)	1.403(2)	C(21)-H(21)	0.9500
C(21)-C(22)	1.385(3)	C(22)-H(22)	0.9500
C(22)-C(23)	1.385(3)	C(23)-H(23)	0.9500
C(23)-C(24)	1.386(3)	C(24)-H(24)	0.9500

C(25)-C(26)	1.393(3)	C(25)-C(30)	1.399(3)
C(26)-H(26)	0.9500	C(26)-C(27)	1.396(3)
C(27)-C(28)	1.390(3)	C(27)-C(31)	1.506(3)
C(28)-H(28)	0.9500	C(28)-C(29)	1.388(3)
C(29)-C(30)	1.391(3)	C(29)-C(32)	1.511(3)
C(30)-H(30)	0.9500	C(31)-H(31A)	0.9800
C(31)-H(31B)	0.9800	C(31)-H(31C)	0.9800
C(32)-H(32A)	0.9800	C(32)-H(32B)	0.9800
C(32)-H(32C)	0.9800	C(33)-C(34)	1.397(3)
C(33)-C(38)	1.397(3)	C(34)-H(34)	0.9500
C(34)-C(35)	1.392(3)	C(35)-C(36)	1.389(3)
C(35)-C(39)	1.507(3)	C(36)-H(36)	0.9500
C(36)-C(37)	1.389(3)	C(37)-C(38)	1.393(3)
C(37)-C(40)	1.511(3)	C(38)-H(38)	0.9500
C(39)-H(39A)	0.9800	C(39)-H(39B)	0.9800
C(39)-H(39C)	0.9800	C(40)-H(40A)	0.9800
C(40)-H(40B)	0.9800	C(40)-H(40C)	0.9800
C(41)-C(42)	1.396(3)	C(41)-C(46)	1.397(3)
C(42)-H(42)	0.9500	C(42)-C(43)	1.391(3)
C(43)-C(44)	1.391(3)	C(43)-C(47)	1.503(3)
C(44)-H(44)	0.9500	C(44)-C(45)	1.392(3)
C(45)-C(46)	1.390(3)	C(45)-C(48)	1.507(3)
C(46)-H(46)	0.9500	C(47)-H(47A)	0.9800
C(47)-H(47B)	0.9800	C(47)-H(47C)	0.9800
C(48)-H(48A)	0.9800	C(48)-H(48B)	0.9800
C(48)-H(48C)	0.9800	C(49)-C(50)	1.396(3)
C(49)-C(54)	1.398(3)	C(50)-H(50)	0.9500
C(50)-C(51)	1.397(3)	C(51)-C(52)	1.384(3)
C(51)-C(55)	1.512(3)	C(52)-H(52)	0.9500
C(52)-C(53)	1.394(3)	C(53)-C(54)	1.392(3)
C(53)-C(56)	1.506(3)	C(54)-H(54)	0.9500
C(55)-H(55A)	0.9800	C(55)-H(55B)	0.9800
C(55)-H(55C)	0.9800	C(56)-H(56A)	0.9800
C(56)-H(56B)	0.9800	C(56)-H(56C)	0.9800
C(57)-C(58)	1.393(3)	C(57)-C(62)	1.402(3)
C(58)-H(58)	0.9500	C(58)-C(59)	1.393(3)

C(59)-C(60)	1.388(3)	C(59)-C(63)	1.507(3)
C(60)-H(60)	0.9500	C(60)-C(61)	1.394(3)
C(61)-C(62)	1.384(3)	C(61)-C(64)	1.510(3)
C(62)-H(62)	0.9500	C(63)-H(63A)	0.9800
C(63)-H(63B)	0.9800	C(63)-H(63C)	0.9800
C(64)-H(64A)	0.9800	C(64)-H(64B)	0.9800
C(64)-H(64C)	0.9800	C(65)-C(66)	1.396(3)
C(65)-C(70)	1.400(3)	C(66)-H(66)	0.9500
C(66)-C(67)	1.396(3)	C(67)-C(68)	1.389(3)
C(67)-C(71)	1.511(3)	C(68)-H(68)	0.9500
C(68)-C(69)	1.396(3)	C(69)-C(70)	1.388(3)
C(69)-C(72)	1.508(3)	C(70)-H(70)	0.9500
C(71)-H(71A)	0.9800	C(71)-H(71B)	0.9800
C(71)-H(71C)	0.9800	C(72)-H(72A)	0.9800
C(72)-H(72B)	0.9800	C(72)-H(72C)	0.9800
C(73)-C(74)	1.394(3)	C(73)-C(78)	1.398(3)
C(74)-H(74)	0.9500	C(74)-C(75)	1.393(3)
C(75)-C(76)	1.390(3)	C(75)-C(79)	1.504(3)
C(76)-H(76)	0.9500	C(76)-C(77)	1.392(3)
C(77)-C(78)	1.393(3)	C(77)-C(80)	1.511(3)
C(78)-H(78)	0.9500	C(79)-H(79A)	0.9800
C(79)-H(79B)	0.9800	C(79)-H(79C)	0.9800
C(80)-H(80A)	0.9800	C(80)-H(80B)	0.9800
C(80)-H(80C)	0.9800	C(81)-C(82)	1.531(3)
C(81)-C(83)	1.516(3)	C(81)-C(84)	1.521(3)
C(82)-H(82A)	0.9800	C(82)-H(82B)	0.9800
C(82)-H(82C)	0.9800	C(83)-H(83A)	0.9800
C(83)-H(83B)	0.9800	C(83)-H(83C)	0.9800
C(84)-H(84A)	0.9800	C(84)-H(84B)	0.9800
C(84)-H(84C)	0.9800	C(85)-C(86)	1.394(3)
C(85)-C(90)	1.394(3)	C(86)-H(86)	0.9500
C(86)-C(87)	1.395(3)	C(87)-C(88)	1.392(3)
C(87)-C(91)	1.505(3)	C(88)-H(88)	0.9500
C(88)-C(89)	1.393(3)	C(89)-C(90)	1.392(3)
C(89)-C(92)	1.507(3)	C(90)-H(90)	0.9500
C(91)-H(91A)	0.9800	C(91)-H(91B)	0.9800

C(91)-H(91C)	0.9800	C(92)-H(92A)	0.9800
C(92)-H(92B)	0.9800	C(92)-H(92C)	0.9800
C(93)-C(94)	1.530(3)	C(93)-C(95)	1.512(3)
C(93)-C(96)	1.530(3)	C(94)-H(94A)	0.9800
C(94)-H(94B)	0.9800	C(94)-H(94C)	0.9800
C(95)-H(95A)	0.9800	C(95)-H(95B)	0.9800
C(95)-H(95C)	0.9800	C(96)-H(96A)	0.9800
C(96)-H(96B)	0.9800	C(96)-H(96C)	0.9800
C(97)-C(98)	1.400(3)	C(97)-C(102)	1.388(3)
C(98)-H(98)	0.9500	C(98)-C(99)	1.392(3)
C(99)-C(100)	1.390(3)	C(99)-C(103)	1.513(3)
C(100)-H(100)	0.9500	C(100)-C(101)	1.390(3)
C(101)-C(102)	1.393(3)	C(101)-C(104)	1.507(3)
C(102)-H(102)	0.9500	C(103)-H(10A)	0.9800
C(103)-H(10B)	0.9800	C(103)-H(10C)	0.9800
C(104)-H(10D)	0.9800	C(104)-H(10E)	0.9800
C(104)-H(10F)	0.9800	C(105)-C(106)	1.524(3)
C(105)-C(107)	1.517(3)	C(105)-C(108)	1.512(3)
C(106)-H(10G)	0.9800	C(106)-H(10H)	0.9800
C(106)-H(10I)	0.9800	C(107)-H(10J)	0.9800
C(107)-H(10K)	0.9800	C(107)-H(10L)	0.9800
C(108)-H(10M)	0.9800	C(108)-H(10N)	0.9800
C(108)-H(10O)	0.9800	C(1A)-C(2A)	1.395(4)
C(1A)-C(2B)	1.847(14)	C(1A)-C(3B)	1.230(19)
C(1A)-C(4B)	0.970(11)	C(1A)-C(6A)	1.328(5)
C(1A)-C(7A)	1.606(5)	C(1B)-H(1BA)	0.9800
C(1B)-H(1BB)	0.9800	C(1B)-H(1BC)	0.9800
C(1B)-C(2B)	1.522(12)	C(1B)-C(4A)	0.860(19)
C(1B)-C(5A)	0.99(2)	C(2A)-H(2A)	0.9500
C(2A)-C(2B)	1.647(14)	C(2A)-C(3A)	1.385(5)
C(2A)-C(3B)	1.57(2)	C(2A)-C(4B)	1.672(12)
C(2B)-H(2BA)	0.9900	C(2B)-H(2BB)	0.9900
C(2B)-C(3A)	1.218(14)	C(2B)-C(3B)	1.504(11)
C(2B)-C(4A)	1.004(12)	C(2B)-C(5A)	1.317(14)
C(2B)-C(6A)	1.712(15)	C(3A)-H(3A)	0.9500
C(3A)-C(4A)	1.390(5)	C(3B)-H(3BA)	0.9900

C(3B)-H(3BB)	0.9900	C(3B)-C(4B)	1.465(11)
C(3B)-C(6A)	1.68(2)	C(4A)-H(4A)	0.9500
C(4A)-C(5A)	1.390(5)	C(4B)-H(4BA)	0.9900
C(4B)-H(4BB)	0.9900	C(4B)-C(5B)	1.471(11)
C(4B)-C(7A)	1.052(11)	C(5A)-H(5A)	0.9500
C(5A)-C(6A)	1.387(5)	C(5B)-H(5BA)	0.9800
C(5B)-H(5BB)	0.9800	C(5B)-H(5BC)	0.9800
C(5B)-C(7A)	1.335(18)	C(6A)-H(6A)	0.9500
C(7A)-H(7AA)	0.9800	C(7A)-H(7AB)	0.9800
C(7A)-H(7AC)	0.9800		
O(2)-Mo(1)-O(1)	112.32(6)	O(2)-Mo(1)-O(3)	121.57(6)
O(3)-Mo(1)-O(1)	113.33(5)	N(1)-Mo(1)-O(1)	103.19(6)
N(1)-Mo(1)-O(2)	101.23(6)	N(1)-Mo(1)-O(3)	101.83(6)
N(1)-Mo(2)-N(2)	103.39(6)	N(1)-Mo(2)-N(3)	106.85(6)
N(1)-Mo(2)-N(4)	105.19(6)	N(2)-Mo(2)-N(3)	116.85(6)
N(4)-Mo(2)-N(2)	112.10(6)	N(4)-Mo(2)-N(3)	111.30(6)
O(1)-Si(1)-C(1)	112.08(8)	O(1)-Si(1)-C(25)	110.87(8)
O(1)-Si(1)-C(33)	110.40(8)	C(25)-Si(1)-C(1)	110.81(8)
C(25)-Si(1)-C(33)	106.73(9)	C(33)-Si(1)-C(1)	105.70(9)
O(2)-Si(2)-C(14)	111.50(7)	O(2)-Si(2)-C(41)	110.85(8)
O(2)-Si(2)-C(49)	108.83(8)	C(41)-Si(2)-C(14)	110.57(9)
C(41)-Si(2)-C(49)	108.10(8)	C(49)-Si(2)-C(14)	106.84(9)
O(3)-Si(3)-C(20)	111.49(7)	O(3)-Si(3)-C(57)	111.58(7)
O(3)-Si(3)-C(65)	109.93(7)	C(57)-Si(3)-C(20)	110.16(8)
C(57)-Si(3)-C(65)	105.66(8)	C(65)-Si(3)-C(20)	107.79(8)
Si(1)-O(1)-Mo(1)	168.88(9)	Si(2)-O(2)-Mo(1)	167.45(9)
Si(3)-O(3)-Mo(1)	166.68(8)	Mo(2)-N(1)-Mo(1)	178.09(10)
C(73)-N(2)-Mo(2)	109.90(11)	C(73)-N(2)-C(81)	114.89(14)
C(81)-N(2)-Mo(2)	135.12(12)	C(85)-N(3)-Mo(2)	113.42(11)
C(85)-N(3)-C(93)	113.92(14)	C(93)-N(3)-Mo(2)	132.63(12)
C(97)-N(4)-Mo(2)	112.63(12)	C(97)-N(4)-C(105)	114.84(14)
C(105)-N(4)-Mo(2)	132.44(12)	C(2)-C(1)-Si(1)	117.59(16)
C(6)-C(1)-Si(1)	125.32(14)	C(6)-C(1)-C(2)	117.03(18)
C(1)-C(2)-H(2)	119.0	C(3)-C(2)-C(1)	122.0(2)
C(3)-C(2)-H(2)	119.0	C(2)-C(3)-H(3)	119.9

C(4)-C(3)-C(2)	120.2(2)	C(4)-C(3)-H(3)	119.9
C(3)-C(4)-H(4)	120.2	C(3)-C(4)-C(5)	119.6(2)
C(5)-C(4)-H(4)	120.2	C(4)-C(5)-H(5)	119.6
C(4)-C(5)-C(6)	120.9(2)	C(6)-C(5)-H(5)	119.6
C(1)-C(6)-C(7)	121.47(16)	C(5)-C(6)-C(1)	120.36(18)
C(5)-C(6)-C(7)	118.15(18)	C(8)-C(7)-C(6)	119.52(16)
C(8)-C(7)-C(12)	118.68(17)	C(12)-C(7)-C(6)	121.80(17)
C(7)-C(8)-H(8)	119.2	C(9)-C(8)-C(7)	121.60(16)
C(9)-C(8)-H(8)	119.2	C(8)-C(9)-C(10)	118.73(17)
C(8)-C(9)-C(13)	119.19(16)	C(10)-C(9)-C(13)	122.07(17)
C(9)-C(10)-H(10)	119.6	C(9)-C(10)-C(11)	120.77(17)
C(11)-C(10)-H(10)	119.6	C(10)-C(11)-C(19)	120.83(16)
C(12)-C(11)-C(10)	119.42(16)	C(12)-C(11)-C(19)	119.75(16)
C(7)-C(12)-H(12)	119.6	C(11)-C(12)-C(7)	120.74(17)
C(11)-C(12)-H(12)	119.6	C(14)-C(13)-C(9)	121.08(17)
C(18)-C(13)-C(9)	119.14(17)	C(18)-C(13)-C(14)	119.75(17)
C(13)-C(14)-Si(2)	125.21(14)	C(15)-C(14)-Si(2)	117.28(14)
C(15)-C(14)-C(13)	117.49(17)	C(14)-C(15)-H(15)	118.9
C(16)-C(15)-C(14)	122.17(18)	C(16)-C(15)-H(15)	118.9
C(15)-C(16)-H(16)	120.2	C(15)-C(16)-C(17)	119.57(19)
C(17)-C(16)-H(16)	120.2	C(16)-C(17)-H(17)	120.2
C(18)-C(17)-C(16)	119.51(19)	C(18)-C(17)-H(17)	120.2
C(13)-C(18)-H(18)	119.4	C(17)-C(18)-C(13)	121.28(18)
C(17)-C(18)-H(18)	119.4	C(20)-C(19)-C(11)	121.98(16)
C(24)-C(19)-C(11)	118.15(16)	C(24)-C(19)-C(20)	119.87(16)
C(19)-C(20)-Si(3)	125.94(13)	C(21)-C(20)-Si(3)	116.58(13)
C(21)-C(20)-C(19)	117.47(16)	C(20)-C(21)-H(21)	118.9
C(22)-C(21)-C(20)	122.18(17)	C(22)-C(21)-H(21)	118.9
C(21)-C(22)-H(22)	120.3	C(23)-C(22)-C(21)	119.49(17)
C(23)-C(22)-H(22)	120.3	C(22)-C(23)-H(23)	120.2
C(22)-C(23)-C(24)	119.55(18)	C(24)-C(23)-H(23)	120.2
C(19)-C(24)-H(24)	119.3	C(23)-C(24)-C(19)	121.31(18)
C(23)-C(24)-H(24)	119.3	C(26)-C(25)-Si(1)	122.69(15)
C(26)-C(25)-C(30)	118.10(17)	C(30)-C(25)-Si(1)	119.02(14)
C(25)-C(26)-H(26)	119.2	C(25)-C(26)-C(27)	121.54(18)
C(27)-C(26)-H(26)	119.2	C(26)-C(27)-C(31)	121.12(19)

C(28)-C(27)-C(26)	118.30(18)	C(28)-C(27)-C(31)	120.57(18)
C(27)-C(28)-H(28)	119.0	C(29)-C(28)-C(27)	122.03(18)
C(29)-C(28)-H(28)	119.0	C(28)-C(29)-C(30)	118.21(19)
C(28)-C(29)-C(32)	120.83(18)	C(30)-C(29)-C(32)	120.96(19)
C(25)-C(30)-H(30)	119.1	C(29)-C(30)-C(25)	121.80(19)
C(29)-C(30)-H(30)	119.1	C(27)-C(31)-H(31A)	109.5
C(27)-C(31)-H(31B)	109.5	C(27)-C(31)-H(31C)	109.5
H(31A)-C(31)-H(31B)	109.5	H(31A)-C(31)-H(31C)	109.5
H(31B)-C(31)-H(31C)	109.5	C(29)-C(32)-H(32A)	109.5
C(29)-C(32)-H(32B)	109.5	C(29)-C(32)-H(32C)	109.5
H(32A)-C(32)-H(32B)	109.5	H(32A)-C(32)-H(32C)	109.5
H(32B)-C(32)-H(32C)	109.5	C(34)-C(33)-Si(1)	122.40(14)
C(38)-C(33)-Si(1)	119.94(15)	C(38)-C(33)-C(34)	117.67(18)
C(33)-C(34)-H(34)	119.0	C(35)-C(34)-C(33)	121.95(18)
C(35)-C(34)-H(34)	119.0	C(34)-C(35)-C(39)	120.36(19)
C(36)-C(35)-C(34)	118.23(19)	C(36)-C(35)-C(39)	121.4(2)
C(35)-C(36)-H(36)	119.0	C(37)-C(36)-C(35)	122.0(2)
C(37)-C(36)-H(36)	119.0	C(36)-C(37)-C(38)	118.13(19)
C(36)-C(37)-C(40)	121.1(2)	C(38)-C(37)-C(40)	120.7(2)
C(33)-C(38)-H(38)	119.0	C(37)-C(38)-C(33)	122.01(19)
C(37)-C(38)-H(38)	119.0	C(35)-C(39)-H(39A)	109.5
C(35)-C(39)-H(39B)	109.5	C(35)-C(39)-H(39C)	109.5
H(39A)-C(39)-H(39B)	109.5	H(39A)-C(39)-H(39C)	109.5
H(39B)-C(39)-H(39C)	109.5	C(37)-C(40)-H(40A)	109.5
C(37)-C(40)-H(40B)	109.5	C(37)-C(40)-H(40C)	109.5
H(40A)-C(40)-H(40B)	109.5	H(40A)-C(40)-H(40C)	109.5
H(40B)-C(40)-H(40C)	109.5	C(42)-C(41)-Si(2)	121.05(14)
C(42)-C(41)-C(46)	117.88(18)	C(46)-C(41)-Si(2)	121.04(15)
C(41)-C(42)-H(42)	119.1	C(43)-C(42)-C(41)	121.83(18)
C(43)-C(42)-H(42)	119.1	C(42)-C(43)-C(47)	120.38(19)
C(44)-C(43)-C(42)	118.4(2)	C(44)-C(43)-C(47)	121.18(19)
C(43)-C(44)-H(44)	119.2	C(43)-C(44)-C(45)	121.60(19)
C(45)-C(44)-H(44)	119.2	C(44)-C(45)-C(48)	120.5(2)
C(46)-C(45)-C(44)	118.44(19)	C(46)-C(45)-C(48)	121.1(2)
C(41)-C(46)-H(46)	119.1	C(45)-C(46)-C(41)	121.8(2)
C(45)-C(46)-H(46)	119.1	C(43)-C(47)-H(47A)	109.5

C(43)-C(47)-H(47B)	109.5	C(43)-C(47)-H(47C)	109.5
H(47A)-C(47)-H(47B)	109.5	H(47A)-C(47)-H(47C)	109.5
H(47B)-C(47)-H(47C)	109.5	C(45)-C(48)-H(48A)	109.5
C(45)-C(48)-H(48B)	109.5	C(45)-C(48)-H(48C)	109.5
H(48A)-C(48)-H(48B)	109.5	H(48A)-C(48)-H(48C)	109.5
H(48B)-C(48)-H(48C)	109.5	C(50)-C(49)-Si(2)	121.82(16)
C(50)-C(49)-C(54)	118.05(18)	C(54)-C(49)-Si(2)	120.13(14)
C(49)-C(50)-H(50)	119.2	C(49)-C(50)-C(51)	121.6(2)
C(51)-C(50)-H(50)	119.2	C(50)-C(51)-C(55)	120.2(2)
C(52)-C(51)-C(50)	118.31(19)	C(52)-C(51)-C(55)	121.5(2)
C(51)-C(52)-H(52)	119.0	C(51)-C(52)-C(53)	122.05(19)
C(53)-C(52)-H(52)	119.0	C(52)-C(53)-C(56)	121.1(2)
C(54)-C(53)-C(52)	118.2(2)	C(54)-C(53)-C(56)	120.7(2)
C(49)-C(54)-H(54)	119.1	C(53)-C(54)-C(49)	121.75(19)
C(53)-C(54)-H(54)	119.1	C(51)-C(55)-H(55A)	109.5
C(51)-C(55)-H(55B)	109.5	C(51)-C(55)-H(55C)	109.5
H(55A)-C(55)-H(55B)	109.5	H(55A)-C(55)-H(55C)	109.5
H(55B)-C(55)-H(55C)	109.5	C(53)-C(56)-H(56A)	109.5
C(53)-C(56)-H(56B)	109.5	C(53)-C(56)-H(56C)	109.5
H(56A)-C(56)-H(56B)	109.5	H(56A)-C(56)-H(56C)	109.5
H(56B)-C(56)-H(56C)	109.5	C(58)-C(57)-Si(3)	122.81(14)
C(58)-C(57)-C(62)	117.84(18)	C(62)-C(57)-Si(3)	119.18(14)
C(57)-C(58)-H(58)	119.2	C(59)-C(58)-C(57)	121.66(18)
C(59)-C(58)-H(58)	119.2	C(58)-C(59)-C(63)	120.67(19)
C(60)-C(59)-C(58)	118.41(19)	C(60)-C(59)-C(63)	120.9(2)
C(59)-C(60)-H(60)	119.0	C(59)-C(60)-C(61)	121.9(2)
C(61)-C(60)-H(60)	119.0	C(60)-C(61)-C(64)	120.64(19)
C(62)-C(61)-C(60)	118.06(19)	C(62)-C(61)-C(64)	121.29(19)
C(57)-C(62)-H(62)	119.0	C(61)-C(62)-C(57)	122.06(18)
C(61)-C(62)-H(62)	119.0	C(59)-C(63)-H(63A)	109.5
C(59)-C(63)-H(63B)	109.5	C(59)-C(63)-H(63C)	109.5
H(63A)-C(63)-H(63B)	109.5	H(63A)-C(63)-H(63C)	109.5
H(63B)-C(63)-H(63C)	109.5	C(61)-C(64)-H(64A)	109.5
C(61)-C(64)-H(64B)	109.5	C(61)-C(64)-H(64C)	109.5
H(64A)-C(64)-H(64B)	109.5	H(64A)-C(64)-H(64C)	109.5
H(64B)-C(64)-H(64C)	109.5	C(66)-C(65)-Si(3)	121.80(14)

C(66)-C(65)-C(70)	117.47(17)	C(70)-C(65)-Si(3)	120.59(14)
C(65)-C(66)-H(66)	118.9	C(65)-C(66)-C(67)	122.12(19)
C(67)-C(66)-H(66)	118.9	C(66)-C(67)-C(71)	120.1(2)
C(68)-C(67)-C(66)	118.22(19)	C(68)-C(67)-C(71)	121.67(19)
C(67)-C(68)-H(68)	119.1	C(67)-C(68)-C(69)	121.72(18)
C(69)-C(68)-H(68)	119.1	C(68)-C(69)-C(72)	120.90(19)
C(70)-C(69)-C(68)	118.33(19)	C(70)-C(69)-C(72)	120.75(19)
C(65)-C(70)-H(70)	118.9	C(69)-C(70)-C(65)	122.11(18)
C(69)-C(70)-H(70)	118.9	C(67)-C(71)-H(71A)	109.5
C(67)-C(71)-H(71B)	109.5	C(67)-C(71)-H(71C)	109.5
H(71A)-C(71)-H(71B)	109.5	H(71A)-C(71)-H(71C)	109.5
H(71B)-C(71)-H(71C)	109.5	C(69)-C(72)-H(72A)	109.5
C(69)-C(72)-H(72B)	109.5	C(69)-C(72)-H(72C)	109.5
H(72A)-C(72)-H(72B)	109.5	H(72A)-C(72)-H(72C)	109.5
H(72B)-C(72)-H(72C)	109.5	C(74)-C(73)-N(2)	120.44(16)
C(74)-C(73)-C(78)	119.32(17)	C(78)-C(73)-N(2)	120.24(16)
C(73)-C(74)-H(74)	119.7	C(75)-C(74)-C(73)	120.59(17)
C(75)-C(74)-H(74)	119.7	C(74)-C(75)-C(79)	120.44(18)
C(76)-C(75)-C(74)	118.83(18)	C(76)-C(75)-C(79)	120.73(18)
C(75)-C(76)-H(76)	119.0	C(75)-C(76)-C(77)	121.95(18)
C(77)-C(76)-H(76)	119.0	C(76)-C(77)-C(78)	118.29(17)
C(76)-C(77)-C(80)	121.58(17)	C(78)-C(77)-C(80)	120.12(18)
C(73)-C(78)-H(78)	119.5	C(77)-C(78)-C(73)	120.99(18)
C(77)-C(78)-H(78)	119.5	C(75)-C(79)-H(79A)	109.5
C(75)-C(79)-H(79B)	109.5	C(75)-C(79)-H(79C)	109.5
H(79A)-C(79)-H(79B)	109.5	H(79A)-C(79)-H(79C)	109.5
H(79B)-C(79)-H(79C)	109.5	C(77)-C(80)-H(80A)	109.5
C(77)-C(80)-H(80B)	109.5	C(77)-C(80)-H(80C)	109.5
H(80A)-C(80)-H(80B)	109.5	H(80A)-C(80)-H(80C)	109.5
H(80B)-C(80)-H(80C)	109.5	N(2)-C(81)-C(82)	110.00(15)
N(2)-C(81)-C(83)	108.89(15)	N(2)-C(81)-C(84)	110.04(15)
C(83)-C(81)-C(82)	108.92(15)	C(83)-C(81)-C(84)	108.99(17)
C(84)-C(81)-C(82)	109.97(17)	C(81)-C(82)-H(82A)	109.5
C(81)-C(82)-H(82B)	109.5	C(81)-C(82)-H(82C)	109.5
H(82A)-C(82)-H(82B)	109.5	H(82A)-C(82)-H(82C)	109.5
H(82B)-C(82)-H(82C)	109.5	C(81)-C(83)-H(83A)	109.5

C(81)-C(83)-H(83B)	109.5	C(81)-C(83)-H(83C)	109.5
H(83A)-C(83)-H(83B)	109.5	H(83A)-C(83)-H(83C)	109.5
H(83B)-C(83)-H(83C)	109.5	C(81)-C(84)-H(84A)	109.5
C(81)-C(84)-H(84B)	109.5	C(81)-C(84)-H(84C)	109.5
H(84A)-C(84)-H(84B)	109.5	H(84A)-C(84)-H(84C)	109.5
H(84B)-C(84)-H(84C)	109.5	C(86)-C(85)-N(3)	120.68(17)
C(86)-C(85)-C(90)	118.76(18)	C(90)-C(85)-N(3)	120.56(17)
C(85)-C(86)-H(86)	119.3	C(85)-C(86)-C(87)	121.48(19)
C(87)-C(86)-H(86)	119.3	C(86)-C(87)-C(91)	120.8(2)
C(88)-C(87)-C(86)	118.30(19)	C(88)-C(87)-C(91)	120.9(2)
C(87)-C(88)-H(88)	119.2	C(87)-C(88)-C(89)	121.56(19)
C(89)-C(88)-H(88)	119.2	C(88)-C(89)-C(92)	120.60(18)
C(90)-C(89)-C(88)	118.80(19)	C(90)-C(89)-C(92)	120.59(19)
C(85)-C(90)-H(90)	119.5	C(89)-C(90)-C(85)	121.07(18)
C(89)-C(90)-H(90)	119.5	C(87)-C(91)-H(91A)	109.5
C(87)-C(91)-H(91B)	109.5	C(87)-C(91)-H(91C)	109.5
H(91A)-C(91)-H(91B)	109.5	H(91A)-C(91)-H(91C)	109.5
H(91B)-C(91)-H(91C)	109.5	C(89)-C(92)-H(92A)	109.5
C(89)-C(92)-H(92B)	109.5	C(89)-C(92)-H(92C)	109.5
H(92A)-C(92)-H(92B)	109.5	H(92A)-C(92)-H(92C)	109.5
H(92B)-C(92)-H(92C)	109.5	N(3)-C(93)-C(94)	110.68(15)
N(3)-C(93)-C(95)	108.79(15)	N(3)-C(93)-C(96)	110.04(16)
C(94)-C(93)-C(96)	108.57(16)	C(95)-C(93)-C(94)	108.57(17)
C(95)-C(93)-C(96)	110.18(18)	C(93)-C(94)-H(94A)	109.5
C(93)-C(94)-H(94B)	109.5	C(93)-C(94)-H(94C)	109.5
H(94A)-C(94)-H(94B)	109.5	H(94A)-C(94)-H(94C)	109.5
H(94B)-C(94)-H(94C)	109.5	C(93)-C(95)-H(95A)	109.5
C(93)-C(95)-H(95B)	109.5	C(93)-C(95)-H(95C)	109.5
H(95A)-C(95)-H(95B)	109.5	H(95A)-C(95)-H(95C)	109.5
H(95B)-C(95)-H(95C)	109.5	C(93)-C(96)-H(96A)	109.5
C(93)-C(96)-H(96B)	109.5	C(93)-C(96)-H(96C)	109.5
H(96A)-C(96)-H(96B)	109.5	H(96A)-C(96)-H(96C)	109.5
H(96B)-C(96)-H(96C)	109.5	C(98)-C(97)-N(4)	119.97(17)
C(102)-C(97)-N(4)	121.09(16)	C(102)-C(97)-C(98)	118.93(18)
C(97)-C(98)-H(98)	119.6	C(99)-C(98)-C(97)	120.84(19)
C(99)-C(98)-H(98)	119.6	C(98)-C(99)-C(103)	120.7(2)

C(100)-C(99)-C(98)	118.89(19)	C(100)-C(99)-C(103)	120.4(2)
C(99)-C(100)-H(100)	119.3	C(101)-C(100)-C(99)	121.4(2)
C(101)-C(100)-H(100)	119.3	C(100)-C(101)-C(102)	118.80(19)
C(100)-C(101)-C(104)	120.92(19)	C(102)-C(101)-C(104)	120.27(19)
C(97)-C(102)-C(101)	121.15(18)	C(97)-C(102)-H(102)	119.4
C(101)-C(102)-H(102)	119.4	C(99)-C(103)-H(10A)	109.5
C(99)-C(103)-H(10B)	109.5	C(99)-C(103)-H(10C)	109.5
H(10A)-C(103)-H(10B)	109.5	H(10A)-C(103)-H(10C)	109.5
H(10B)-C(103)-H(10C)	109.5	C(101)-C(104)-H(10D)	109.5
C(101)-C(104)-H(10E)	109.5	C(101)-C(104)-H(10F)	109.5
H(10D)-C(104)-H(10E)	109.5	H(10D)-C(104)-H(10F)	109.5
H(10E)-C(104)-H(10F)	109.5	N(4)-C(105)-C(106)	109.48(15)
N(4)-C(105)-C(107)	109.33(16)	N(4)-C(105)-C(108)	110.63(16)
C(107)-C(105)-C(106)	107.76(18)	C(108)-C(105)-C(106)	109.78(19)
C(108)-C(105)-C(107)	109.8(2)	C(105)-C(106)-H(10G)	109.5
C(105)-C(106)-H(10H)	109.5	C(105)-C(106)-H(10I)	109.5
H(10G)-C(106)-H(10H)	109.5	H(10G)-C(106)-H(10I)	109.5
H(10H)-C(106)-H(10I)	109.5	C(105)-C(107)-H(10J)	109.5
C(105)-C(107)-H(10K)	109.5	C(105)-C(107)-H(10L)	109.5
H(10J)-C(107)-H(10K)	109.5	H(10J)-C(107)-H(10L)	109.5
H(10K)-C(107)-H(10L)	109.5	C(105)-C(108)-H(10M)	109.5
C(105)-C(108)-H(10N)	109.5	C(105)-C(108)-H(10O)	109.5
H(10M)-C(108)-H(10N)	109.5	H(10M)-C(108)-H(10O)	109.5
H(10N)-C(108)-H(10O)	109.5	C(2A)-C(1A)-C(2B)	59.1(5)
C(2A)-C(1A)-C(7A)	117.1(3)	C(3B)-C(1A)-C(2A)	73.3(11)
C(3B)-C(1A)-C(2B)	54.1(6)	C(3B)-C(1A)-C(6A)	82.3(10)
C(3B)-C(1A)-C(7A)	115.2(7)	C(4B)-C(1A)-C(2A)	88.1(8)
C(4B)-C(1A)-C(2B)	130.2(8)	C(4B)-C(1A)-C(3B)	82.6(9)
C(4B)-C(1A)-C(6A)	141.3(8)	C(4B)-C(1A)-C(7A)	39.2(7)
C(6A)-C(1A)-C(2A)	120.8(3)	C(6A)-C(1A)-C(2B)	62.8(5)
C(6A)-C(1A)-C(7A)	122.2(3)	C(7A)-C(1A)-C(2B)	168.8(5)
H(1BA)-C(1B)-H(1BB)	109.5	H(1BA)-C(1B)-H(1BC)	109.5
H(1BB)-C(1B)-H(1BC)	109.5	C(2B)-C(1B)-H(1BA)	109.5
C(2B)-C(1B)-H(1BB)	109.5	C(2B)-C(1B)-H(1BC)	109.5
C(4A)-C(1B)-H(1BA)	73.0	C(4A)-C(1B)-H(1BB)	111.5
C(4A)-C(1B)-H(1BC)	135.1	C(4A)-C(1B)-C(2B)	38.6(9)

C(4A)-C(1B)-C(5A)	97.3(15)	C(5A)-C(1B)-H(1BA)	161.0
C(5A)-C(1B)-H(1BB)	89.2	C(5A)-C(1B)-H(1BC)	65.6
C(5A)-C(1B)-C(2B)	58.8(10)	C(1A)-C(2A)-H(2A)	120.4
C(1A)-C(2A)-C(2B)	74.3(5)	C(1A)-C(2A)-C(3B)	48.5(8)
C(1A)-C(2A)-C(4B)	35.4(4)	C(2B)-C(2A)-H(2A)	161.5
C(2B)-C(2A)-C(4B)	102.1(6)	C(3A)-C(2A)-C(1A)	119.1(3)
C(3A)-C(2A)-H(2A)	120.4	C(3A)-C(2A)-C(2B)	46.4(5)
C(3A)-C(2A)-C(3B)	95.6(5)	C(3A)-C(2A)-C(4B)	147.9(5)
C(3B)-C(2A)-H(2A)	123.8	C(3B)-C(2A)-C(2B)	55.6(5)
C(3B)-C(2A)-C(4B)	53.6(5)	C(4B)-C(2A)-H(2A)	88.2
C(1A)-C(2B)-H(2BA)	129.1	C(1A)-C(2B)-H(2BB)	65.6
C(1B)-C(2B)-C(1A)	130.2(13)	C(1B)-C(2B)-C(2A)	147.3(14)
C(1B)-C(2B)-H(2BA)	100.3	C(1B)-C(2B)-H(2BB)	100.3
C(1B)-C(2B)-C(6A)	91.3(12)	C(2A)-C(2B)-C(1A)	46.6(4)
C(2A)-C(2B)-H(2BA)	88.6	C(2A)-C(2B)-H(2BB)	47.0
C(2A)-C(2B)-C(6A)	89.6(7)	H(2BA)-C(2B)-H(2BB)	104.3
C(3A)-C(2B)-C(1A)	100.8(9)	C(3A)-C(2B)-C(1B)	107.0(14)
C(3A)-C(2B)-C(2A)	55.4(6)	C(3A)-C(2B)-H(2BA)	51.2
C(3A)-C(2B)-H(2BB)	53.1	C(3A)-C(2B)-C(3B)	107.0(13)
C(3A)-C(2B)-C(5A)	141.7(12)	C(3A)-C(2B)-C(6A)	138.9(11)
C(3B)-C(2B)-C(1A)	41.5(8)	C(3B)-C(2B)-C(1B)	146.0(17)
C(3B)-C(2B)-C(2A)	59.6(10)	C(3B)-C(2B)-H(2BA)	100.3
C(3B)-C(2B)-H(2BB)	100.3	C(3B)-C(2B)-C(6A)	62.8(11)
C(4A)-C(2B)-C(1A)	150.2(12)	C(4A)-C(2B)-C(1B)	32.3(10)
C(4A)-C(2B)-C(2A)	128.7(12)	C(4A)-C(2B)-H(2BA)	72.7
C(4A)-C(2B)-H(2BB)	91.1	C(4A)-C(2B)-C(3A)	76.7(10)
C(4A)-C(2B)-C(3B)	167.9(16)	C(4A)-C(2B)-C(5A)	72.2(8)
C(4A)-C(2B)-C(6A)	122.1(11)	C(5A)-C(2B)-C(1A)	95.3(8)
C(5A)-C(2B)-C(1B)	39.9(9)	C(5A)-C(2B)-C(2A)	137.0(11)
C(5A)-C(2B)-H(2BA)	133.9	C(5A)-C(2B)-H(2BB)	105.3
C(5A)-C(2B)-C(3B)	108.1(14)	C(5A)-C(2B)-C(6A)	52.6(6)
C(6A)-C(2B)-C(1A)	43.6(4)	C(6A)-C(2B)-H(2BA)	161.1
C(6A)-C(2B)-H(2BB)	88.1	C(2A)-C(3A)-H(3A)	119.9
C(2A)-C(3A)-C(4A)	120.3(3)	C(2B)-C(3A)-C(2A)	78.2(7)
C(2B)-C(3A)-H(3A)	156.7	C(2B)-C(3A)-C(4A)	44.7(6)
C(4A)-C(3A)-H(3A)	119.9	C(1A)-C(3B)-C(2A)	58.2(9)

C(1A)-C(3B)-C(2B)	84.4(12)	C(1A)-C(3B)-H(3BA)	104.2
C(1A)-C(3B)-H(3BB)	141.6	C(1A)-C(3B)-C(4B)	41.1(7)
C(1A)-C(3B)-C(6A)	51.4(9)	C(2A)-C(3B)-H(3BA)	160.1
C(2A)-C(3B)-H(3BB)	93.0	C(2A)-C(3B)-C(6A)	93.2(10)
C(2B)-C(3B)-C(2A)	64.7(9)	C(2B)-C(3B)-H(3BA)	107.1
C(2B)-C(3B)-H(3BB)	107.1	C(2B)-C(3B)-C(6A)	64.6(10)
H(3BA)-C(3B)-H(3BB)	106.8	C(4B)-C(3B)-C(2A)	66.7(9)
C(4B)-C(3B)-C(2B)	120.9(15)	C(4B)-C(3B)-H(3BA)	107.1
C(4B)-C(3B)-H(3BB)	107.1	C(4B)-C(3B)-C(6A)	86.9(12)
C(6A)-C(3B)-H(3BA)	67.2	C(6A)-C(3B)-H(3BB)	166.0
C(1B)-C(4A)-C(2B)	109.2(16)	C(1B)-C(4A)-C(3A)	157.7(16)
C(1B)-C(4A)-H(4A)	77.7	C(1B)-C(4A)-C(5A)	44.8(14)
C(2B)-C(4A)-C(3A)	58.5(8)	C(2B)-C(4A)-H(4A)	159.9
C(2B)-C(4A)-C(5A)	64.4(8)	C(3A)-C(4A)-H(4A)	120.5
C(3A)-C(4A)-C(5A)	118.9(4)	C(5A)-C(4A)-H(4A)	120.5
C(1A)-C(4B)-C(2A)	56.4(6)	C(1A)-C(4B)-C(3B)	56.4(10)
C(1A)-C(4B)-H(4BA)	55.9	C(1A)-C(4B)-H(4BB)	101.7
C(1A)-C(4B)-C(5B)	149.8(14)	C(1A)-C(4B)-C(7A)	105.2(11)
C(2A)-C(4B)-H(4BA)	99.4	C(2A)-C(4B)-H(4BB)	53.7
C(3B)-C(4B)-C(2A)	59.7(10)	C(3B)-C(4B)-H(4BA)	107.8
C(3B)-C(4B)-H(4BB)	107.8	C(3B)-C(4B)-C(5B)	118.0(13)
H(4BA)-C(4B)-H(4BB)	107.1	C(5B)-C(4B)-C(2A)	151.3(12)
C(5B)-C(4B)-H(4BA)	107.8	C(5B)-C(4B)-H(4BB)	107.8
C(7A)-C(4B)-C(2A)	139.1(10)	C(7A)-C(4B)-C(3B)	145.0(15)
C(7A)-C(4B)-H(4BA)	49.6	C(7A)-C(4B)-H(4BB)	104.9
C(7A)-C(4B)-C(5B)	61.3(10)	C(1B)-C(5A)-C(2B)	81.3(10)
C(1B)-C(5A)-C(4A)	37.8(11)	C(1B)-C(5A)-H(5A)	83.8
C(1B)-C(5A)-C(6A)	153.8(13)	C(2B)-C(5A)-C(4A)	43.5(6)
C(2B)-C(5A)-H(5A)	157.0	C(2B)-C(5A)-C(6A)	78.5(7)
C(4A)-C(5A)-H(5A)	120.2	C(6A)-C(5A)-C(4A)	119.7(4)
C(6A)-C(5A)-H(5A)	120.2	C(4B)-C(5B)-H(5BA)	109.5
C(4B)-C(5B)-H(5BB)	109.5	C(4B)-C(5B)-H(5BC)	109.5
H(5BA)-C(5B)-H(5BB)	109.5	H(5BA)-C(5B)-H(5BC)	109.5
H(5BB)-C(5B)-H(5BC)	109.5	C(7A)-C(5B)-C(4B)	43.7(6)
C(7A)-C(5B)-H(5BA)	85.8	C(7A)-C(5B)-H(5BB)	153.1
C(7A)-C(5B)-H(5BC)	84.5	C(1A)-C(6A)-C(2B)	73.6(5)

C(1A)-C(6A)-C(3B)	46.3(6)	C(1A)-C(6A)-C(5A)	121.2(3)
C(1A)-C(6A)-H(6A)	119.4	C(2B)-C(6A)-H(6A)	163.0
C(3B)-C(6A)-C(2B)	52.5(5)	C(3B)-C(6A)-H(6A)	127.1
C(5A)-C(6A)-C(2B)	48.9(5)	C(5A)-C(6A)-C(3B)	95.7(5)
C(5A)-C(6A)-H(6A)	119.4	C(1A)-C(7A)-H(7AA)	109.5
C(1A)-C(7A)-H(7AB)	109.5	C(1A)-C(7A)-H(7AC)	109.5
C(4B)-C(7A)-C(1A)	35.7(7)	C(4B)-C(7A)-C(5B)	75.0(9)
C(4B)-C(7A)-H(7AA)	125.8	C(4B)-C(7A)-H(7AB)	73.9
C(4B)-C(7A)-H(7AC)	120.3	C(5B)-C(7A)-C(1A)	106.3(8)
C(5B)-C(7A)-H(7AA)	136.2	C(5B)-C(7A)-H(7AB)	32.8
C(5B)-C(7A)-H(7AC)	80.6	H(7AA)-C(7A)-H(7AB)	109.5
H(7AA)-C(7A)-H(7AC)	109.5	H(7AB)-C(7A)-H(7AC)	109.5
